# Stereoselective Fe-Catalyzed Decoupled Cross-Couplings: Chiral Vinyl Oxazolidinones as Effective Radical Lynchpins for Diastereoselective C(sp^2^)–C(sp^3^) Bond Formation

**DOI:** 10.1021/acscatal.4c04568

**Published:** 2024-08-15

**Authors:** Tapas Maity, Ángel Rentería-Gómez, Osvaldo Gutierrez

**Affiliations:** Department of Chemistry, Texas A&M University, College Station, Texas 77843, United States

**Keywords:** iron, cross-couplings, radicals, catalysis, diastereoselective, difunctionalization

## Abstract

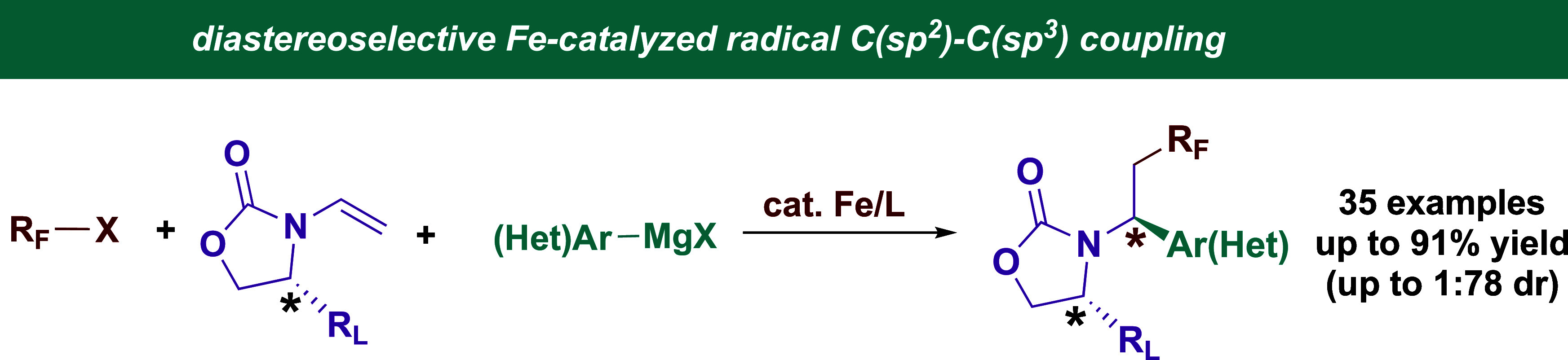

Modular, catalytic, and stereoselective methods for the dicarbofunctionalization of alkenes can streamline the synthesis of chiral active pharmaceutical ingredients (APIs) and agrochemicals. However, despite the inherent attractive properties of iron as catalysts for practical pharmaceutical synthesis (i.e., less expensive, more abundant, less toxic, and lower carbon footprint in comparison to other transition metals), iron-based catalytic methods that enable highly stereoselective dicarbofunctionalization of alkenes are lacking. Herein, we report the use of readily available chiral vinyl oxazolidinones as effective chiral radical lynchpins to enable practical and diastereoselective (up to 1:78 dr) Fe-catalyzed dicarbofunctionalization with fluoroalkyl halides and hetero(aryl) Grignard reagents. Experimental and computational mechanistic studies are carried out to elucidate the origin of stereoinduction and to build a stereochemical model for the rational reaction design.

## Introduction

Alkenes are versatile building blocks in chemical synthesis.^1^ Driven by the pharmaceutical industry’s drive to “escape from flatland” by developing practical synthetic methods by increasing carbon bond saturation and/or installation of new chiral centers in drug candidates,^2^ transition metal-catalyzed three-component dicarbofunctionalization (DCF) of alkenes has emerged as an effective and highly versatile strategy.^3^ In pharmaceutical synthesis, methods for the selective installation of new C(sp^3^)–fluoroalkyl bonds are highly desirable due to the well-known ability of the difluoro methylene group (−CF_2_−)^4^ to improve lipophilicity, bioavailability, and metabolic stability of biologically active molecules. Notably, in some cases, molecules that bear C(sp^3^)–CF_2_R motifs have shown even more desirable properties than those with more traditional and “flat-like” C(sp^2^)–CF_3_ and C(sp^2^)–CF_2_H bonds.^5^

However, the use of sp^3^-hybridized coupling partners, especially sp^3^-fluoroalkyls, in transition metal-catalyzed three-component DCF of alkenes still faces significant hurdles for practical applications. For example, palladium catalysts have been extensively used in this context.^3,6^ However, the high cost, increased carbon footprint, and localized global mining access of palladium coupled with slow rates for oxidative addition to sp^3^-hybridized electrophiles and facile β-H elimination can limit their broad applicability especially in large-scale pharmaceutical synthesis.^6b^ Recently, nickel- and copper-based catalytic systems have emerged as potentially more sustainable alternative and complementary strategies to enable selective DCF of alkenes using sp^3^-hybridized electrophiles or nucleophiles.^7,8^ Nonetheless, in contrast to other transition metals, iron is less expensive, more abundant, and less toxic and produces significantly less carbon footprint. These attractive properties make iron a highly desirable catalyst for practical pharmaceutical synthesis.^9,10^ In this vein, our group has pioneered the use of inexpensive iron salts in combination with commercially available ligands to enable selective three-component DCF of alkenes using alkyl halides and sp^2^-hybridized Grignard reagents as coupling partners.^11,12^

**Scheme 1:**
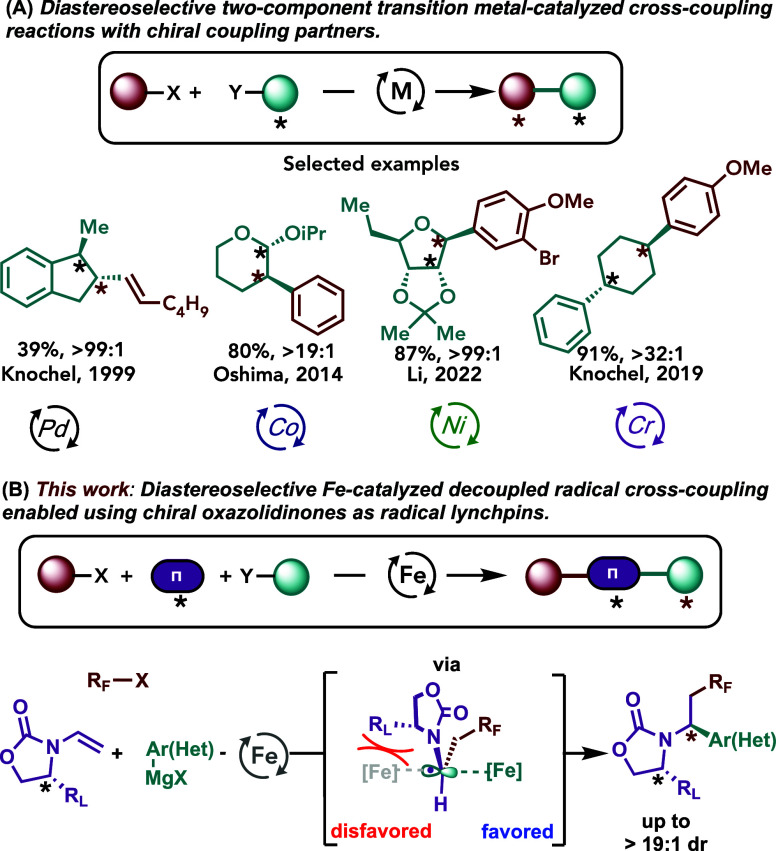
Diastereoselective Cross-Coupling Reactions Using a Chiral Substrate

In contrast to their two-component variants, methods for stereoselective transition metal-catalyzed *three*-component cross-couplings with alkenes are severely limited.^7c^ In this context, although the use of Evan’s chiral oxazolidinones in practical asymmetric C–C bond formations in both academic and industrial settings is well-established,^13^ the complementary strategy that uses chiral (enantiopure) coupling partners to control the diastereoselectivity in transition-metal catalyzed DCF of alkenes is underdeveloped. Notably, there are sporadic methods that use enantiopure chiral coupling partners to control the diastereoselectivity in transition metal-catalyzed “two-component” cross-coupling reactions (Scheme 1A).^14^ Specifically, Knochel and co-workers reported the diastereoselective Pd-catalyzed cross-coupling of cyclic and open chain chiral zinc reagents with alkenyl iodides and alkyl chlorides albeit low yields.^14a^ Oshima reported a cobalt-catalyzed diastereoselective cross-coupling between aryl Grignard reagents and optically pure bromo acetals.^14b^ Under nickel catalysis, the Li group disclosed a cross-electrophile diastereoselective coupling to form a series of aryl-nucleosides.^14c^ Finally, under chromium catalysis, the Knochel group reported a method for highly chemo- and diastereoselective Csp^2^–Csp^3^ cross-couplings.^14d^ Herein, we report the development of the first highly diastereoselective and broadly applicable three-component DCF of alkenes using a bisphosphine-iron-catalyzed decoupled radical cross-coupling strategy using readily available chiral oxazolidinones with alkyl halides and (hetero)aryl Grignard reagents (Scheme 1B).

## Results and Discussion

To initiate our studies, we selected chiral vinyl oxazolidinones as commercially available and modular α-amide radical precursors for Fe-catalyzed decoupled cross-coupling reactions. The basis for the choice of chiral alkene originates from (1) our recent report on DCF of enamides,^15^ (2) the wide use of Evans’ chiral auxiliaries in organic chemistry for practical and stereoselective construction of enantioenriched compounds,^16^ and (3) potential application in medicinal chemistry as oxazolidinones represent an important class of heterocyclic motifs found in many natural products, pharmaceuticals, and biologically active molecules.^17^ Moreover, the use of enantioenriched alkenes, including vinyl oxazolidinones, in three-component transition-metal catalyzed DCF of alkenes remains virtually unexplored.^18^ In this vein, we hypothesized that incorporating this chiral functionality into an alkene could be an alternative and practical strategy to achieve high stereocontrol of the C(sp^3^)–C(sp^2^) bond formation between iron-aryl species and alkyl radical, which remains a grand challenge in Fe-catalyzed radical cross-couplings.^12a^

**Scheme 2:**
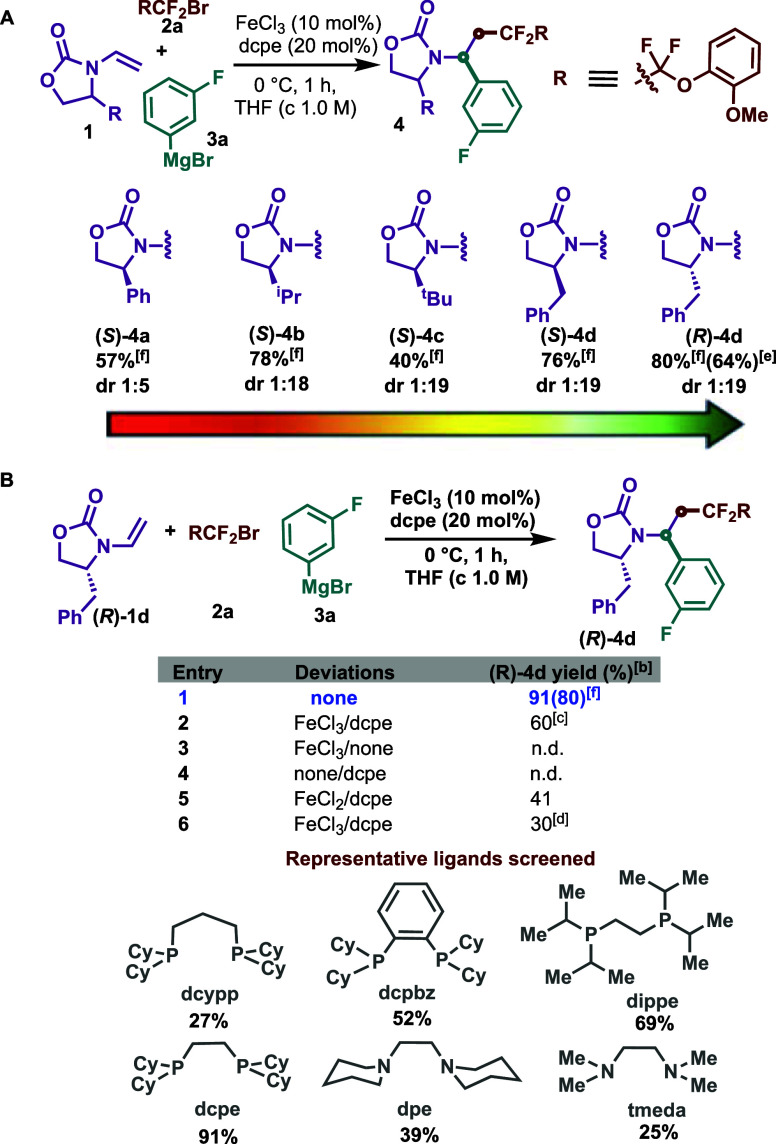
(A) Screening of the Chiral Substrate; (B) Deviations in the Optimized Reaction Conditions^,^^,^^,^^,^^,^ Reaction conditions: **1** (0.2 mmol, 1.0 equiv), **2a** (0.4 mmol, 2.0 equiv), FeCl_3_ (10 mol %), dcpe (20 mol %), THF (*c* 1.0 M), 0 °C, slow addition of **3a** (0.8 mmol, 4.0 equiv) in 1 h, and nitrogen atmosphere; d.r. was determined by crude ^1^H NMR. Determined by ^1^H NMR using 1,2-dibromomethane as an internal standard. 0.4 mmol **3a** was used. FeCl_3_ (5 mol %), dcpe (10 mol %). yield (%) of isolated product of 2 mmol scale reaction. Isolated yield (%).

Gratifyingly, after extensive screening (Supporting Information), we identified 4-benzyl-3-vinyloxazolidin-2-one (*R*)-**4d** bearing a benzyl substituent to give the highest diastereomeric ratio (dr 1:19) and good isolated yield of the desired product **4d** (Scheme 2A). Notably, vinyloxazolidinone (**4b**) also gave good yields and high levels of diastereoselectivity. Other systems such as phenyl- and ^*t*^Bu-substituted oxazolidinone (**4a** and **4c**) gave inferior results in terms of yield and/or selectivity. With the best chiral substrate in hand, we next examined the impact of the ligand on this diastereoselective decoupled cross-coupling (Scheme 2B). As in the past, the combination of iron(III) chloride with privileged 1,2-bis(dicyclohexylphosphino) ethane (dcpe) ligand gave the best NMR yield (91%).^15^ Notably, the use of 1,2-bis(diisopropylphosphino)ethane (dippe) provided the desired product but in lower yields (69%). Interestingly, bidentate nitrogen-containing ligands also worked in our system (25–39%). Additional control experiments (entries 3–4) confirmed that both iron precatalyst and ligand are essential to promote this transformation (entries 3–5).^11b^ Lowering the catalyst and ligand loadings (from 10/20 to 5/10 mol %, entry 6) or modifying the aryl Grignard reagent (entry 2) resulted in lower yields. We note that the reaction proceeded smoothly at a larger scale (2.0 mmol), forming the desired product (*R*)-**4d** in 64% isolated yield.

**Scheme 3:**
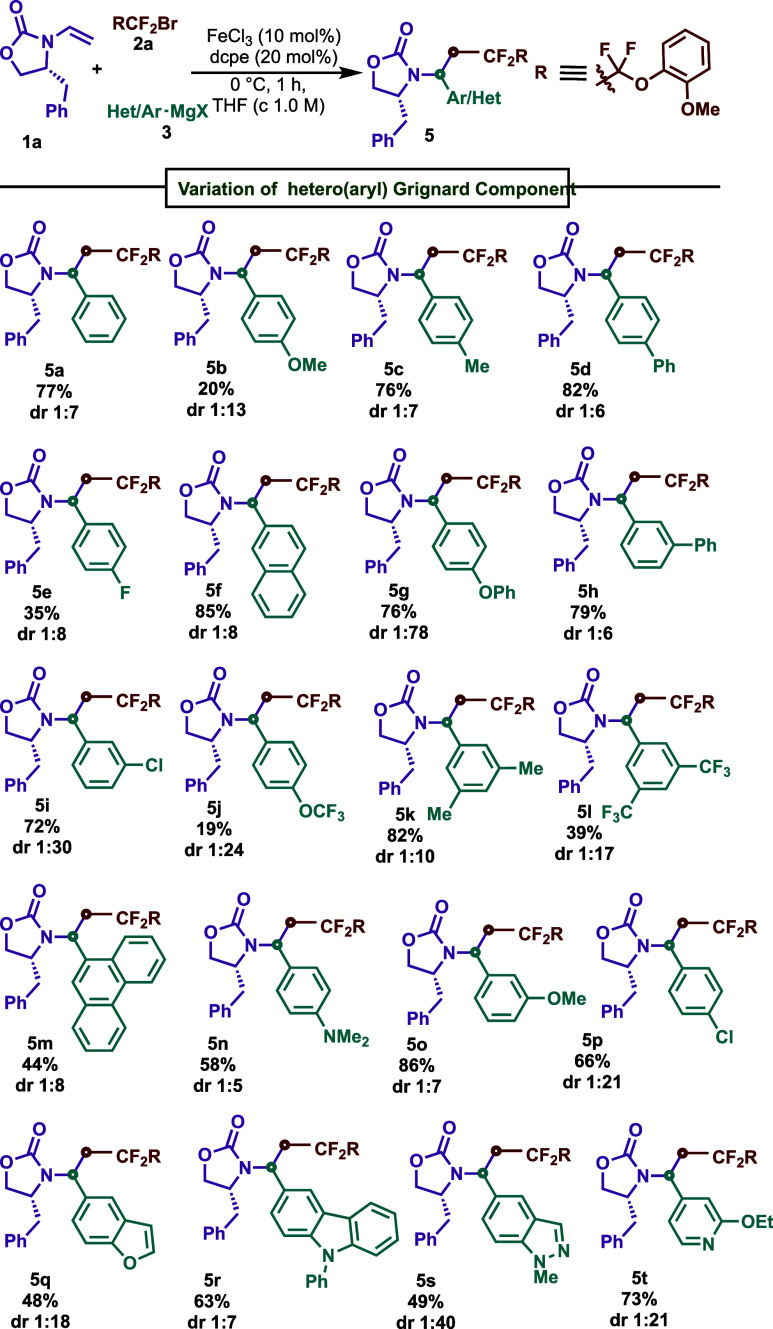
Scope of the Aryl Grignard Reagents for DS-CCR Conditions: **1a** (0.2 mmol), **2a** (0.4 mmol), **3** (0.8 mmol), FeCl_3_(10 mol %), dcpe (20 mol %), THF (*c* 1.0 M), 0 °C, slow addition of **3** in 1 h, argon atmosphere, and d.r. was determined by crude ^1^H NMR.

With optimized conditions in hand, we next examined the generality of this diastereoselective radical cross-coupling reaction in terms of the nucleophile scope (Scheme 3). Notably, a wide range of aryl Grignard reagents were able to deliver the intended cross-coupling product with fluoroalkyl bromide **2a** and (*R*)-4-benzyl-3-vinyloxazolidin-2-one **1a**. For example, electron-poor and -rich substituents were also well tolerated in different positions with varying yields. Electron-donating groups, including -OMe (**5b** and **5o**), –OCF_3_ (**5j**), and –F (**5e**) at the *meta* position were better suited for our conditions than at the *para* position. Furthermore, π-extended systems were also engaged with good efficiency with a high to moderate yield (85%, **5f**, and 44%, **5m**). *N*-/O- heterocycles, including carbazole (**5r**), indazole (**5s**), pyridine(**5t**), and benzofuran (**5q**), were also effective in conjunction with “turbo Grignard”, leading to *bis*-heterocycles in good yields (48–73%).^19^

**Scheme 4:**
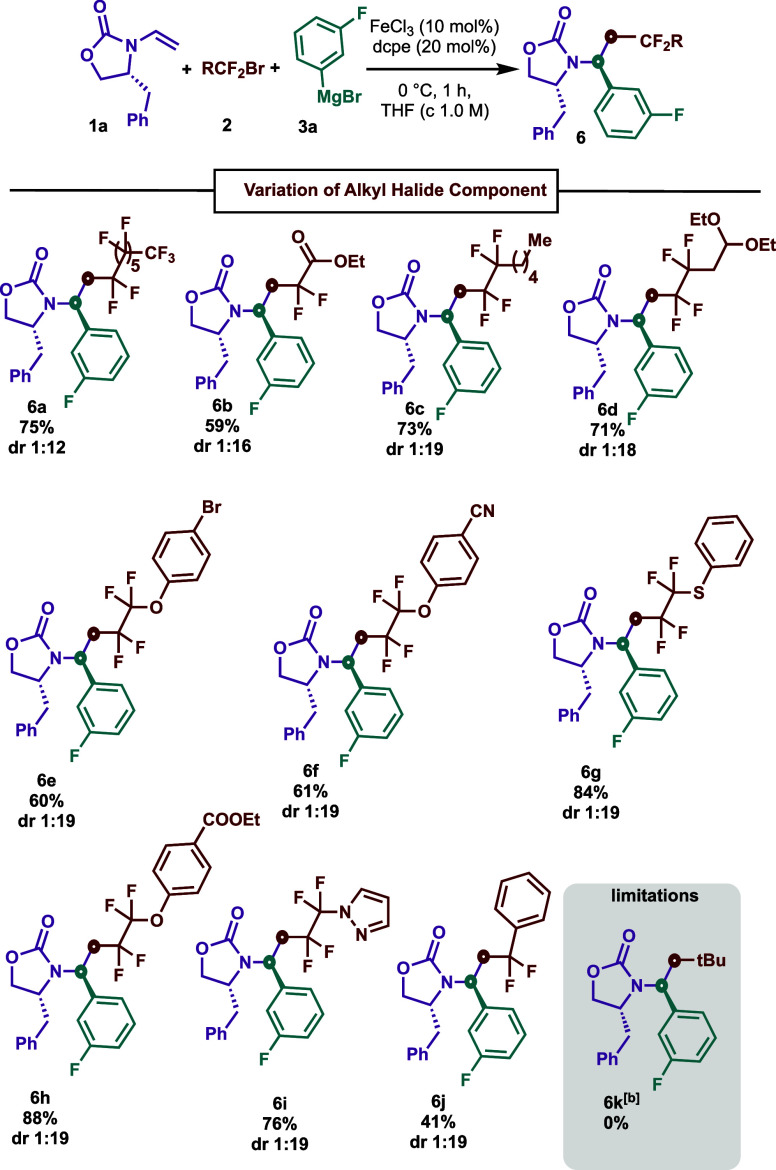
Scope of Fluoroalkyl Halides for DS-CCR^,^ Conditions: **1a** (0.2 mmol), **2** (0.4 mmol), **3a** (0.8 mmol), FeCl_3_(10 mol %), dcpe (20 mol %), THF (*c* 1.0 M), 0 °C, slow addition of **3a** in 1 h, and argon atmosphere. *tert*-butyl iodide was used, and d.r. was determined by crude ^1^H NMR.

We next proceeded to assess the substrate scope in terms of fluoroalkyl halides as radical precursors (Scheme 4). Overall, a broad spectrum of di-, tetra-, and perfluoro alkyl bromides as radical precursors carrying varied functionalities such as fluoroalkyl-rich (**6a**) chains, extended alkyls (**6c**), aryl (**6j**), aryl ethers with relevant functionalities (**6e**–**h**), heteroaryl (**6i**), and diethoxyalkyl as protected aldehydes (**6d**) were compatible in this transformation. Notably, an α,α-difluoro ester bromide interacts in the reaction to obtain **6b** in reasonable yield (59%). Not surprisingly, nucleophilic alkyl radicals did not participate in this iron-catalyzed three-component cross-coupling reaction (**6k**), consistent with the high energy barrier associated for polarity mismatched^20^ radical addition to electron-rich alkene.

**Scheme 5:**
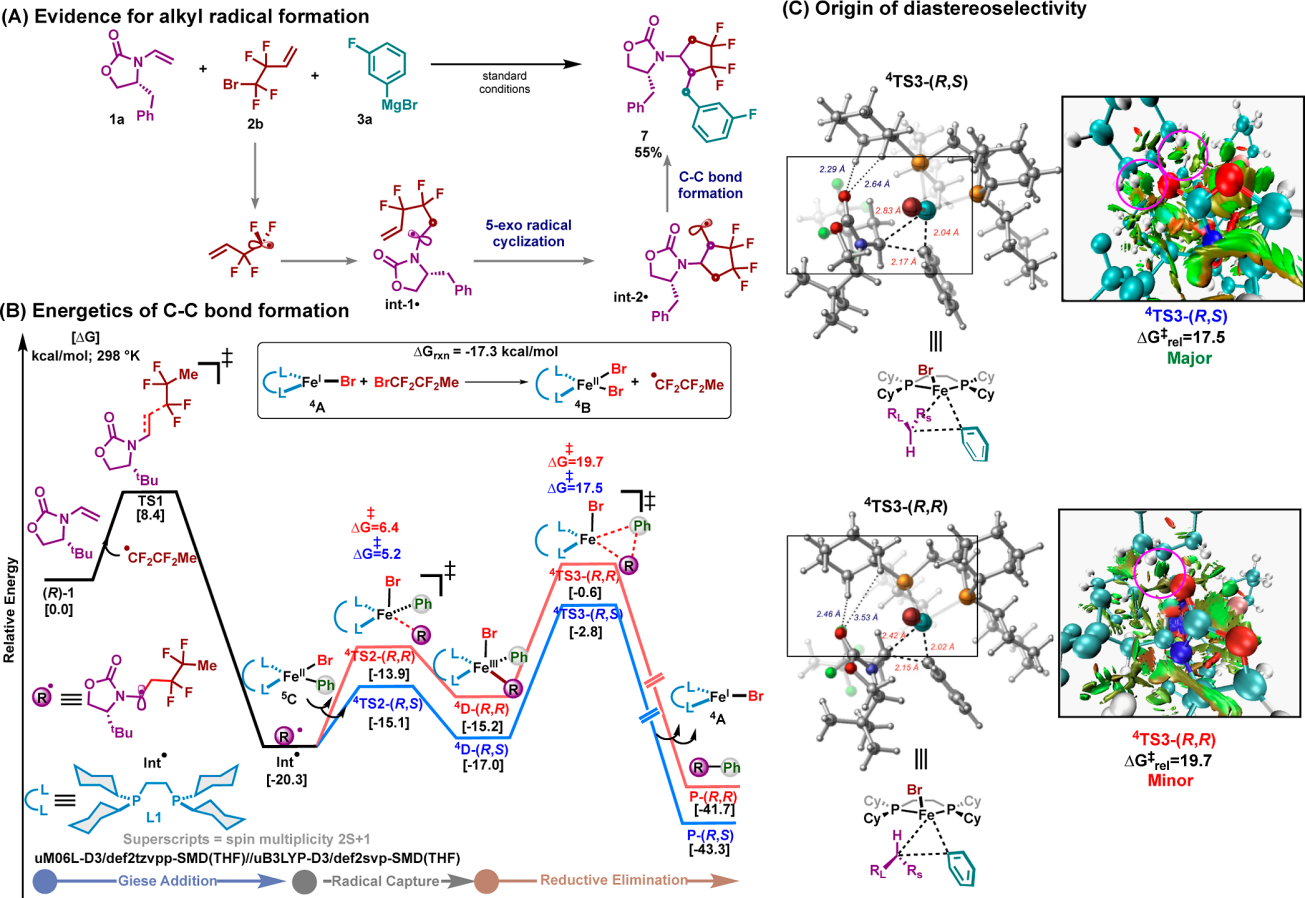
Mechanistic Studies

Finally, to confirm the formation of the Int-1 radical, we used tetrafluoroalkyl halide **2b** with a pendent alkene as a “radical trap” (Scheme 5A). Under our optimized reaction conditions, this reagent led to the formation of cyclic compound **7** in 55% yield. Based on these results and previous reports,^10b,11b,12,15^ a plausible mechanism is shown in Scheme 5B. Fe(I) **A** can undergo halogen-atom abstraction to form the radical **MeCF**_**2**_**CF**_**2**_^●^ and Fe (II) **B** species (energetically feasible by −17.3 kcal/mol).^11b,12c^ Then, **MeCF**_**2**_**CF**_**2**_^●^ can escape the solvent cage to undergo radical addition to *N*-vinyl compound (*R*)-**1** to form **Int**^**●**^ (downhill in 20.3 kcal/mol via a low energy barrier of 8.4 kcal/mol) with the concomitant formation of Fe(II) **B**. Simultaneously, the gradual addition of Grignard reagent can facilitate selective monotransmetalation of **B** into **C**.^11b^ Finally, **Int**^**●**^ will selectively and reversibly engage in radical addition to monoaryl Fe(II) **C** to form Fe(III) **D** that can then undergo reductive elimination to form the desired product. Consistent with the experiment, the energy difference between the two lowest energy diastereomeric transition states for the reductive elimination step is ∼2.2 kcal/mol (Scheme 5C) in favor of the (*R*,*S*) diastereomer.^18^ Closer inspection of the lowest energy diastereomeric transition states revealed an attractive C–H···O^12a^ interaction in ^**4**^**TS3-**(*R*,*S*), which is absent in the competing transition state, as evident from the NCI (noncovalent interaction) plots. In addition, upon interaction with the cyclohexyl group, both transition states exhibit a considerable difference in CH–Fe distance (2.83 Å vs 2.42 Å). This could promote ^**4**^**TS3**-(*R*,*S*) to undergo C–C bond formation faster over ^**4**^**TS3**-(*R*,*R*).

## Conclusions

In conclusion, we have developed the first highly diastereoselective bisphosphine-iron-catalyzed radical cross-coupling reaction to enable 1,2-dicarbofunctionalization of chiral vinyl oxazolidinones with diverse (fluoro)alkyl halides and aryl Grignard reagents. Mechanistic probes are consistent with radical translocation to form an α-amide radical that is rapidly intercepted by a monoaryl bisphosphine-iron leading to diastereoselective carbon–carbon bond formation. Further studies to develop catalytic asymmetric synthesis using this strategy are in progress in our laboratory and will be reported in due course.

## Supporting Information Available

Experimental procedures, product characterizations, DFT calculation data, and copies of the ^1^H and ^13^C NMR spectra (PDF)
