# Supplementary material for: Stereoselective Fe-Catalyzed Decoupled Cross-Couplings: Chiral Vinyl Oxazolidinones as Effective Radical Lynchpins for Diastereoselective C(sp2)–C(sp3) Bond Formation
Source: ACS Catal. 2024 Aug 15;14(17):13049–54. doi: 10.1021/acscatal.4c04568 (PMC11385348; doi:10.1021/acscatal.4c04568)
Supplement: Supplementary file 1 — cs4c04568_si_001.pdf [file cs4c04568_si_001.pdf]

Stereoselective Fe-catalyzed Decoupled Cross-couplings: Chiral vinyl oxazolidinones as effective radical lynchpins for diastereoselective C(sp^2)-C(sp^3) bond formation

Tapas Maity,‡ Ángel Rentería-Gómez,‡ Osvaldo Gutierrez*

Department of Chemistry, Texas A&M University, College Station, Texas 77843, United States.
*Correspondence to: og.labs@tamu.edu

Table of Contents

1. General Considerations S1
2. General Procedure1 for preparation of 3-vinyloxazolidin-2-one derivatives S2
3. General Procedure 2 for Iron-Catalyzed Multicomponent Cross-Coupling Reaction S2
4. Analysis of diastereoisomers S3
5. Computational Details S5
6. Limitations S6
7. Grignard reagent optimization table S7
8. Product Characterization Data S8
9. Reference S26
10. Spectral Data S28
11. Cartesian Coordinates S101

1. General Considerations.

Unless otherwise indicated, all reactions were carried out under a nitrogen atmosphere in oven (-110 °C) or flame-dried glassware. When necessary, solvents and reagents were dried prior to use. Tetrahydrofuran (THF) was dried by passage through activated alumina in Inert's PureSolv MB-SPS solvent purification system. All solvents were obtained from VWR, Sigma-Aldrich, or Fisher. Organometallic reagents were purchased from Sigma-Aldrich and Synthonix. Silicycle 250 μm silica-gel F-254 plates were used to perform analytical thin layer chromatography (TLC). Column chromatography was performed with Silica gel (230-400 mesh) and Biotage® Selekt Flash Systems silica gel chromatography was performed with prepacked silica-gel cartridges (Sfar; Biotage). NMR (^{1}H, ^{13}C and ^{19}F) spectra were recorded on Acsend TM 400 (Bruker) NMR spectrometer. Chemical shifts (δ) are reported in parts per million (ppm) relative to the internal residual solvent resonance peak δ 7.26 ($CDCl_3$) and δ 0.00 (TMS) for all ^{1}H and δ 77.16 ($CDCl_3$) and δ 0.00 (TMS) for all ^{13}C. Other data are designated as follows: multiplicity (s = singlet, d = doublet, t = triplet, q = quartet, qu = quintet, sept = septet, oct = octet, m = multiplet, dd= doublet of doublets, dt = doublet of triplets, td = triplet of doublets, dq = doublet of quartets, qd = quartet of doublets, tt = triplet of triplets, tdd = triplet of doublet of doublets, bs = broad singlet), coupling constants (*J*) are reported in Hertz (Hz), and number of protons. Electrospray ionization (ESI) and atmospheric chemical ionization (APCI) mass spectrometry experiments were performed using a Thermo Scientific Q Exactive Focus. Sample was loop injected (10 μL) and methanol was used as a mobile solvent at a flow rate of 600 μL/min. The Q Exactive Focus HESI source was operated in full MS in positive mode. The mass resolution was tuned to 70000 FWHM at m/z 200. The electrospray spray voltage was set to 3.5 kV and for APCI the discharge current was set at 5 μA. The sheath gas and auxiliary gas flow rates were set to 40 and 10 arbitrary units, respectively. The transfer capillary temperature was held at 320 °C and the S-Lens RF level was set at 50 v. Exactive Series 2.11 /Xcalibur 4.2.47 software was used for data acquisition and processing. Thermo Scientific Nicolet Summit FTIR spectrometer was employed for recording IR spectra and are reported in wavenumbers (cm^{-1}). General Procedure 1 for preparation of 3-vinyloxazolidin-2-one derivatives.[1]

2. General Procedure1 for preparation of 3-vinyloxazolidin-2-one derivatives

A mixture of amide **A** (3.9 mmol, 1.0 equiv), vinyl iodide **B** (7.9 mmol, 2.0 equiv), *N,N*-dimethylethylenediamine (0.4 mmol, 0.1 equiv), K_2CO_3 (2.2 mmol, 0.55 equiv), CuI (0.20 mmol, 0.05 equiv), and toluene (1.2 mL) was placed in a tightly closed sealed tube fitted with a magnetic stir bar. The mixture was stirred for 16 hours at 110°C in an oil bath and then cooled to room temperature. The tube was carefully opened, and its contents were filtered through celite, the solid residue was rinsed with CH_2Cl_2, and the combined filtrates were evaporated and purified on silica gel column chromatography with hexane/ethyl acetate to yield the 3-vinyloxazolidin-2-one derivatives **1**. The spectroscopic data for the compounds **(*S*)-1a**, **(*S*)-1b**, **(*S*)-1d**, and **(*R*)-1d** were identical to those reported in the literature.[2]

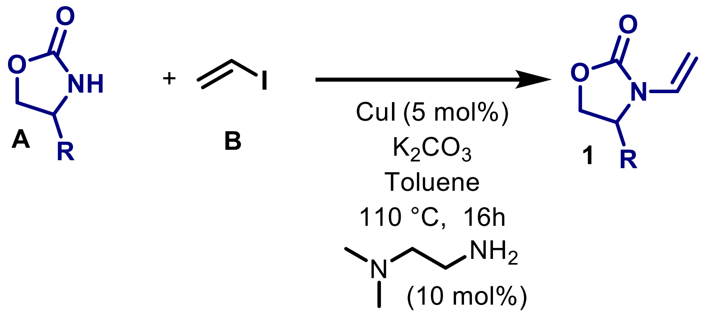

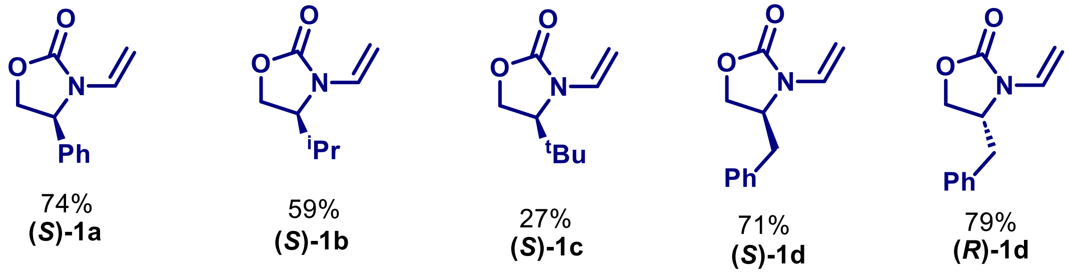

3. General Procedure 2 for Iron-Catalyzed Multicomponent Cross-Coupling Reaction

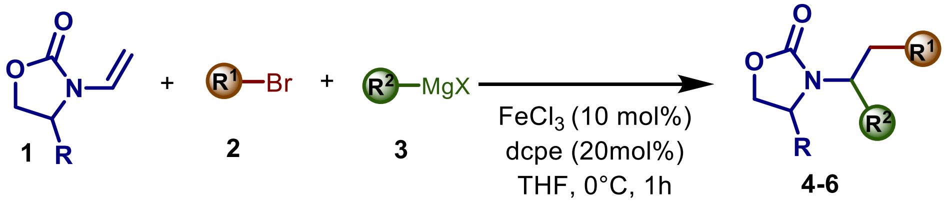

Standard-scale: A flame-dried 5 mL microwave vial with a stir bar was transferred into an argon-filled glovebox and the vial was charged with $FeCl_3$ (3.2 mg, 0.02 mmol), 1,2-bis(dicyclohexylphosphanyl)ethane (dcpe) (16.9 mg, 0.04 mmol), fluoroalkyl halide **2** (0.4 mmol, 2.0 equiv) and amide **1** (0.2 mmol, 1.0 equiv) (using oven-dried glass pipette to transfer alkyl halide to the vial). The vial was sealed with a Teflon cap and brought out of the glovebox without solvent. THF (0.2 mL) was then added into the reaction mixture. The resulting green solution was stirred at room temperature for 5 min. The reaction mixture was then cooled to 0 °C and a RMgBr **3** (0.5–1.0 M solution in THF, 4.0 equiv) was added slowly for 1 h using a syringe pump. Over which time the heterogeneous solution turned from red to yellow, brown, grass green or orange color (depending on ArMgBr and substrate). The resulting reaction mixture was then stirred at 0 °C for an additional 10 min. After completion, the reaction mixture was quenched with a 1.0 M aqueous solution of hydrochloric acid (0.5 mL) and water (0.5 mL) and then extracted with ethyl acetate (3 x 2 mL). The combined organic solution was filtered through a short pad of silica and evaporation of solvent gave a residue that was purified on silica gel column chromatography with hexane/ethyl acetate to obtain product **4-6**.

4. Analysis of diastereoisomers.

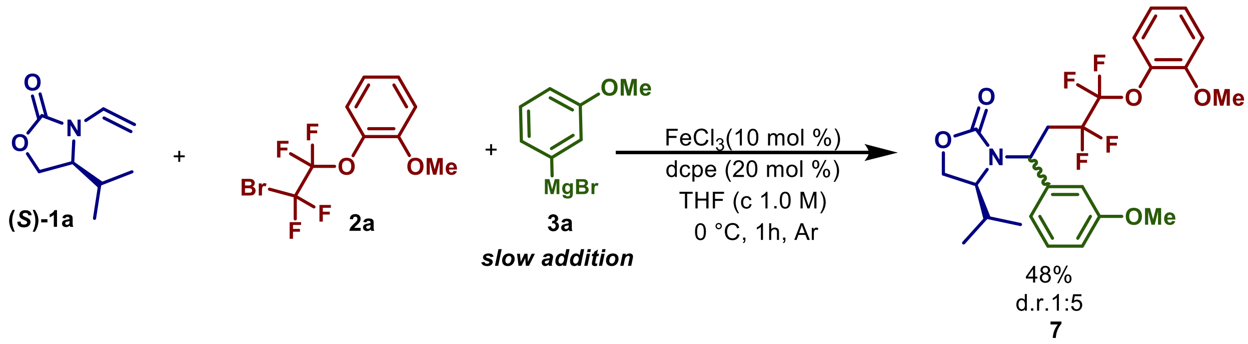

Compound **7** was synthesized following the general procedure 2 (standard-scale), using 1-(2-bromo-1,1,2,2-tetrafluoroethoxy)-2-methoxybenzene (120.4 mg, 0.4 mmol), (*S*)-4-isopropyl-3-vinyloxazolidin-2-one (31.0 mg, 0.2 mmol) and 3-methoxyphenyl magnesium bromide (0.8 mL, 1.0 M solution in THF, 0.8 mmol). Product 7 was obtained as a colorless liquid (46.6 mg, 48% yield) after purification by column chromatography on silica gel with hexane/EtOAc (8:2). The compounds are formed as a mixture of diastereomers in a ratio 1:5 as determined by crude ^{1}H NMR. Remarkably, after careful separation by prep-TLC, it was possible to characterize both diastereoisomers by ^{1}H-NMR (Figure S1-S2). Showing a clear difference in the chemical shift of the signals between the minor and major diastereoisomers. Signals A and B, which are characteristic of all the synthesized compounds, were used to calculate the diastereoisomer ratio in the reaction mixture (Figure S3).

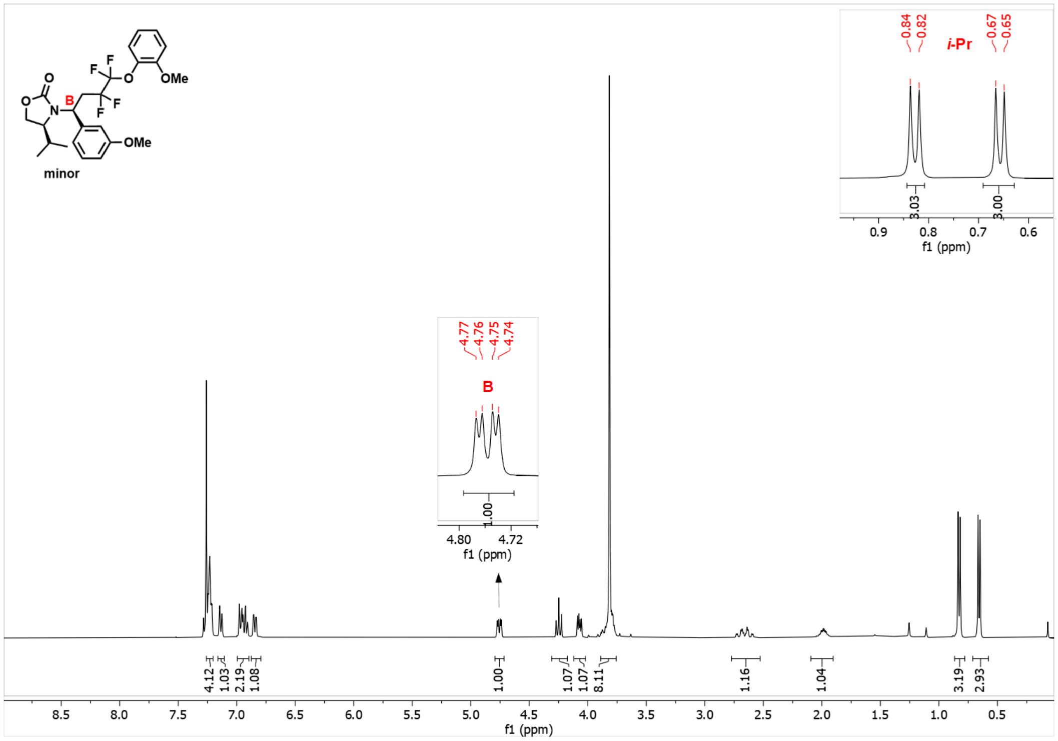

Figure S1. ^{1}H NMR ($CDCl_3$, 400 MHz) of minor diasteroisomer **7**.

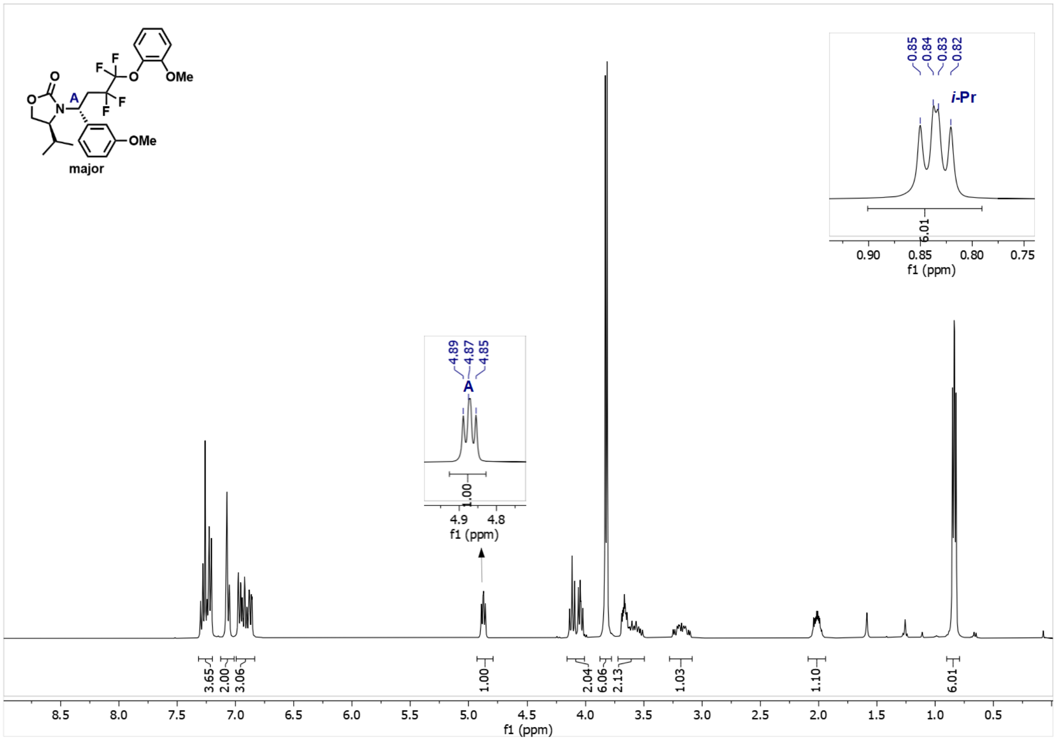

Figure S2. ^{1}H NMR ($CDCl_3$, 400 MHz) of major diasteroisomer **7**.

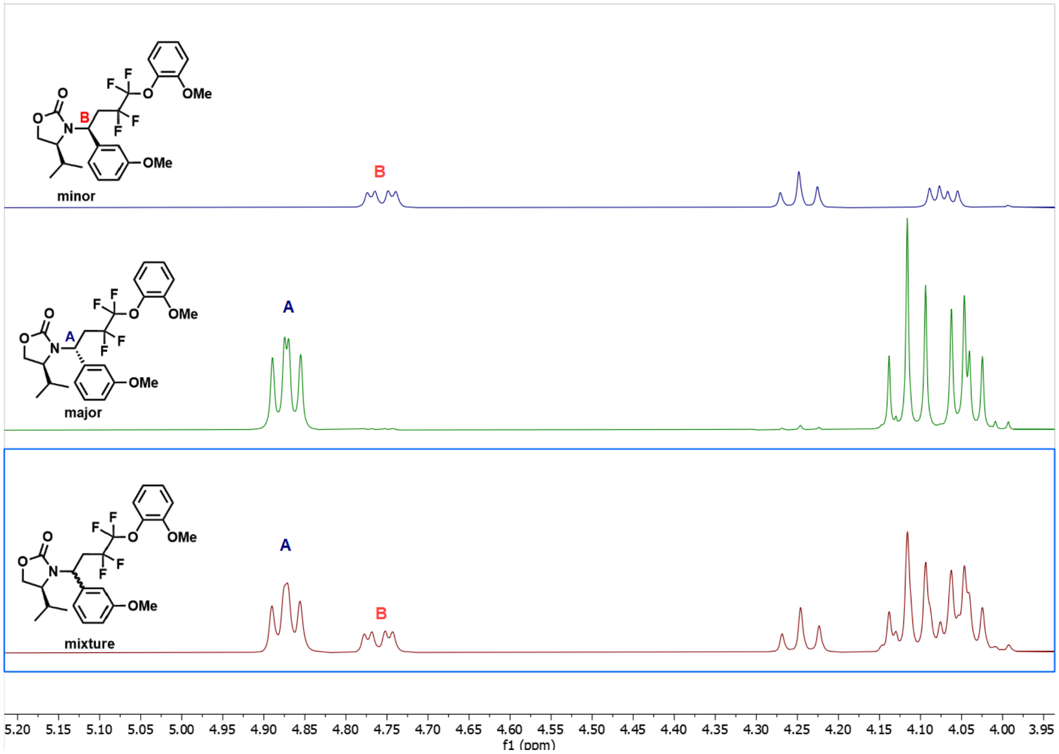

Figure S3. Comparison in ^{1}H NMR (5.20 to 3.95 ppm) spectras between a mixture and isolated diastereoisomers of **7**.

The stereochemistry of the major diastereoisomer product was determined through reported crystallographic analysis (*Angew. Chem. Int. Ed.* **2021**, *60*, 14399, see Figure S4). Upon close examination of this crystal structure, it was noted that the isopropyl group is opposite the aryl moiety. With this background, we have concluded that the major diastereoisomer resulting from our transformation has an analogous stereochemistry and was chosen for the assignment of our synthesized compounds. Remarkably, the formation of the major diastereoisomer is supported by computational studies (Figure S5).

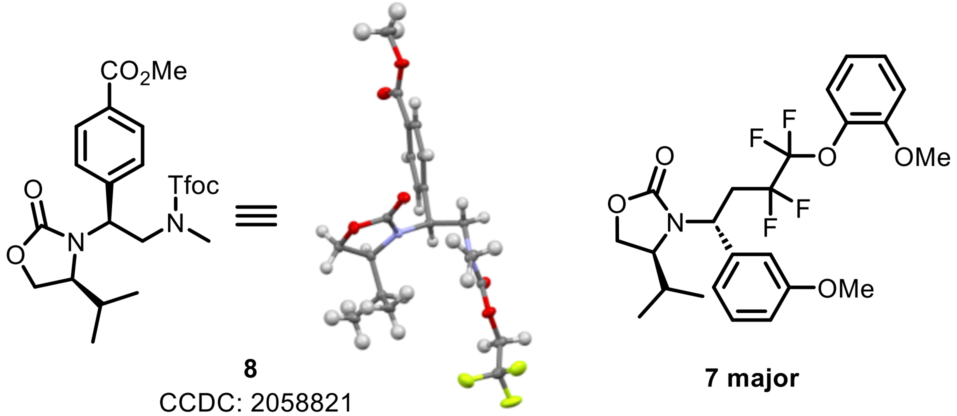

8
CCDC: 2058821

7 major

Figure S4. The assignment of the stereocenters in the major diastereoisomer **7** is based on crystallographic data from compound **8**.

5. Computational Details.

All geometry optimizations of intermediates and transition states were achieved using spin unrestricted UB3LYP[3]-D3[4]/def2svp[5]method with “opt=noeigen” and “guess=mix” keywords as implemented in Gaussian16[6]. Frequency calculations were also conducted at the same level of theory to obtain vibrational frequencies to determine the identity of stationary points as intermediates (no imaginary frequencies) or transition states (only one imaginary frequency), as well as obtain thermal correction to enthalpy and free energy at 298 K. Intrinsic Reaction Coordinate (IRC) calculations were done on the transition states to verify the correct transition state associated with the reaction. The endpoint geometries obtained from the IRC calculations were further optimized to verify the authenticity of the transition state. Also, an extensive conformational search was performed for all the organoiron intermediates and transition states. Additionally, the spin of each intermediate and transition state for the organoiron species was selected based on our previous mechanistic reports of related catalytic cycles.[7] Only the lowest-energy species were shown and discussed. Moreover, the solvation effects were taken into consideration using the SMD solvent model[8] with tetrahydrofuran, as the solvent used experimentally. Finally, we performed single-point calculations on the optimized geometries using the more robust uM06L[9]-D3/def2-TZVPP[5] method to improve our numerical precision. Therefore, the final reported energy was calculated at the uM06L-D3/def2tzvpp-SMD(THF)// uB3LYP-D3/def2svp-SMD(THF) level of theory.

All structural figures were generated with CYLview.[10] Distances in structural figures are shown in Å and energies are in kcal/mol. Noncovalent interaction (NCI) analysis, also known as reduced density gradient (RDG) method, was performed on Multiwfn to study the possible effect of noncovalent interaction in the relevant transition states.[11] Extension distance of 0 Bohr, medium quality grid (totally about 512000 points) were set by default. Further visualization of the color-filled RDG isosurface was realized by VMD, where RDG isosurface and color range were set as 0.5, and -0.035 to 0.2, respectively.[12]

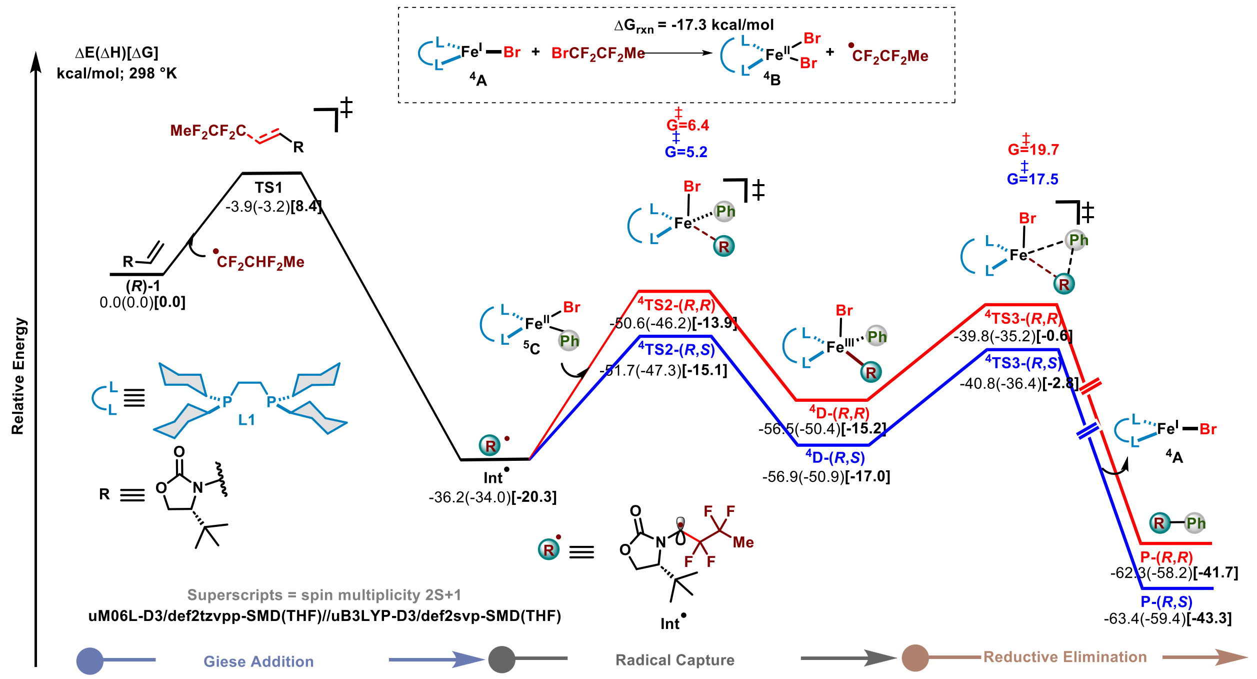

Figure S5. DFT calculation for dicarbofunctionalization of chiral vinyl oxazolidinone. Calculated Gibbs free energies [uM06L-D3/def2tzvpp-SMD(THF)// uB3LYP-D3/def2svp-SMD(THF)] are given in kcal mol^{-1}.

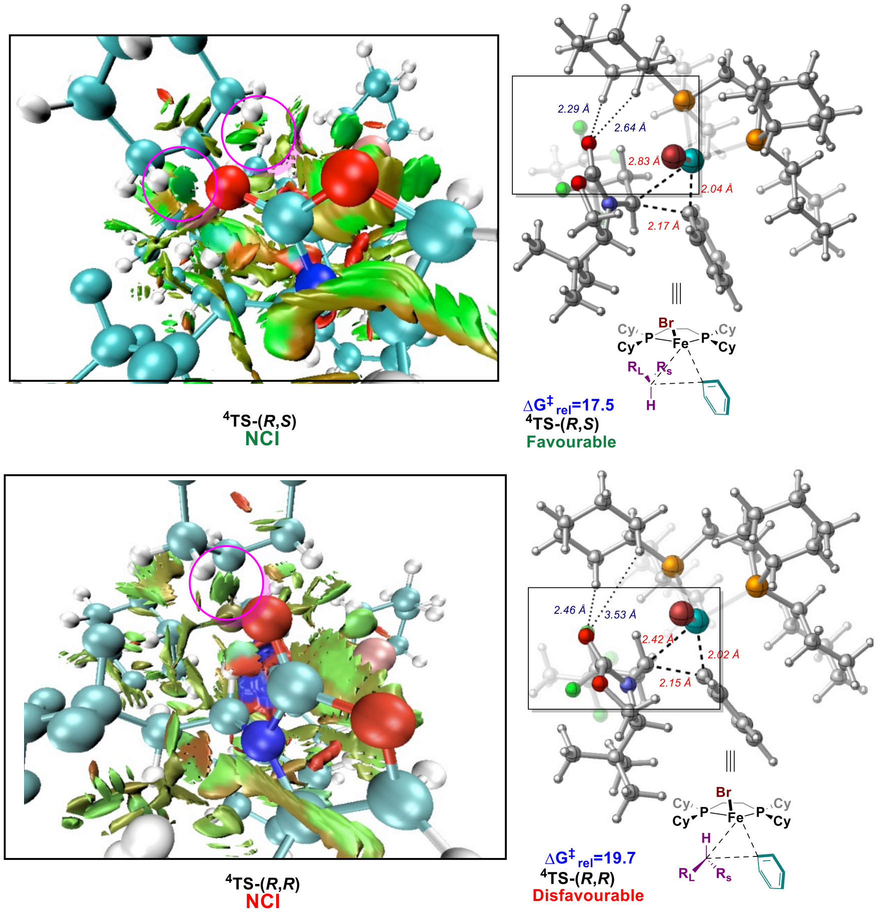

Figure S6. NCI (non-covalentinteraction)plots for ^{4}TS3-(*R*,*R*) and ^{4}TS3-(*R*,*S*).

6. Limitations:

Despite numerous attempts using representative vinyl- and alkyl Grignard reagents we have not observed the desired three component products. We recovered majority of the alkene from the reaction mixture (65-95%). We are extensively working on designing systems that utilize alkyl Grignard (or zinc) reagents in related transformations and will report in due course.

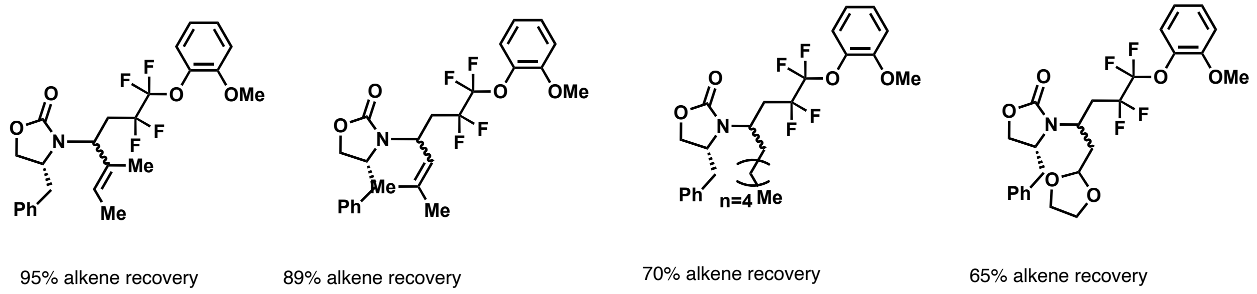

7. Grignard reagent optimization:

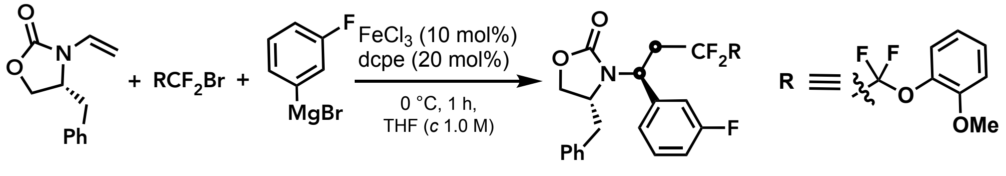

Potential side products

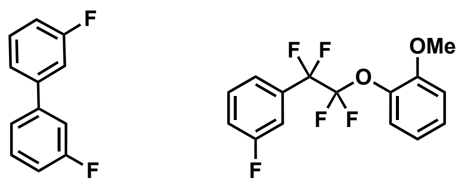

Table 1. Grignard reagnent optimization

Alkene (equiv.)	Radical precursor (equiv.)	Grignard reagents (equiv.)	NMR yield (%)	Alkene recovered
1.0	2.0	1.0	N.D.	91
1.0	2.0	2.0	39	50
1.0	2.0	3.0	63	31

We discovered that using 1, 2 or 3 equivalents of Grignard reagent led to comparable yields (i.e., N.D., 39%, 63% yield respectively). However, adding an excess of Grignard reagent improves the yield. A closer inspection of the products revealed that a major byproduct of the reaction is biaryl formation presumably from over reduction of the monoaryl Fe(II) to bisaryl Fe(II) and two componenet coupling product.

8. Product Characterization Data.

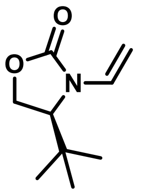

(*S*)-4-(*tert*-butyl)-3-vinyloxazolidin-2-one (*S*)-1c: Compound **(*S*)-1c** was synthesized following the general procedure 1, using (*S*)-4-(*tert*-butyl)oxazolidin-2-one (558.4 mg, 3.9 mmol), vinyl iodide (0.6 mL, 7.7 mmol), *N,N'*-dimethylethylenediamine (43 mL, 0.39 mmol), K_2CO_3 (299 mg, 2.1 mmol), CuI (38 mg, 0.20 mmol), and toluene (1.2 mL). The product **(*S*)-1c** was obtained as a colorless liquid (178.2 mg, 27% yield) after purification by column chromatography on silica gel with hexane/EtOAc (8:2).

^{1}H NMR (400 MHz, $CDCl_3$) δ = 6.71 (dd, J = 16.0, 9.4 Hz, 1H), 4.70 (d, *J* = 16.0 Hz, 1H), 4.49 (d, *J* = 9.4 Hz, 1H), 4.30 – 4.20 (m, 2H), 0.97 (s, 9H);

^{13}C NMR (100 MHz, $CDCl_3$) δ = 156.8, 131.5, 95.6, 65.6, 62.5, 36.7, 26.3;

IR (film) 2963.85, 2875.34, 1750.12, 1637.68, 1477.87, 1417.03, 1394.84, 1369.23, 1324.88, 1247.65, 1223.87, 1206.75, 1146.30, 1083.75, 1066.49, 1052.11, 1022.63, 1005.09, 973.84, 862.21, 765.66, 743.80, 681.62, 637.31, 602.17, 406.29 cm^{-1};

HRMS (ESI+) calcd for $C_9H_{15}NO_2$ $[M+H]^+$ m/z = 170.1176; found: 170.1169.

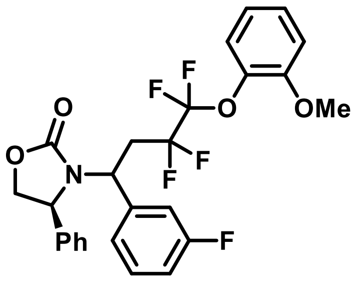

(4*S*)-4-phenyl-3-(3,3,4,4-tetrafluoro-1-(3-fluorophenyl)-4-(2-methoxyphenoxy)butyl)oxazolidin-2-one (*S*)-4a: Compound **(*S*)-4a** was synthesized following the general procedure 2 (standard-scale), using 1-(2-bromo-1,1,2,2-tetrafluoroethoxy)-2-methoxybenzene (120.4 mg, 0.4 mmol), (*S*)-4-phenyl-3-vinyloxazolidin-2-one (37.8 mg, 0.2 mmol) and 3-Fluorophenylmagnesium bromide (1.6 mL, 0.5 M solution in THF, 0.8 mmol). The product **(*S*)-4a** was obtained as a colorless liquid (57.8 mg, 57% yield) after purification by column chromatography on silica gel with hexane/EtOAc (8:2). The compounds are formed as a mixture of inseparable diastereomers in a ratio 5:1 as determined by crude ^{1}H NMR.

^{1}H NMR (400 MHz, $CDCl_3$, major) δ = 7.39 – 7.30 (m, 5H), 7.24 – 7.16 (m, 3H), 7.13 – 7.08 (m, 1H), 7.03 – 6.91 (m, 4H), 4.66 (t, *J* = 8.9 Hz, 1H), 4.59 – 4.50 (m, 2H), 4.12 (t, *J* = 8.8 Hz, 1H), 3.81 (s, 3H), 3.77 – 3.62 (m, 1H), 3.14 – 2.91 (m, 1H);

^{13}C NMR (100 MHz, $CDCl_3$, major) δ = 163.1 (d, *J* = 246.6 Hz), 158.0, 152.5, 141.2 (d, *J* = 6.7 Hz), 137.7, 136.2, 130.5 (d, *J* = 8.2 Hz), 129.6, 129.4, 127.9, 127.7, 123.9, 123.6 (d, *J* = 2.8 Hz), 120.7, 120.4 – 114.3 (m), 115.5 (d, *J* = 20.9 Hz), 114.8 (d, *J* = 22.0 Hz), 112.9, 70.2, 60.8, 56.1, 51.5, 32.9 (t, *J* = 20.1 Hz);

^{19}F NMR (376 MHz, $CDCl_3$,major) δ = -88.70 to -88.91 (m, 2F), -117.79 to -111.91 (m, 1F), -116.68 to -118.58 (m, 2F);

IR (film) 3069.31, 3035.34, 3010.65, 2966.09, 2913.90, 2842.15, 1746.99, 1591.77, 1502.00, 1457.95, 1410.68, 1339.28, 1304.77, 1282.48, 1259.29, 1222.68, 1188.96, 1170.56, 1106.92, 1042.62, 1024.14, 1000.02, 951.47, 877.78, 748.98, 700.91, 609.63, 558.41, 521.40, 489.10, 456.09 cm^{-1};

HRMS (ESI+) calcd for $C_{26}H_{22}F_5NO_4$ $[M+H]^+$ m/z = 508.1542; found: 508.1537.

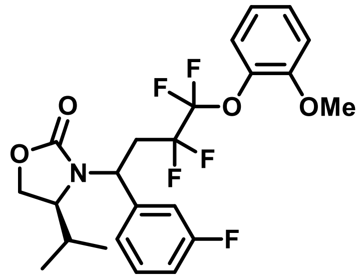

(4*S*)-4-isopropyl-3-(3,3,4,4-tetrafluoro-1-(3-fluorophenyl)-4-(2-methoxyphenoxy)butyl)oxazolidin-2-one (*S*)-4b: Compound **(*S*)-4b** was synthesized following the general procedure 2 (standard-scale), using 1-(2-bromo-1,1,2,2-tetrafluoroethoxy)-2-methoxybenzene (120.4 mg, 0.4 mmol), (*S*)-4-isopropyl-3-vinyloxazolidin-2-one (31.0 mg, 0.2 mmol) and 3-Fluorophenylmagnesium bromide (1.6 mL, 0.5 M solution in THF, 0.8 mmol). The product **(*S*)-4b** was obtained as a colorless liquid (73.8 mg, 78% yield) after purification by column chromatography on silica gel with hexane/EtOAc (8:2). The compounds are formed as a mixture of inseparable diastereomers in a ratio 18:1 as determined by crude ^{1}H NMR.

^{1}H NMR (400 MHz, CDCl$_3$,major) δ = 7.41 – 7.33 (m, 1H), 7.32 – 7.20 (m, 4H), 7.06 – 7.01 (m, 1H), 6.98 – 6.95 (m, 1H), 6.92 (td, *J* = 7.7, 1.5 Hz, 1H), 4.89 (t, *J* = 6.9 Hz, 1H), 4.13 (t, *J* = 8.9 Hz, 1H), 4.09 – 4.02 (m, 1H), 3.83 (s, 3H), 3.70 – 3.63 (m, 1H), 3.60 – 3.44 (m, 1H), 3.32 – 3.15 (m, 1H), 2.06 – 1.97 (m, 1H), 0.84 (dd, *J* = 10.7, 6.9 Hz, 6H);

^{13}C NMR (100 MHz, CDCl$_3$,major) δ = 163.1 (d, *J* = 246.6 Hz), 158.5, 152.5, 142.1 (d, *J* = 6.7 Hz), 137.7, 130.6 (d, *J* = 8.2 Hz), 127.7, 123.8, 123.4 (d, *J* = 2.8 Hz), 120.7, 120.4 – 114.3 (m), 115.6 (d, *J* = 20.9 Hz), 114.7 (d, *J* = 22.0 Hz), 112.9, 62.9, 59.7, 56.1, 51.2, 32.3 (t, *J* = 20.1 Hz), 27.6, 18.1, 13.9;

^{19}F NMR (376 MHz, CDCl$_3$,major) δ = -88.62 to -88.81 (m, 2F), -111.58 to -111.75 (m, 1F), -116.81 to -117.57 (m, 2F);

IR (film) 3071.60, 2963.93, 2929.84, 2843.86, 1740.62, 1591.95, 1502.50, 1455.99, 1420.23, 1393.14, 1347.31, 1303.05, 1282.26, 1259.44, 1188.83, 1171.61, 1107.53, 1042.69, 1025.23, 954.33, 909.17, 854.05, 766.89, 749.05, 730.24, 704.59, 647.39, 607.57, 521.43, 489.36, 455.73 cm^{-1};

HRMS (APCI+) calcd for $C_{23}H_{24}F_5NO_4$ $[M+H]^+$ m/z = 474.1698; found: 474.1687.

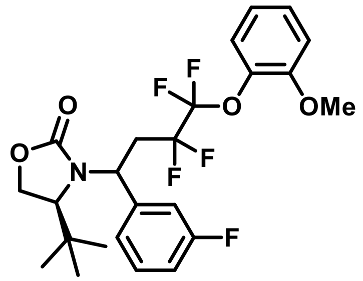

(4*S*)-4-(tert-butyl)-3-(3,3,4,4-tetrafluoro-1-(3-fluorophenyl)-4-(2-methoxyphenoxy)butyl)oxazolidin-2-one (*S*)-4c: Compound **(*S*)-4c** was synthesized following the general procedure 2 (standard-scale), using 1-(2-bromo-1,1,2,2-tetrafluoroethoxy)-2-methoxybenzene (120.4 mg, 0.4 mmol), (*S*)-4-(tert-butyl)-3-vinyloxazolidin-2-one (33.8 mg, 0.2 mmol) and 3-Fluorophenylmagnesium bromide (1.6 mL, 0.5 M solution in THF, 0.8 mmol). The product **(*S*)-4c** was obtained as a colorless liquid (39.0 mg, 40% yield) after purification by column chromatography on silica gel with hexane/EtOAc (8:2).

^{1}H NMR (400 MHz, CDCl$_3$) δ = 7.37 – 7.25 (m, 3H), 7.25 – 7.19 (m, 2H), 7.08 – 7.01 (m, 1H), 6.97 (dd, *J* = 8.2, 1.5 Hz, 1H), 6.91 (td, *J* = 7.6, 1.5 Hz, 1H), 4.78 (dd, *J* = 8.0, 5.5 Hz, 1H), 4.11 (dd, *J* = 9.2, 3.9 Hz, 1H), 4.00 (t, *J* = 8.9 Hz, 1H), 3.85 (s, 3H), 3.66 – 3.47 (m, 2H), 3.36 (dd, *J* = 8.9, 3.9 Hz, 1H), 1.04 (s, 9H);

^{13}C NMR (100 MHz, CDCl$_3$) δ = 163.1 (d, *J* = 246.6 Hz), 159.5, 152.6, 142.4 (d, *J* = 6.7 Hz), 137.8, 130.6 (d, *J* = 8.2 Hz), 127.6, 123.8, 123.4 (d, *J* = 2.8 Hz), 120.6, 120.4 – 114.3 (m), 115.7 (d, *J* = 20.9 Hz), 114.8 (d, *J* = 22.0 Hz), 112.9, 65.3, 65.0, 56.1, 55.5, 35.3, 34.7 (t, *J* = 20.1 Hz), 25.8;

^{19}F NMR (376 MHz, CDCl$_3$) δ = -88.62 to -88.84 (m, 2F), -111.52 to -111.63 (m, 1F), -115.43 to -118.35 (m, 2F);

IR (film) 2960.70, 2922.66, 2852.87, 1742.99, 1591.89, 1503.11, 1456.75, 1425.40, 1401.32, 1367.55, 1334.36, 1304.60, 1282.50, 1260.79, 1227.25, 1189.82, 1170.97, 1108.05, 1042.56, 1026.85, 1000.23, 958.69, 879.91, 769.53, 748.56, 703.66, 608.53, 555.89, 521.72, 488.11, 462.72 cm^{-1};

HRMS (ESI+) calcd for $C_{24}H_{26}F_5NO_4$ $[M+H]^+$ m/z = 488.1855; found: 488.1843.

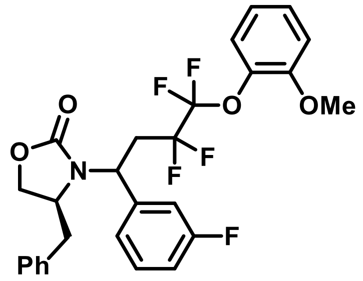

(4S)-4-benzyl-3-(3,3,4,4-tetrafluoro-1-(3-fluorophenyl)-4-(2-methoxyphenoxy)butyl)oxazolidin-2-one (*S*)-4d: Compound **(*S*)-4d** was synthesized following the general procedure 2 (standard-scale), using 1-(2-bromo-1,1,2,2-tetrafluoroethoxy)-2-methoxybenzene (120.4 mg, 0.4 mmol), (*S*)-4-benzyl-3-vinyloxazolidin-2-one (40.6 mg, 0.2 mmol) and 3-Fluorophenylmagnesium bromide (1.6 mL, 0.5 M solution in THF, 0.8 mmol). The product **(*S*)-4d** was obtained as a colorless liquid (79.3 mg, 76% yield) after purification by column chromatography on silica gel with hexane/EtOAc (8:2).

^{1}H NMR (400 MHz, CDCl$_3$) δ = 7.38 – 7.29 (m, 3H), 7.29 – 7.21 (m, 4H), 7.18 (dt, *J* = 9.8, 2.2 Hz, 1H), 7.15 – 7.09 (m, 2H), 7.04 (tdd, *J* = 8.4, 2.5, 1.0 Hz, 1H), 6.98 (dd, *J* = 8.7, 1.6 Hz, 1H), 6.93 (td, *J* = 7.6, 1.5 Hz, 1H), 4.99 (dd, *J* = 8.7, 5.2 Hz, 1H), 4.14 – 4.06 (m, 1H), 4.04 – 3.96 (m, 2H), 3.83 (s, 3H), 3.80 – 3.66 (m, 1H), 3.15 – 3.00 (m, 2H), 2.64 (dd, *J* = 13.8, 8.6 Hz, 1H);

^{13}C NMR (100 MHz, CDCl$_3$) δ = 163.1 (d, *J* = 246.6 Hz), 157.9, 152.5, 141.7 (d, *J* = 6.7 Hz), 137.7, 135.6, 130.6 (d, *J* = 8.2 Hz), 129.1, 129.0, 127.7, 127.4, 123.9, 123.3 (d, *J* = 2.8 Hz), 120.7, 120.4 – 114.3 (m), 115.6 (d, *J* = 20.9 Hz), 114.7 (d, *J* = 22.0 Hz), 112.9, 67.4, 56.7, 56.1, 51.3, 39.0, 32.9 (t, *J* = 20.1 Hz);

^{19}F NMR (376 MHz, CDCl$_3$) δ = -88.62 to -88.76 (m, 2F), -111.42 to -111.56 (m, 1F), -117.12 to -117.38 (m, 2F);

IR (film) 3066.53, 3028.23, 3006.28, 2925.19, 2842.46, 1743.98, 1591.76, 1501.42, 1455.00, 1417.90, 1389.35, 1349.77, 1302.82, 1282.36, 1259.46, 1188.42, 1171.18, 1107.25, 1042.47, 1025.96, 956.28, 878.55, 788.00, 765.91, 748.73, 702.16, 607.83, 561.58, 521.78, 504.70 cm^{-1};

HRMS (APCI+) calcd for $C_{27}H_{24}F_5NO_4$ $[M+H]^+$ m/z = 522.1698; found: 522.1701.

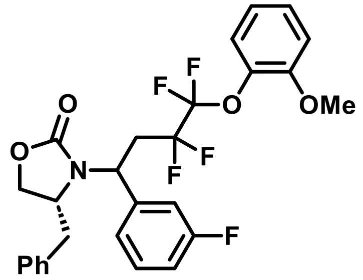

(4*R*)-4-benzyl-3-(3,3,4,4-tetrafluoro-1-(3-fluorophenyl)-4-(2-methoxyphenoxy)butyl)oxazolidin-2-one (*R*)-4d: Compound **(*R*)-4d** was synthesized following the general procedure 2 (standard-scale), using 1-(2-bromo-1,1,2,2-tetrafluoroethoxy)-2-methoxybenzene (120.4 mg, 0.4 mmol), (*S*)-4-benzyl-3-vinyloxazolidin-2-one (40.6 mg, 0.2 mmol) and 3-Fluorophenylmagnesium bromide (1.6 mL, 0.5 M solution in THF, 0.8 mmol). The product **(*R*)-4d** was obtained as a colorless liquid (83.0 mg, 80% yield) after purification by column chromatography on silica gel with hexane/EtOAc (8:2).

^{1}H NMR (400 MHz, CDCl$_3$) δ = 7.38 – 7.29 (m, 3H), 7.28 – 7.22 (m, 4H), 7.18 (dt, *J* = 9.8, 2.2 Hz, 1H), 7.15 – 7.10 (m, 2H), 7.04 (td, *J* = 8.4, 1.7 Hz, 1H), 6.98 (dd, *J* = 8.6, 1.4 Hz, 1H), 6.94 (td, *J* = 7.9, 1.4 Hz, 1H), 4.98 (dd, *J* = 8.7, 5.1 Hz, 1H), 4.18 – 4.06 (m, 1H), 4.04 – 3.95 (m, 2H), 3.83 (s, 3H), 3.80 – 3.65 (m, 1H), 3.15 – 3.01 (m, 2H), 2.65 (dd, *J* = 13.7, 8.5 Hz, 1H);

^{13}C NMR (100 MHz, CDCl$_3$) δ = 163.1 (d, *J* = 246.6 Hz), 157.9, 152.5, 141.7 (d, *J* = 6.7 Hz), 137.7, 135.6, 130.6 (d, *J* = 8.2 Hz), 129.1, 129.0, 127.7, 127.4, 123.9, 123.3 (d, *J* = 2.8 Hz), 120.7, 120.4 – 114.3 (m), 115.6 (d, *J* = 20.9 Hz), 114.7 (d, *J* = 22.0 Hz), 112.9, 67.4, 56.7, 56.1, 51.3, 38.9, 32.9 (t, *J* = 20.1 Hz);

^{19}F NMR (376 MHz, CDCl$_3$) δ = -88.62 to -88.80 (m, 2F), -111.41 to -111.60 (m, 1F), -117.14 to -117.39 (m, 2F);

IR (film) 3064.72, 3029.14, 3006.75, 2946.91, 2842.15, 1743.52, 1591.90, 1501.76, 1454.96, 1418.26, 1389.43, 1350.42, 1302.96, 1282.38, 1259.87, 1188.71, 1171.08, 1107.05, 1042.20, 1025.27, 955.23, 878.64, 788.39, 765.77, 738.17, 700.62, 607.23, 559.99, 521.19, 503.50 cm^{-1};

HRMS (ESI+) calcd for $C_{27}H_{24}F_5NO_4$ $[M+H]^+$ m/z = 522.1698; found: 522.1702.

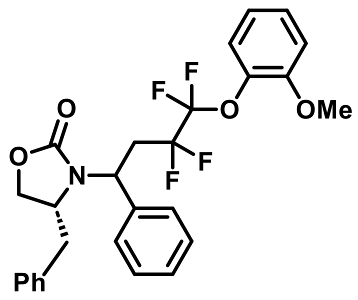

(4*R*)-4-benzyl-3-(3,3,4,4-tetrafluoro-4-(2-methoxyphenoxy)-1-phenylbutyl)oxazolidin-2-one (5a): Compound **5a** was synthesized following the general procedure 2 (standard-scale), using 1-(2-bromo-1,1,2,2-tetrafluoroethoxy)-2-methoxybenzene (120.4 mg, 0.4 mmol), (*R*)-4-benzyl-3-vinyloxazolidin-2-one (40.6 mg, 0.2 mmol) and Phenylmagnesium bromide (0.8 mL, 1.0 M solution in THF, 0.8 mmol). The product **5a** was obtained as a colorless liquid (77.1 mg, 77% yield) after purification by column chromatography on silica gel with hexane/EtOAc (8:2).

^{1}H NMR (400 MHz, $CDCl_3$) δ = 7.50 – 7.45 (m, 2H), 7.42 – 7.28 (m, 5H), 7.26 – 7.20 (m, 3H), 7.13 – 7.08 (m, 2H), 6.99 – 6.91 (m, 2H), 5.03 (dd, *J* = 9.0, 4.9 Hz, 1H), 4.13 – 4.04 (m, 1H), 4.03 – 3.95 (m, 2H), 3.83 (s, 3H), 3.80 – 3.68 (m, 1H), 3.14 – 2.99 (m, 2H), 2.61 (dd, *J* = 13.8, 8.8 Hz, 1H);

^{13}C NMR (100 MHz, $CDCl_3$) δ = 158.0, 152.6, 139.3, 137.8, 135.8, 129.1(2), 129.0(9), 129.0(6), 128.6, 127.7, 127.6, 127.3, 123.9, 120.7, 119.3 – 114.3 (m), 112.9, 67.3, 56.6, 56.1, 51.7, 38.9, 32.8 (t, *J* = 20.2 Hz);

^{19}F NMR (376 MHz, $CDCl_3$) δ = -88.23 to -89.13 (m, 2F), -117.25 to -117.46 (m, 2F);;

IR (film) 3064.04, 3029.3, 3006.72, 2959.54, 2931.02, 2841.71, 1743.76, 1603.76, 1501.10, 1455.66, 1417.39, 1417.39, 1389.42, 1355.10, 1282.10, 1260.82, 1235.88, 1188.17, 1170.56, 1106.48, 1042.05, 1025.37, 994.52, 935.31, 854.12, 807.82, 734.26, 699.29, 604.92, 567.36, 504.34 cm^{-1};

HRMS (ESI+) calcd for $C_{27}H_{25}F_4NO_4$ $[M+H]^+$ m/z = 504.1792; found: 504.1792.

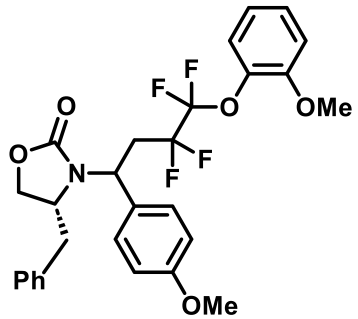

(4*R*)-4-benzyl-3-(3,3,4,4-tetrafluoro-4-(2-methoxyphenoxy)-1-(4-methoxyphenyl)butyl)oxazolidin-2-one (5b): Compound **5b** was synthesized following the general procedure 2 (standard-scale), using 1-(2-bromo-1,1,2,2-tetrafluoroethoxy)-2-methoxybenzene (120.4 mg, 0.4 mmol), (*R*)-4-benzyl-3-vinyloxazolidin-2-one (40.6 mg, 0.2 mmol) and 4-methoxyphenylmagnesium bromide (1.6 mL, 0.5 M solution in THF, 0.8 mmol). The product **5b** was obtained as a colorless liquid (20.9 mg, 20% yield) after purification by column chromatography on silica gel with hexane/EtOAc (8:2).

^{1}H NMR (400 MHz, $CDCl_3$) δ = 7.40 (d, *J* = 8.7 Hz, 2H), 7.33 – 7.27 (m, 2H), 7.27 – 7.20 (m, 3H), 7.11 (d, *J* = 6.8 Hz, 2H), 7.00 – 6.92 (m, 2H), 6.90 (d, *J* = 8.8 Hz, 2H), 4.98 (dd, *J* = 8.7, 5.2 Hz, 1H), 4.10 – 3.94 (m, 3H), 3.83 (s, 3H), 3.81 (s, 3H), 3.78 – 3.64 (m, 1H), 3.14 – 2.96 (m, 2H), 2.59 (dd, *J* = 13.7, 8.6 Hz, 1H);

^{13}C NMR (100 MHz, $CDCl_3$) δ = 159.6, 157.9, 152.5, 137.7, 135.8, 131.3, 128.9(7), 128.9(6), 128.8, 127.6, 127.2, 123.8, 120.6, 119.3 – 114.3 (m), 114.2, 112.8, 67.2, 56.4, 56.0, 55.3, 51.1, 32.8 (t, *J* = 20.2 Hz);

^{19}F NMR (376 MHz, $CDCl_3$) δ = -88.23 to -89.23 (m, 2F), -117.01 to -117.72 (m, 2F);;

IR (film) 3063.51, 3027.67, 3004.06, 2957.81, 2934.68, 2840.01, 1742.92, 1610.58, 1585.59, 1513.68, 1501.42, 1455.87, 1440.14, 1415.67, 1389.20, 1352.34, 1304.41, 1282.27, 1251.05, 1170.90, 1106.46, 1025.53, 833.94, 793.14, 741.44, 701.00, 635.81, 615.53, 565.26, 504.43, 458.10 cm^{-1};

HRMS (ESI+) calcd for $C_{28}H_{27}F_4NO_5$ $[M+H]^+$ m/z = 534.1898; found: 534.1902.

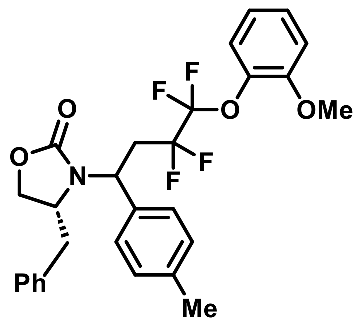

(4*R*)-4-benzyl-3-(3,3,4,4-tetrafluoro-4-(2-methoxyphenoxy)-1-(*p*-tolyl)butyl)oxazolidin-2-one (5c): Compound **5c** was synthesized following the general procedure 2 (standard-scale), using 1-(2-bromo-1,1,2,2-tetrafluoroethoxy)-2-methoxybenzene (120.4 mg, 0.4 mmol), (*R*)-4-benzyl-3-vinyloxazolidin-2-one (40.6 mg, 0.2 mmol) and *p*-Tolylmagnesium bromide (0.8 mL, 1.0 M solution in THF, 0.8 mmol). The product **5c** was obtained as a colorless liquid (78.8 mg, 76% yield) after purification by column chromatography on silica gel with hexane/EtOAc (8:2).

^{1}H NMR (400 MHz, $CDCl_3$) δ = 7.37 (d, *J* = 8.1 Hz, 2H), 7.33 – 7.27 (m, 2H), 7.27 – 7.19 (m, 5H), 7.11 (d, *J* = 6.9 Hz, 2H), 6.98 (dd, *J* = 8.7, 1.3 Hz, 1H), 6.94 (td, *J* = 7.9, 1.5 Hz, 1H), 5.01 (dd, *J* = 8.9, 5.1 Hz, 1H), 4.09 – 3.95 (m, 3H), 3.83 (s, 3H), 3.78 – 3.67 (m, 1H), 3.15 – 2.97 (m, 2H), 2.59 (dd, *J* = 13.7, 8.7 Hz, 1H), 2.36 (s, 3H);

^{13}C NMR (100 MHz, $CDCl_3$) δ = 158.0, 152.6, 138.4, 137.8, 136.3, 135.9, 129.7, 129.1, 127.7, 127.5, 127.3, 123.9, 120.7, 120.4 – 114.3 (m), 112.9, 67.2, 56.5, 56.1, 51.4, 38.9, 32.8 (t, *J* = 20.2 Hz), 21.2;

^{19}F NMR (376 MHz, $CDCl_3$) δ = -88.27 to -89.26 (m, 2F), -116.19 to -118.13 (m, 2F);

IR (film) 3063.54, 3027.87, 3006.09, 2925.23, 2841.52, 1743.70, 1603.96, 1501.31, 1455.60, 1415.49, 1387.84, 1351.53, 1319.93, 1301.92, 1282.18, 1260.75, 1236.08, 1187.04, 1170.30, 1106.39, 1041.83, 1022.68, 841.91, 819.10, 765.77, 740.75, 700.92, 639.95, 616.20, 570.05, 504.64 cm^{-1};

HRMS (ESI+) calcd for $C_{28}H_{27}F_4NO_4$ $[M+H]^+$ m/z = 534.1949; found: 534.1949.

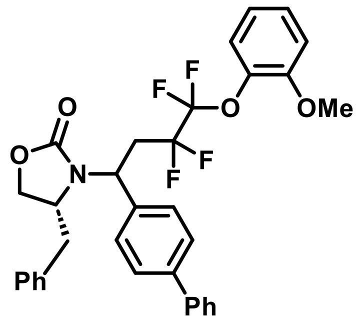

(4*R*)-3-(1-([1,1'-biphenyl]-4-yl)-3,3,4,4-tetrafluoro-4-(2-methoxyphenoxy)butyl)-4-benzyloxazolidin-2-one (5d): Compound **5d** was synthesized following the general procedure 2 (standard-scale), using 1-(2-bromo-1,1,2,2-tetrafluoroethoxy)-2-methoxybenzene (120.4 mg, 0.4 mmol), (*R*)-4-benzyl-3-vinyloxazolidin-2-one (40.6 mg, 0.2 mmol) and 4-Biphenylmagnesium bromide (1.6 mL, 0.5 M solution in THF, 0.8 mmol). The product **5d** was obtained as a colorless liquid (95.3 mg, 82% yield) after purification by column chromatography on silica gel with hexane/EtOAc (8:2).

^{1}H NMR (400 MHz, $CDCl_3$) δ = 7.61 (t, *J* = 8.4 Hz, 4H), 7.56 – 7.51 (m, 2H), 7.46 (t, *J* = 7.6 Hz, 2H), 7.38 – 7.21 (m, 6H), 7.14 (d, *J* = 7.1 Hz, 2H), 7.00 – 6.92 (m, 2H), 5.06 (dd, *J* = 8.9, 5.1 Hz, 1H), 4.13 – 3.98 (m, 3H), 3.84 (s, 3H), 3.83 – 3.72 (m, 1H), 3.21 – 3.05 (m, 2H), 2.66 (dd, *J* = 13.6, 8.9 Hz, 1H);

^{13}C NMR (100 MHz, $CDCl_3$) δ = 158.0, 152.6, 141.5, 140.5, 138.3, 137.8, 135.8, 129.1(2), 129.0(9), 129.9, 128.1, 127.8, 127.7, 127.4, 127.2, 124.0, 120.7, 120.4 – 114.3 (m), 112.9, 67.4, 56.7, 56.1, 51.5, 39.0, 32.9 (t, *J* = 20.2 Hz);

^{19}F NMR (376 MHz, $CDCl_3$) δ = -88.04 to -89.50 (m, 2F), -116.01 to -117.43 (m, 2F);

IR (film) 3060.76, 3029.89, 3005.89, 2958.65, 2841.05, 1743.73, 1603.47, 1501.35, 1488.24, 1455.33, 1413.00, 1389.06, 1389.06, 1351.33, 1320.43, 1282.01, 1260.58, 1235.78, 1188.59, 1170.37, 1106.85, 1041.78, 1024.24, 1007.20, 915.93, 843.71, 763.03, 734.67, 697.19, 639.72, 607.06, 571.51, 505.33, 457.47 cm^{-1};

HRMS (ESI+) calcd for $C_{33}H_{29}F_4NO_4$ $[M+H]^+$ m/z = 580.2105; found: 580.2107.

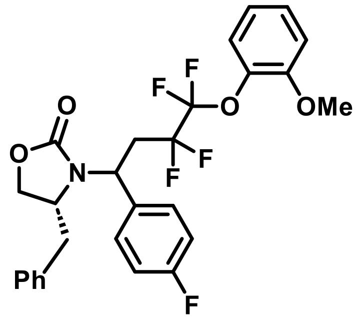

(4*R*)-4-benzyl-3-(3,3,4,4-tetrafluoro-1-(4-fluorophenyl)-4-(2-methoxyphenoxy)butyl)oxazolidin-2-one (5e): Compound **5e** was synthesized following the general procedure 2 (standard-scale), using 1-(2-bromo-1,1,2,2-tetrafluoroethoxy)-2-methoxybenzene (120.4 mg, 0.4 mmol), (*R*)-4-benzyl-3-vinyloxazolidin-2-one (40.6 mg, 0.2 mmol) and 4-Fluorophenylmagnesium bromide (0.8 mL, 1.0 M solution in THF, 0.8 mmol). The product **5e** was obtained as a colorless liquid (36.0 mg, 35% yield) after purification by column chromatography on silica gel with hexane/EtOAc (8:2).

^{1}H NMR (400 MHz, $CDCl_3$) δ = 7.47 – 7.40 (m, 2H), 7.35 – 7.29 (m, 2H), 7.29 – 7.22 (m, 3H), 7.15 – 7.10 (m, 2H), 7.09 – 7.04 (m, 2H), 6.98 (dd, *J* = 8.6, 1.4 Hz, 1H), 6.94 (td, *J* = 7.8, 1.4 Hz, 1H), 4.95 (dd, *J* = 8.4, 5.5 Hz, 1H), 4.14 – 4.06 (m, 1H), 4.02 – 3.93 (m, 2H), 3.83 (s, 3H), 3.79 – 3.62 (m, 1H), 3.16 – 3.01 (m, 2H), 2.65 (dd, *J* = 13.8, 8.8 Hz, 1H);

^{13}C NMR (100 MHz, $CDCl_3$) δ = 162.8 (d, *J* = 246.2 Hz), 158.0, 152.5, 137.8, 135.7, 135.2 (d, *J* = 3.4 Hz), 129.4(7) (d, *J* = 8.1 Hz), 129.2, 129.0, 127.7, 127.4, 123.9, 120.8, 120.4 – 114.3 (m), 116.0 (d, *J* = 21.3 Hz), 112.9, 67.4, 56.7, 56.1, 51.2, 39.0, 33.1 (t, *J* = 20.6 Hz);

^{19}F NMR (376 MHz, $CDCl_3$) δ = -88.29 to -88.92 (m, 2F), -113.05 to -113.38 (m, 1F), 117.09 to -117.40 (m, 2F);

IR (film) 3067.15, 3028.63, 3007.27, 2930.70, 2842.23, 1743.04, 1604.48, 1501.51, 1455.90, 1416.65, 1389.40, 1349.86, 1301.83, 1282.36, 1260.93, 1222.66, 1188.29, 1170.62, 1160.92, 1107.57, 1042.17, 1025.30, 840.80, 810.78, 766.48, 738.24, 700.64, 634.79, 607.77, 569.38, 556.38, 519.83, 503.62, 424.11, 410.46 cm^{-1};

HRMS (ESI+) calcd for $C_{27}H_{24}F_5NO_4$ $[M+H]^+$ m/z = 522.1698; found: 522.1696.

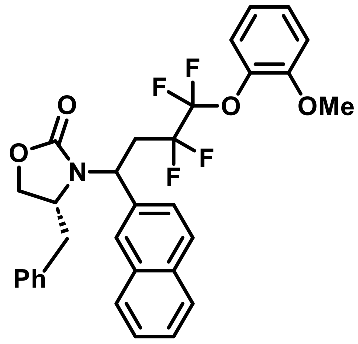

(4*R*)-4-benzyl-3-(3,3,4,4-tetrafluoro-4-(2-methoxyphenoxy)-1-(naphthalen-2-yl)butyl)oxazolidin-2-one (5f): Compound **5f** was synthesized following the general procedure 2 (standard-scale), using 1-(2-bromo-1,1,2,2-tetrafluoroethoxy)-2-methoxybenzene (120.4 mg, 0.4 mmol), (*R*)-4-benzyl-3-vinyloxazolidin-2-one (40.6 mg, 0.2 mmol) and 2-naphthylmagnesium bromide (1.6 mL, 0.5 M solution in THF, 0.8 mmol). The product **5f** was obtained as a colorless liquid (94.6 mg, 85% yield) after purification by column chromatography on silica gel with hexane/EtOAc (8:2).

^{1}H NMR (400 MHz, $CDCl_3$) δ = 7.90 – 7.82 (m, 3H), 7.75 (s, 1H), 7.68 (dd, *J* = 8.6, 1.7 Hz, 1H), 7.54 – 7.50 (m, 2H), 7.31 – 7.22 (m, 5H), 7.11 (d, *J* = 6.6 Hz, 2H), 6.98 (d, *J* = 8.0 Hz, 1H), 6.94 (td, *J* = 7.8, 1.4 Hz, 1H), 5.20 (dd, *J* = 8.9, 5.0 Hz, 1H), 4.14 – 3.97 (m, 3H), 3.95 – 3.83 (m, 1H), 3.83 (s, 3H), 3.27 – 3.08 (m, 2H), 2.63 (dd, *J* = 13.7, 8.7 Hz, 1H);

^{13}C NMR (100 MHz, $CDCl_3$) δ = 158.1, 152.6, 137.8, 136.8, 135.9, 133.2(8), 133.2(5), 129.2, 129.1, 128.2, 127.8, 127.7, 127.3, 126.7, 126.6(5), 126.4, 125.4, 124.0, 120.7, 120.4 – 114.3 (m), 112.9, 67.3, 56.6, 56.1, 51.9, 39.0, 33.1 (t, *J* = 20.5 Hz);

^{19}F NMR (376 MHz, $CDCl_3$) δ = -88.16 to -88.13 (m, 2F), 116.84 to -117.74 (m, 2F);

IR (film) 3060.28, 3027.81, 2958.79, 2931.04, 2870.81, 2841.46, 1744.39, 1602.95, 1501.66, 1455.73, 1417.49, 1390.05, 1337.12, 1282.22, 1262.02, 1188.93, 1170.98, 1107.54, 1042.67, 1026.13, 895.39, 859.39, 820.91, 733.75, 700.04, 606.89, 568.86, 478.35 cm^{-1};

HRMS (ESI+) calcd for $C_{31}H_{27}F_4NO_4$ $[M+H]^+$ m/z = 554.1949; found: 554.1952.

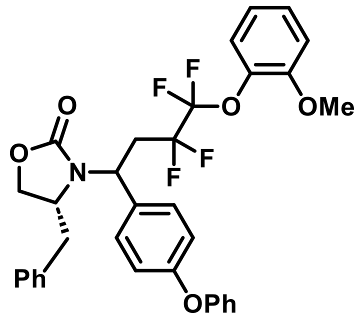

(4*R*)-4-benzyl-3-(3,3,4,4-tetrafluoro-4-(2-methoxyphenoxy)-1-(4-phenoxyphenyl)butyl)oxazolidin-2-one (5g): Compound **5g** was synthesized following the general procedure 2 (standard-scale), using 1-(2-bromo-1,1,2,2-tetrafluoroethoxy)-2-methoxybenzene (120.4 mg, 0.4 mmol), (*R*)-4-benzyl-3-vinyloxazolidin-2-one (40.6 mg, 0.2 mmol) and 4-Phenoxyphenylmagnesium bromide (1.6 mL, 0.5 M solution in THF, 0.8 mmol). The product **5g** was obtained as a colorless liquid (90.6 mg, 76% yield) after purification by column chromatography on silica gel with hexane/EtOAc (8:2).

^{1}H NMR (400 MHz, CDCl$_3$) δ = 7.42 (d, *J* = 8.7 Hz, 2H), 7.38 – 7.29 (m, 4H), 7.29 – 7.22 (m, 3H), 7.16 – 7.10 (m, 3H), 7.05 – 6.96 (m, 5H), 6.94 (td, *J* = 7.8, 1.4 Hz, 1H), 4.98 (dd, *J* = 8.6, 5.2 Hz, 1H), 4.14 – 4.05 (m, 1H), 4.04 – 3.95 (m, 2H), 3.84 (s, 3H), 3.81 – 3.64 (m, 1H), 3.18 – 2.99 (m, 2H), 2.65 (dd, *J* = 13.7, 8.7 Hz, 1H);

^{13}C NMR (100 MHz, CDCl$_3$) δ = 157.9, 157.7, 156.7, 152.6, 137.8, 135.8, 133.8, 130.0, 129.2, 129.1(2), 129.0(6), 127.7, 127.4, 123.9, 123.8, 120.7, 120.4 – 114.3 (m), 119.4, 118.9, 112.9, 67.3, 56.6, 56.1, 51.2, 38.9, 33.0 (t, *J* = 20.5 Hz);

^{19}F NMR (376 MHz, CDCl$_3$) δ = -88.24 to -89.14 (m, 2F), 116.97 to -117.75 (m, 2F);

IR (film) 3062.92, 3029.40, 3006.41, 2957.43, 2930.18, 2870.65, 2841.41, 1744.95, 1589.19, 1503.00, 1488.62, 1455.78, 1416.60, 1389.27, 1350.88, 1317.06, 1282.31, 1261.44, 1232.69, 1188.78, 1169.93, 1107.70, 1042.60, 1024.58, 909.97, 871.41, 734.40, 692.71, 616.11, 564.56, 501.34, 501.34, 413.64 cm^{-1};

HRMS (APCI+) calcd for $C_{33}H_{29}F_4NO_5$ $[M+H]^+$ m/z = 596.2055; found: 596.2031.

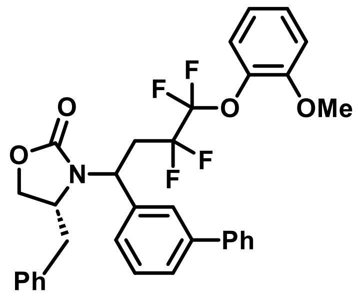

(4*R*)-3-(1-([1,1'-biphenyl]-3-yl)-3,3,4,4-tetrafluoro-4-(2-methoxyphenoxy)butyl)-4-benzyloxazolidin-2-one (5h): Compound **5h** was synthesized following the general procedure 2 (standard-scale), using 1-(2-bromo-1,1,2,2-tetrafluoroethoxy)-2-methoxybenzene (120.4 mg, 0.4 mmol), (*R*)-4-benzyl-3-vinyloxazolidin-2-one (40.6 mg, 0.2 mmol) and 3-Biphenylmagnesium bromide (1.6 mL, 0.5 M solution in THF, 0.8 mmol). The product **5h** was obtained as a colorless liquid (91.6 mg, 79% yield) after purification by column chromatography on silica gel with hexane/EtOAc (8:2).

^{1}H NMR (400 MHz, CDCl$_3$) δ = 7.65 (s, 1H), 7.61 – 7.55 (m, 3H), 7.49 – 7.43 (m, 4H), 7.40 – 7.34 (m, 1H), 7.32 – 7.22 (m, 6H), 7.12 (d, *J* = 6.9 Hz, 2H), 6.99 – 6.91 (m, 2H), 5.08 (dd, *J* = 8.9, 4.9 Hz, 1H), 4.15 – 4.07 (m, 1H), 4.06 – 3.97 (m, 2H), 3.93 – 3.82 (s, 1H), 3.81 (s, 3H), 3.20 – 3.04 (m, 2H), 2.66 (dd, *J* = 13.8, 8.8 Hz, 1H);

^{13}C NMR (100 MHz, CDCl$_3$) δ = 158.0, 152.6, 142.1, 140.6, 139.9, 137.8, 135.9, 129.6, 129.1(3), 129.1(0), 129.0, 127.8, 127.7, 127.4(0), 127.3(7), 126.5, 126.4, 124.0, 120.7, 120.4 – 114.3 (m), 112.9, 67.4, 56.7, 56.1, 51.8, 39.0, 32.9 (t, *J* = 20.5 Hz);

^{19}F NMR (376 MHz, CDCl$_3$) δ = -88.06 to -89.14 (m, 2F), 116.90 to -117.58 (m, 2F);

IR (film) 3062.42, 3029.96, 2958.79, 2932.07, 2840.89, 1742.86, 1603.02, 1501.32, 1480.43, 1455.44, 1416.89, 1389.57, 1348.80, 1282.21, 1260.49, 1237.24, 1188.56, 1170.24, 1107.46, 1042.33, 1024.60, 901.28, 852.80, 807.74, 736.98, 698.59, 635.23, 614.20, 560.62, 504.85, 419.13, 405.60 cm^{-1};

HRMS (APCI+) calcd for $C_{33}H_{29}F_4NO_5$ $[M+H]^+$ m/z = 580.2105; found: 580.2080.

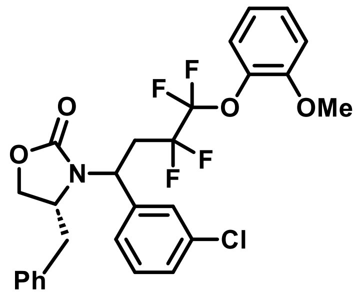

(4*R*)-4-benzyl-3-(1-(3-chlorophenyl)-3,3,4,4-tetrafluoro-4-(2-methoxyphenoxy)butyl)oxazolidin-2-one (5i): Compound **5i** was synthesized following the general procedure 2 (standard-scale), using 1-(2-bromo-1,1,2,2-tetrafluoroethoxy)-2-methoxybenzene (120.4 mg, 0.4 mmol), (*R*)-4-benzyl-3-vinyloxazolidin-2-one (40.6 mg, 0.2 mmol) and 3-chlorophenylmagnesium bromide (1.6 mL, 0.5 M solution in THF, 0.8 mmol). The product **5i** was obtained as a colorless liquid (77.5 mg, 72% yield) after purification by column chromatography on silica gel with hexane/EtOAc (8:2).

^{1}H NMR (400 MHz, CDCl$_3$) δ = 7.32 - 7.26 (m, 2H), 7.26 – 7.21 (m, 4H), 7.21 – 7.13 (m, 3H), 7.07 – 7.02 (m, 2H), 6.90 (dd, *J* = 8.6, 1.4 Hz, 1H), 6.85 (td, *J* = 7.9, 1.4 Hz, 1H), 4.88 (dd, *J* = 8.6, 5.2 Hz, 1H), 4.07 – 3.99 (m, 1H), 3.94 – 3.87 (m, 2H), 3.75 (s, 3H), 3.70 – 3.56 (td, *J* = 17.4, 8.8 Hz, 1H), 3.05 – 2.90 (m, 2H), 2.57 (dd, *J* = 13.8, 8.3 Hz, 1H);

^{13}C NMR (100 MHz, CDCl$_3$) δ = 157.9, 152.5, 141.3, 137.7, 135.7, 134.9, 130.5, 129.2, 129.0, 128.9, 127.8, 127.7, 127.5, 125.8, 123.9, 120.7, 120.4 – 114.3 (m), 112.9, 67.4, 56.7, 56.1, 51.3, 39.0, 32.8 (t, *J* = 20.5 Hz);

^{19}F NMR (376 MHz, CDCl$_3$) δ = -88.15 to -89.14 (m, 2F), 117.04 to -117.46 (m, 2F);

IR (film) 3064.67, 3028.50, 3006.10, 2958.82, 2931.92, 2841.43, 1743.72, 1602.88, 1575.00, 1501.62, 1478.64, 1455.76, 1417.03, 1389.67, 1346.87, 1303.06, 1282.27, 1260.78, 1188.95, 1170.47, 1107.70, 1042.28, 1025.78, 934.98, 853.34, 765.01, 735.35, 700.84, 607.56, 564.34, 502.84, 442.18, 422.83, 411.84 cm^{-1};

HRMS (ESI+) calcd for $C_{27}H_{24}ClF_4NO_4$ $[M+H]^+$ m/z = 538.1403; found: 538.1383.

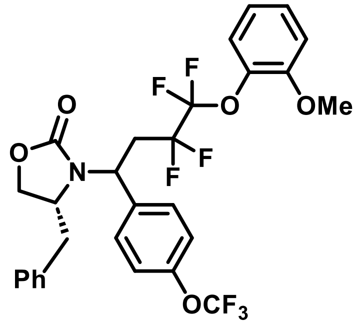

(4*R*)-4-benzyl-3-(3,3,4,4-tetrafluoro-4-(2-methoxyphenoxy)-1-(4-(trifluoromethoxy)phenyl)butyl)oxazolidin-2-one (5j): Compound **5j** was synthesized following the general procedure 2 (standard-scale), using 1-(2-bromo-1,1,2,2-tetrafluoroethoxy)-2-methoxybenzene (120.4 mg, 0.4 mmol), (*R*)-4-benzyl-3-vinyloxazolidin-2-one (40.6 mg, 0.2 mmol) and 4-(Trifluoromethoxy)phenylmagnesium bromide (1.6 mL, 0.5 M solution in THF, 0.8 mmol). The product **5j** was obtained as a colorless liquid (22.6 mg, 19% yield) after purification by column chromatography on silica gel with hexane/EtOAc (8:2).

^{1}H NMR (400 MHz, CDCl$_3$) δ = 7.48 (d, *J* = 8.7 Hz, 2H), 7.35 – 7.26 (m, 3H), 7.26 – 7.19 (m, 4H), 7.14 (d, *J* = 6.9 Hz, 2H), 6.98 (dd, *J* = 8.9, 1.4 Hz, 1H), 6.94 (td, *J* = 7.9, 1.4 Hz, 1H), 4.94 (dd, *J* = 8.3, 5.5 Hz, 1H), 4.14 – 4.08 (m, 1H), 4.02 – 3.95 (m, 2H), 3.83 (s, 3H), 3.80 – 3.63 (m, 1H), 3.18 – 3.00 (m, 2H), 2.69 (dd, *J* = 13.7, 8.6 Hz, 1H);

^{13}C NMR (100 MHz, CDCl$_3$) δ = 157.9, 152.5, 149.3(4) (q, *J* = 1.8 Hz), 138.0, 137.7, 135.6, 129.3, 129.2, 129.0, 127.8, 127.5, 123.9, 121.4, 120.8, 120.5 (q, *J* = 256.0 Hz), 120.4 – 114.3 (m), 112.9, 67.5, 56.8, 56.1, 51.3, 39.0, 32.2 (t, *J* = 20.5 Hz);

^{19}F NMR (376 MHz, CDCl$_3$) δ = -57.73 to -57.89 (m, 3F), -88.63 to -88.75 (m, 2F), 117.09 to -117.28 (m, 2F);

IR (film) 3067.90, 3029.05, 3007.34, 2928.15, 2843.86, 1747.76, 1604.88, 1503.78, 1456.25, 1417.61, 1351.25, 1259.57, 1220.31, 1170.59, 1109.14, 1043.94, 1027.16, 922.76, 854.73, 768.56, 750.11, 701.42, 612.39, 569.54, 441.04, 426.10, 409.47 cm^{-1};

HRMS (APCI+) calcd for $C_{28}H_{24}F_7NO_5$ $[M+H]^+$ m/z = 588.1615; found: 588.1591.

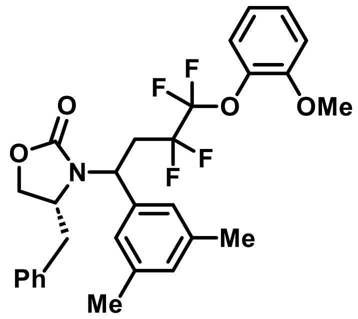

(4*R*)-4-benzyl-3-(1-(3,5-dimethylphenyl)-3,3,4,4-tetrafluoro-4-(2-methoxyphenoxy)butyl)oxazolidin-2-one (5k): Compound **5k** was synthesized following the general procedure 2 (standard-scale), using 1-(2-bromo-1,1,2,2-tetrafluoroethoxy)-2-methoxybenzene (120.4 mg, 0.4 mmol), (*R*)-4-benzyl-3-vinyloxazolidin-2-one (40.6 mg, 0.2 mmol) and 3,5-Dimethylphenylmagnesium bromide (1.6 mL, 0.5 M solution in THF, 0.8 mmol). The product **5k** was obtained as a colorless liquid (87.2 mg, 82% yield) after purification by column chromatography on silica gel with hexane/EtOAc (8:2).

^{1}H NMR (400 MHz, CDCl$_3$) δ = 7.36 – 7.28 (m, 2H), 7.28 – 7.20 (m, 3H), 7.15 – 7.10 (m, 2H), 7.05 (s, 2H), 7.00 – 6.97 (m, 2H), 6.94 (td, *J* = 7.8, 1.4 Hz, 1H), 4.98 (dd, *J* = 9.3, 4.6 Hz, 1H), 4.12 – 4.05 (m, 1H), 4.01 – 3.95 (m, 2H), 3.84 (s, 3H), 3.83 – 3.72 (m, 1H), 3.12 – 2.92 (m, 2H), 2.61 (dd, *J* = 13.8, 8.5 Hz, 1H), 2.34 (s, 6H);

^{13}C NMR (100 MHz, CDCl$_3$) δ = 158.0, 152.6, 139.3, 138.7, 137.8, 136.0, 130.2, 129.1(0), 129.0(5), 127.7, 127.3, 125.3, 124.0, 120.7, 120.4 – 114.3 (m), 112.9, 67.2, 56.6, 56.1, 51.6, 38.9, 32.7 (t, *J* = 20.5 Hz), 21.5;

^{19}F NMR (376 MHz, CDCl$_3$) δ = -88.10 to -89.32 (m, 2F), 117.38 to -117.74 (m, 2F);

IR (film) 3063.33, 3007.80, 2920.36, 2841.92, 1744.34, 1604.07, 1501.52, 1455.77, 1416.90, 1388.52, 1348.36, 1282.40, 1260.53, 1188.05, 1170.85, 1107.18, 1042.03, 1023.46, 932.00, 853.90, 736.01, 701.13, 620.67, 563.38, 502.92, 422.98 cm^{-1};

HRMS (ESI+) calcd for $C_{29}H_{29}F_4NO_4$ $[M+H]^+$ m/z = 532.2105; found: 532.2081.

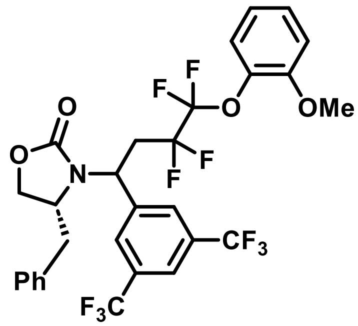

(4*R*)-4-benzyl-3-(1-(3,5-bis(trifluoromethyl)phenyl)-3,3,4,4-tetrafluoro-4-(2-methoxyphenoxy)butyl)oxazolidin-2-one (5l): Compound **5l** was synthesized following the general procedure 2 (standard-scale), using 1-(2-bromo-1,1,2,2-tetrafluoroethoxy)-2-methoxybenzene (120.4 mg, 0.4 mmol), (*R*)-4-benzyl-3-vinyloxazolidin-2-one (40.6 mg, 0.2 mmol) and 3,5-Bis(trifluoromethyl)phenylmagnesium bromide (1.6 mL, 0.5 M solution in THF, 0.8 mmol). The product **5l** was obtained as a colorless liquid (49.5 mg, 39% yield) after purification by column chromatography on silica gel with hexane/EtOAc (8:2).

^{1}H NMR (400 MHz, CDCl$_3$) δ = 7.85 (s, 1H), 7.79 (s, 2H), 7.38 – 7.28 (m, 3H), 7.24 (d, *J* = 7.6 Hz, 2H), 7.18 (d, *J* = 6.9 Hz, 2H), 7.00 – 6.96 (m, 1H), 6.94 (td, *J* = 7.9, 1.5 Hz, 1H), 4.98 (dd, *J* = 8.1, 5.7 Hz, 1H), 4.26 – 4.18 (m, 1H), 4.05 – 3.95 (m, 2H), 3.83 (s, 3H), 3.81 – 3.66 (m, 1H), 3.22 – 3.05 (m, 2H), 2.79 (dd, *J* = 14.1, 7.3 Hz, 1H);

^{13}C NMR (100 MHz, CDCl$_3$) δ = 157.9, 152.5, 141.8, 137.6, 135.6, 132.4(5) (q, *J* = 33.4 Hz), 129.4, 129.0, 128.1 (q, J = 3.2 Hz), 127.8, 127.7, 123.8, 123.1 (q, *J* = 271.2 Hz), 122.7 (qu, *J* = 3.7 Hz), 120.8, 120.4 – 114.3 (m), 112.8, 67.7, 57.1, 56.0, 51.3, 39.2, 33.1 (t, *J* = 20.5 Hz);

^{19}F NMR (376 MHz, CDCl$_3$) δ = -62.5 to -63.04 (m, 6F), -88.61 to -88.83 (m, 2F), 116.57 to -117.07 (m, 2F);

IR (film) 3066.58, 3030.02, 3007.71, 2932.63, 2843.94, 1742.74, 1604.89, 1503.11, 1456.72, 1420.45, 1375.07, 1348.07, 1276.55, 1262.05, 1169.71, 1126.36, 1107.94, 1042.85, 1026.43, 945.61, 903.68, 845.19, 766.99, 748.01, 702.92, 681.45, 624.03, 606.53, 562.84, 502.12 cm^{-1};

HRMS (ESI+) calcd for $C_{29}H_{23}F_{10}NO_4$ $[M+H]^+$ m/z = 640.1540; found: 640.1508.

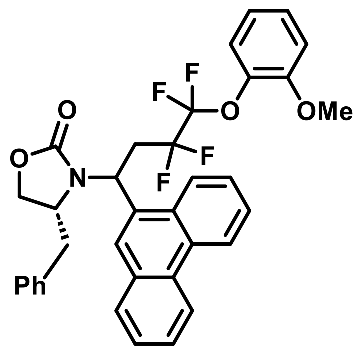

(4*R*)-4-benzyl-3-(3,3,4,4-tetrafluoro-4-(2-methoxyphenoxy)-1-(phenanthren-9-yl)butyl)oxazolidin-2-one (5m): Compound **5m** was synthesized following the general procedure 2 (standard-scale), using 1-(2-bromo-1,1,2,2-tetrafluoroethoxy)-2-methoxybenzene (120.4 mg, 0.4 mmol), (*R*)-4-benzyl-3-vinyloxazolidin-2-one (40.6 mg, 0.2 mmol) and 9-Phenanthrylmagnesium bromide (1.6 mL, 0.5 M solution in THF, 0.8 mmol). The product **5m** was obtained as a colorless liquid (53.0 mg, 44% yield) after purification by column chromatography on silica gel with hexane/EtOAc (8:2).

^{1}H NMR (400 MHz, CDCl$_3$) δ = 8.81 – 8.76 (m, 1H), 8.70 (d, *J* = 8.2 Hz, 1H), 8.45 – 8.36 (m, 1H), 8.00 – 7.94 (m, 2H), 7.76 – 7.64 (m, 4H), 7.29 – 7.23 (m, 2H), 7.19 – 7.10 (m, 3H), 6.98 – 6.92 (m, 2H), 6.90 – 6.81 (m, 2H), 6.23 (d, *J* = 8.1, 5.7 Hz, 1H), 4.12 – 3.98 (m, 3H), 3.95 – 3.80 (m, 1H), 3.78 (s, 3H), 3.24 – 3.04 (m, 1H), 2.68 – 2.54 (m, 1H), 2.29 (dd, *J* = 14.1, 7.3 Hz, 1H);

^{13}C NMR (100 MHz, CDCl$_3$) δ = 158.1, 152.5, 137.8, 135.9, 133.1, 131.0, 130.9, 130.7, 130.1, 129.0, 129.0, 128.9, 127.8, 127.7, 127.3, 127.2, 127.1, 125.6, 123.9, 123.6, 122.8, 120.8, 120.4 – 114.3 (m), 112.9, 67.3, 56.1, 55.7, 47.2, 39.0, 31.8 (t, *J* = 20.5 Hz);

^{19}F NMR (376 MHz, CDCl$_3$) δ = -88.24 to -89.20 (m, 2F), 117.29 to -117.76 (m, 2F);

IR (film) 3064.58, 3026.44, 3004.74, 2961.36, 2928.29, 2840.60, 1739.90, 1603.74, 1501.37, 1454.20, 1413.83, 1336.85, 1282.49, 1261.48, 1190.05, 1171.29, 1108.25, 1042.82, 1024.22, 977.28, 932.83, 853.45, 771.20, 747.58, 728.22, 701.46, 617.17, 566.06, 505.65, 429.28 cm^{-1};

HRMS (APCI+) calcd for $C_{35}H_{29}F_4NO_4$ $[M+H]^+$ m/z = 604.2105; found: 604.2094.

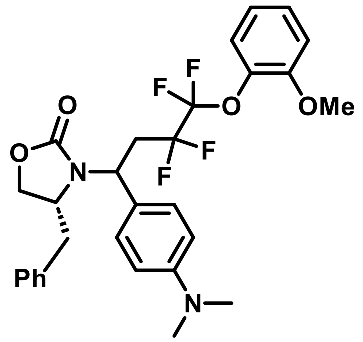

(4*R*)-4-benzyl-3-(1-(4-(dimethylamino)phenyl)-3,3,4,4-tetrafluoro-4-(2-methoxyphenoxy)butyl)oxazolidin-2-one (5n): Compound **5n** was synthesized following the general procedure 2 (standard-scale), using 1-(2-bromo-1,1,2,2-tetrafluoroethoxy)-2-methoxybenzene (120.4 mg, 0.4 mmol), (*R*)-4-benzyl-3-vinyloxazolidin-2-one (40.6 mg, 0.2 mmol) and (4-(dimethylamino)phenyl)magnesium bromide (1.6 mL, 0.5 M solution in THF, 0.8 mmol). The product **5n** was obtained as a colorless liquid (63.6 mg, 58% yield) after purification by column chromatography on silica gel with hexane/EtOAc (8:2).

^{1}H NMR (400 MHz, CDCl$_3$) δ = 7.37 – 7.20 (m, 7H), 7.11 (d, *J* = 7.0 Hz, 2H), 7.00 – 6.90 (m, 2H), 6.71 (d, *J* = 8.6 Hz, 2H), 4.98 (dd, *J* = 9.0, 5.0 Hz, 1H), 4.06 – 3.95 (m, 3H), 3.84 (s, 3H), 3.77 – 3.64 (m, 1H), 3.11 – 3.03 (m, 1H), 2.96 (s, 6H), 2.57 (dd, *J* = 13.6, 9.1 Hz, 1H);

^{13}C NMR (100 MHz, CDCl$_3$) δ = 158.0, 152.6, 150.6, 137.9, 136.1, 129.2, 129.1, 129.0, 128.5, 127.6, 127.2, 126.7, 124.0, 120.7, 120.4 – 114.3 (m), 112.9, 112.5, 67.2, 56.4, 56.2, 51.2, 40.5, 32.8 (t, *J* = 20.5 Hz);

^{19}F NMR (376 MHz, CDCl$_3$) δ = -88.31 to -89.16 (m, 2F), 117.13 to -117.84 (m, 2F);

IR (film) 3064.93, 3027.19, 2922.84, 2842.66, 2807.38, 1744.08, 1613.21, 1523.80, 1501.66, 1455.57, 1417.37, 1352.37, 1325.38, 1282.36, 1261.25, 1222.09, 1189.18, 1170.05, 1107.74, 1043.00, 1026.18, 947.27, 817.81, 750.12, 701.78, 570.09, 505.04, 422.56, 405.40 cm^{-1};
HRMS (APCI+) calcd for $C_{29}H_{30}F_4N_2O_4$ $[M+H]^+$ m/z = 547.2214; found: 547.2206.

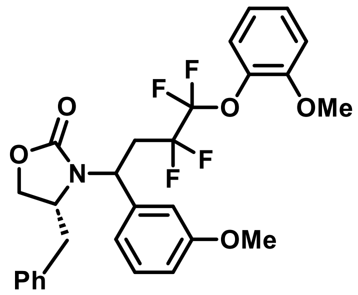

(4*R*)-4-benzyl-3-(3,3,4,4-tetrafluoro-4-(2-methoxyphenoxy)-1-(3-methoxyphenyl)butyl)oxazolidin-2-one (5o): Compound **5o** was synthesized following the general procedure 2 (standard-scale), using 1-(2-bromo-1,1,2,2-tetrafluoroethoxy)-2-methoxybenzene (120.4 mg, 0.4 mmol), (*R*)-4-benzyl-3-vinyloxazolidin-2-one (40.6 mg, 0.2 mmol) and 3-methoxyphenylmagnesium bromide (0.8 mL, 1.0 M solution in THF, 0.8 mmol). The product **5o** was obtained as a colorless liquid (91.7 mg, 86% yield) after purification by column chromatography on silica gel with hexane/EtOAc (8:2).

^{1}H NMR (400 MHz, $CDCl_3$) δ = 7.32 – 7.22 (m, 6H), 7.13 – 7.09 (m, 2H), 7.06 – 7.02 (m, 2H), 6.99 – 6.95 (m, 1H), 6.95 – 6.91 (m, 1H), 6.90 – 6.87 (m, 1H), 5.01 (dd, *J* = 9.1, 4.8 Hz, 1H), 4.12 – 4.05 (m, 1H), 4.02 – 3.96 (m, 2H), 3.83 (s, 3H), 3.82 (s, 3H), 3.80 – 3.67 (m, 1H), 3.16 – 2.98 (m, 2H), 2.60 (dd, *J* = 13.7, 8.8 Hz, 1H);

^{13}C NMR (100 MHz, $CDCl_3$) δ = 160.2, 158.0, 152.5, 140.9, 137.7, 135.8, 130.1, 129.0, 127.7, 127.3, 123.9, 120.7, 120.4 – 114.3 (m), 119.7, 113.9, 113.4, 112.9, 67.3, 56.5, 56.1, 55.4, 51.6, 38.9, 32.7 (t, *J* = 20.5 Hz);

^{19}F NMR (376 MHz, $CDCl_3$) δ = -88.24 to -89.11 (m, 2F), 117.16 to -117.51 (m, 2F);
IR (film) 3063.25, 3028.18, 3005.73, 2943.96, 2839.76, 2168.25, 2158.06, 1743.42, 1601.86, 1586.15, 1501.51, 1455.72, 1438.13, 1389.37, 1349.10, 1281.84, 1260.05, 1188.42, 1170.32, 1107.28, 1041.27, 929.43, 854.76, 765.07, 737.00, 737.00, 701.12, 607.10, 559.24, 504.14, 456.02 cm^{-1};
HRMS (APCI+) calcd for $C_{28}H_{27}F_4NO_5$ $[M+H]^+$ m/z = 534.1898; found: 534.1877.

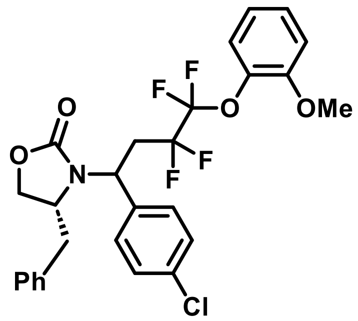

(4*R*)-4-benzyl-3-(1-(4-chlorophenyl)-3,3,4,4-tetrafluoro-4-(2-methoxyphenoxy)butyl)oxazolidin-2-one (5p): Compound **5p** was synthesized following the general procedure 2 (standard-scale), using 1-(2-bromo-1,1,2,2-tetrafluoroethoxy)-2-methoxybenzene (120.4 mg, 0.4 mmol), (*R*)-4-benzyl-3-vinyloxazolidin-2-one (40.6 mg, 0.2 mmol) and 4-chlorophenylmagnesium bromide (0.8 mL, 1.0 M solution in diethyl ether, 0.8 mmol). The product **5p** was obtained as a colorless liquid (70.8 mg, 66% yield) after purification by column chromatography on silica gel with hexane/EtOAc (8:2).

^{1}H NMR (400 MHz, $CDCl_3$) δ = 7.41 – 7.30 (m, 6H), 7.28 – 7.22 (m, 3H), 7.15 – 7.09 (m, 2H), 6.98 (dd, *J* = 8.6, 1.3 Hz, 1H), 6.94 (td, *J* = 7.9, 1.4 Hz, 1H), 4.94 (dd, *J* = 8.4, 5.5 Hz, 1H), 4.12 – 4.05 (m, 1H), 4.03 – 3.94 (m, 2H), 3.83 (s, 3H), 3.77 – 3.62 (m, 1H), 3.16 – 3.00 (m, 2H), 2.65 (dd, *J* = 13.7, 8.8 Hz, 1H);

^{13}C NMR (100 MHz, $CDCl_3$) δ = 158.0, 152.5, 137.8, 137.7, 135.7, 134.6, 129.3, 129.2, 129.1, 129.0, 127.8, 127.4, 123.9, 120.7, 120.4 – 114.3 (m), 112.9, 67.4, 56.7, 56.1, 51.3, 39.0, 33.0 (t, *J* = 20.5 Hz);

^{19}F NMR (376 MHz, $CDCl_3$) δ = -88.29 to -89.08 (m, 2F), 116.95 to -117.55 (m, 2F);
IR (film) 3063.96, 3028.73, 3005.83, 2931.33, 2841.56, 1743.14, 1603.72, 1501.15, 1455.74, 1413.34, 1348.26, 1315.27, 1282.18, 1260.41, 1188.57, 1170.30, 1091.33, 1042.12, 1025.66, 1014.53, 828.62, 767.33, 738.00, 700.73, 651.13, 631.65, 614.59, 569.02, 509.16, 445.51 cm^{-1};

HRMS (APCI+) calcd for $C_{27}H_{24}ClF_4NO_4$ $[M+H]^+$ m/z = 538.1403; found: 538.1380.

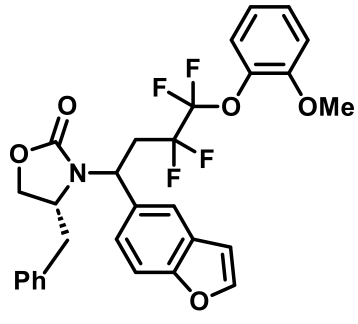

(4*R*)-3-(1-(benzofuran-5-yl)-3,3,4,4-tetrafluoro-4-(2-methoxyphenoxy)butyl)-4-benzyloxazolidin-2-one (5q): Compound **5q** was synthesized following the general procedure 2 (standard-scale), using 1-(2-bromo-1,1,2,2-tetrafluoroethoxy)-2-methoxybenzene (120.4 mg, 0.4 mmol), (*R*)-4-benzyl-3-vinyloxazolidin-2-one (40.6 mg, 0.2 mmol) and benzofuran-5-ylmagnesium bromidelithium chloride complex (1.05 mL, 0.76 M solution in THF, 0.8 mmol). The product **5q** was obtained as a colorless liquid (51.6 mg, 48% yield) after purification by column chromatography on silica gel with hexane/EtOAc (8:2).

^{1}H NMR (400 MHz, $CDCl_3$) δ = 7.72 (d, *J* = 1.6 Hz, 1H), 7.65 (d, *J* = 2.1 Hz, 1H), 7.51 (d, *J* = 8.6 Hz, 1H), 7.42 (dd, *J* = 8.6, 1.8 Hz, 1H), 7.32 – 7.22 (m, 5H), 7.15 – 7.10 (m, 2H), 6.99 – 6.95 (m, 1H), 6.93 (td, *J* = 7.8, 1.4 Hz, 1H), 6.78 (dd, *J* = 2.2, 0.8 Hz, 1H), 5.10 (dd, *J* = 8.8, 5.3 Hz, 1H), 4.10 – 3.95 (m, 3H), 3.92 – 3.76 (m, 4H), 3.22 – 3.07 (m, 2H), 2.64 (dd, *J* = 13.6, 8.6 Hz, 1H);

^{13}C NMR (100 MHz, $CDCl_3$) δ = 158.1, 154.8, 152.6, 146.0, 137.8, 135.8, 134.1, 129.0(9), 129.0(7), 128.0, 127.7, 127.3, 124.0, 123.9, 120.7, 120.4 – 114.3 (m), 120.3, 112.9, 111.9, 106.9, 67.3, 56.6, 56.1, 51.9, 38.9, 33.4 (t, *J* = 20.5 Hz);

^{19}F NMR (376 MHz, $CDCl_3$) δ = -88.55 to -88.86 (m, 2F), 117.14 to -117.56 (m, 2F);

IR (film) 3117.36, 3064.81, 3027.64, 3005.47, 2931.52, 2841.34, 1742.19, 1603.97, 1501.35, 1455.44, 1389.85, 1333.38, 1282.54, 1260.28, 1188.40, 1170.47, 1106.93, 1042.37, 1026.13, 933.17, 886.12, 813.15, 764.95, 739.96, 700.08, 645.85, 607.43, 525.18, 504.56, 431.93, 412.97 cm^{-1};

HRMS (ESI+) calcd for $C_{29}H_{25}F_4NO_5$ $[M+H]^+$ m/z = 544.1742; found: 544.1726.

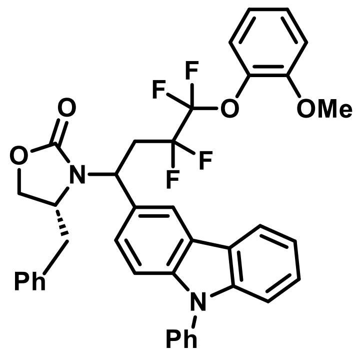

(4*R*)-3-(1-(benzofuran-5-yl)-3,3,4,4-tetrafluoro-4-(2-methoxyphenoxy)butyl)-4-benzyloxazolidin-2-one (5r): Compound **5r** was synthesized following the general procedure 2 (standard-scale), using 1-(2-bromo-1,1,2,2-tetrafluoroethoxy)-2-methoxybenzene (120.4 mg, 0.4 mmol), (*R*)-4-benzyl-3-vinyloxazolidin-2-one (40.6 mg, 0.2 mmol) and (9-phenyl-9*H*-carbazol-3-yl)magnesium bromide (0.8 mL, 1.0 M solution in THF, 0.8 mmol). The product **5r** was obtained as a colorless liquid (84.8 mg, 63% yield) after purification by column chromatography on silica gel with hexane/EtOAc (8:2).

^{1}H NMR (400 MHz, $CDCl_3$) δ = 8.18 – 8.14 (m, 1H), 8.13 (d, *J* = 1.7 Hz, 1H), 7.64 – 7.59 (m, 2H), 7.58 – 7.53 (m, 3H), 7.50 – 7.45 (m, 1H), 7.44 – 7.38 (m, 3H), 7.35 – 7.19 (m, 8H), 7.15 – 7.10 (m, 2H), 6.98 (dd, *J* = 8.3, 1.4 Hz, 1H), 6.94 (td, *J* = 7.7, 1.4 Hz, 1H), 5.23 (dd, *J* = 8.9, 5.1 Hz, 1H), 4.11 – 3.97 (m, 3H), 3.96 – 3.86 (m, 1H), 3.84 (s, 3H), 3.27 – 3.09 (m, 2H), 2.64 (dd, *J* = 13.7, 8.5 Hz, 1H);

^{13}C NMR (100 MHz, $CDCl_3$) δ = 158.1, 152.6, 141.5, 140.8, 137.9, 137.5, 136.1, 131.1, 130.1, 129.1(4), 129.1(0), 127.8, 127.7, 127.3, 127.2, 126.5, 125.6, 124.0, 123.6, 123.2, 120.7, 120.6, 120.4 – 114.3 (m), 120.3, , 119.5, 112.9, 110.4, 110.1, 67.3, 56.5, 56.2, 52.1, 33.3 (t, *J* = 20.5 Hz);

^{19}F NMR (376 MHz, $CDCl_3$) δ = -88.27 to -88.79 (m, 2F), 117.10 to -117.53 (m, 2F);

IR (film) 3062.08, 3028.15, 2957.58, 2840.38, 1742.47, 1598.11, 1500.84, 1454.48, 1438.19, 1360.91, 1332.50, 1302.61, 1281.80, 1261.53, 1232.43, 1188.69, 1170.65, 1107.64, 1042.66, 1025.66, 929.83, 852.24, 813.31, 733.43, 697.43, 642.35, 567.84, 526.62, 495.79, 454.94, 424.89 cm^{-1};
HRMS (ESI+) calcd for $C_{39}H_{32}F_4N_2O_4$ $[M+H]^+$ m/z = 669.2371; found: 669.2346.

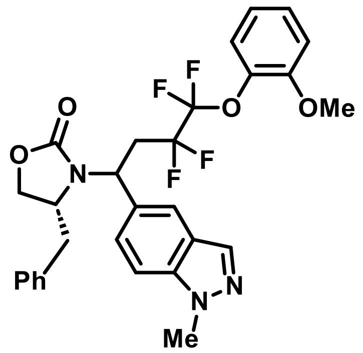

(4*R*)-4-benzyl-3-(3,3,4,4-tetrafluoro-4-(2-methoxyphenoxy)-1-(1-methyl-1H-indazol-5-yl)butyl)oxazolidin-2-one (5s): Compound **5s** was synthesized following the general procedure 2 (standard-scale), using 1-(2-bromo-1,1,2,2-tetrafluoroethoxy)-2-methoxybenzene (120.4 mg, 0.4 mmol), (*R*)-4-benzyl-3-vinyloxazolidin-2-one (40.6 mg, 0.2 mmol) and (1-methyl-1*H*-indazol-5-yl)magnesium bromide lithium chloride complex (1.15 mL, 0.7 M solution in THF, 0.8 mmol). The product **5s** was obtained as a colorless liquid (54.1 mg, 49% yield) after purification by column chromatography on silica gel with hexane/EtOAc (6:4).
^{1}H NMR (400 MHz, $CDCl_3$) δ = 7.98 (s, 1H), 7.70 (s, 1H), 7.61 (dd, *J* = 8.8, 1.6 Hz, 1H), 7.42 (d, *J* = 8.8 Hz, 1H), 7.31 – 7.22 (m, 5H), 7.12 – 7.07 (m, 2H), 6.99 – 6.91 (m, 2H), 5.12 (dd, *J* = 8.5, 5.5 Hz, 1H), 4.12 – 4.03 (m, 4H), 4.02 – 3.92 (m, 2H), 3.83 (s, 3H), 3.80 – 3.70 (m, 1H), 3.24 – 3.06 (m, 2H), 2.62 (dd, *J* = 13.8, 8.5 Hz, 1H);
^{13}C NMR (100 MHz, $CDCl_3$) δ = 158.1, 152.5, 139.8, 137.8, 135.8, 133.1, 131.7, 129.1, 129.0, 127.7, 127.3, 126.3, 124.0, 123.9, 120.7, 120.4 – 114.3 (m), 119.8, 112.9, 109.9, 67.3, 56.5, 56.1, 51.9, 38.9, 35.7, 33.2 (t, *J* = 20.5 Hz);
^{19}F NMR (376 MHz, $CDCl_3$) δ = -88.51 to -88.92 (m, 2F), 117.10 to -117.54 (m, 2F);
IR (film) 3062.73, 3027.67, 3004.80, 2937.55, 2841.37, 1740.77, 1603.98, 1501.23, 1455.69, 1415.76, 1385.67, 1347.66, 1282.32, 1260.58, 1223.13, 1188.09, 1170.30, 1106.32, 1042.26, 1025.08, 938.69, 913.24, 889.36, 810.15, 766.17, 737.84, 699.84, 619.75, 567.94, 523.78, 504.73, 433.49 cm^{-1};
HRMS (APCI+) calcd for $C_{29}H_{27}F_4N_3O_4$ $[M+H]^+$ m/z = 558.2010; found: 558.2000.

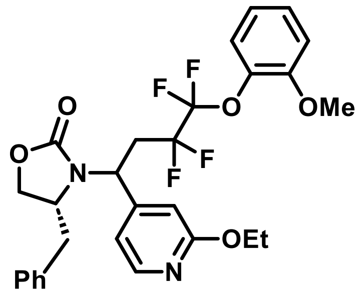

(4*R*)-4-benzyl-3-(1-(2-ethoxypyridin-4-yl)-3,3,4,4-tetrafluoro-4-(2-methoxyphenoxy)butyl)oxazolidin-2-one (5t): Compound **5t** was synthesized following the general procedure 2 (standard-scale), using 1-(2-bromo-1,1,2,2-tetrafluoroethoxy)-2-methoxybenzene (120.4 mg, 0.4 mmol), (*R*)-4-benzyl-3-vinyloxazolidin-2-one (40.6 mg, 0.2 mmol) and (2-ethoxypyridin-4-yl)magnesium bromide lithium chloride complex (0.93 mL, 0.86 M solution in THF, 0.8 mmol). The product **5t** was obtained as a colorless liquid (80.1 mg, 73% yield) after purification by column chromatography on silica gel with hexane/EtOAc (8:2).
^{1}H NMR (400 MHz, $CDCl_3$) δ = 8.15 (d, *J* = 5.4 Hz, 1H), 7.33 – 7.22 (m, 5H), 7.15 – 7.08 (m, 2H), 6.99 – 6.92 (m, 3H), 6.74 (s, 1H), 4.94 (dd, *J* = 8.8, 4.9 Hz, 1H), 4.36 (q, *J* = 7.1 Hz, 2H), 4.16 – 4.09 (m, 1H), 4.04 – 3.95 (m, 2H), 3.84 (s, 3H), 3.73 – 3.60 (m, 1H), 3.16 – 2.98 (m, 2H), 2.65 (dd, *J* = 13.7, 8.7 Hz, 1H), 1.40 (t, *J* = 7.0 Hz, 3H);
^{13}C NMR (100 MHz, $CDCl_3$) δ = 164.6, 157.9, 152.5, 150.6, 147.9, 137.6, 135.4, 129.1, 129.0, 127.7, 127.4, 123.8, 120.7, 120.4 – 114.3 (m), 115.4, 112.8, 109.5, 67.4, 62.0, 56.8, 56.0, 50.6, 39.0, 32.3 (t, *J* = 20.5 Hz), 14.7;

^{19}F NMR (376 MHz, CDCl$_3$) δ = -88.18 to -88.82 (m, 2F), 116.90 to -117.28 (m, 2F);
IR (film) 3063.01, 3027.94, 2978.94, 2933.36, 2841.94, 1746.21, 1607.48, 1560.15, 1501.89, 1482.05, 1455.92, 1419.12, 1381.65, 1342.23, 1318.27, 1282.62, 1260.32, 1189.04, 1170.48, 1107.55, 1041.86, 882.42, 853.69, 767.14, 734.72, 700.46, 607.18, 561.21, 526.30, 504.02, 459.17 cm^{-1};
HRMS (APCI+) calcd for $C_{28}H_{28}F_4N_2O_5$ $[M+H]^+$ m/z = 549.2007; found: 549.1992.

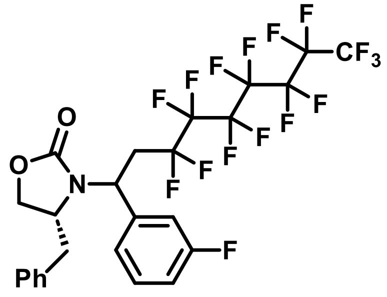

(4*R*)-4-benzyl-3-(3,3,4,4,5,5,6,6,7,7,8,8,9,9,9-pentadecafluoro-1-(3-fluorophenyl)nonyl)oxazolidin-2-one (6a): Compound **6a** was synthesized following the general procedure 2 (standard-scale), using 1-Bromoperfluoroheptane (179.6 mg, 0.4 mmol), (*R*)-4-benzyl-3-vinyloxazolidin-2-one (40.6 mg, 0.2 mmol) and 3-Fluorophenylmagnesium bromide (1.6 mL, 0.5 M solution in THF, 0.8 mmol). The product **6a** was obtained as a colorless liquid (100.1 mg, 75% yield) after purification by column chromatography on silica gel with hexane/EtOAc (8:2).
^{1}H NMR (400 MHz, CDCl$_3$) δ = 7.39 – 7.26(m, 4H), 7.17 – 7.11 (m, 3H), 7.12 – 7.02 (m, 2H), 4.73 (dd, *J* = 8.4, 5.4 Hz, 1H), 4.12 (t, *J* = 8.3 Hz, 1H), 4.03 – 3.96 (m, 1H), 3.96 – 3.86 (m, 1H), 3.83 – 3.65(m, 1H), 3.12 (dd, *J* = 13.8, 5.0 Hz, 1H), 2.94 – 2.77 (m, 1H), 2.70 (dd, *J* = 13.8, 8.6 Hz, 1H);
^{13}C NMR (100 MHz, CDCl$_3$) δ = 163.1 (d, *J* = 246.6 Hz), 158.0, 140.9 (d, *J* = 6.7 Hz), 135.5, 130.9 (d, *J* = 8.2 Hz), 129.3, 129.0, 127.6, 123.2 (d, *J* = 2.8 Hz), 116.05 (d, *J* = 20.9 Hz), 114.7 (d, *J* = 22.0 Hz), 67.5, 56.9, 51.4, 39.0, 32.8 (t, *J* = 20.1 Hz);
^{19}F NMR (376 MHz, CDCl$_3$) δ = -80.70 to -80.84 (m, 3F), -110.87 – 111.20 (m, 1F), -112.91 to -115.42 (m, 2F), -121.42 to -121.68 (m, 2F), -121.95 to -121.16 (m, 2F), -122.57 to -122.85 (m, 2F), -123.37 to -123.58 (m, 2F), -126.01 to -126.22 (m, 2F);
IR (film) 3066.89, 3030.82, 2958.42, 2923.73, 2853.78, 1743.12, 1593.25, 1487.78, 1454.70, 1420.05, 1366.32, 1235.26, 1203.60, 1146.26, 1072.97, 1042.57, 958.35, 883.02, 789.99, 765.53, 738.75, 700.37, 656.41, 570.02, 522.99, 503.71 cm^{-1};
HRMS (APCI+) calcd for $C_{25}H_{17}F_{16}NO_2$ $[M+H]^+$ m/z = 668.1077; found: 668.1059.

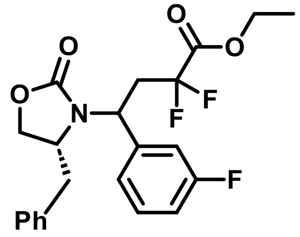

Ethyl 4-((R)-4-benzyl-2-oxooxazolidin-3-yl)-2,2-difluoro-4-(3-fluorophenyl)butanoate (6b): Compound **6b** was synthesized following the general procedure 2 (standard-scale), using 2-bromo-2,2-difluoroacetate (81.2 mg, 0.4 mmol), (*R*)-4-benzyl-3-vinyloxazolidin-2-one (40.6 mg, 0.2 mmol) and 3-Fluorophenylmagnesium bromide (1.6 mL, 0.5 M solution in THF, 0.8 mmol). The product **6b** was obtained as a colorless liquid (49.7 mg, 59% yield) after purification by column chromatography on silica gel with hexane/EtOAc (8:2).
^{1}H NMR (400 MHz, CDCl$_3$) δ = 7.30 – 7.22 (m, 3H), 7.22 – 7.17 (m, 1H), 7.11 – 7.01 (m, 4H), 6.96 (tdd, *J* = 8.3, 2.6, 1.0 Hz, 1H), 4.68 (dd, *J* = 8.8, 5.4 Hz, 1H), 4.17 (q, *J* = 7.1 Hz, 2H), 4.05 – 3.97 (m, 1H), 3.91 – 3.80 (m, 2H), 3.60 – 3.44 (m, 1H), 2.98 (dd, *J* = 13.8, 4.7 Hz, 1H), 2.89 – 2.74 (m, 1H), 2.57 (dd, *J* = 13.8, 8.8 Hz, 1H), 1.26 (t, *J* = 7.1 Hz, 3H).
^{13}C NMR (100 MHz, CDCl$_3$) δ = 163.7 (t, *J* = 32.3 Hz), 163.1 (d, *J* = 246.6 Hz), 157.9, 141.0 9 (d, *J* = 6.7 Hz), 135.6, 130.7 (d, *J* = 8.2 Hz), 129.2, 129.1, 127.5, 123.4 (d, *J* = 2.8 Hz), 115.8 (d, *J* = 20.9 Hz), 115.1 (t, *J* = 250.1 Hz), 114.9 (d, *J* = 22.0 Hz), 67.4, 63.4, 56.7, 51.9, 38.7, 36.4 (t, *J* = 20.1 Hz), 14.0;
^{19}F NMR (376 MHz, CDCl$_3$) δ = = -104.24 to -106.11 (m, 2F), -111.17 – 111.50 (m, 1F);
IR (film) 3063.59, 3028.82, 2982.94, 2935.19, 1745.86, 1615.11, 1591.99, 1488.34, 1429.08, 1374.08, 1298.38, 1234.98, 1189.71, 1149.85, 1069.30, 1029.72, 956.41, 879.24, 852.74, 767.16, 739.57, 701.95, 521.26, 505.69, 452.10 cm^{-1};

HRMS (ESI+) calcd for $C_{22}H_{22}F_3NO_4$ $[M+H]^+$ m/z = 422.1574; found: 422.1561.

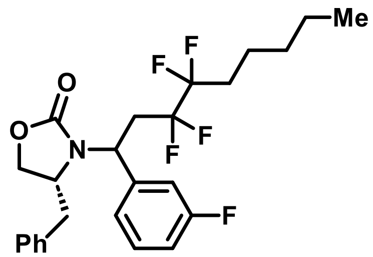

(4*R*)-4-benzyl-3-(3,3,4,4-tetrafluoro-1-(3-fluorophenyl)nonyl)oxazolidin-2-one (6c): Compound **6c** was synthesized following the general procedure 2 (standard-scale), using 1-Bromo-1,1,2,2-tetrafluoroheptane (100.4 mg, 0.4 mmol), (*R*)-4-benzyl-3-vinyloxazolidin-2-one (40.6 mg, 0.2 mmol) and 3-Fluorophenylmagnesium bromide (1.6 mL, 0.5 M solution in THF, 0.8 mmol). The product **6c** was obtained as a colorless liquid (68.5 mg, 73% yield) after purification by column chromatography on silica gel with hexane/EtOAc (8:2).

^{1}H NMR (400 MHz, CDCl$_3$) δ = 7.35 – 7.26 (m, 4H), 7.22 – 7.17 (m, 1H), 7.16 – 7.07(m, 3H), 7.05 – 6.99 (m, 1H), 4.92 (dd, *J* = 8.9, 5.1 Hz, 1H), 4.14 – 4.05 (m, 1H), 4.02 – 3.87(m, 2H), 3.56 – 3.38 (m, 1H), 3.03 (dd, *J* = 13.8, 4.4 Hz, 1H), 2.88 – 2.71 (m, 1H), 2.59 (dd, *J* = 13.7, 8.9 Hz, 1H), 2.07 – 1.90 (m, 2H), 1.65 – 1.50 (m, 2H), 1.38 – 1.29 (m, 4H), 0.91 (t, *J* = 7.0 Hz, 3H);

^{13}C NMR (100 MHz, CDCl$_3$) δ = 163.1 (d, *J* = 246.6 Hz), 158.0, 140.9 (d, *J* = 6.7 Hz), 135.6, 130.7 (d, *J* = 8.2 Hz), 129.2, 129.0, 127.5, 123.2 (d, *J* = 2.8 Hz), 115.6 (d, *J* = 20.9 Hz), 114.7 (d, *J* = 22.0 Hz), 67.4, 56.6, 51.3, 39.0, 31.6, 29.9 (t, *J* = 20.1 Hz), 22.5, 20.2, 14.0;

^{19}F NMR (376 MHz, CDCl$_3$) δ = -111.37 to -111.57 (m, 1F), -114.25 to -116.63 (m, 4F);

IR (film) 3064.65, 3029.43, 2958.08, 2935.51, 2873.34, 1742.90, 1615.30, 1592.14, 1487.73, 1453.98, 1418.08, 1381.35, 1232.11, 1174.84, 1072.01, 1031.73, 957.02, 878.23, 788.56, 765.11, 740.29,700.82, 583.34, 521.64, 504.66, 424.97 cm^{-1};

HRMS (APCI+) calcd for $C_{25}H_{28}F_5NO_2$ $[M+H]^+$ m/z = 470.2118; found: 470.2097.

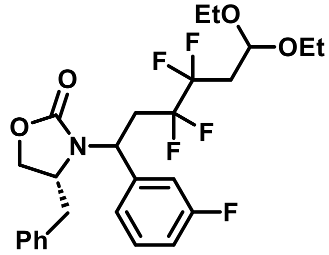

(4*R*)-4-benzyl-3-(6,6-diethoxy-3,3,4,4-tetrafluoro-1-(3-fluorophenyl)hexyl)oxazolidin-2-one (6d): Compound **6d** was synthesized following the general procedure 2 (standard-scale), using 1-bromo-4,4-diethoxy-1,1,2,2-tetrafluorobutane (98.8 mg, 0.4 mmol), (*R*)-4-benzyl-3-vinyloxazolidin-2-one (40.6 mg, 0.2 mmol) and 3-Fluorophenylmagnesium bromide (1.6 mL, 0.5 M solution in THF, 0.8 mmol). The product **6d** was obtained as a colorless liquid (73.2 mg, 71% yield) after purification by column chromatography on silica gel with hexane/EtOAc (8:2).

^{1}H NMR (400 MHz, CDCl$_3$) δ = 7.37 – 7.24 (m, 4H), 7.18 (d, *J* = 7.9 Hz, 1H), 7.16 – 7.06 (m, 3H), 7.06 – 7.01 (m, 1H), 4.94 – 4.86 (m, 2H), 4.14 – 4.04 (m, 1H), 4.00 – 3.89 (m, 2H), 3.71 – 3.62 (m, 2H), 3.59 – 3.43 (m, 3H), 3.02 (dd, *J* = 13.8, 4.4 Hz, 1H), 2.90 – 2.72 (m, 1H), 2.59 (dd, *J* = 13.6, 9.0 Hz, 1H), 2.39 (td, *J* = 18.8, 5.4 Hz, 2H), 1.21 (t, *J* = 7.1 Hz, 6H);

^{13}C NMR (100 MHz, CDCl$_3$) δ = 163.1 (d, *J* = 246.6 Hz), 157.9, 141.6 (d, *J* = 6.7 Hz), 135.6, 130.7 (d, *J* = 8.2 Hz), 129.2, 129.0, 127.5, 123.2 (d, *J* = 2.8 Hz), 115.7 (d, *J* = 20.9 Hz), 114.7 (d, *J* = 22.0 Hz), 97.4, 67.3, 61.8, 56.6, 51.3, 39.0, 34.7 (t, *J* = 20.5 Hz), 31.3 (t, *J* = 20.1 Hz), 15.3.

^{19}F NMR (376 MHz, CDCl$_3$) δ = -111.31 to -111.52 (m, 1F), -114.09 to -114.34 (m, 2F), -114.37 to -116.62 (m, 2F);

IR (film) 3064.39, 3029.39, 2977.66, 2929.52, 1743.37, 1615.37, 1592.17, 1487.60, 1453.83, 1416.99, 1377.06, 1232.36, 1176.68, 1094.01, 1055.84, 961.55, 894.67, 823.33, 790.18, 763.89, 739.70, 700.98, 584.36, 521.72, 503.61, 434.90 cm^{-1};

HRMS (ESI+) calcd for $C_{26}H_{30}F_5NO_4$ $[M+Na]^+$ m/z = 538.1987; found: 538.1965.

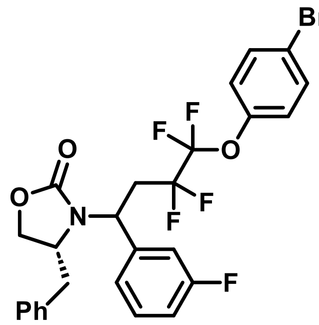

(4*R*)-4-benzyl-3-(4-(4-bromophenoxy)-3,3,4,4-tetrafluoro-1-(3-fluorophenyl)butyl)oxazolidin-2-one (6e): Compound **6e** was synthesized following the general procedure 2 (standard-scale), using 1-bromo-4-(2-bromo-1,1,2,2-tetrafluoroethoxy)benzene (140.6 mg, 0.4 mmol), (*R*)-4-benzyl-3-vinyloxazolidin-2-one (40.6 mg, 0.2 mmol) and 3-Fluorophenylmagnesium bromide (1.6 mL, 0.5 M solution in THF, 0.8 mmol). The product **6e** was obtained as a colorless liquid (68.5 mg, 60% yield) after purification by column chromatography on silica gel with hexane/EtOAc (8:2).

^{1}H NMR (400 MHz, $CDCl_3$) δ = 7.50 (d, *J* = 8.9 Hz, 2H), 7.36 – 7.28 (m, 4H), 7.22 – 7.17 (m, 1H), 7.17 – 7.11 (m, 3H), 7.11 – 7.03 (m, 3H), 4.85 (dd, *J* = 8.4, 5.4 Hz, 1H), 4.14 – 4.09 (m, 1H), 4.02 – 3.93 (m, 2H), 3.81 – 3.63 (m, 1H), 3.13 (dd, *J* = 13.7, 4.7 Hz, 1H), 3.01 – 2.85 (m, 1H), 2.69 (dd, *J* = 13.8, 8.6 Hz, 1H);

^{13}C NMR (100 MHz, $CDCl_3$) δ = 163.1 (d, *J* = 246.6 Hz), 158.0, 148.0, 141.3 (d, *J* = 6.7 Hz), 135.5, 132.9, 130.8 (d, *J* = 8.2 Hz), 129.2, 129.0, 127.5, 123.6, 123.3 (d, *J* = 2.8 Hz), 120.4 – 114.3 (m), 120.0, 115.8 (d, *J* = 20.9 Hz), 114.8 (d, *J* = 22.0 Hz), 67.4, 56.8, 51.5, 38.9, 32.9 (t, *J* = 20.1 Hz);

^{19}F NMR (376 MHz, $CDCl_3$) δ = -88.29 to -88.62 (m, 2F), -114.14 to -114.32 (m, 1F), -116.39 to -118.74 (m, 2F);

IR (film) 3064.85, 3029.64, 3005.58, 2960.70, 2918.68, 1742.78, 1615.17, 1591.88, 1484.51, 1453.84, 1418.76, 1389.70, 1348.83, 1312.19, 1228.28, 1182.52, 1108.99, 1093.83, 1067.89, 1043.90, 1012.18, 955.06, 878.34, 852.85, 829.78, 782.55, 764.49, 737.79, 700.67, 646.30, 584.97, 532.77, 521.35, 493.77 cm^{-1};

HRMS (ESI+) calcd for $C_{26}H_{21}BrF_5NO_3$ $[M+H]^+$ m/z = 570.0698; found: 570.0688.

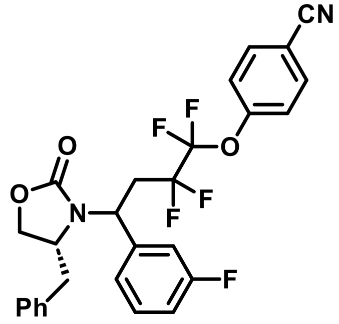

4-(4-((*R*)-4-benzyl-2-oxooxazolidin-3-yl)-1,1,2,2-tetrafluoro-4-(3-fluorophenyl)butoxy)benzonitrile (6f): Compound **6f** was synthesized following the general procedure 2 (standard-scale), using 4-(2-bromo-1,1,2,2-tetrafluoroethoxy)benzonitrile (119.2 mg, 0.4 mmol), (*R*)-4-benzyl-3-vinyloxazolidin-2-one (40.6 mg, 0.2 mmol) and 3-Fluorophenylmagnesium bromide (1.6 mL, 0.5 M solution in THF, 0.8 mmol). The product **6f** was obtained as a colorless liquid (63.0 mg, 61% yield) after purification by column chromatography on silica gel with hexane/EtOAc (8:2).

^{1}H NMR (400 MHz, $CDCl_3$) δ = 7.70 (d, *J* = 8.9 Hz, 2H), 7.38 – 7.27 (m, 6H), 7.20 – 7.10 (m, 4H), 7.05 (td, *J* = 8.3, 2.9 Hz, 1H), 4.82 (dd, *J* = 8.4, 5.4 Hz, 1H), 4.16 – 4.10 (m, 1H), 4.03 – 3.91 (m, 2H), 3.85 – 3.67 (m, 1H), 3.14 (dd, *J* = 13.9, 4.7 Hz, 1H), 3.00 – 2.83 (m, 1H), 2.70 (dd, *J* = 13.6, 8.5 Hz, 1H);

^{13}C NMR (100 MHz, $CDCl_3$) δ = 163.1 (d, *J* = 246.6 Hz), 158.0, 152.3, 141.2 (d, *J* = 6.7 Hz), 135.5, 134.1, 130.9 (d, *J* = 8.2 Hz), 129.2, 129.0, 127.5, 123.2 (d, *J* = 2.8 Hz), 122.2, 120.4 – 114.3 (m), 117.9, 115.9 (d, *J* = 20.9 Hz), 114.7 (d, *J* = 22.0 Hz), 110.6, 67.5, 56.9, 51.5, 38.9, 32.9 (t, *J* = 20.1 Hz);

^{19}F NMR (376 MHz, $CDCl_3$) δ = -88.38 to -88.50 (m, 2F), -111.07 to -111.20 (m, 1F), -116.39 to -118.74 (m, 2F);

IR (film) 3108.09, 3064.57, 3029.62, 3005.89, 2959.54, 2923.03, 2232.19, 1742.95, 1592.29, 1498.62, 1453.97, 1418.28, 1390.05, 1348.12, 1308.93, 1227.84, 1172.82, 1115.71, 1043.69, 999.81, 954.10, 865.65, 842.49, 788.17, 764.78, 734.33, 700.52, 584.16, 548.92, 521.57, 503.72, 479.78 cm^{-1};

HRMS (ESI+) calcd for $C_{27}H_{21}F_5N_2O_3$ $[M+H]^+$ m/z = 517.1545; found: 517.1536.

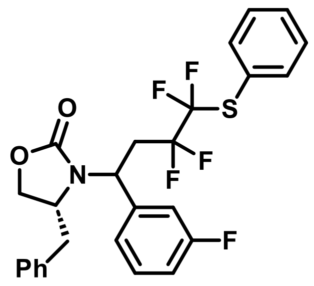

(4*R*)-4-benzyl-3-(3,3,4,4-tetrafluoro-1-(3-fluorophenyl)-4-(phenylthio)butyl)oxazolidin-2-one (6g): Compound **6g** was synthesized following the general procedure 2 (standard-scale), using (2-bromo-1,1,2,2-tetrafluoroethyl)(phenyl)sulfane (81.2 mg, 0.4 mmol), (*R*)-4-benzyl-3-vinyloxazolidin-2-one (40.6 mg, 0.2 mmol) and 3-Fluorophenylmagnesium bromide (1.6 mL, 0.5 M solution in THF, 0.8 mmol). The product **6g** was obtained as a colorless liquid (85.3 mg, 84% yield) after purification by column chromatography on silica gel with hexane/EtOAc (8:2).

^{1}H NMR (400 MHz, CDCl$_3$) δ = 7.66 (d, *J* = 6.9 Hz, 2H), 7.50 – 7.44 (m, 1H), 7.43 – 7.37 (m, 2H), 7.37 – 7.26 (m, 4H), 7.20 – 7.15 (m, 1H), 7.15 - 7.08 (m, 3H), 7.04 (td, *J* = 8.4, 2.1 Hz, 1H), 4.13 – 4.05 (m, 1H), 4.14 – 4.05 (m, 1H), 4.01 – 3.91 (m, 2H), 3.75 – 3.58 (m, 1H), 3.08 (dd, *J* = 13.8, 4.7 Hz, 1H), 2.95 – 2.79 (m, 1H), 2.65 (dd, *J* = 13.7, 8.7 Hz, 1H);

^{13}C NMR (100 MHz, CDCl$_3$) δ = 163.1 (d, *J* = 246.6 Hz), 157.9, 141.4 (d, *J* = 6.7 Hz), 137.3, 135.6, 130.8 (d, *J* = 8.2 Hz), 130.7, 129.4, 129.2, 129.0, 127.5, 123.8, 123.2 (d, *J* = 2.8 Hz), 120.4 – 114.3 (m), 115.7(d, *J* = 20.9 Hz), 114.7 (d, *J* = 22.0 Hz), 67.4, 56.7, 51.5, 38.9, 32.8 (t, *J* = 20.1 Hz);

^{19}F NMR (376 MHz, CDCl$_3$) δ = -88.67 to -88.84 (m, 2F), -110.91 to -113.22 (m, 3F);

IR (film) 3063.37, 3029.14, 3005.71, 2957.29, 2922.15, 1740.87, 1615.42, 1591.79, 1486.55, 1453.62, 1441.91, 1417.69, 1386.01, 1231.99, 1146.91, 1066.09, 1002.76, 923.50, 787.92, 764.41, 741.13, 701.68, 689.96, 646.61, 581.18, 558.65, 521.24, 502.02 cm^{-1};

HRMS (ESI+) calcd for $C_{26}H_{22}F_5NO_2S$ $[M+H]^+$ m/z = 508.1364; found: 508.1355.

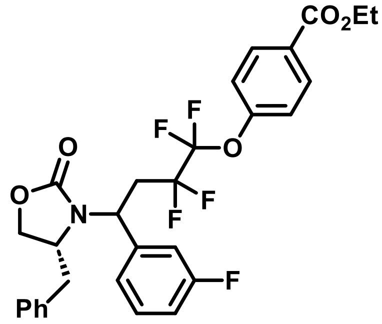

Ethyl 4-(4-((*R*)-4-benzyl-2-oxooxazolidin-3-yl)-1,1,2,2-tetrafluoro-4-(3-fluorophenyl)butoxy)benzoate (6h): Compound **6h** was synthesized following the general procedure 2 (standard-scale), using 4-(2-bromo-1,1,2,2-tetrafluoroethoxy)benzoate (138.0 mg, 0.4 mmol), (*R*)-4-benzyl-3-vinyloxazolidin-2-one (40.6 mg, 0.2 mmol) and 3-Fluorophenylmagnesium bromide (1.6 mL, 0.5 M solution in THF, 0.8 mmol). The product **6h** was obtained as a colorless liquid (99.2 mg, 88% yield) after purification by column chromatography on silica gel with hexane/EtOAc (8:2).

^{1}H NMR (400 MHz, CDCl$_3$) δ = 8.08 (d, *J* = 9.0 Hz, 2H), 7.38 – 7.31 (m, 3H), 7.31 – 7.25 (m, 3H), 7.22 – 7.17 (m, 1H), 7.18 – 7.09 (m, 3H), 7.05 (tdd, *J* = 8.4, 2.6, 1.1 Hz, 1H), 4.85 (dd, *J* = 8.4, 5.4 Hz, 1H), 4.38 (q, *J* = 7.1 Hz, 2H), 4.15 – 4.09 (m, 1H), 4.04 – 3.92 (m, 2H), 3.82 – 3.67 (m, 1H), 3.13 (dd, *J* = 13.8, 4.8 Hz, 1H), 3.02 – 2.86 (m, 1H), 2.70 (dd, *J* = 13.8, 8.6 Hz, 1H), 1.40 (t, *J* = 7.1 Hz, 3H);

^{13}C NMR (100 MHz, CDCl$_3$) δ = 165.7, 163.1 (d, *J* = 246.6 Hz), 158.0, 152.6, 141.3 (d, *J* = 6.7 Hz), 135.6, 131.5, 130.8 (d, *J* = 8.2 Hz), 129.2, 129.0, 128.8, 127.5, 123.3 (d, *J* = 2.8 Hz), 121.2, 120.4 – 114.3 (m), 115.8 (d, *J* = 20.9 Hz), 114.8 (d, *J* = 22.0 Hz), 67.5, 61.4, 56.9, 51.5, 39.0, 32.9 (t, *J* = 20.1 Hz), 14.4;

^{19}F NMR (376 MHz, CDCl$_3$) δ = -88.35 to -88.44 (m, 2F), -111.17 to -111.29 (m, 1F), -116.56 to -118.75 (m, 2F);

IR (film) 3066.30, 3029.15, 2983.18, 2936.62, 2908.45, 1744.92, 1717.08, 1605.72, 1592.24, 1504.76, 1488.29, 1453.85, 1415.24, 1390.86, 1367.35, 1275.82, 1241.28, 1166.76, 1105.39, 1044.62, 1018.14, 956.69, 875.22, 857.75, 789.08, 763.72, 739.54, 723.42, 701.98, 585.33, 534.56, 521.15, 504.17, 450.90 cm^{-1};
HRMS (APCI+) calcd for $C_{29}H_{26}F_5NO_5$ $[M+H]^+$ m/z = 564.1804; found: 564.1792.

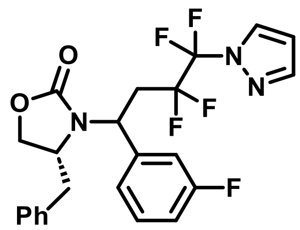

(4*R*)-4-benzyl-3-(3,3,4,4-tetrafluoro-1-(3-fluorophenyl)-4-(1H-pyrazol-1-yl)butyl)oxazolidin-2-one (6i): Compound **6i** was synthesized following the general procedure 2 (standard-scale), using 1-(2-bromo-1,1,2,2-tetrafluoroethyl)-1H-pyrazole (98.8 mg, 0.4 mmol), (*R*)-4-benzyl-3-vinyloxazolidin-2-one (40.6 mg, 0.2 mmol) and 3-Fluorophenylmagnesium bromide (1.6 mL, 0.5 M solution in THF, 0.8 mmol). The product **6i** was obtained as a colorless liquid (70.8 mg, 76% yield) after purification by column chromatography on silica gel with hexane/EtOAc (8:2).
^{1}H NMR (400 MHz, CDCl$_3$) δ = 7.82 (s, 1H), 7.77 (s, 1H), 7.36 – 7.24 (m, 4H), 7.20 – 7.14 (m, 1H), 7.14 – 7.06 (m, 3H), 7.03 (td, *J* = 8.4, 3.2 Hz, 1H), 6.47 (s, 1H), 4.92 (dd, *J* = 9.1, 5.0 Hz, 1H), 4.15 – 4.01 (m, 1H), 4.00 – 3.89 (m, 2H), 3.81 – 3.62 (m, 1H), 3.09 – 2.91 (m, 2H), 2.58 (dd, *J* = 13.7, 8.9 Hz, 1H);
^{13}C NMR (100 MHz, CDCl$_3$) δ = 163.1 (d, *J* = 246.6 Hz), 157.9, 143.3, 141.3 (d, *J* = 6.7 Hz), 135.6, 130.7 (d, *J* = 8.2 Hz), 129.3, 129.1, 129.0, 127.5, 123.2 (d, *J* = 2.8 Hz), 120.4 – 114.3 (m), 115.7 (d, *J* = 20.9 Hz), 114.7 (d, *J* = 22.0 Hz), 108.3, 67.4, 56.6, 51.2, 39.0, 32.5 (t, *J* = 20.1 Hz);
^{19}F NMR (376 MHz, CDCl$_3$) δ = -97.47 to -99.01 (m, 2F), -111.21 to -111.42 (m, 1F), -113.59 to -116.21 (m, 2F);
IR (film) 3134.59, 3064.17, 3029.82, 3005.45, 2917.83, 1742.42, 1615.39, 1592.03, 1525.38, 1488.08, 1453.84, 1422.89, 1392.94, 1343.05, 1237.90, 1163.64, 1114.11, 1056.23, 1042.66, 977.25, 917.12, 898.77, 762.02, 735.29, 700.56, 641.15, 616.30, 582.08, 537.54, 521.23, 503.40, 452.25, 422.78 cm^{-1};
HRMS (APCI+) calcd for $C_{23}H_{20}F_5N_3O_2$ $[M+H]^+$ m/z = 566.1548; found: 566.1541.

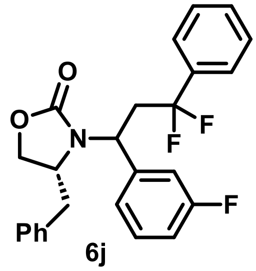

6j

(4*R*)-4-benzyl-3-(3,3-difluoro-1-(3-fluorophenyl)-3-phenylpropyl)oxazolidin-2-one (6j): Compound **6j** was synthesized following the general procedure 2 (standard-scale), using (bromodifluoromethyl)benzene (82.8 mg, 0.4 mmol), (*R*)-4-benzyl-3-vinyloxazolidin-2-one (40.6 mg, 0.2 mmol) and 3-Fluorophenylmagnesium bromide (1.6 mL, 0.5 M solution in THF, 0.8 mmol). The product **6j** was obtained as a colorless liquid (34.9 mg, 41% yield) after purification by column chromatography on silica gel with hexane/EtOAc (8:2).
^{1}H NMR (400 MHz, CDCl$_3$) δ = 7.54 – 7.49 (m, 2H), 7.45 – 7.40 (m, 3H), 7.34 – 7.23 (m, 4H), 7.18 – 7.12 (m, 1H), 7.13 – 7.05 (m, 3H), 7.00 (td, *J* = 8.3, 3.3 Hz, 1H), 4.89 (dd, *J* = 8.8, 4.7 Hz, 1H), 3.96 – 3.84 (m, 3H), 3.72 – 3.56 (m, 1H), 2.99 – 2.83 (m, 2H), 2.48 (dd, *J* = 13.8, 8.7 Hz, 1H);
^{13}C NMR (100 MHz, CDCl$_3$) δ = 163.1 (d, *J* = 246.6 Hz), 157.9, 152.5, 142.3 (d, *J* = 6.7 Hz), 136.7 (t, *J* = 25.9 Hz) , 135.7, 130.6 (d, *J* = 8.2 Hz), 130.3, 129.1, 129.0, 128.7, 127.4, 125.1 (t, *J* = 5.9 Hz), 123.2 (d, *J* = 2.8 Hz), 122.1 (t, *J*= 241.1 Hz), 115.2 (d, *J* = 20.9 Hz), 114.7 (d, *J* = 22.0 Hz), 67.2, 56.5, 52.3, 40.4 (t, *J* = 26.9 Hz) , 38.81;
^{19}F NMR (376 MHz, CDCl$_3$) δ = -97.67 to -96.43 (m, 2F), -111.49 to -111.75 (m, 1F);

IR (film) 3134.59, 3064.17, 3029.82, 3005.45, 2917.83, 1742.42, 1615.39, 1592.03, 1525.38, 1488.08, 1453.84, 1422.89, 1392.94, 1343.05, 1237.90, 1163.64, 1114.11, 1056.23, 1042.66, 977.25, 917.12, 898.77, 762.02, 735.29, 700.56, 641.15, 616.30, 582.08, 537.54, 521.23, 503.40, 452.25, 422.78 cm^{-1};
HRMS (ESI+) calcd for $C_{25}H_{22}F_3NO_2$ $[M+H]^+$ m/z = 426.1675; found: 426.1664.

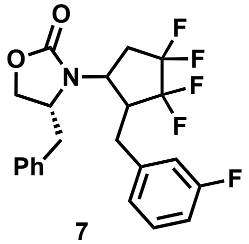

7

(4*R*)-4-benzyl-3-(3,3,4,4-tetrafluoro-2-(3-fluorobenzyl)cyclopentyl)oxazolidin-2-one (7): Compound **7** was synthesized following the general procedure 2 (standard-scale), using 4-bromo-3,3,4,4-tetrafluorobut-1-ene (82.4 mg, 0.4 mmol), (*R*)-4-benzyl-3-vinyloxazolidin-2-one (40.6 mg, 0.2 mmol) and 3-Fluorophenylmagnesium bromide (1.6 mL, 0.5 M solution in THF, 0.8 mmol). The product **7** was obtained as a colorless liquid (46.5 mg, 55% yield) after purification by column chromatography on silica gel with hexane/EtOAc (8:2).
^{1}H NMR (400 MHz, $CDCl_3$) δ = 7.39 – 7.29 (m, 4H), 7.09 (t, *J* = 8.8 Hz, 3H), 6.99 (t, *J* = 8.2 Hz, 2H), 4.29 (q, *J* = 8.9 Hz, 1H), 4.11 (t, *J* = 7.9 Hz, 1H), 4.06 – 3.93 (m, 2H), 3.29 – 3.13 (m, 1H), 3.11 – 2.92 (m, 4H), 2.58 (td, *J* = 15.1, 8.6 Hz, 1H), 2.23 (dd, *J* = 13.3, 9.8 Hz, 1H).
^{13}C NMR (100 MHz, $CDCl_3$) δ = 164.21, 161.76, 157.69, 140.63, 140.56, 134.67, 130.25, 130.17, 129.19, 128.84, 127.62, 124.53, 124.50, 115.90, 115.68, 113.90, 113.69, 67.02, 58.21, 48.40, 47.03, 46.83, 46.63, 39.21, 32.96, 28.65.
^{19}F NMR (376 MHz, $CDCl_3$) δ = -112.81 (td, *J* = 9.4, 6.1 Hz), -120.84 – -122.98 (m).
IR (film) 3030.33, 1746.51, 1615.88, 1589.91, 1490.28, 1454.91, 1394.77, 1354.96, 1252.75, 1184.60, 1140.93, 1095.12, 1036.02, 977.71, 769.68, 737.20, 702.01, 689.48
HRMS (APCI+) calcd for $C_{22}H_{21}F_5NO_2$ $[M+H]^+$ m/z = 426.1492; found: 426.1483

9. References

[1] Nguyen, T. B.; Martel, A.; Dhal, R.; Dujardin, G. 1,3-Dipolar Cycloaddition of *N*-Substituted Dipolarophiles and Nitrones: Highly Efficient Solvent Free Reaction. *J. Org. Chem.* **2008**, *73*, 2621-2632.
[2] (a) Gaulon, C.;c) Dhal, R.; Dujardin, G. Preparation of chiral N-vinyl oxazolidinones by a simple general procedure. *Synthesis*, **2003**, *14*, 2269-2272; (b) Feltenberger, J. B.; Hayashi, R.; Tang, Y.; Babiash, E. S.; Hsung, R. P. Enamide-Benzyne- [2+ 2] Cycloaddition: Stereoselective Tandem [2+ 2] − Pericyclic Ring-Opening− Intramolecular N-Tethered [4+ 2] Cycloadditions. *Org. lett.* **2009**, *11*, 3666-3669; (c) Brice, J. L.; Meerdink, J. E.; Stahl, S. S. Formation of enamides via palladium (II)-catalyzed vinyl transfer from vinyl ethers to nitrogen nucleophiles. *Org. lett.* **2004**, *6*,1845-1848.
[3] (a) Lee, C.; Yang, W.; Parr, R. G. Development of the Colle-Salvetti Correlation-Energy Formula into a Functional of the Electron Density. *Phys. Rev. B* **1988**, *37*, 785–789. (b) Becke, A. D. Density Functional Thermochemistry. III. The Role of Exact Exchange. *J. Chem. Phys.* **1993**, *98*, 5648–5652.
[4] (a) Grimme, S. Accurate description of van der Waals complexes by density functional theory including empirical corrections. *J. Comput. Chem.* **2004**, *25*, 1463–1473. (b) Grimme, S.; Antony, J.; Ehrlich, S.; Krieg, H. A consistent and accurate ab initio parametrization of density functional dispersion correction (DFT-D) for the 94 elements H-Pu. *J. Chem. Phys.* **2010**, *132*, 154104. (c) Grimme, S. Density functional theory with London dispersion corrections. *WIRES Comput. Mol. Sci.* **2011**, *1*, 211-228. (d) Ehrlich, S.; Moellmann, J.; Grimme, S. Dispersion-Corrected Density Functional Theory for Aromatic Interactions in Complex Systems. *Acc. Chem. Res.* **2012**, *46*, 916–926.
[5] Gaussian 16, Revision C.01, Frisch, M. J.; Trucks, G. W.; Schlegel, H. B.; Scuseria, G. E.; Robb, M. A.; Cheeseman, J. R.; Scalmani, G.; Barone, V.; Petersson, G. A.; Nakatsuji, H.; Li, X.; Caricato, M.; Marenich, A. V.; Bloino, J.; Janesko, B. G.; Gomperts, R.; Mennucci, B.; Hratchian, H. P.; Ortiz, J. V.; Izmaylov, A. F.; Sonnenberg, J. L.; Williams-Young, D.; Ding, F.; Lipparini, F.; Egidi, F.; Goings, J.; Peng, B.; Petrone, A.; Henderson, T.; Ranasinghe, D.; Zakrzewski, V. G.; Gao, J.; Rega, N.; Zheng, G.; Liang, W.; Hada, M.; Ehara, M.; Toyota, K.; Fukuda, R.; Hasegawa, J.; Ishida, M.; Nakajima, T.; Honda, Y.; Kitao, O.; Nakai, H.; Vreven, T.; Throssell, K.; Montgomery, J. A., Jr.; Peralta, J. E.; Ogliaro, F.; Bearpark, M. J.; Heyd, J. J.; Brothers, E. N.; Kudin, K. N.; Staroverov, V. N.; Keith, T. A.; Kobayashi, R.; Normand, J.; Raghavachari, K.; Rendell, A. P.; Burant, J. C.; Iyengar, S. S.; Tomasi, J.; Cossi, M.; Millam, J. M.; Klene, M.; Adamo, C.; Cammi, R.; Ochterski, J. W.; Martin, R. L.; Morokuma, K.; Farkas, O.; Foresman, J. B.; Fox, D. J. Gaussian, Inc., Wallingford CT, 2016.
[6] (a) Liu, L.; Aguilera, M. C.; Lee, W.; Youshaw, C. R.; Neidig, M. L.; Gutierrez, O. General Method for Iron-catalyzed Multicomponent Radical Cascades Cross-couplings. *Science.* **2021**, *374*, 432−439. (b) Youshaw, C. R.; Yang, M. H.; Gogoi, A. R.; Rentería-Gómez, A.; Liu, L.; Morehead, L. M.; Gutierrez, O. Iron-Catalyzed Enantioselective Multicomponent Cross-Couplings of α-Boryl Radicals. *Org. Lett.* **2023**, *25*, 8320-8325. (c) Lee, W.; Zhou, J.; Gutierrez, O. Mechanism of Nakamura's

Iron-Catalyzed Asymmetric Cross-coupling Reaction: The Role of Spin in Controlling Selectivity. *J. Am. Chem. Soc.* **2017**, *139*, 16126−16133. (d) Aguilera, M. C.; Gogoi, A. R.; Lee, W.; Liu, L.; Brennessel, W.; Gutierrez, O.; Neidig, M. L. Insight into Radical Initiation, Solvent Effects and Biphenyl Production in Iron-Bisphosphine Cross-Couplings. *ACS Catal.* **2023**, *13*, 8987−8996. (e) Renteria-Gomez, A.; Lee, W.; Yin, S.; Davis, M.; Gogoi, A. R; Gutierrez, O. General and Practical Route to Diverse 1-(Difluoro)alkyl-3-aryl Bicyclo[1.1.1]pentanes Enabled by an Fe-Catalyzed Multicomponent Radical Cross-Coupling Reaction. *ACS Catal.* **2022**, *12*, 11547-11556.
[7] Marenich, A. V.; Cramer, C. J.; Truhlar, D. G. Universal Solvation Model Based on Solute Electron Density and on a Continuum Model of the Solvent Defined by the Bulk Dielectric Constant and Atomic Surface Tensions. *J. Phys. Chem. B* **2009**, *113*, 6378–6396.
[8] Y. Zhao and D. G. Truhlar, "A new local density functional for main-group thermochemistry, transition metal bonding, thermochemical kinetics, and noncovalent interactions," *J. Chem. Phys.*, **125** (2006), 194101: 1-18.
[9] (a) Weigend, F.; Ahlrichs, R. Balanced basis sets of split valence, triple zeta valence and quadruple zeta valence quality for H to Rn: Design and assessment of accuracy. *Phys. Chem. Chem. Phys.* **2005**, *7*, 3297-3305. (b) Weigend, F. Accurate Coulomb-fitting basis sets for H to Rn. *Phys. Chem. Chem. Phys.* **2006**, *8*, 1057-1065.
[10] Legault, C. Y. (2009) CYLview, 1.0b, Universite de Sherbrooke: Sherbrooke, Canada, http://www.cylview.org.
[11] Lu, T.; Chen, F., Multiwfn: A multifunctional wavefunction analyzer. *J. Comp. Chem.* **2012**, *33*, 580-592.
[12] Humphrey, W.; Dalke, A.; Schulten, K., VMD – Visual Molecular Dynamics. *J. Mol. Graphics* **1996**, *14*, 33-38.

10. Spectral Data

Compound (*S*)-1c. Top: ^{1}H NMR ($CDCl_3$, 400 MHz). Bottom: ^{13}C NMR ($CDCl_3$, 100 MHz).

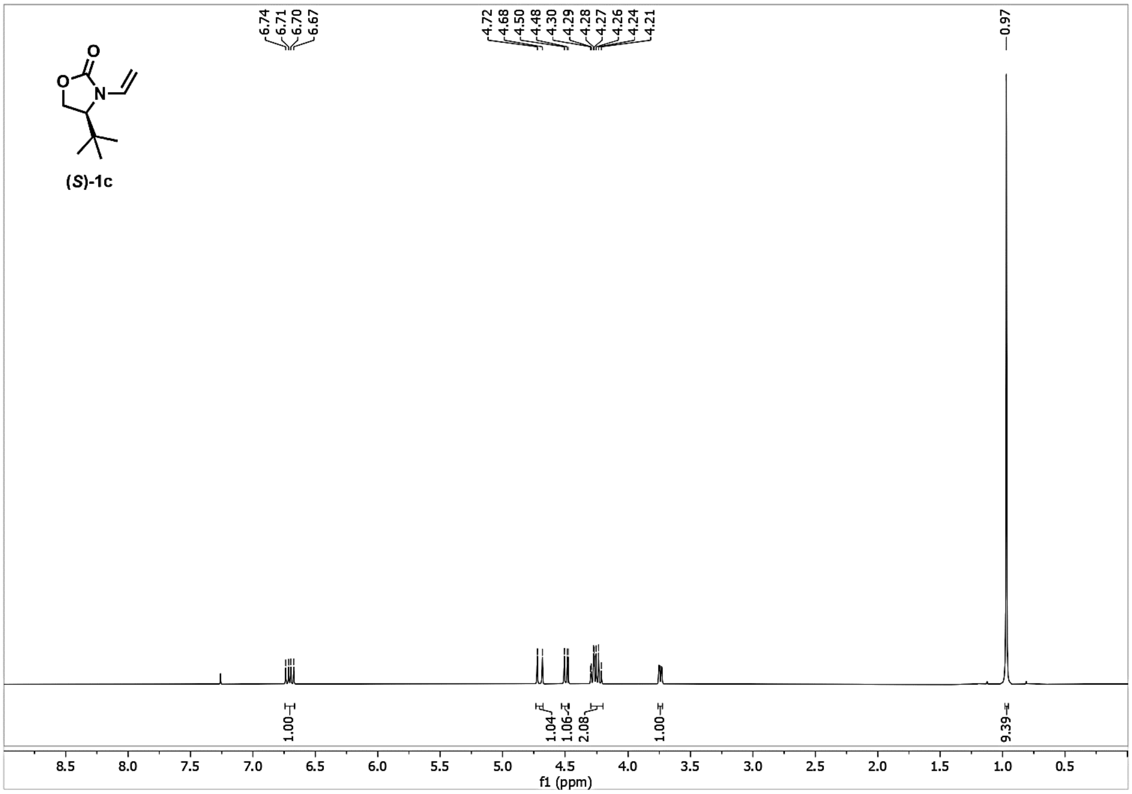

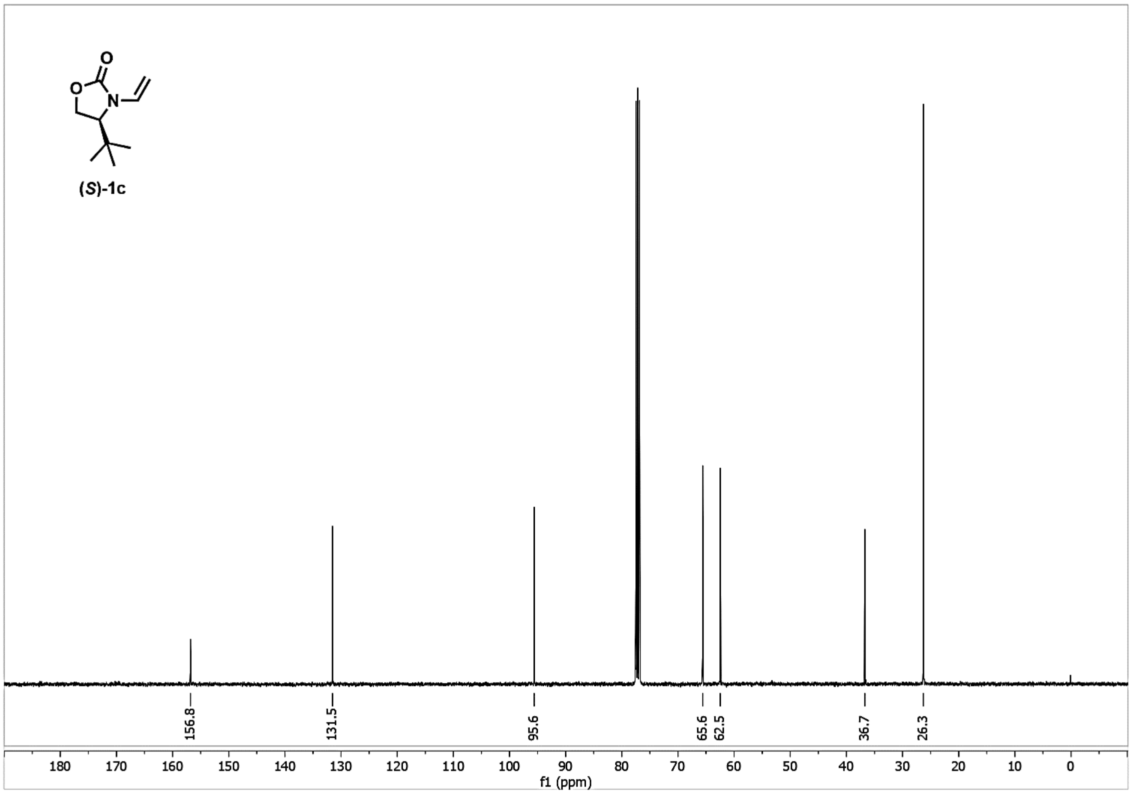

Compound (*S*)-4a. Top: ^{1}H NMR ($CDCl_3$, 400 MHz). Bottom: ^{13}C NMR ($CDCl_3$, 100 MHz). Integrations shown for the major diastereoisomer.

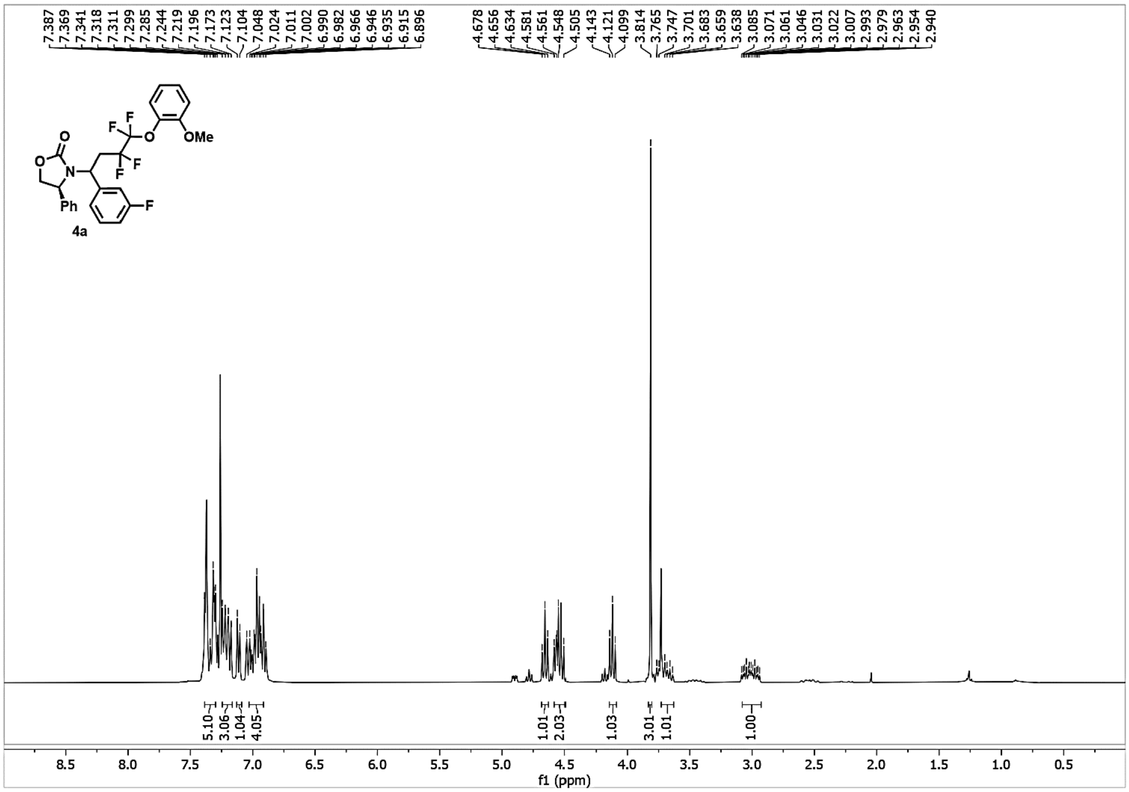

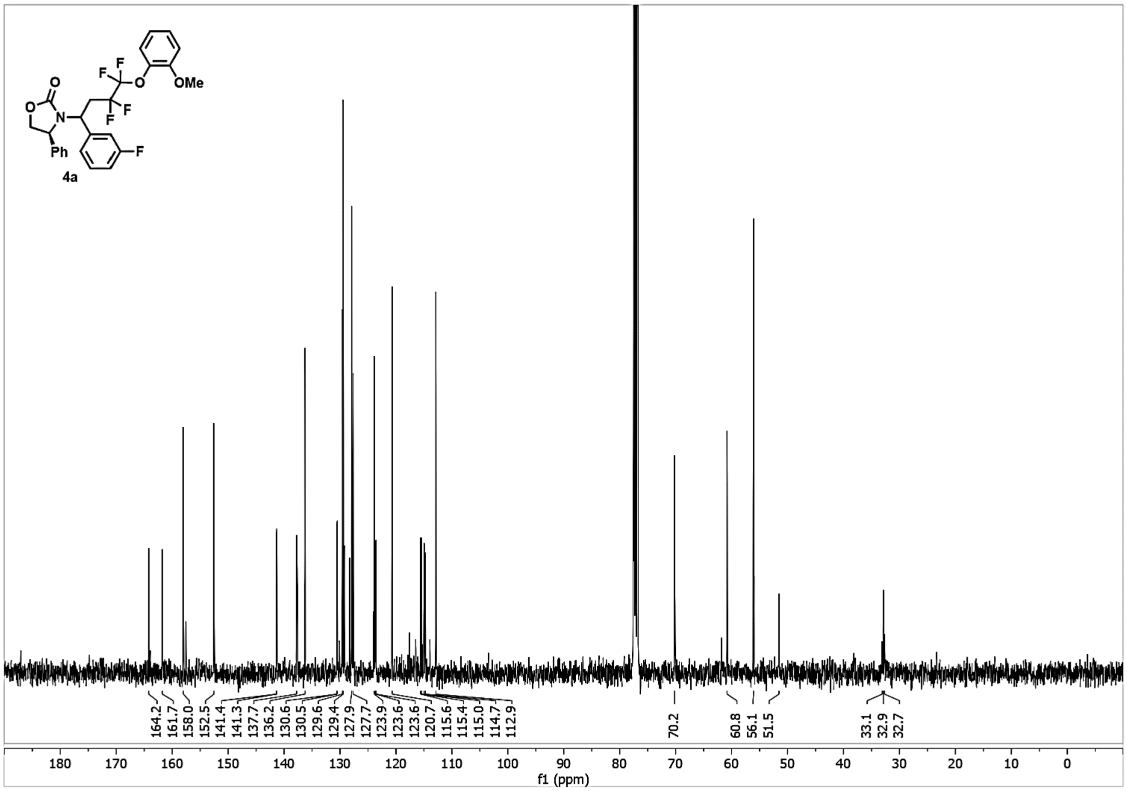

Compound (*S*)-4a. ^{19}F NMR ($CDCl_3$, 376 MHz).

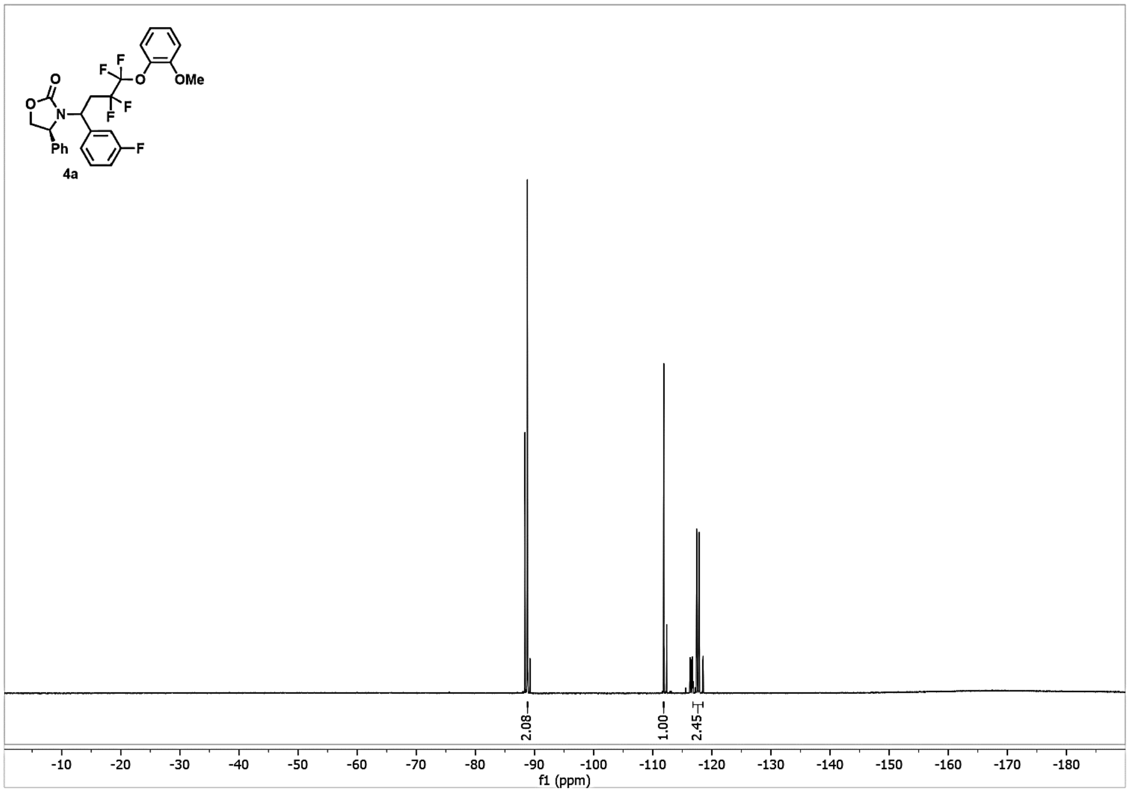

Compound (*S*)-4b. Top: ^1H NMR ($CDCl_3$, 400 MHz). Bottom: ^{13}C NMR ($CDCl_3$, 100 MHz). Integrations shown for the major diastereoisomer.

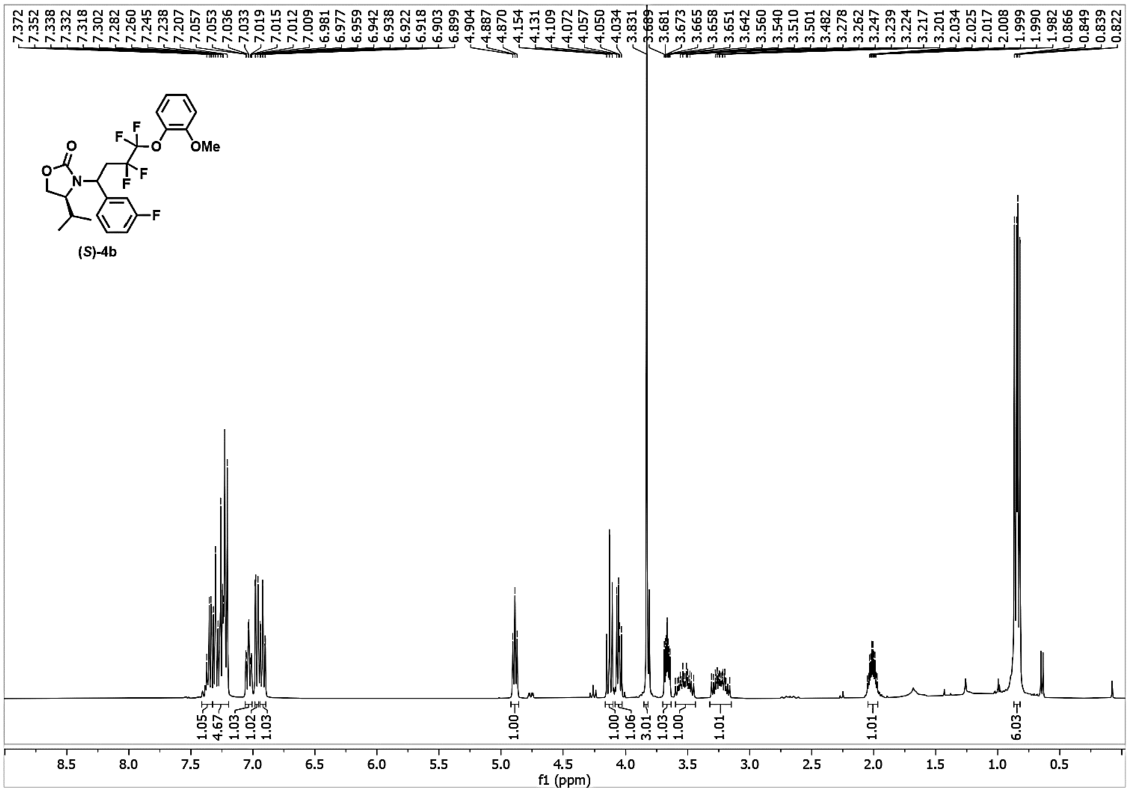

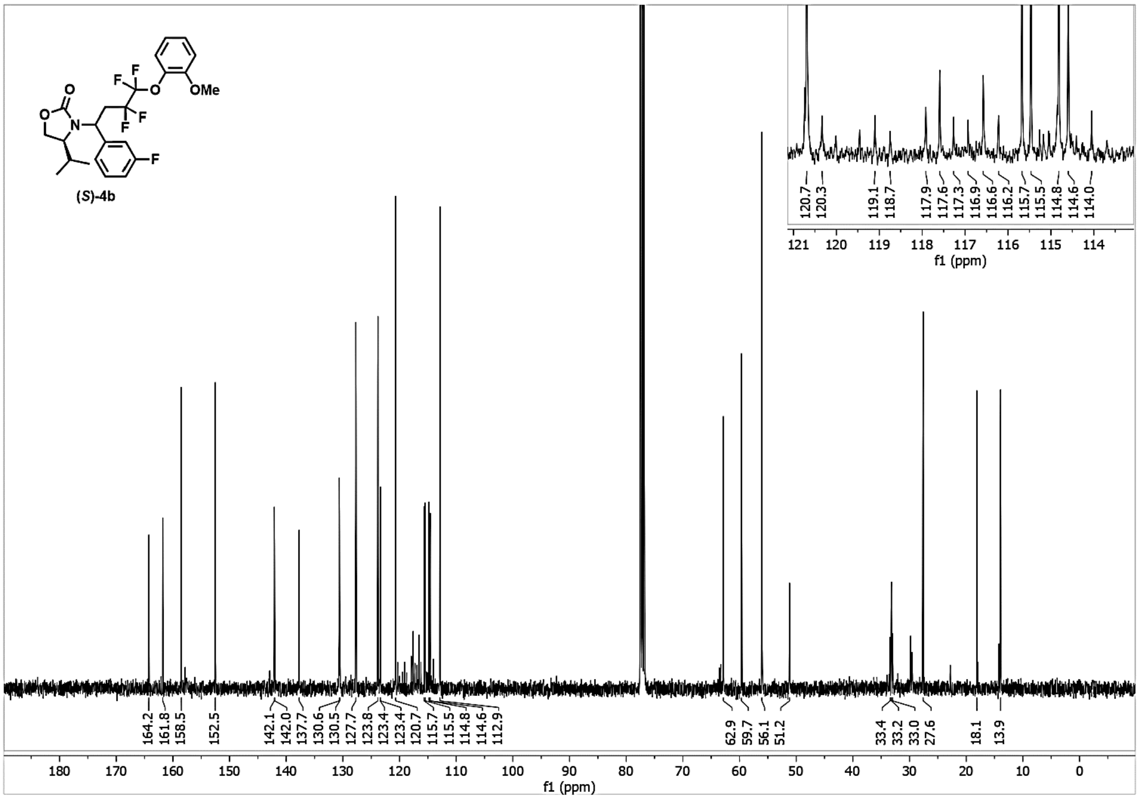

Compound (*S*)-4b. ^{19}F NMR ($CDCl_3$, 376 MHz).

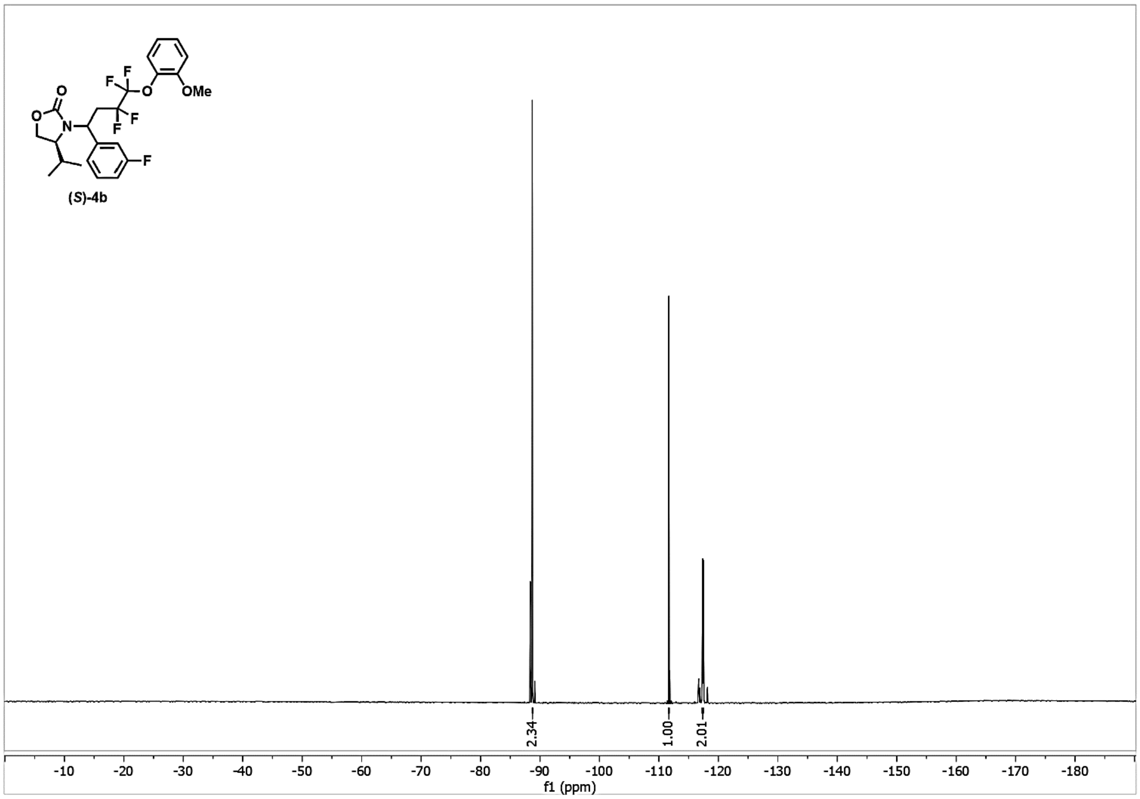

Compound (*S*)-4c. Top: ^{1}H NMR ($CDCl_3$, 400 MHz). Bottom: ^{13}C NMR ($CDCl_3$, 100 MHz).

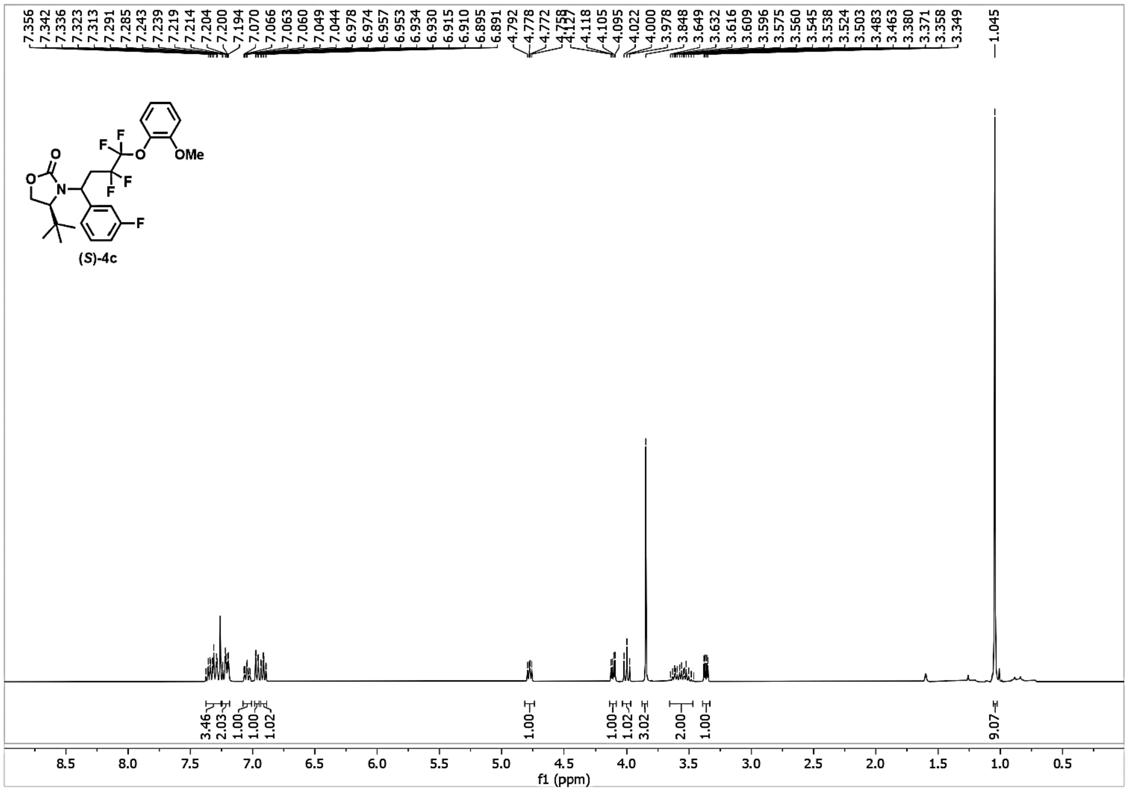

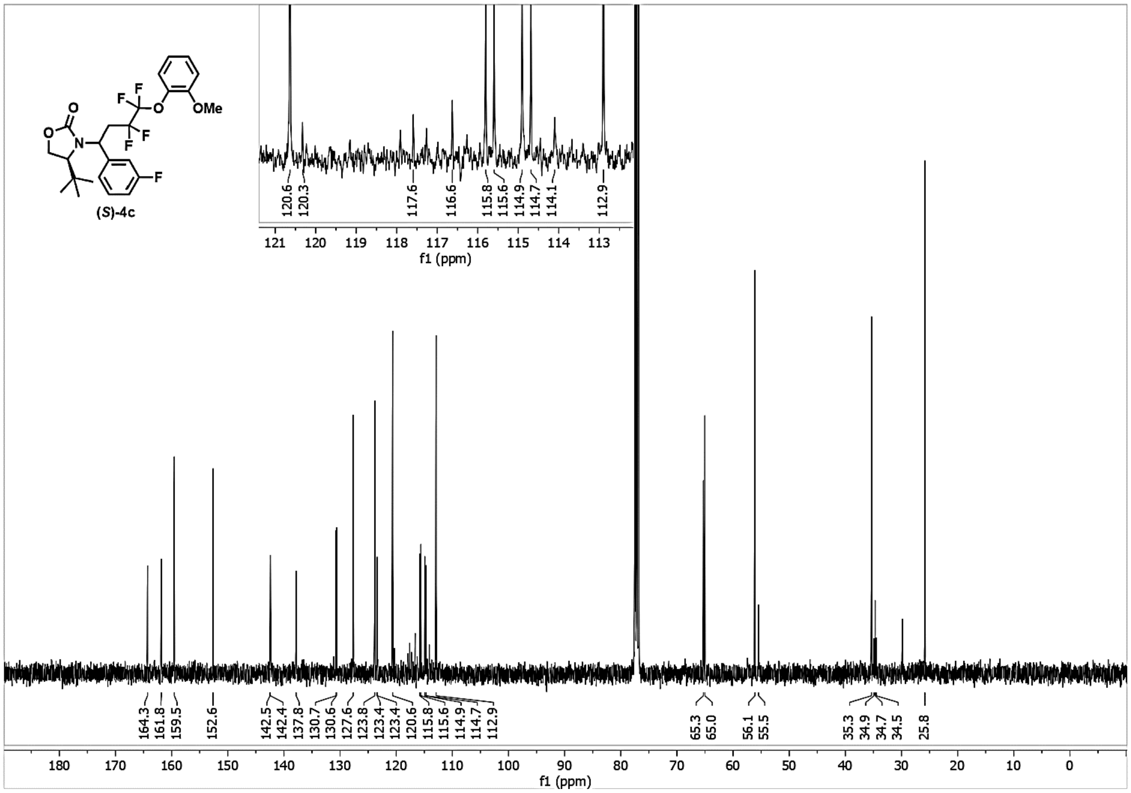

Compound (*S*)-4c. ^{19}F NMR ($CDCl_3$, 376 MHz).

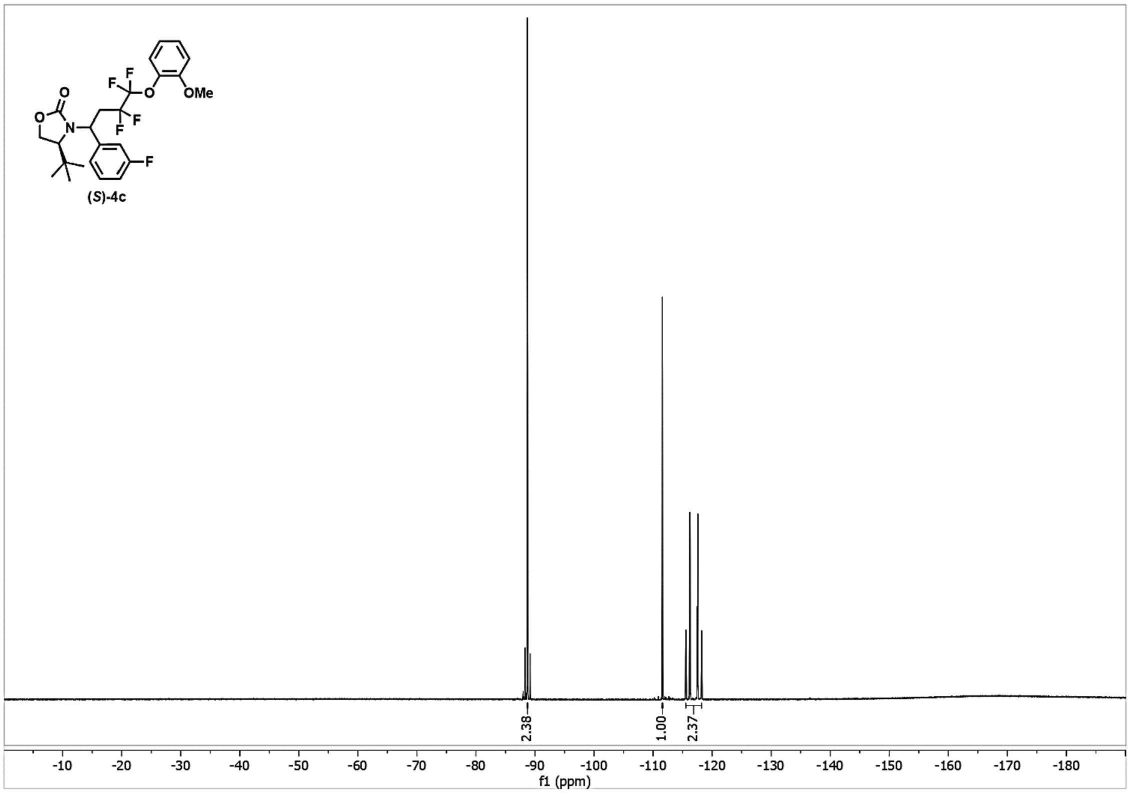

Compound (*S*)-4d. Top: ^{1}H NMR ($CDCl_3$, 400 MHz). Bottom: ^{13}C NMR ($CDCl_3$, 100 MHz).

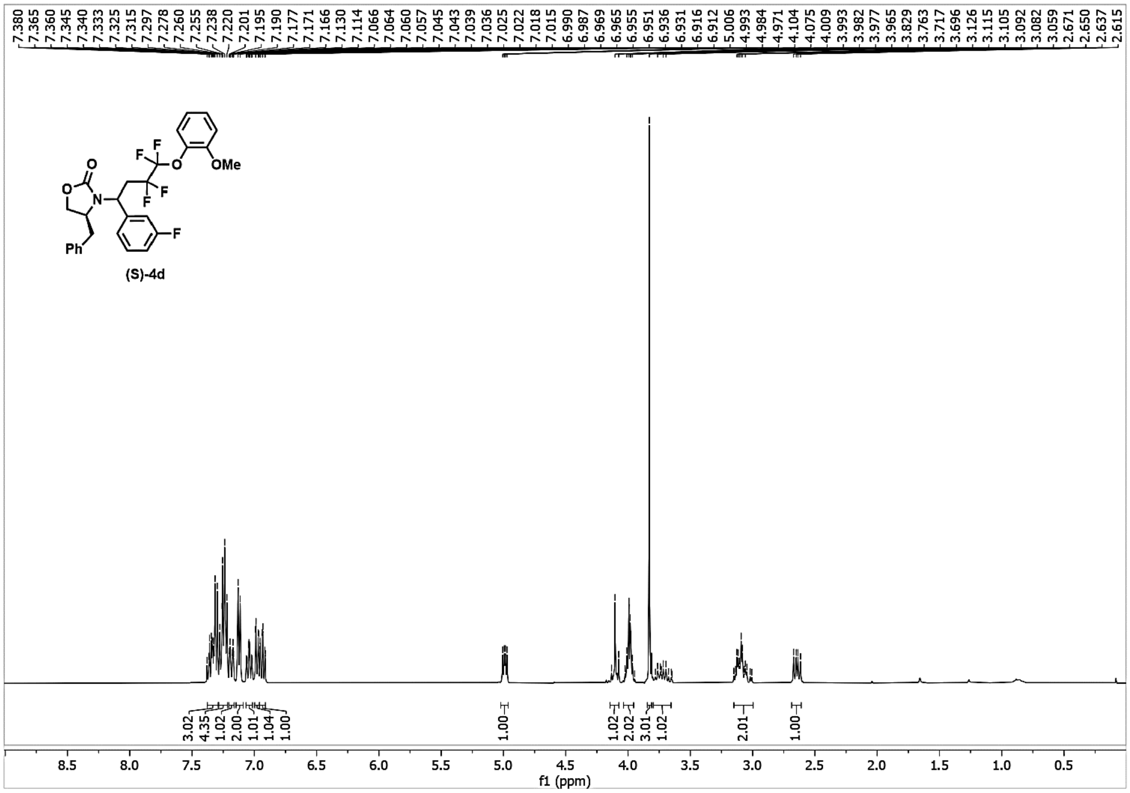

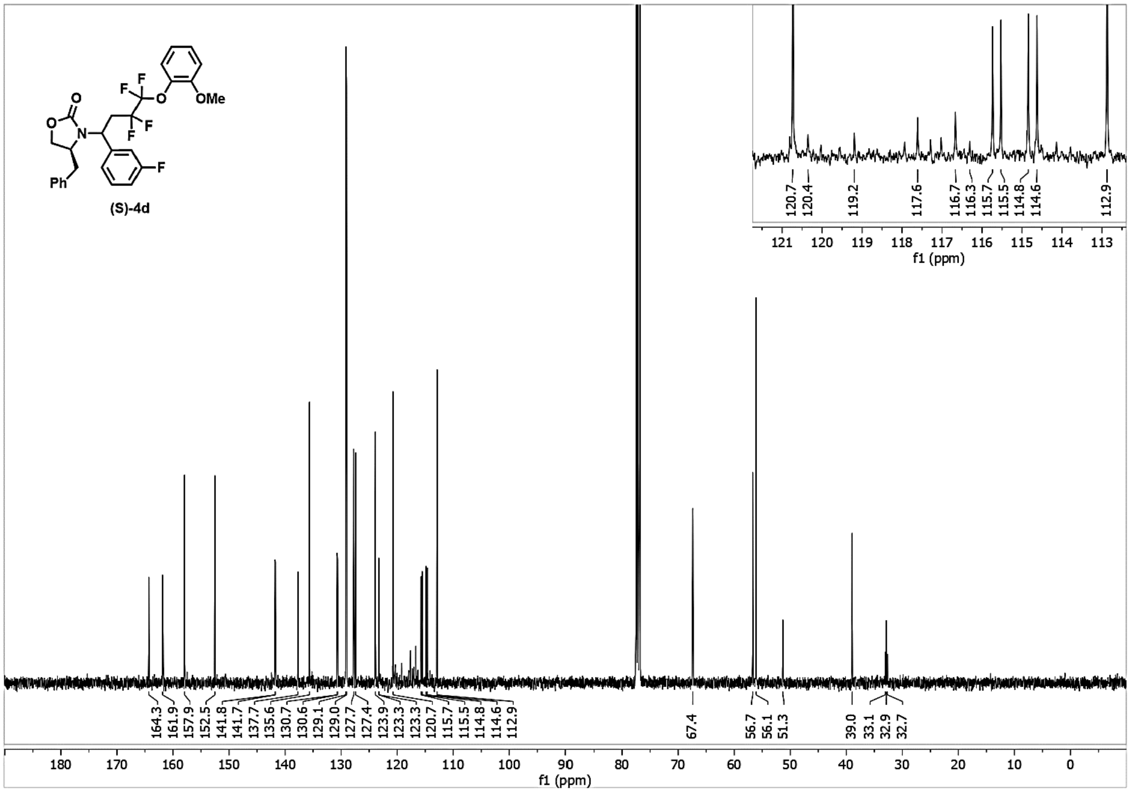

Compound (*S*)-4d. ^{19}F NMR ($CDCl_3$, 376 MHz).

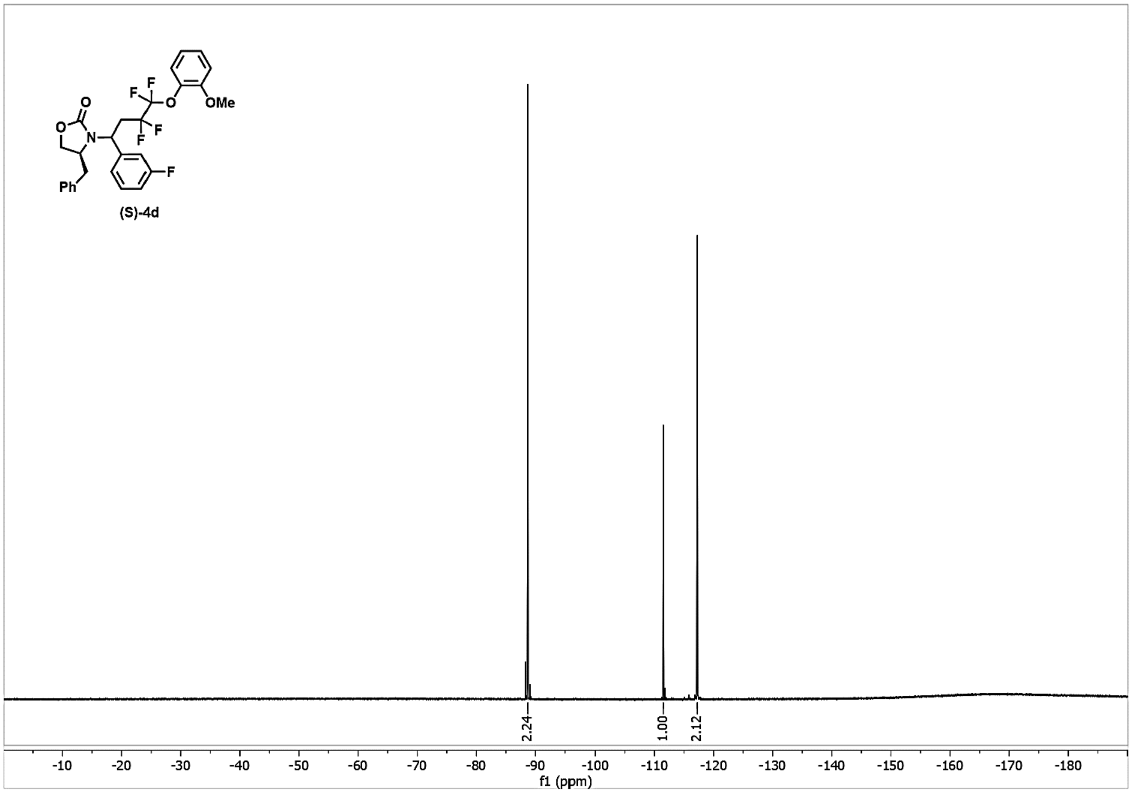

Compound (*R*)-4d. Top: ^{1}H NMR ($CDCl_3$, 400 MHz). Bottom: ^{13}C NMR ($CDCl_3$, 100 MHz).

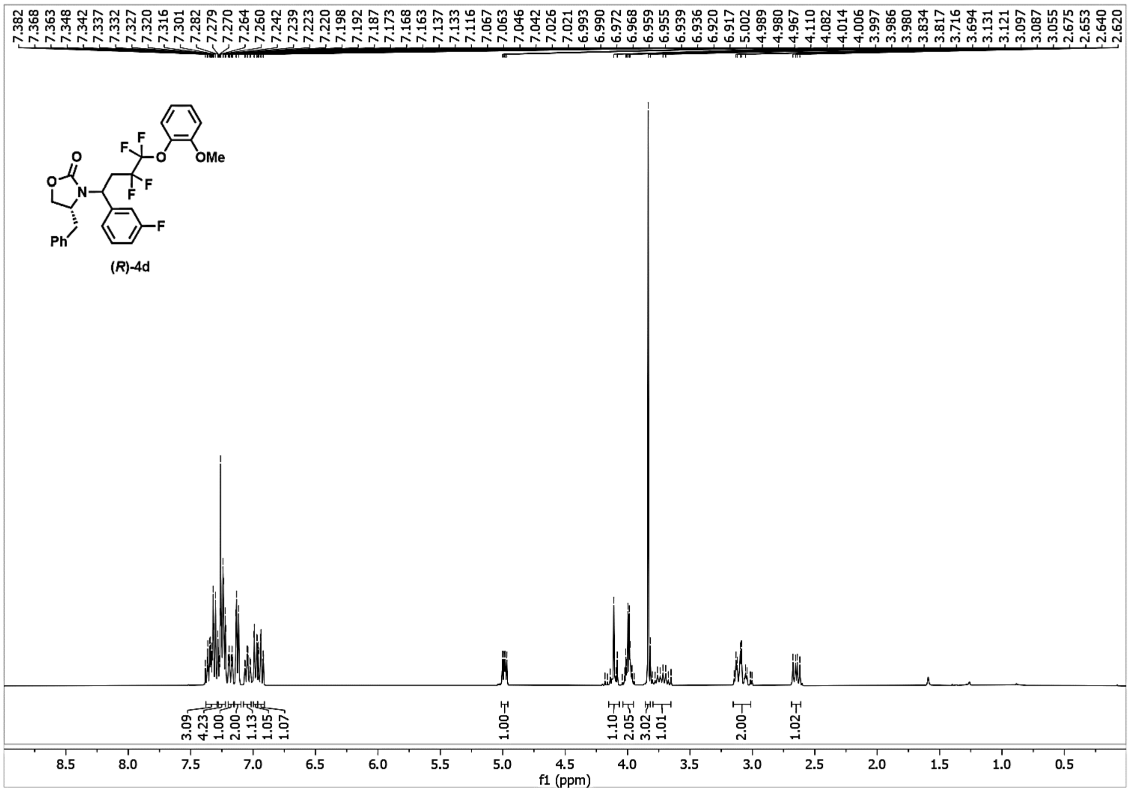

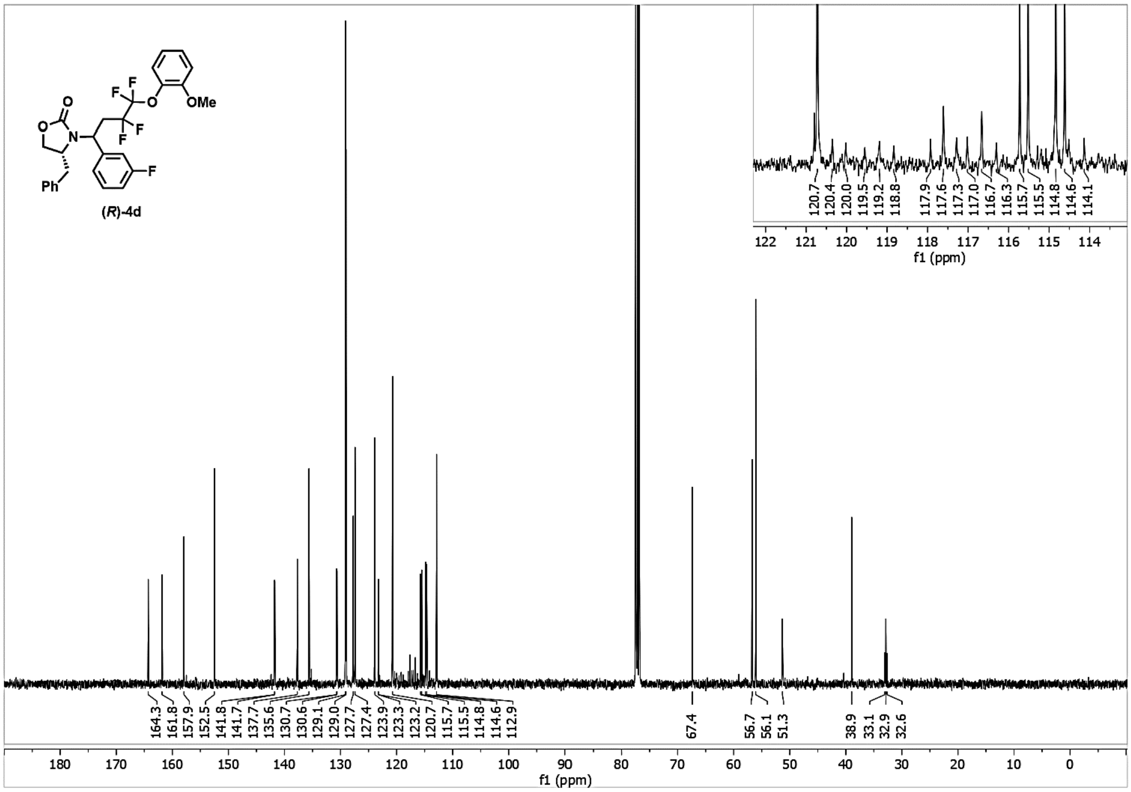

Compound (*R*)-4d. ^{19}F NMR ($CDCl_3$, 376 MHz).

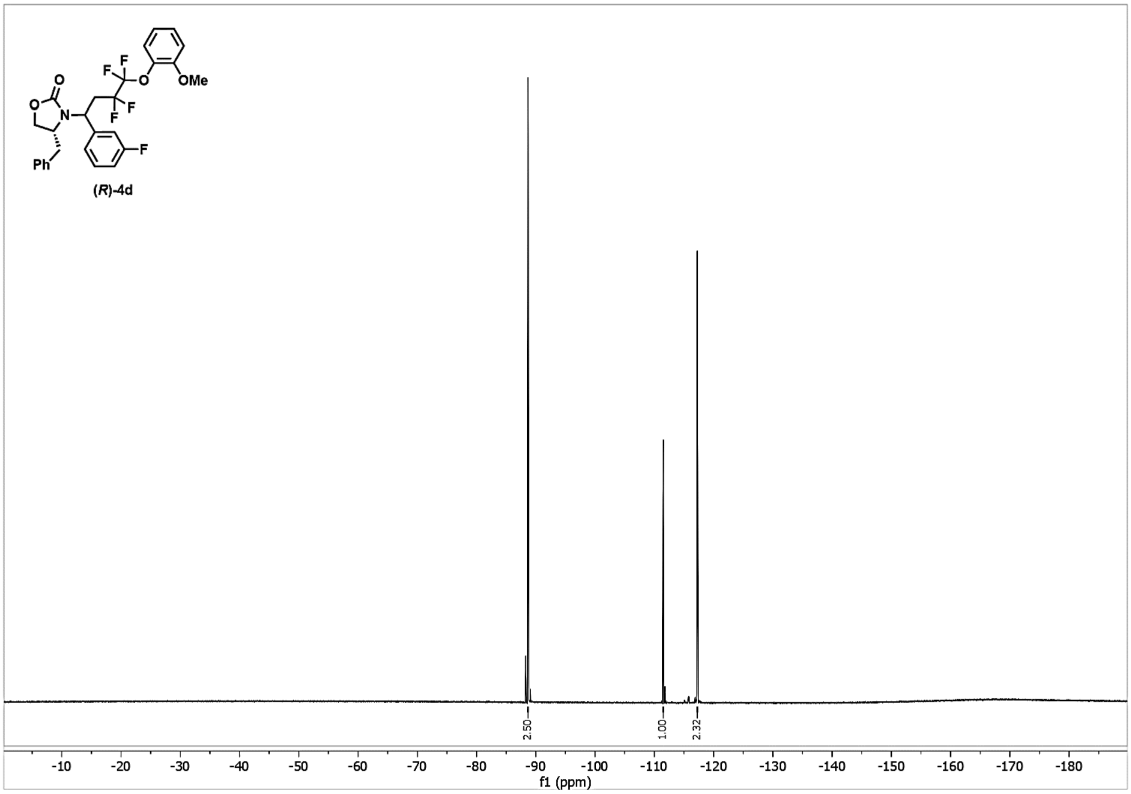

Compound 5a. Top: ^{1}H NMR ($CDCl_3$, 400 MHz). Bottom: ^{13}C NMR ($CDCl_3$, 100 MHz). Integrations shown for the major diastereoisomer.

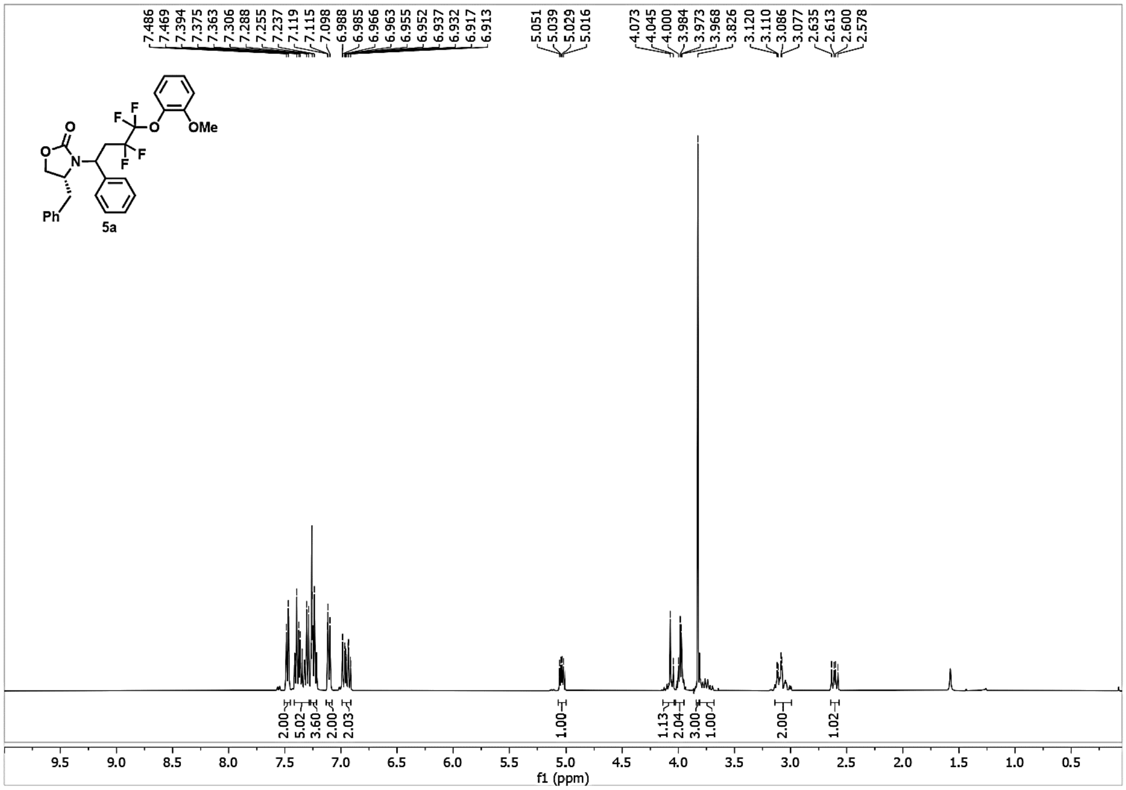

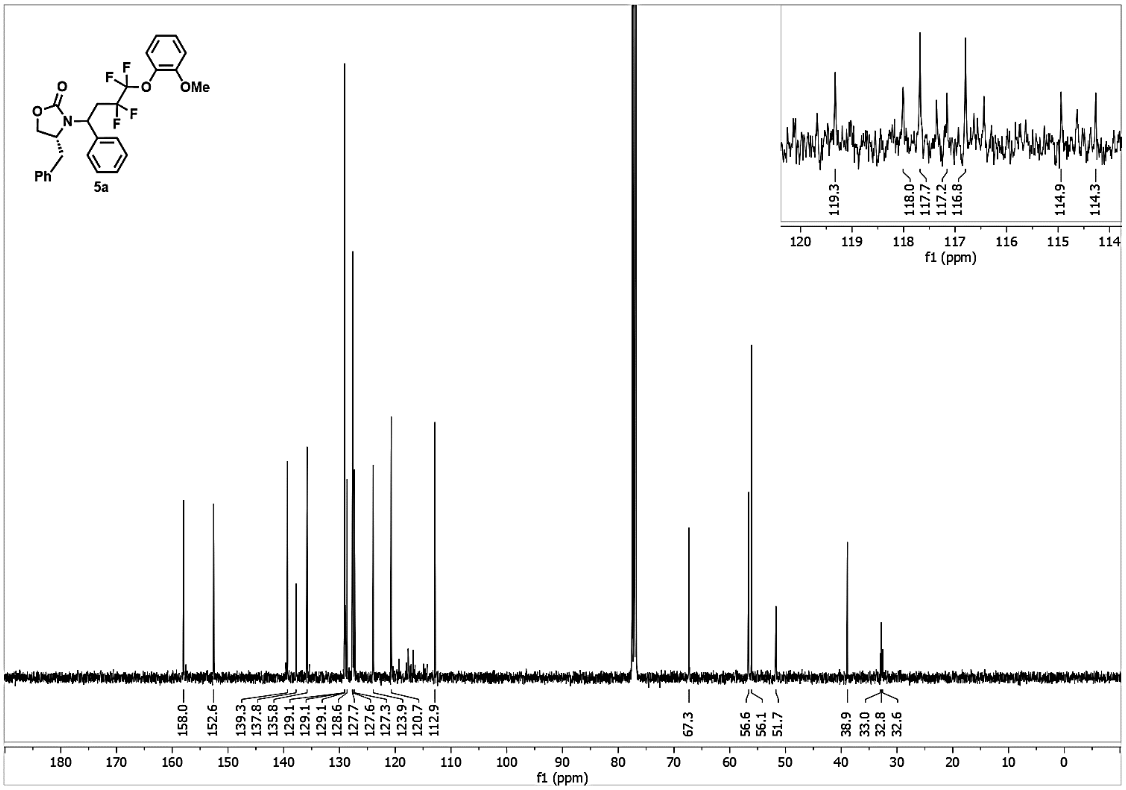

Compound 5a. ^{19}F NMR ($CDCl_3$, 376 MHz).

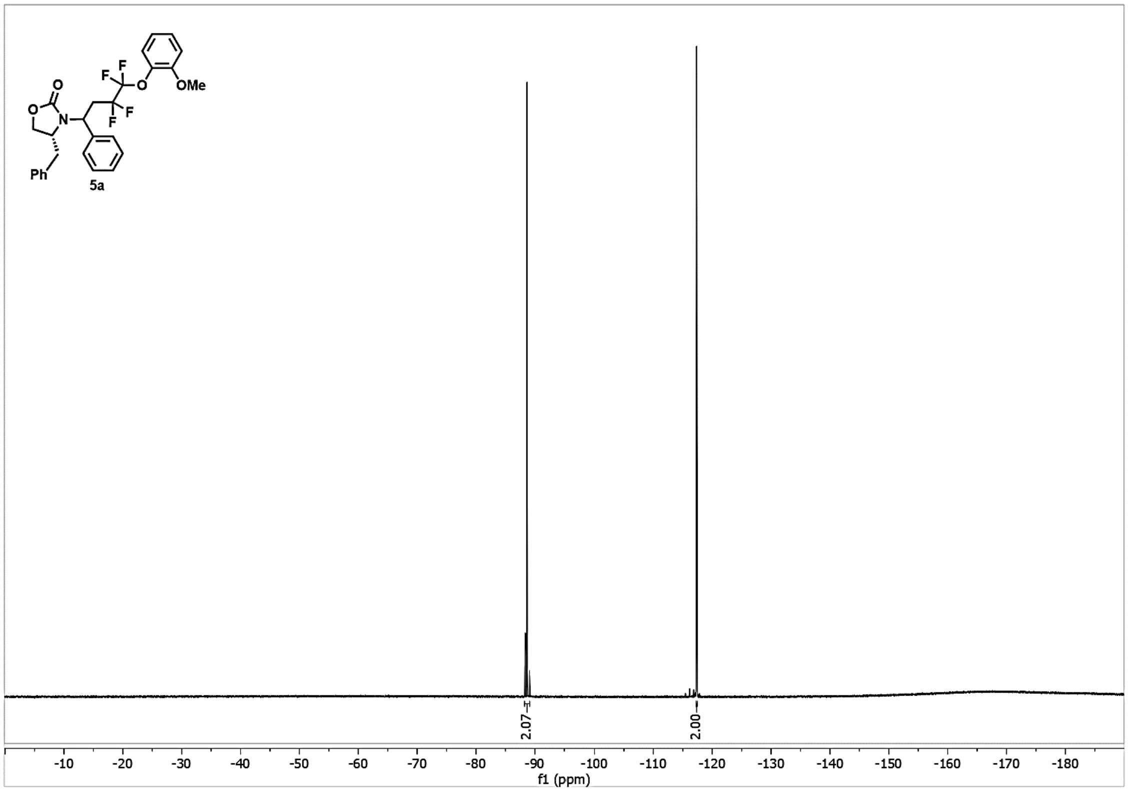

Compound 5b. Top: ^{1}H NMR ($CDCl_3$, 400 MHz). Bottom: ^{13}C NMR ($CDCl_3$, 100 MHz).

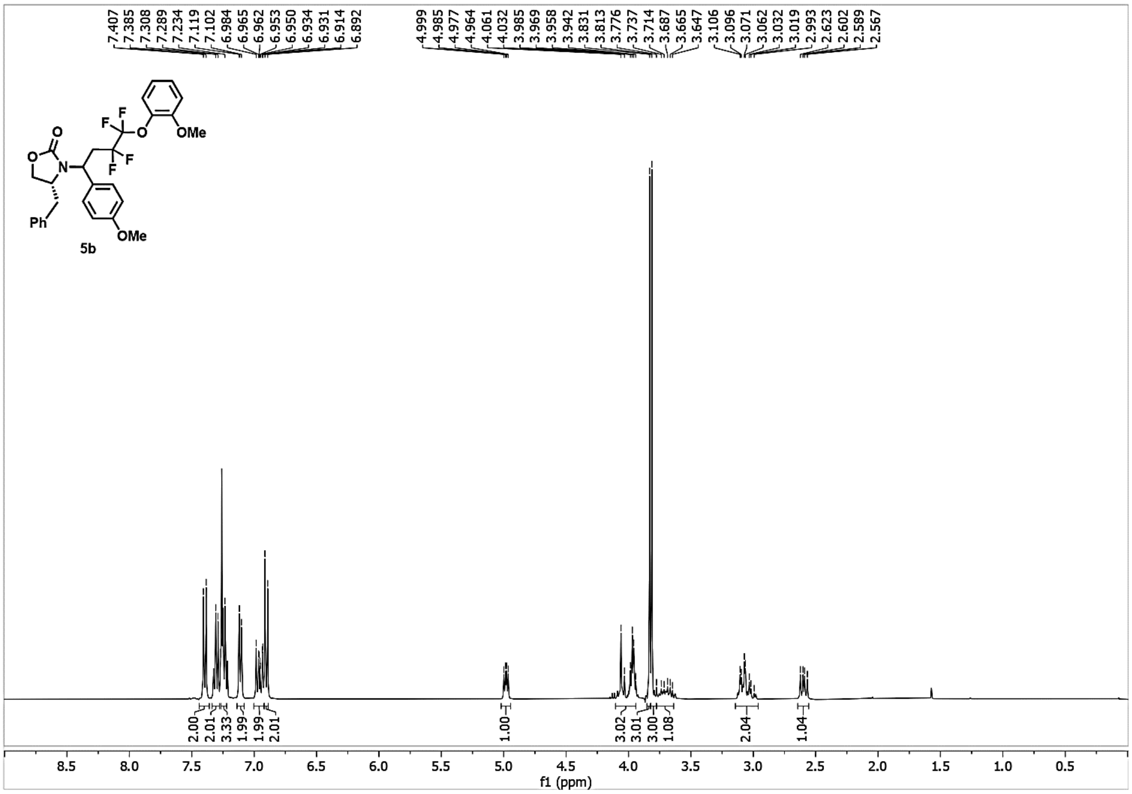

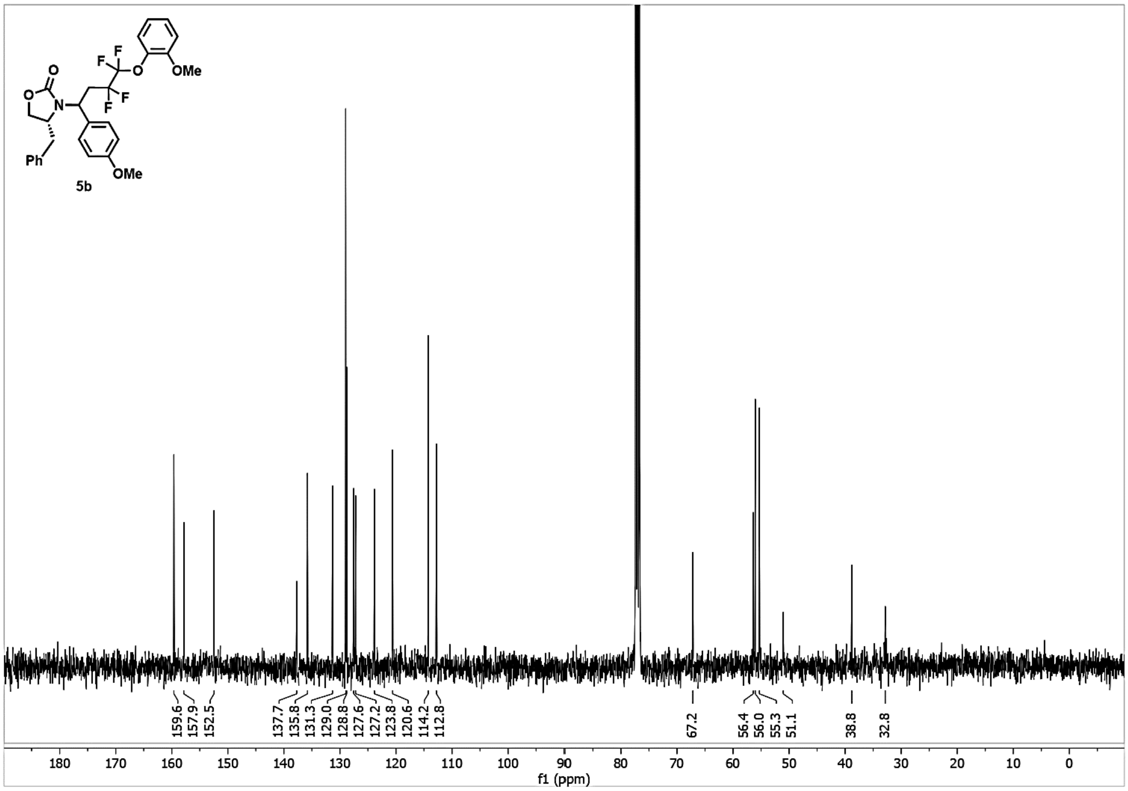

Compound 5b. ^{19}F NMR ($CDCl_3$, 376 MHz).

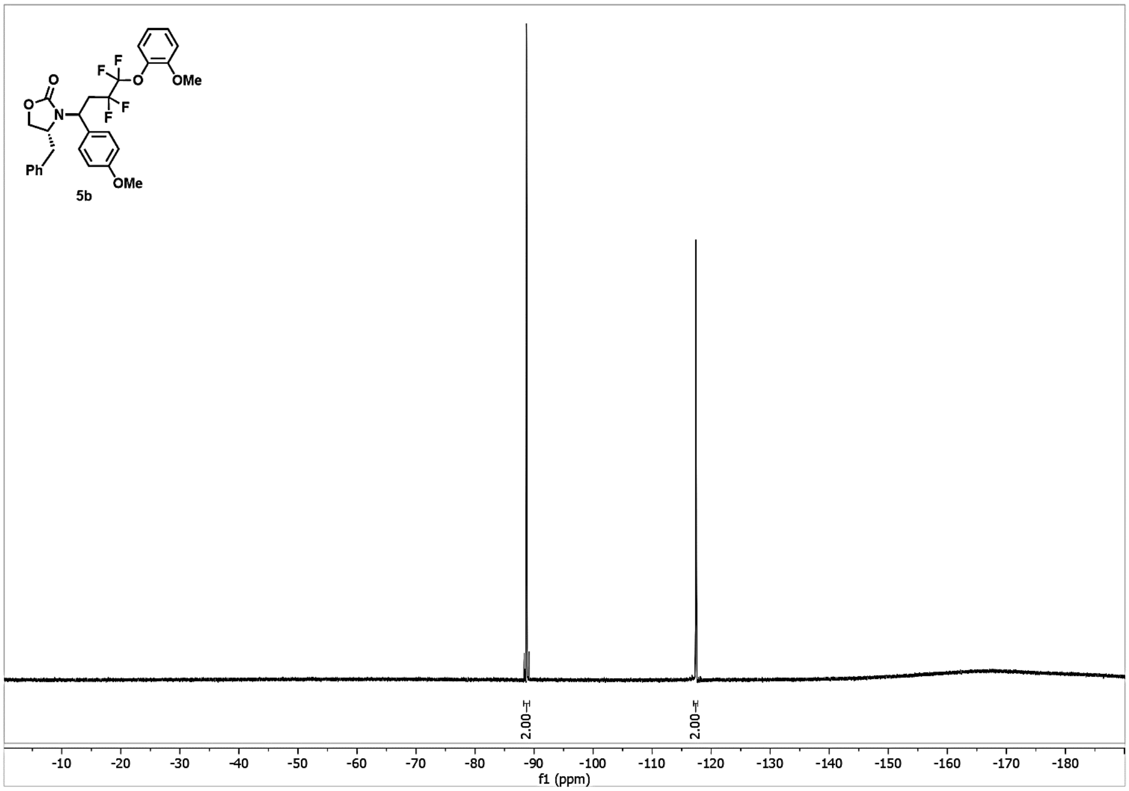

Compound 5c. Top: ^{1}H NMR ($CDCl_3$, 400 MHz). Bottom: ^{13}C NMR ($CDCl_3$, 100 MHz). Integrations shown for the major diastereoisomer.

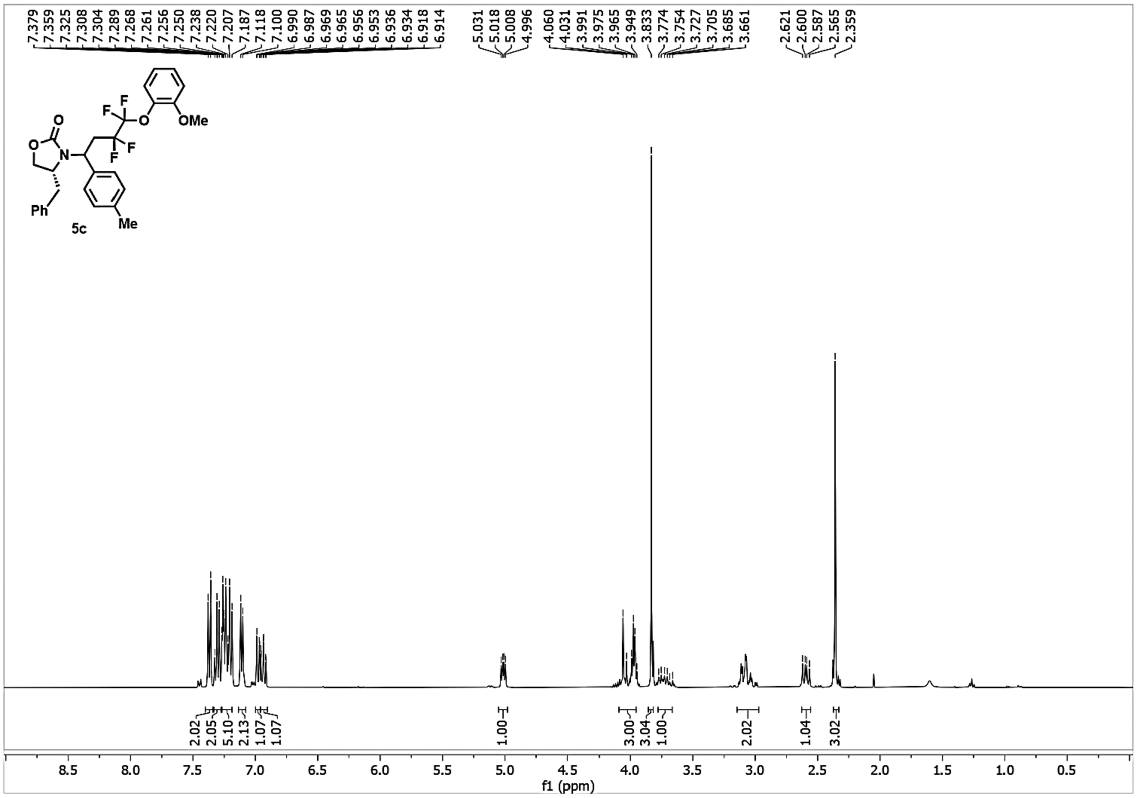

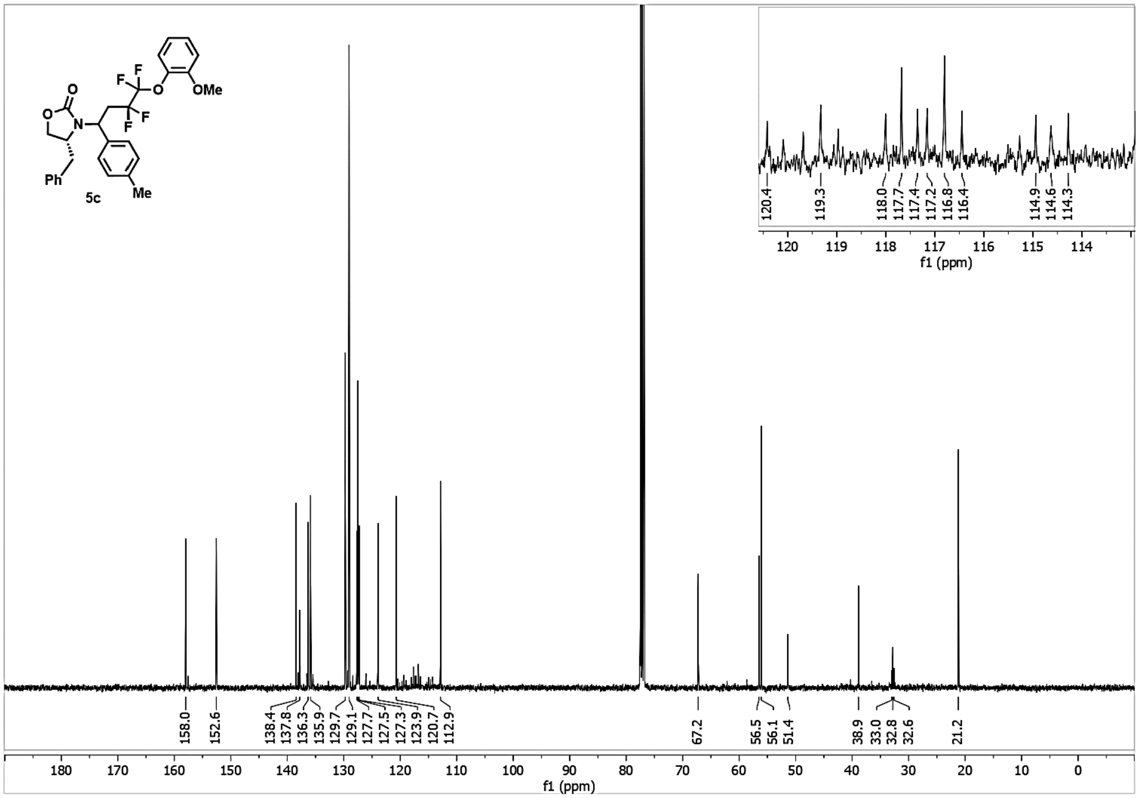

Compound 5c. ^{19}F NMR ($CDCl_3$, 376 MHz).

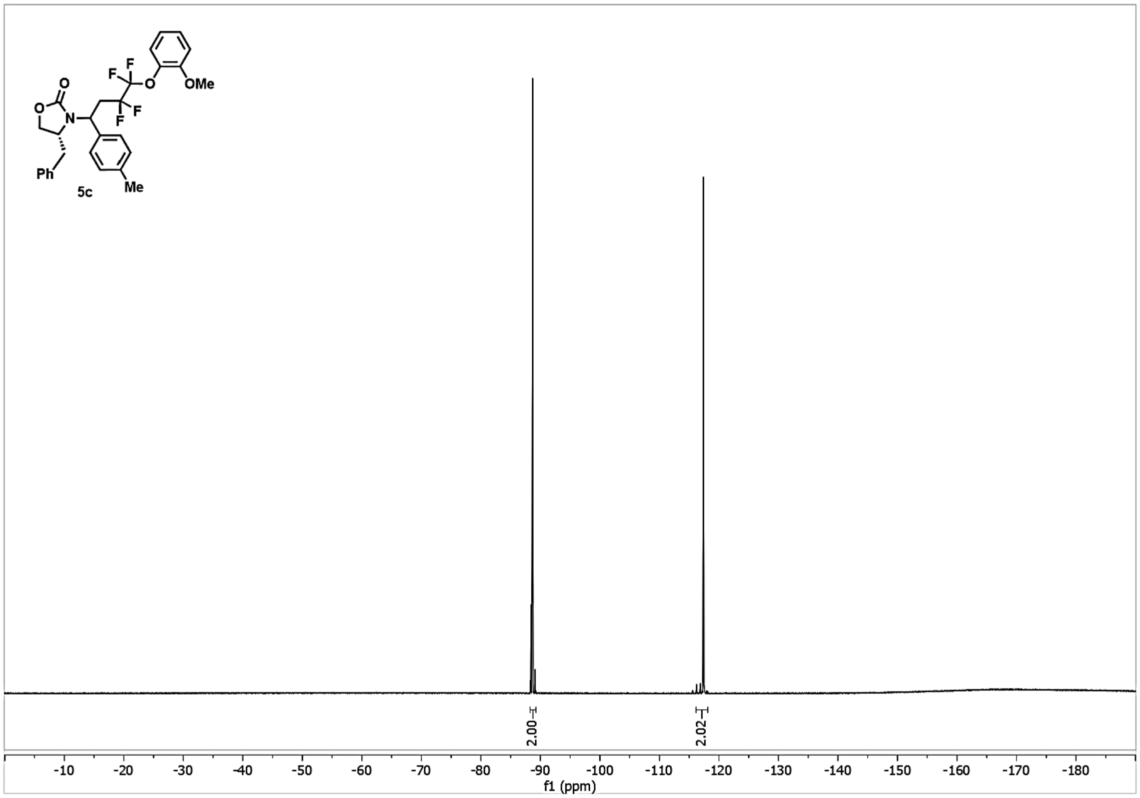

Compound 5d. Top: ^{1}H NMR ($CDCl_3$, 400 MHz). Bottom: ^{13}C NMR ($CDCl_3$, 100 MHz). Integrations shown for the major diastereoisomer.

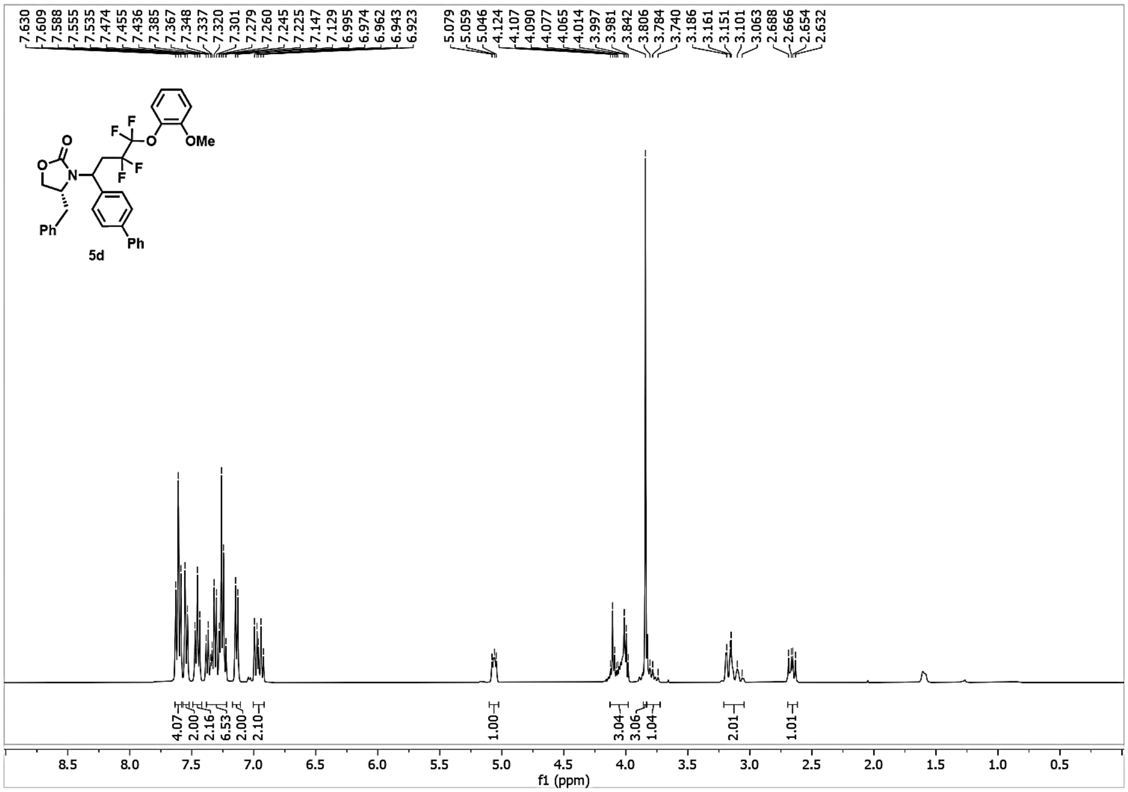

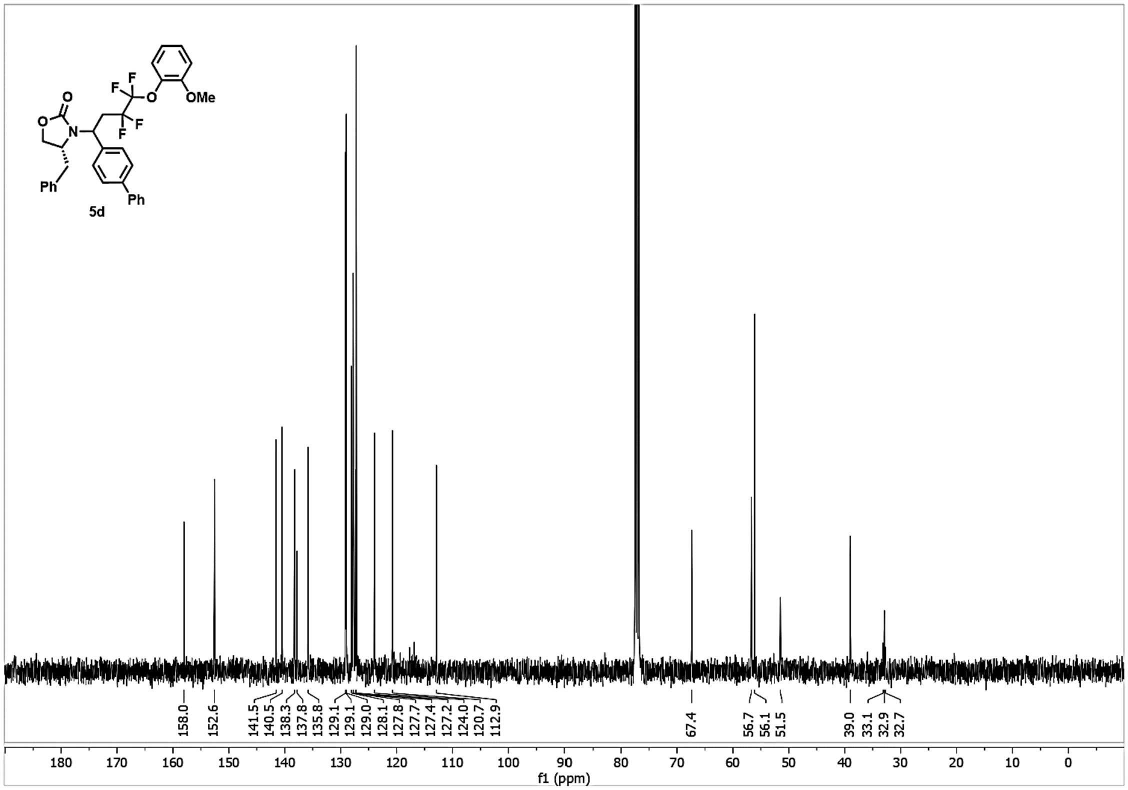

Compound 5d. ^{19}F NMR ($CDCl_3$, 376 MHz).

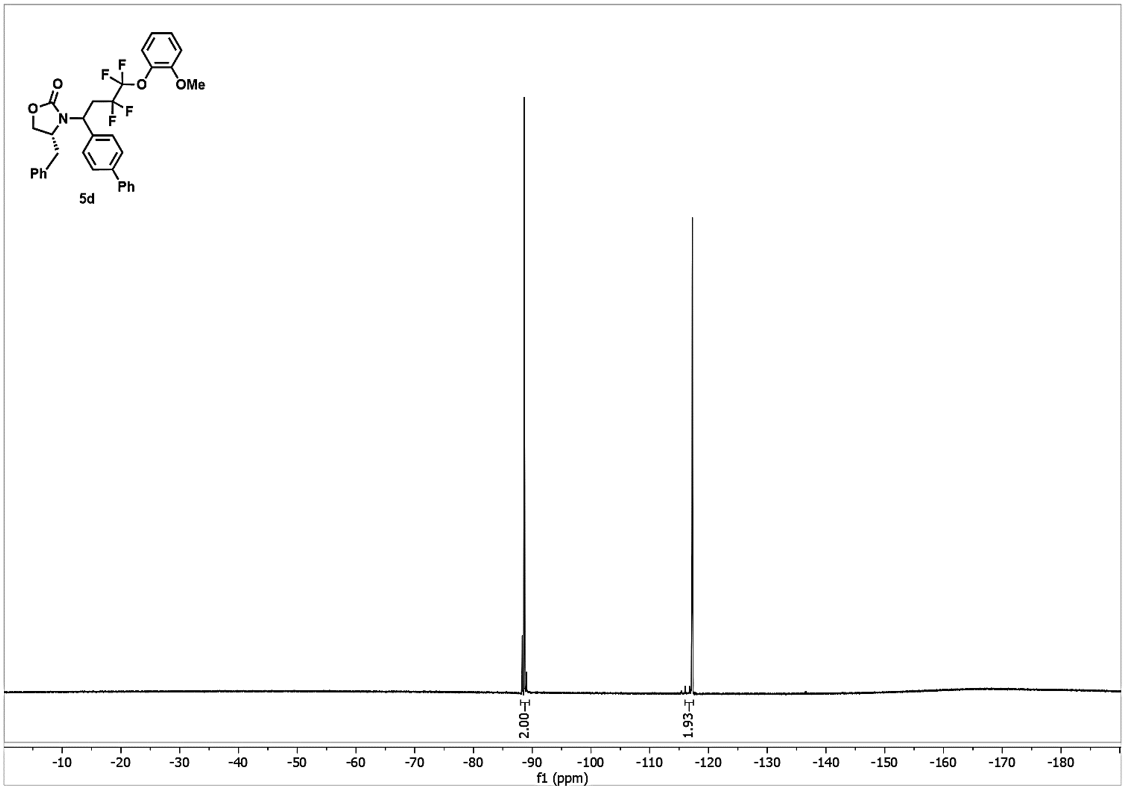

Compound 5e. Top: ^{1}H NMR ($CDCl_3$, 400 MHz). Bottom: ^{13}C NMR ($CDCl_3$, 100 MHz). Integrations shown for the major diastereoisomer.

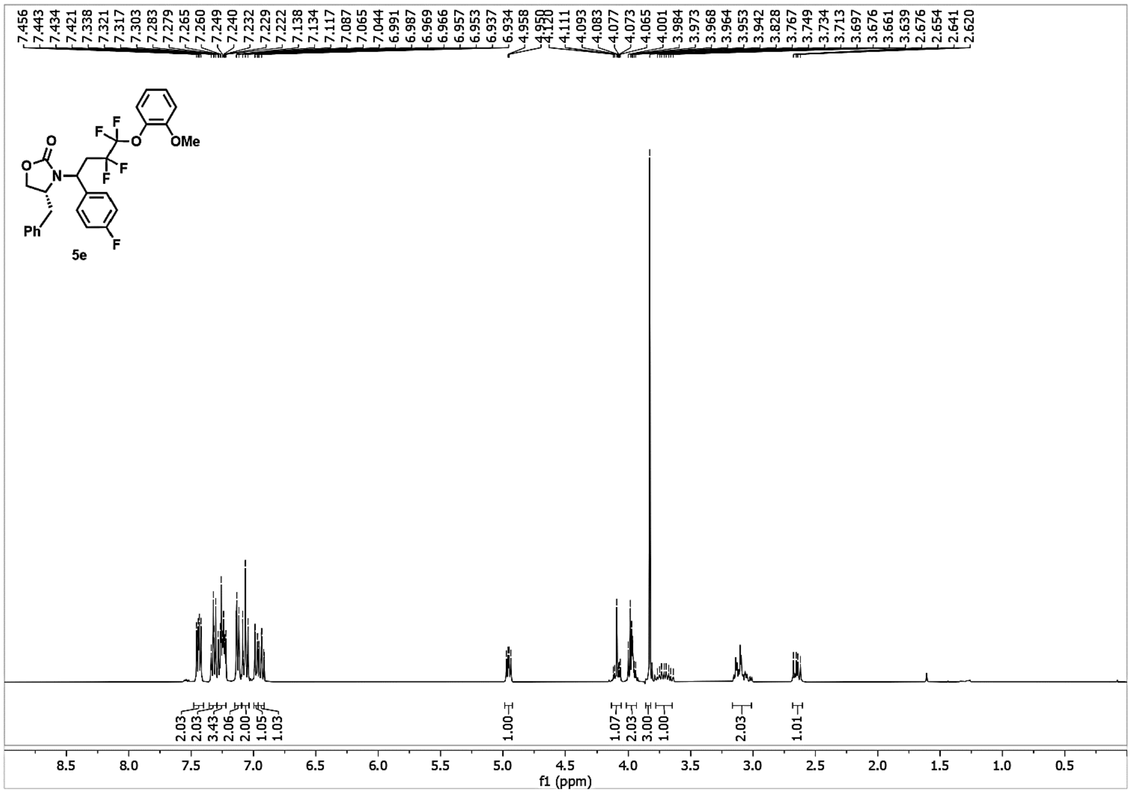

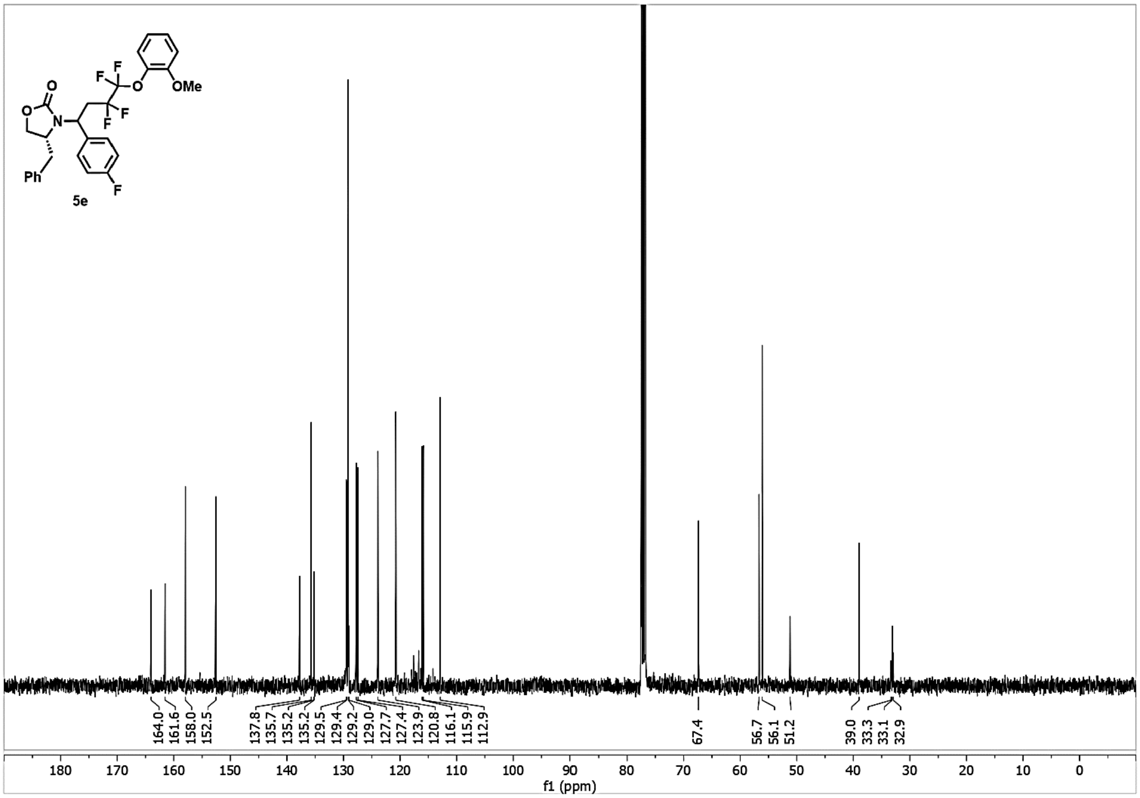

Compound 5e. ^{19}F NMR ($CDCl_3$, 376 MHz).

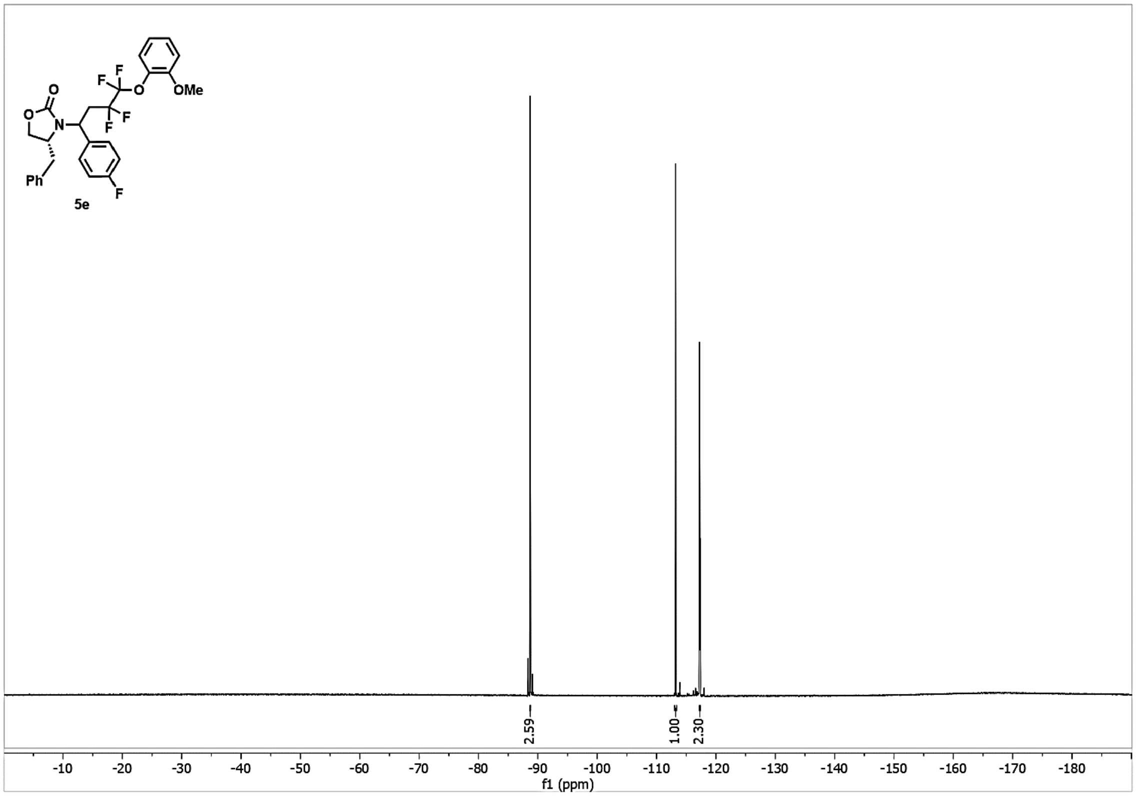

Compound 5f. Top: ^1H NMR ($CDCl_3$, 400 MHz). Bottom: ^{13}C NMR ($CDCl_3$, 100 MHz). Integrations shown for the major diastereoisomer.

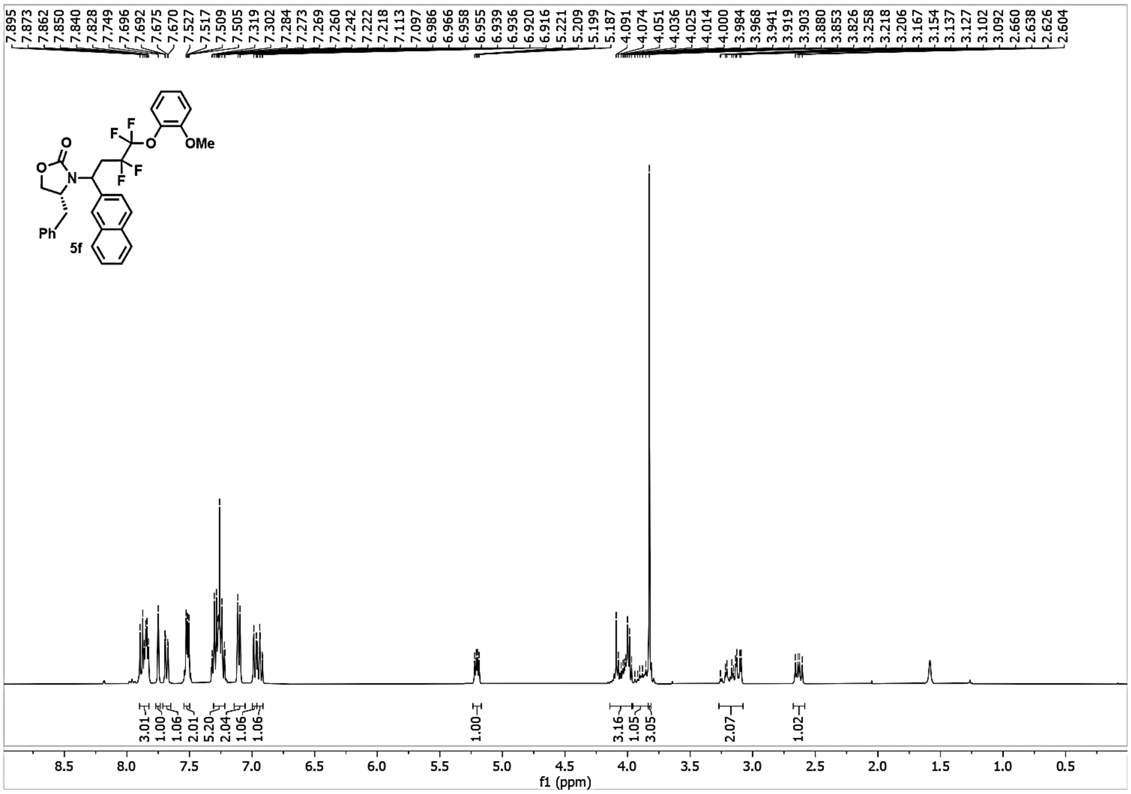

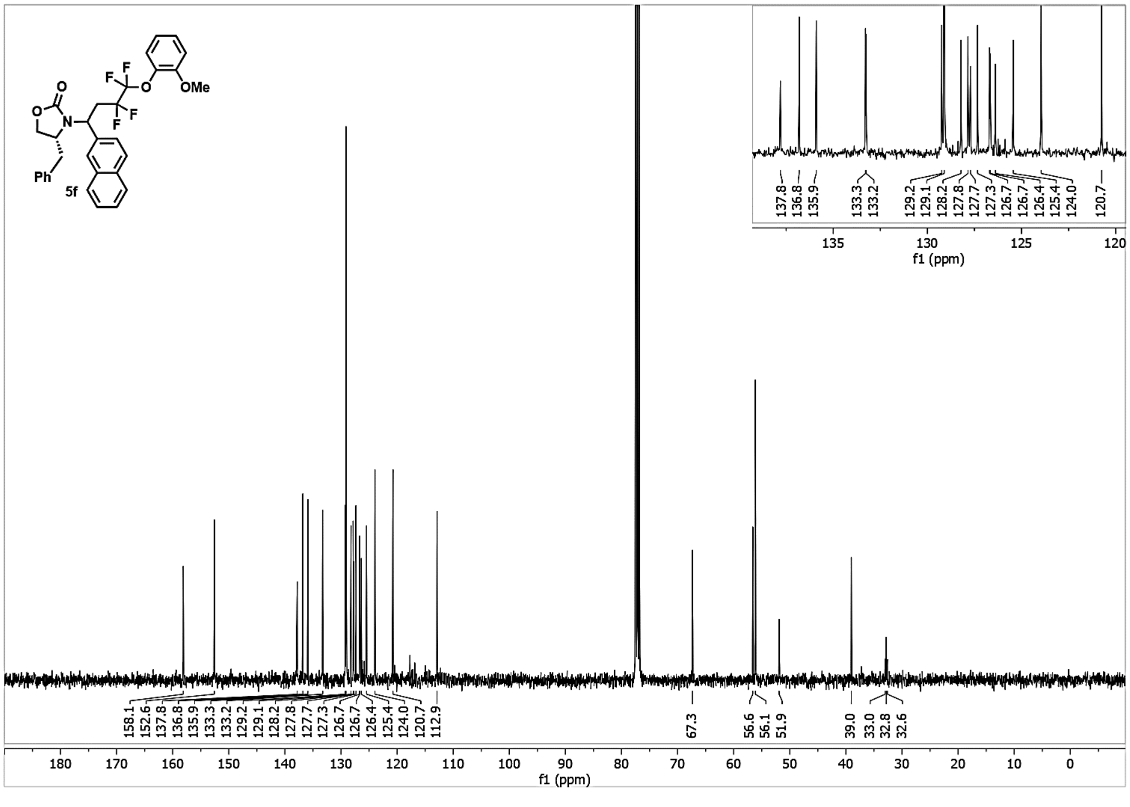

Compound 5f. ^{19}F NMR ($CDCl_3$, 376 MHz).

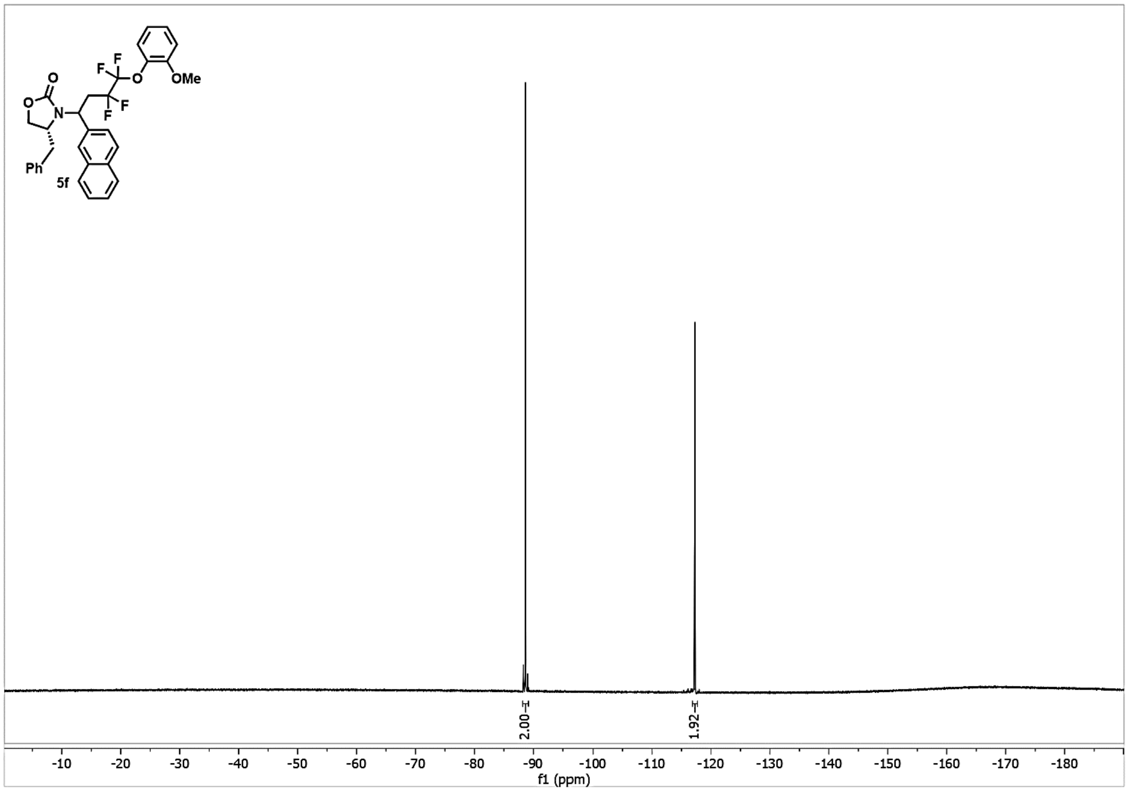

Compound 5g. Top: ^{1}H NMR ($CDCl_3$, 400 MHz). Bottom: ^{13}C NMR ($CDCl_3$, 100 MHz).

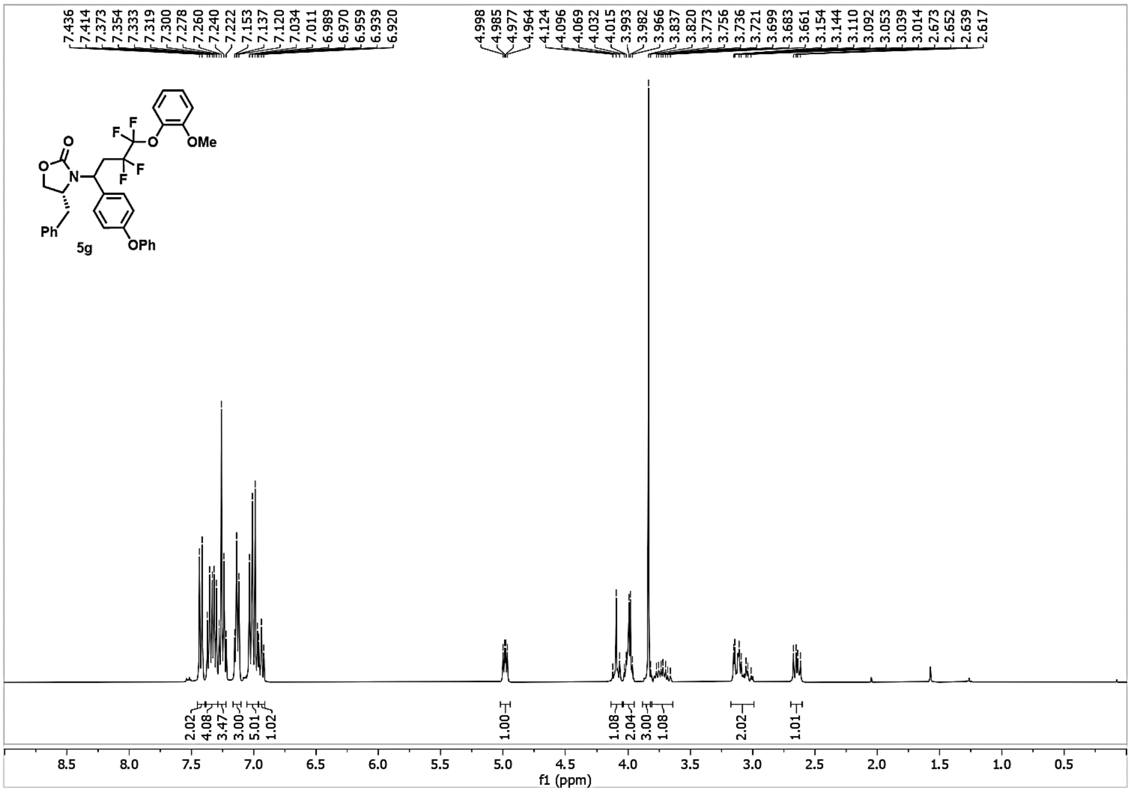

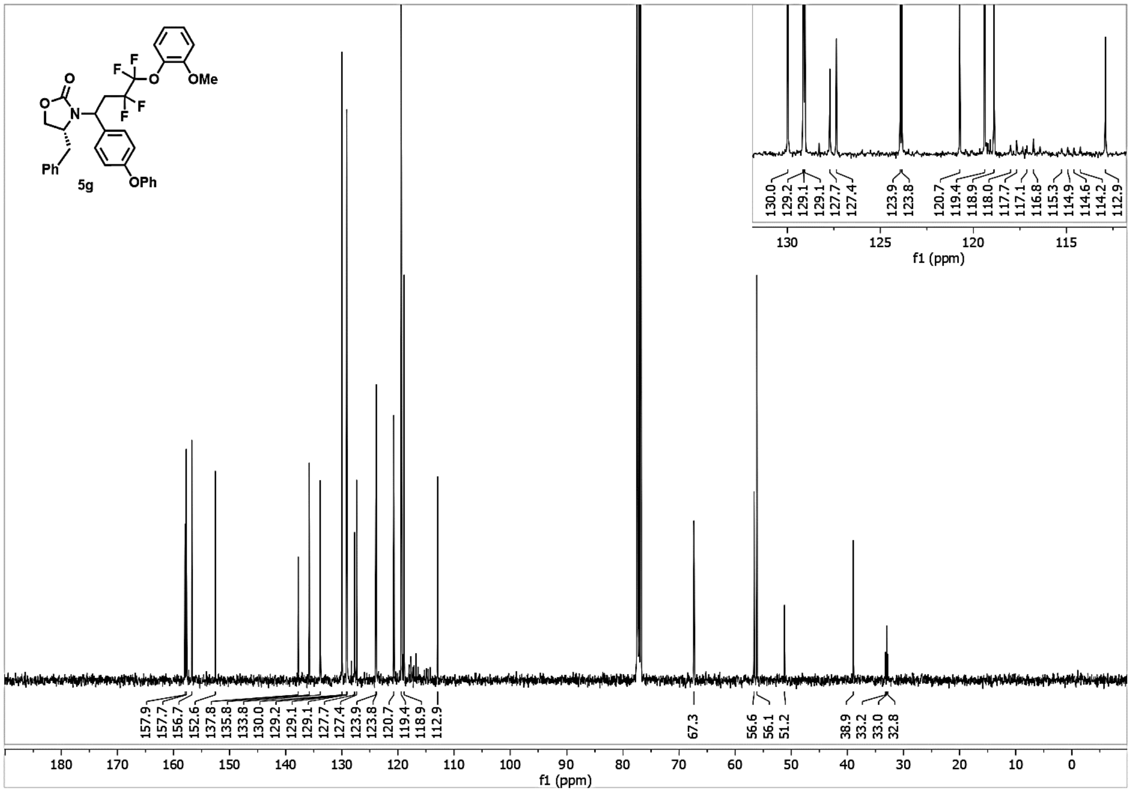

Compound 5g. ^{19}F NMR ($CDCl_3$, 376 MHz).

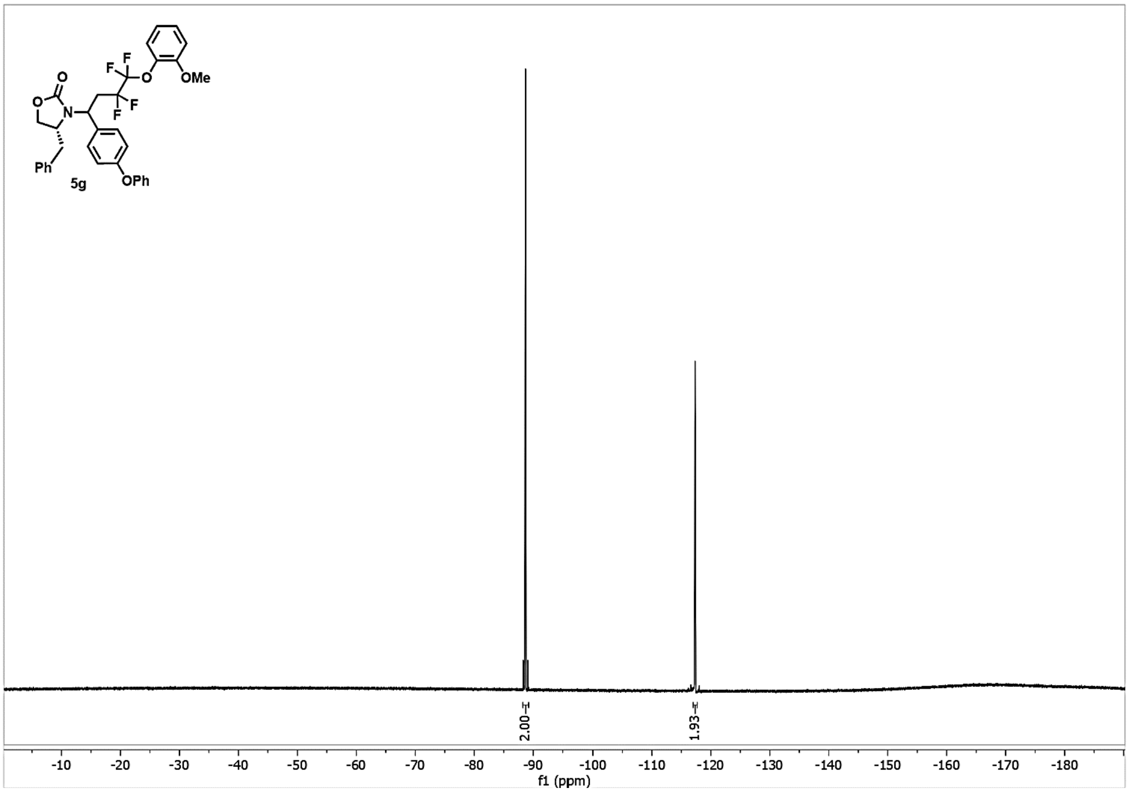

Compound 5h. Top: ^{1}H NMR ($CDCl_3$, 400 MHz). Bottom: ^{13}C NMR ($CDCl_3$, 100 MHz).

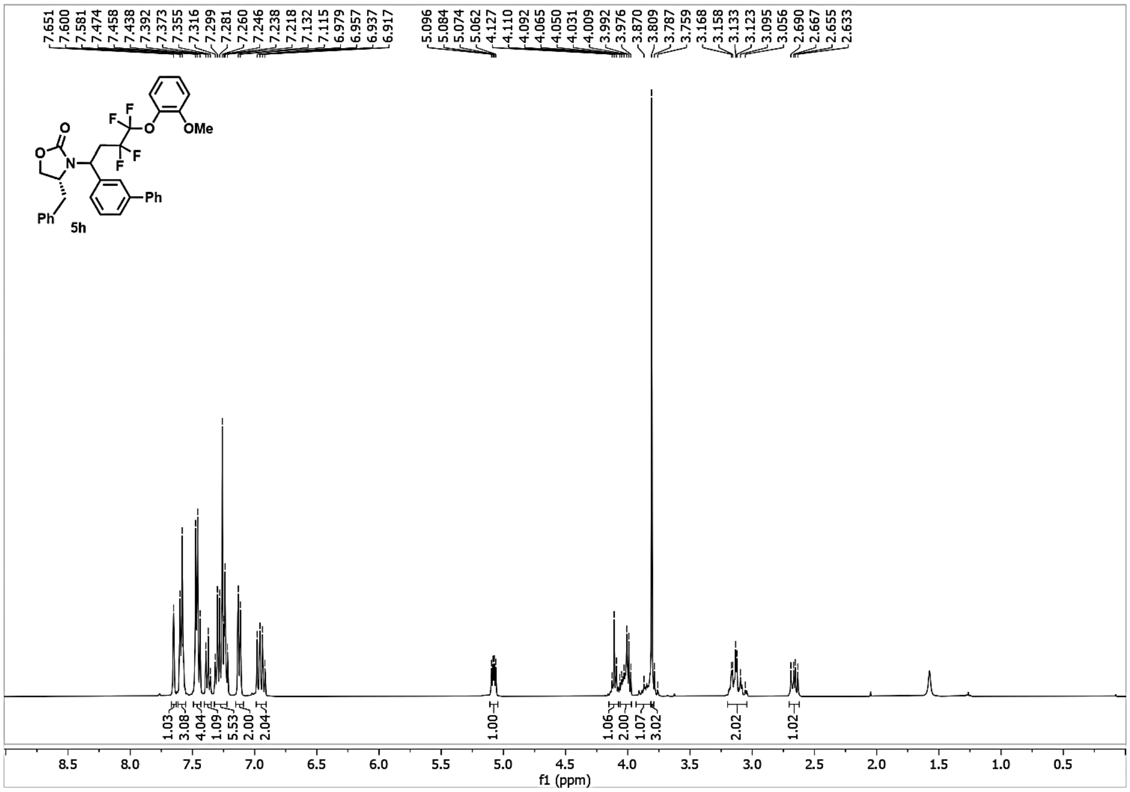

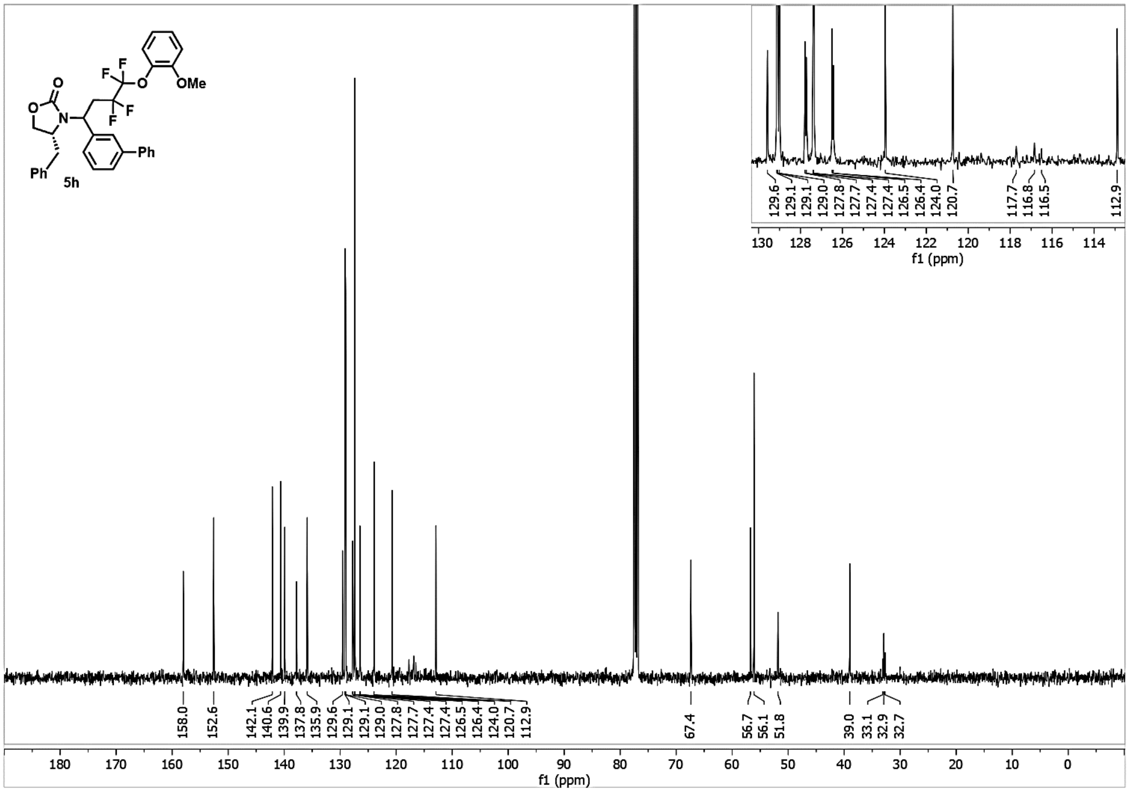

Compound 5h. ^{19}F NMR ($CDCl_3$, 376 MHz).

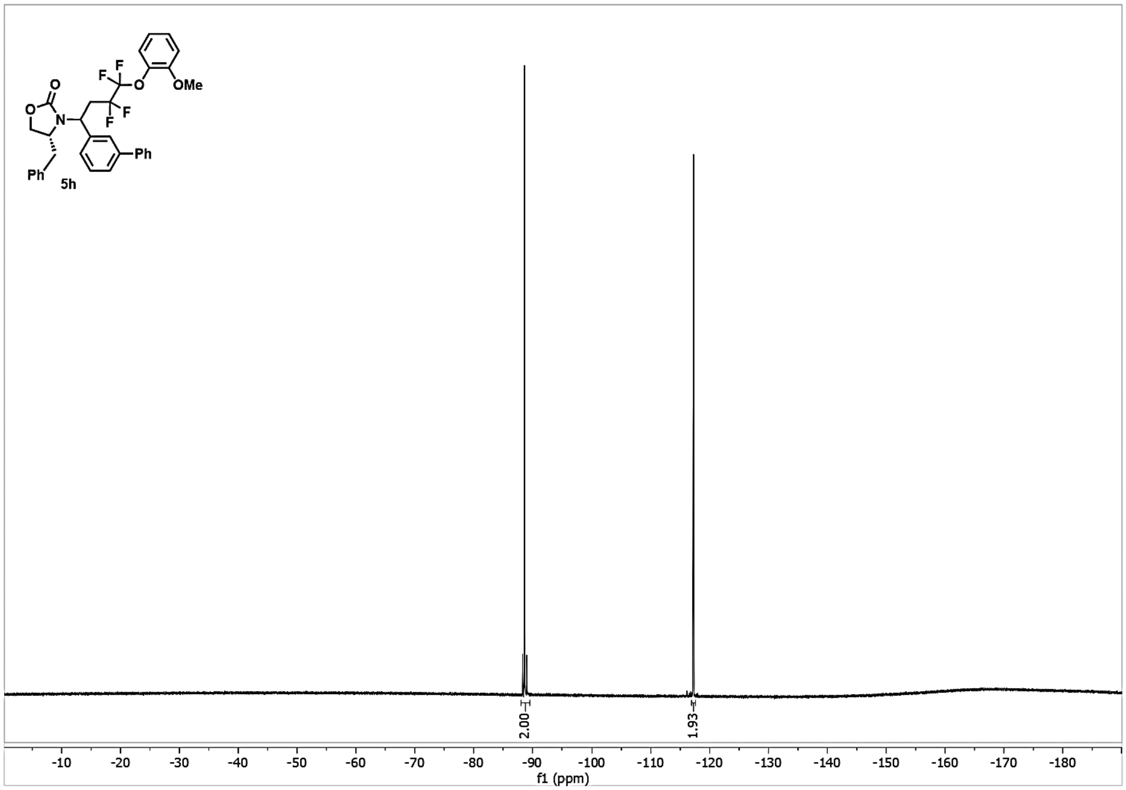

Compound 5i. Top: ^{1}H NMR ($CDCl_3$, 400 MHz). Bottom: ^{13}C NMR ($CDCl_3$, 100 MHz).

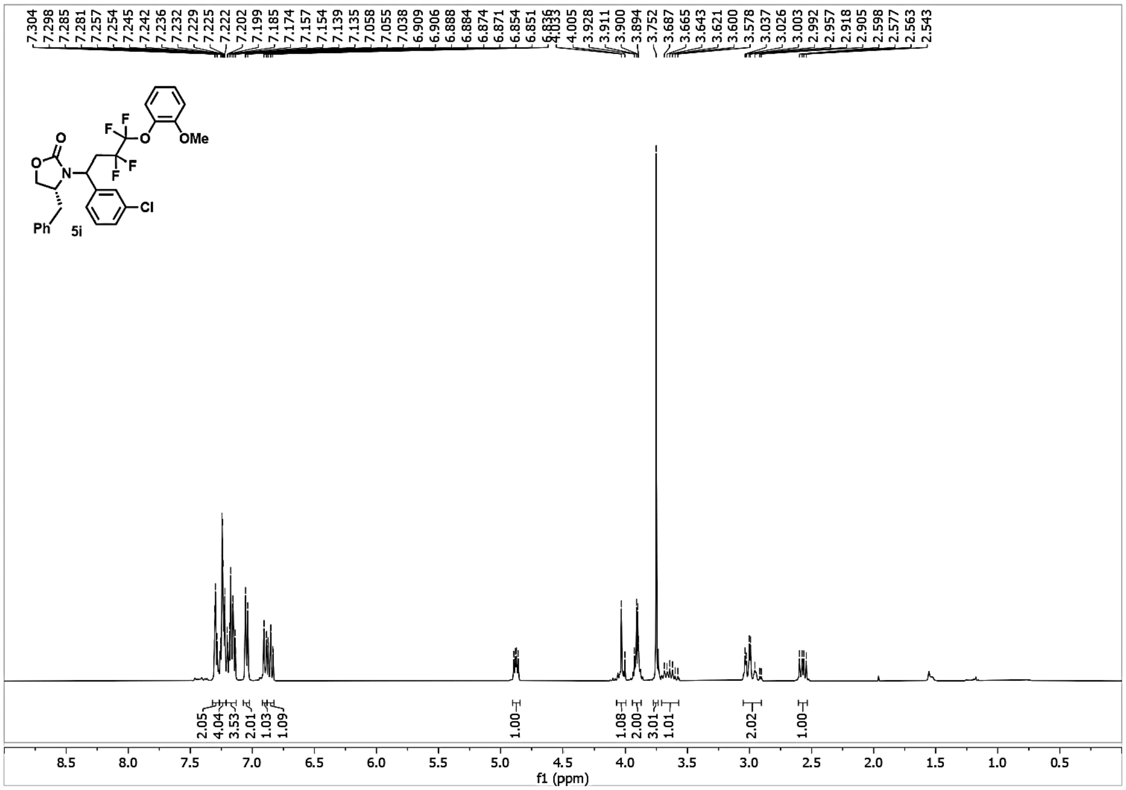

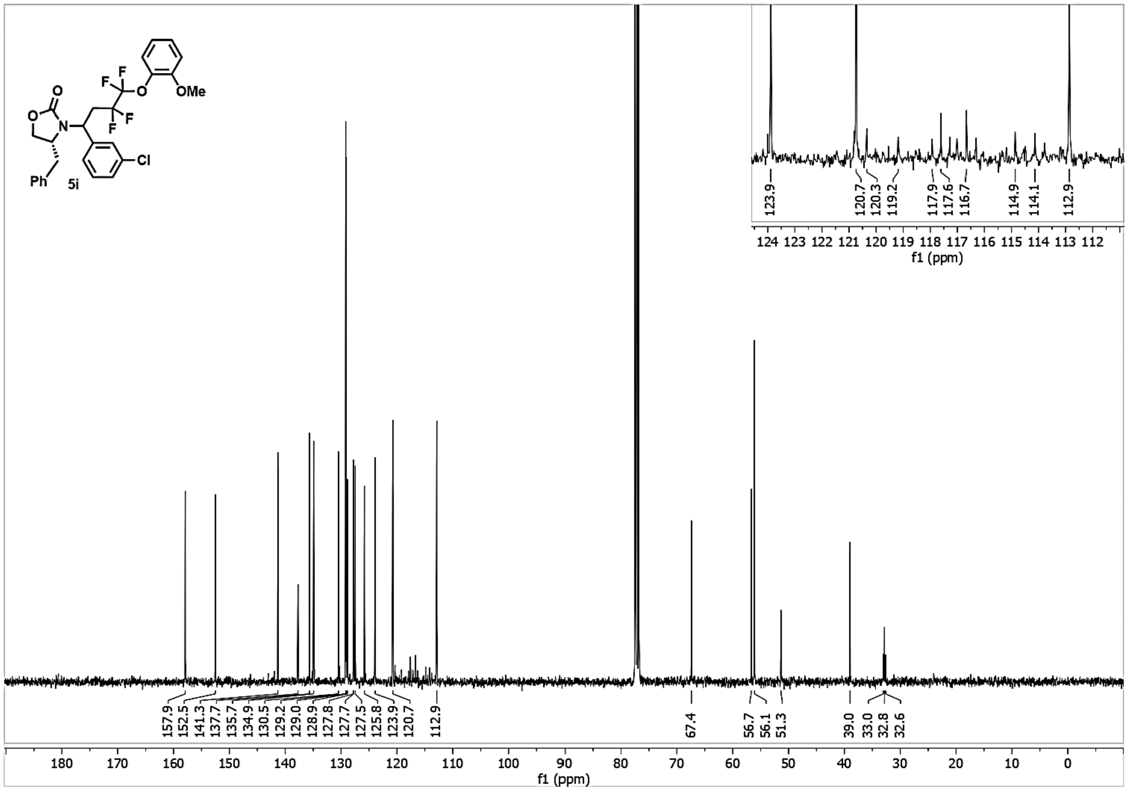

Compound 5i. ^{19}F NMR ($CDCl_3$, 376 MHz).

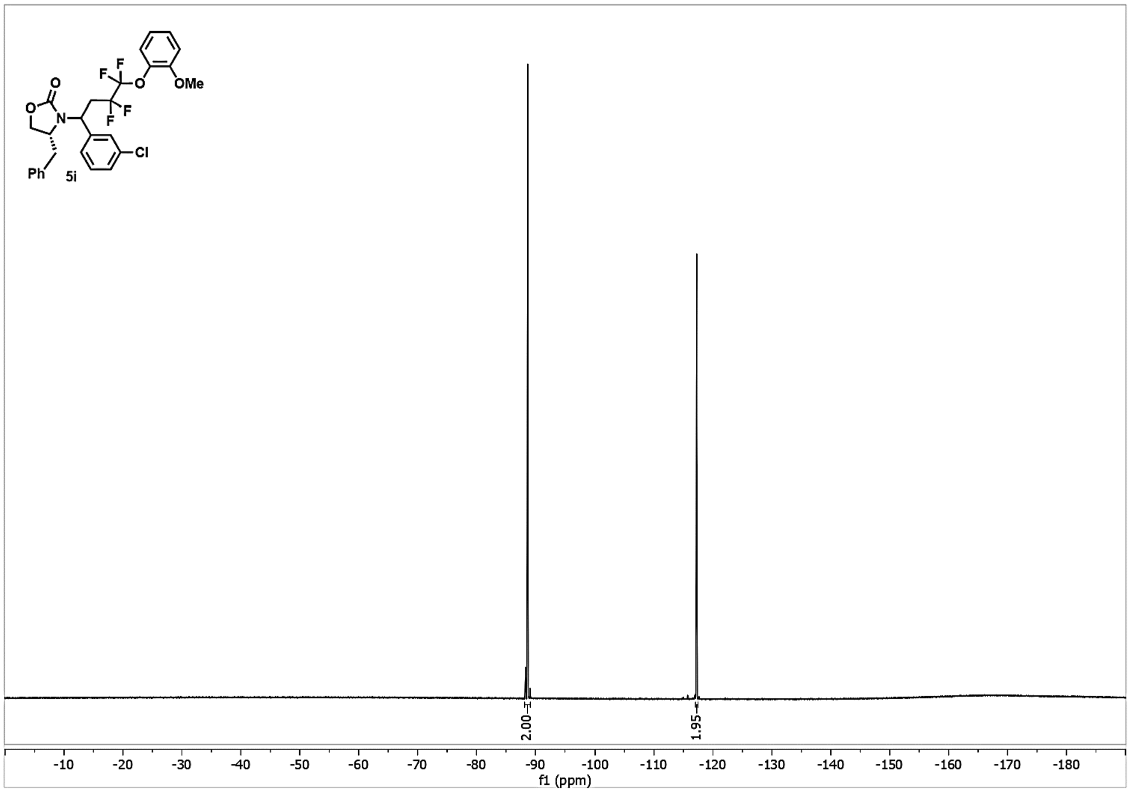

Compound 5j. Top: ^{1}H NMR ($CDCl_3$, 400 MHz). Bottom: ^{13}C NMR ($CDCl_3$, 100 MHz).

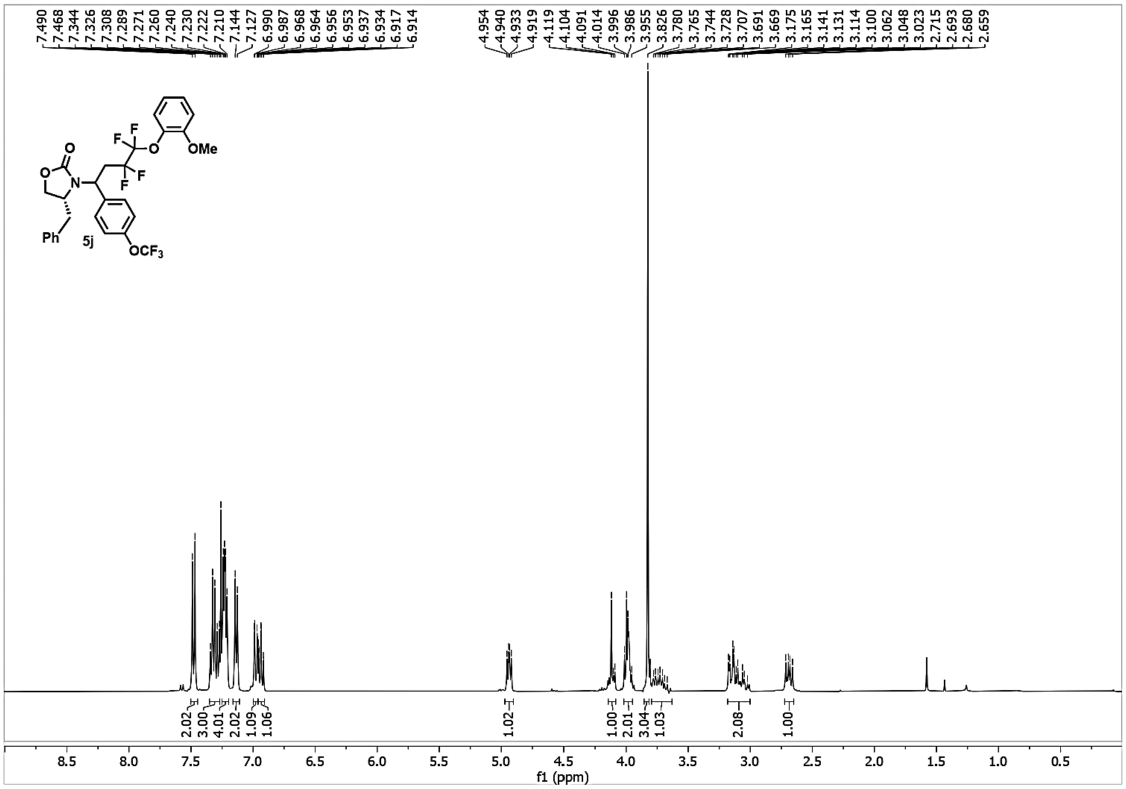

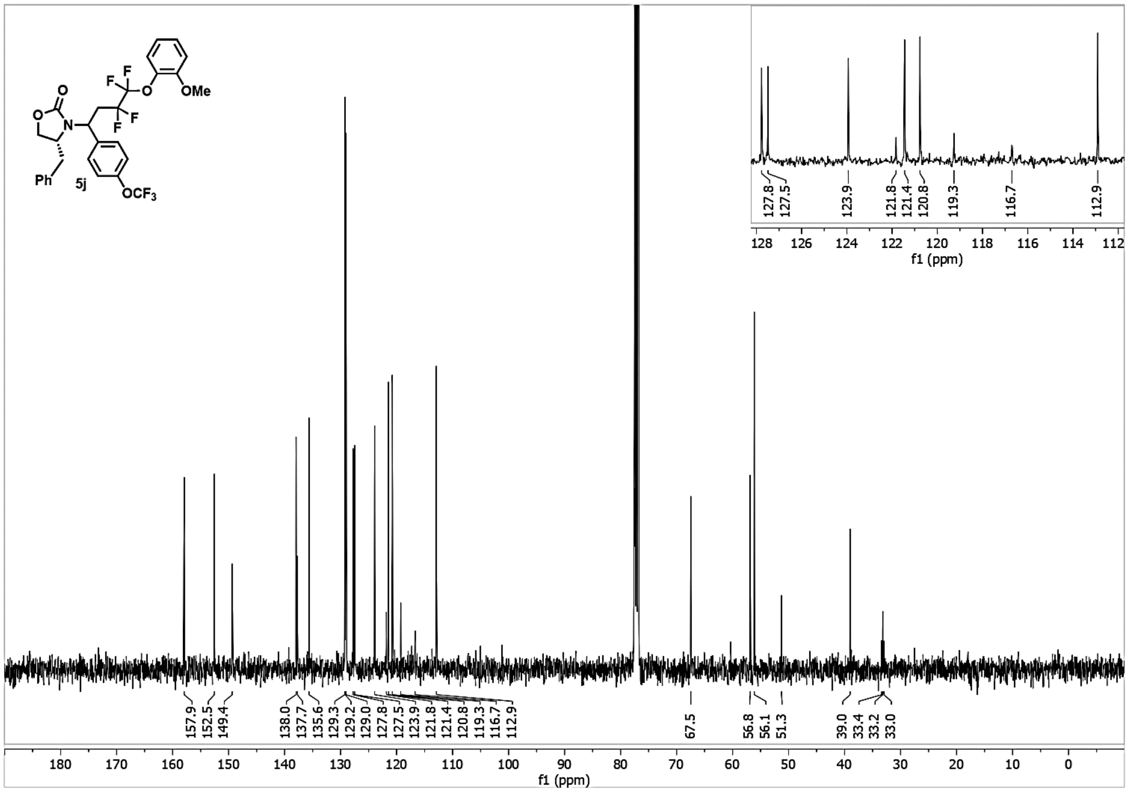

Compound 5j. ^{19}F NMR ($CDCl_3$, 376 MHz).

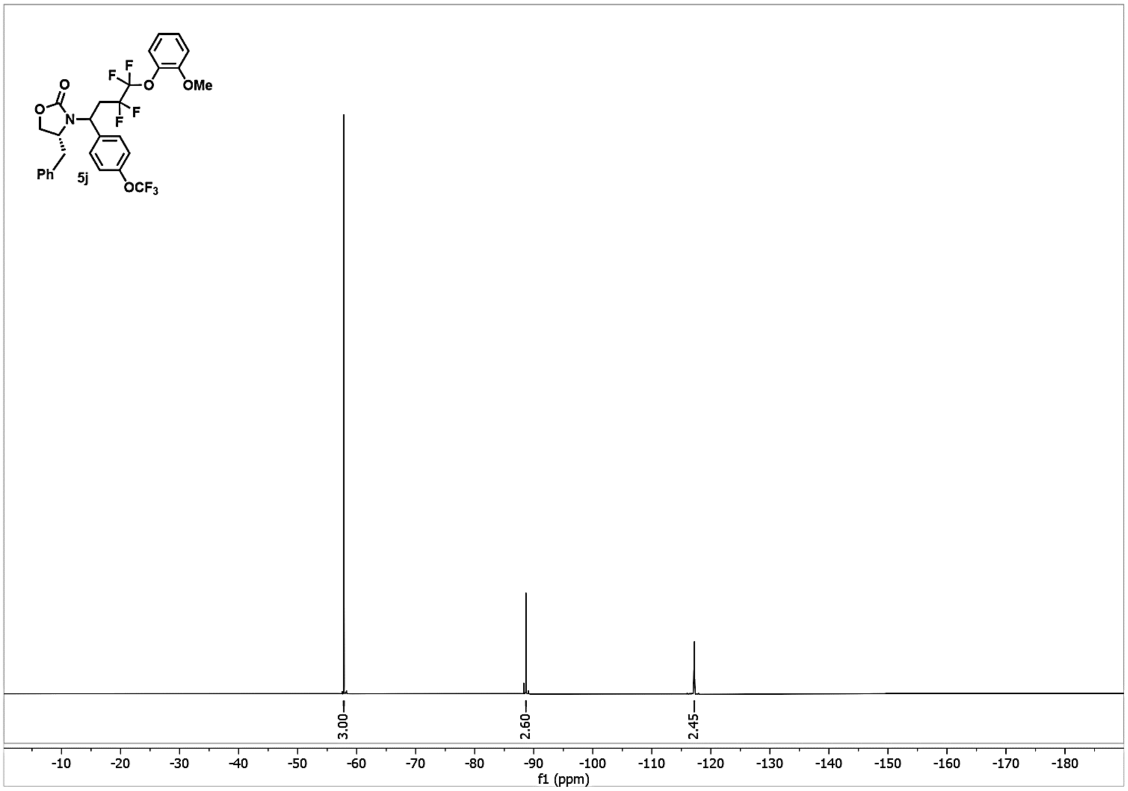

Compound 5k. Top: ^{1}H NMR ($CDCl_3$, 400 MHz). Bottom: ^{13}C NMR ($CDCl_3$, 100 MHz). Integrations shown for the major diastereoisomer.

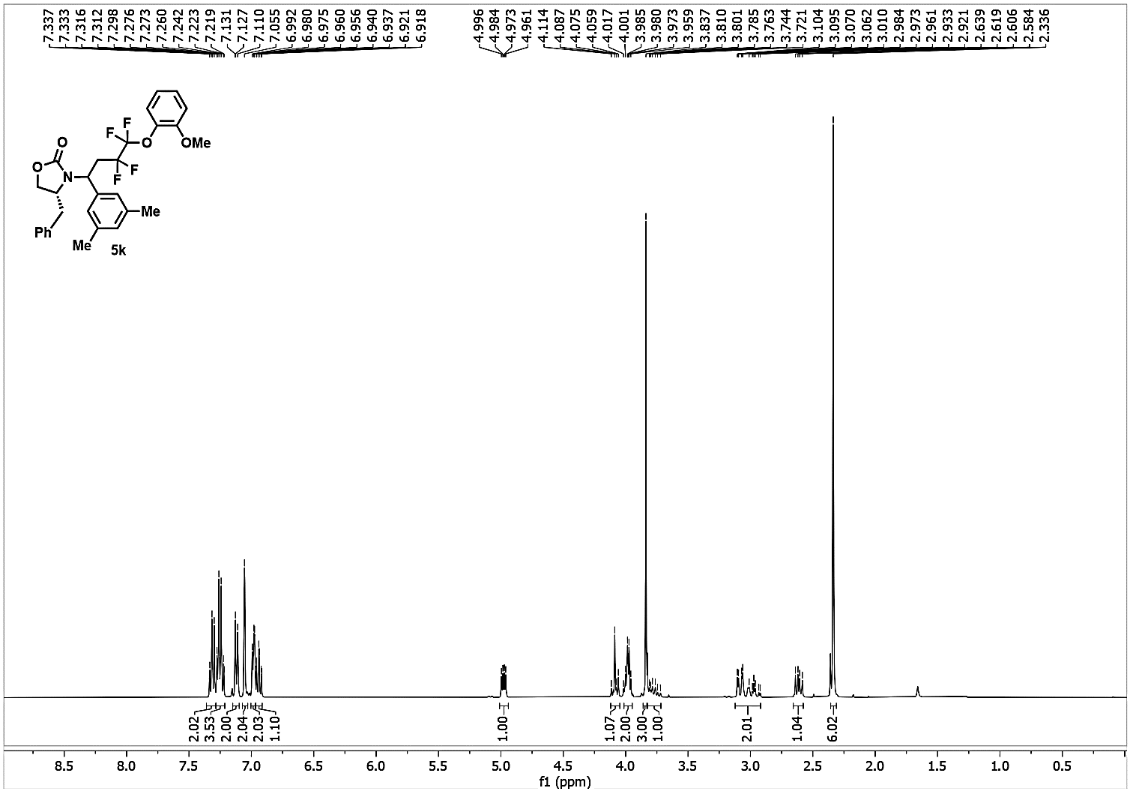

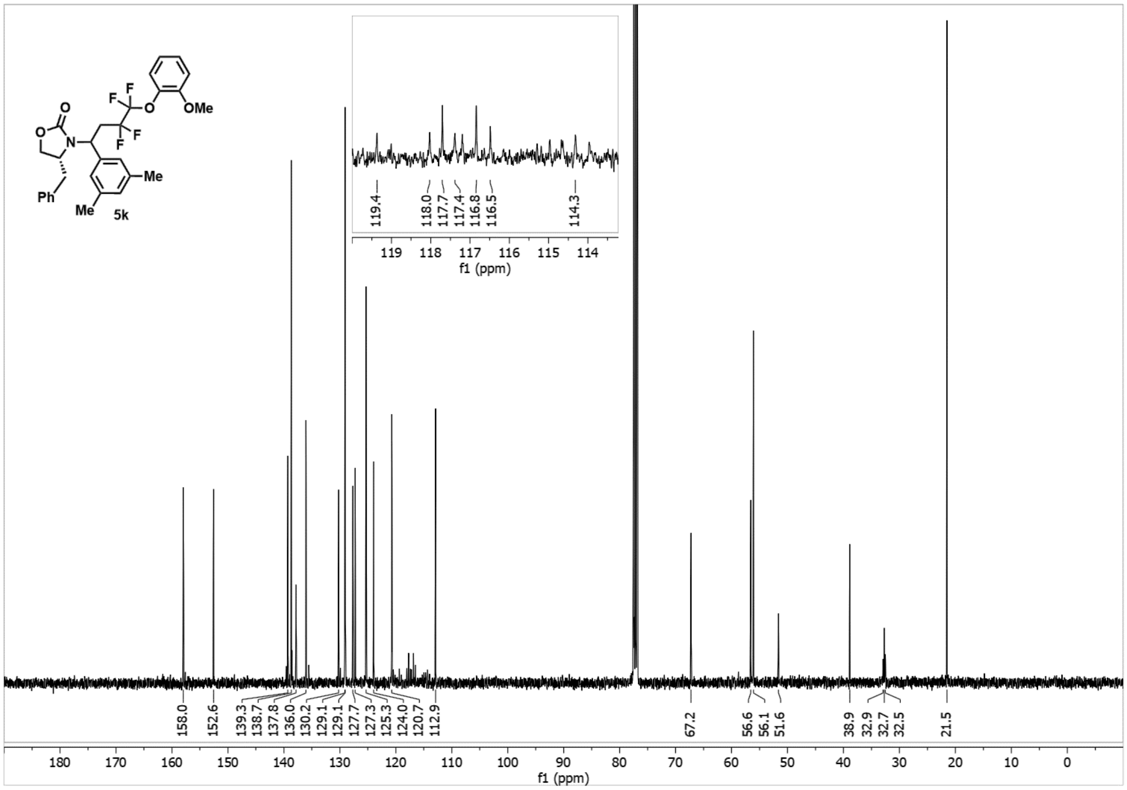

Compound 5k. ^{19}F NMR ($CDCl_3$, 376 MHz).

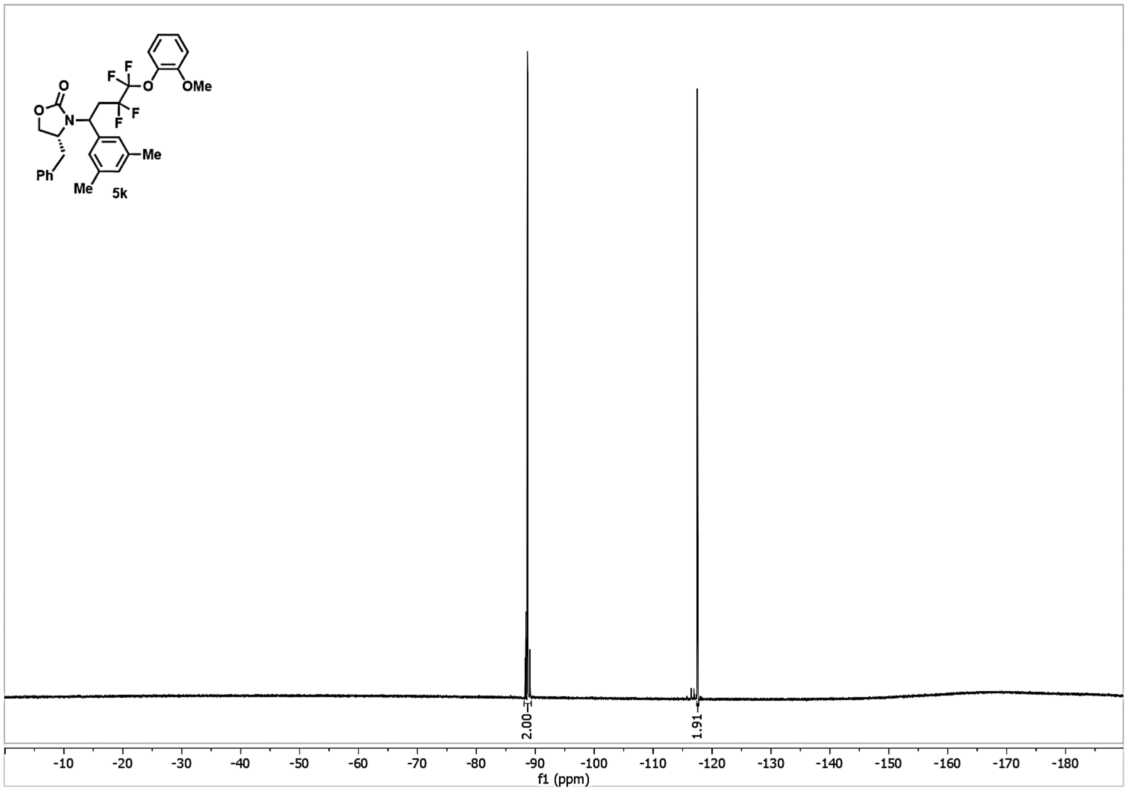

Compound 5l. Top: ^{1}H NMR ($CDCl_3$, 400 MHz). Bottom: ^{13}C NMR ($CDCl_3$, 100 MHz).

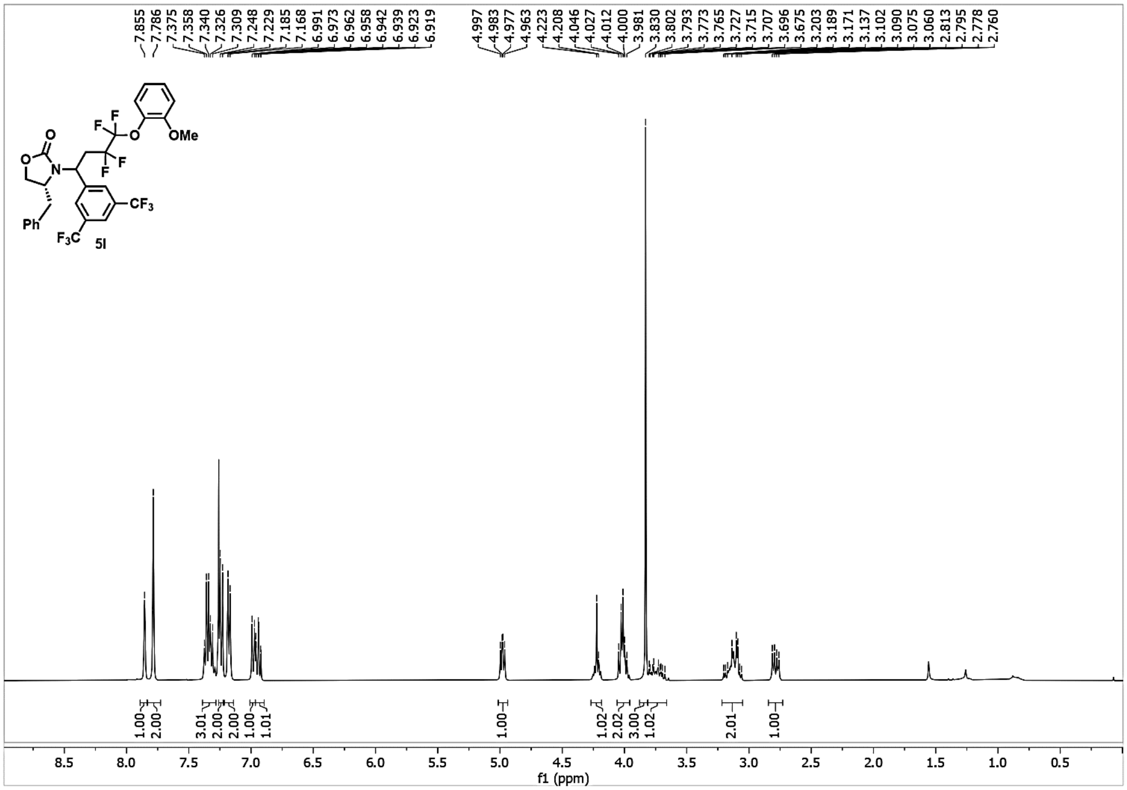

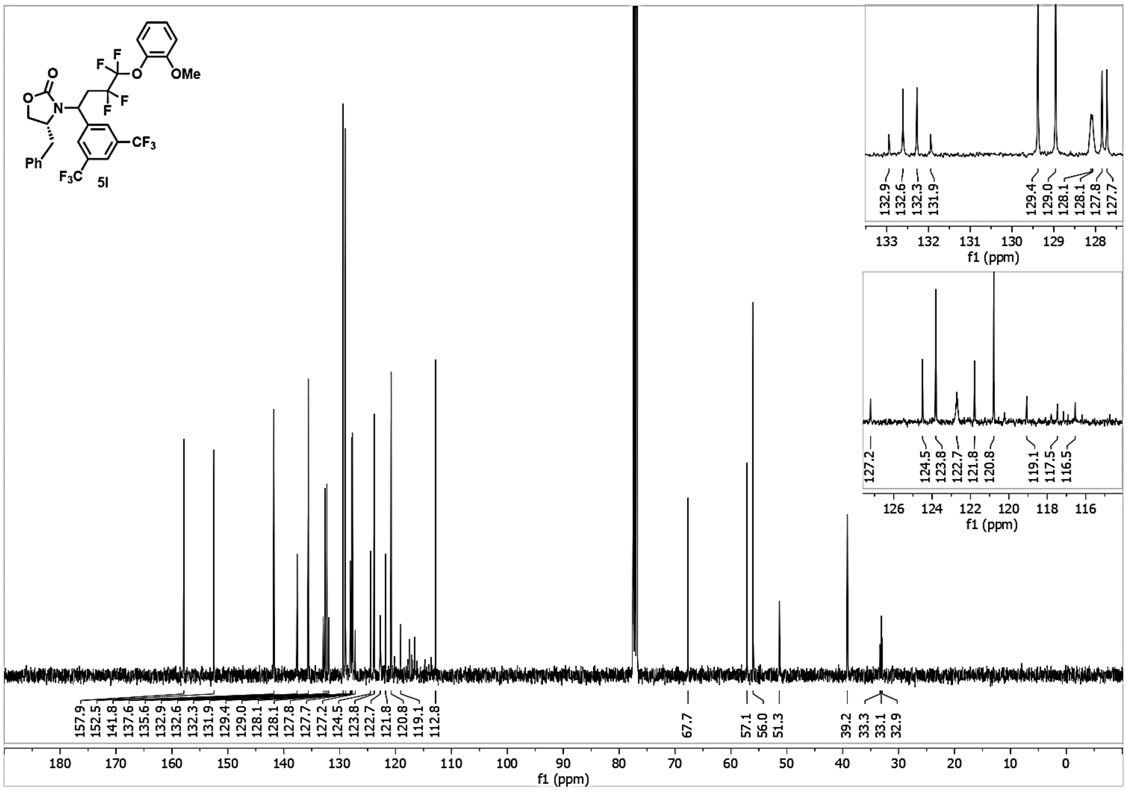

Compound 5l. ^{19}F NMR ($CDCl_3$, 376 MHz).

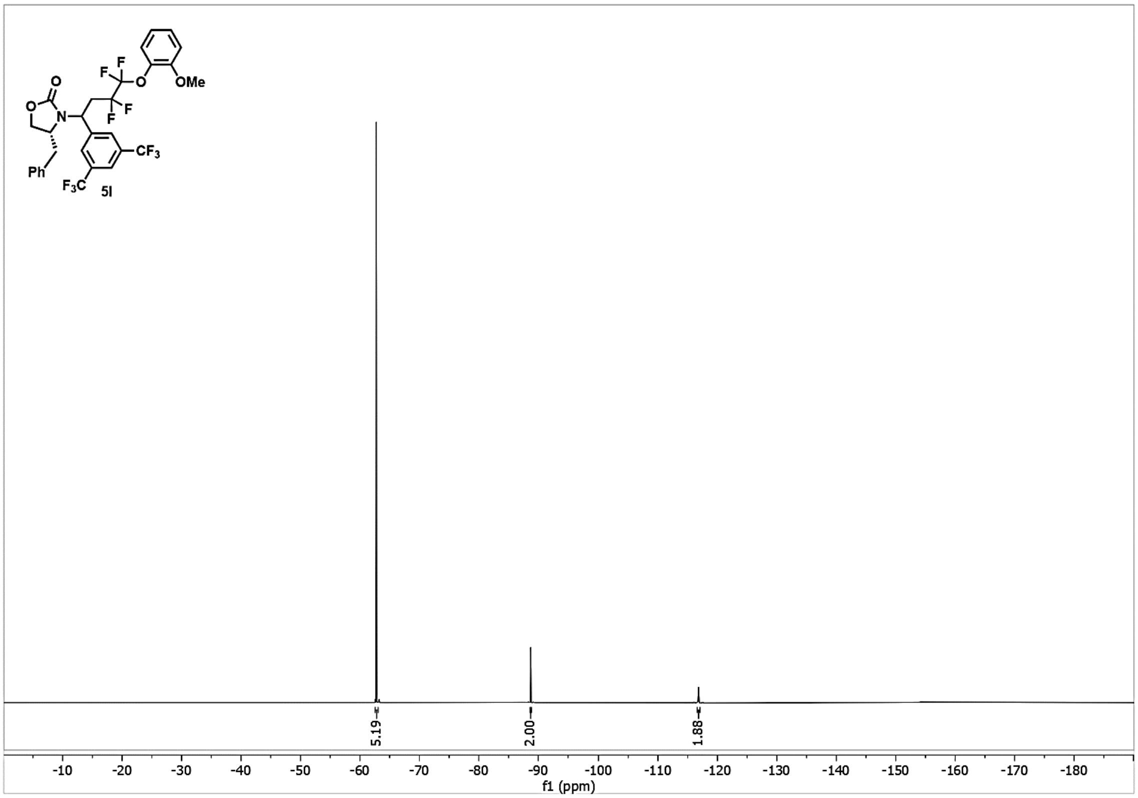

Compound 5m. Top: ^{1}H NMR ($CDCl_3$, 400 MHz). Bottom: ^{13}C NMR ($CDCl_3$, 100 MHz). Integrations shown for the major diastereoisomer.

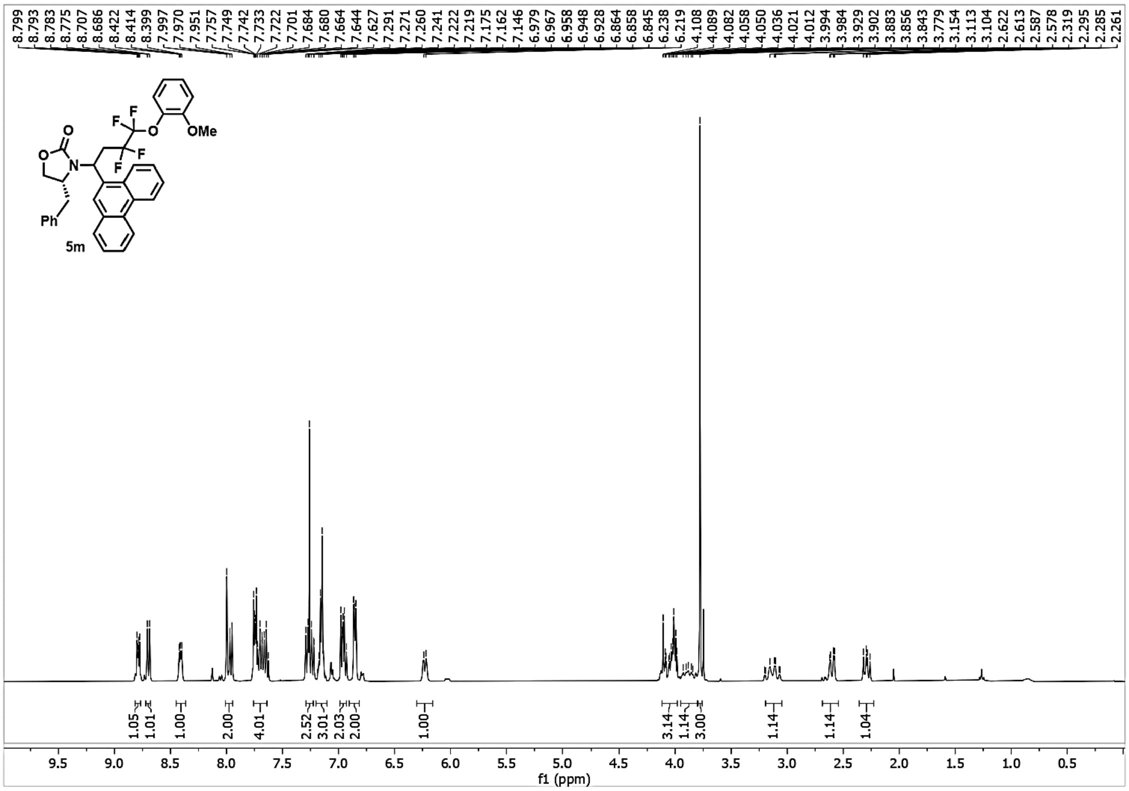

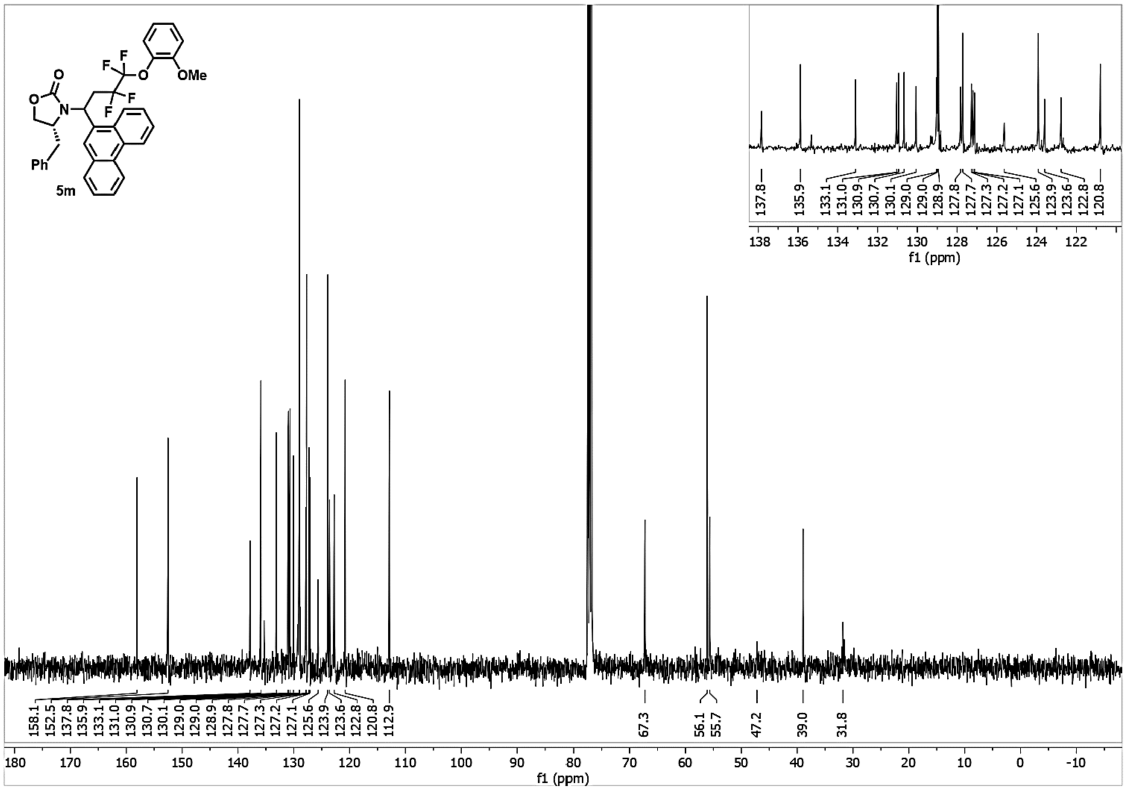

Compound 5m. ^{19}F NMR ($CDCl_3$, 376 MHz).

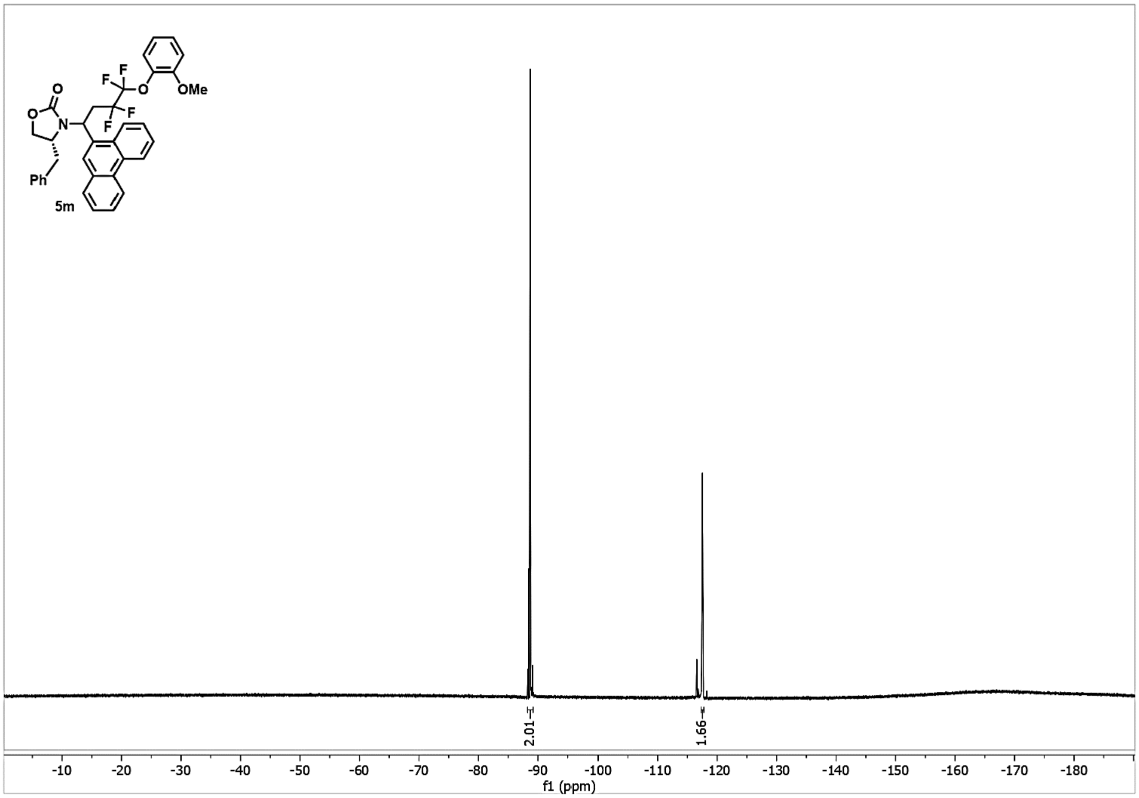

Compound 5n. Top: ^{1}H NMR ($CDCl_3$, 400 MHz). Bottom: ^{13}C NMR ($CDCl_3$, 100 MHz). Integrations shown for the major diastereoisomer.

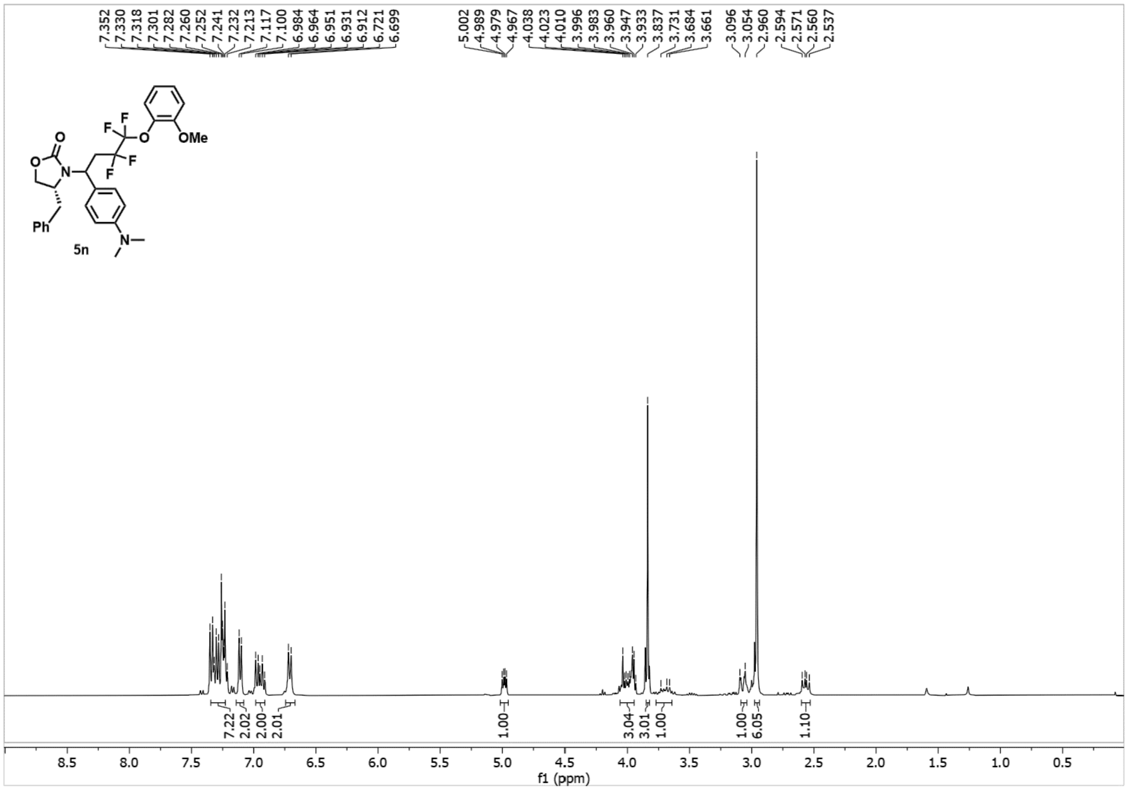

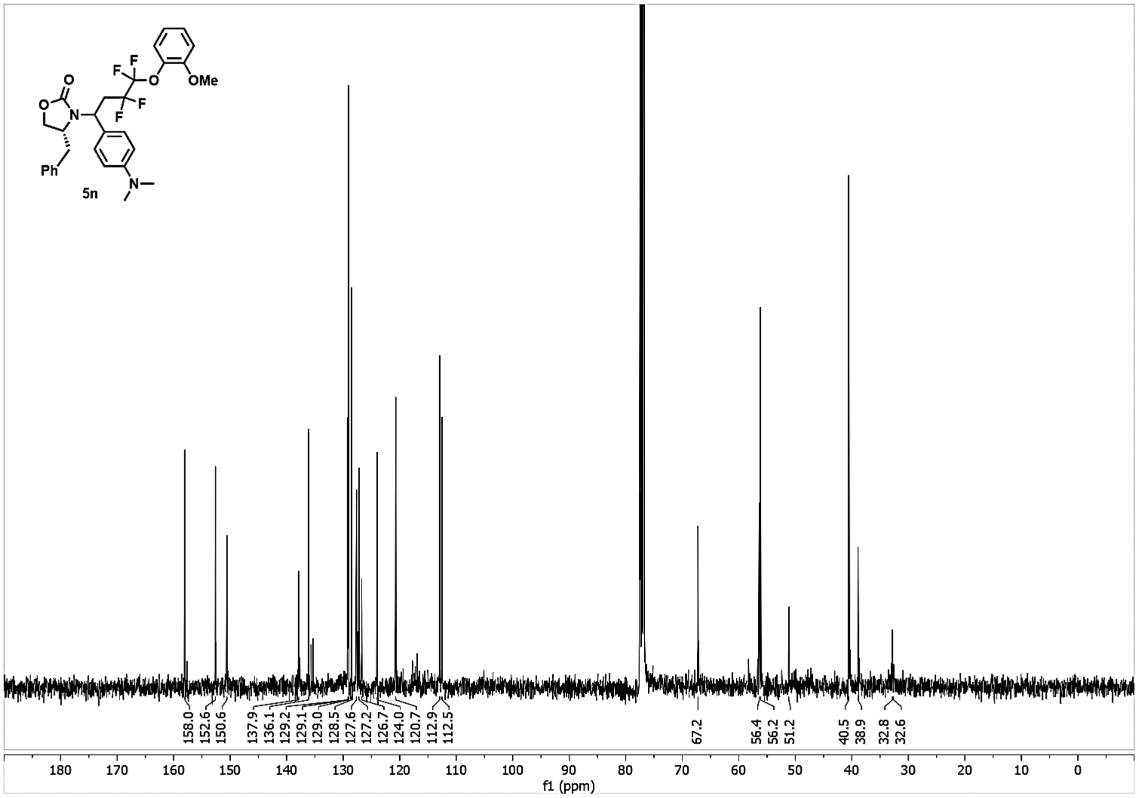

Compound 5n. ^{19}F NMR (CDCl$_3$, 376 MHz).

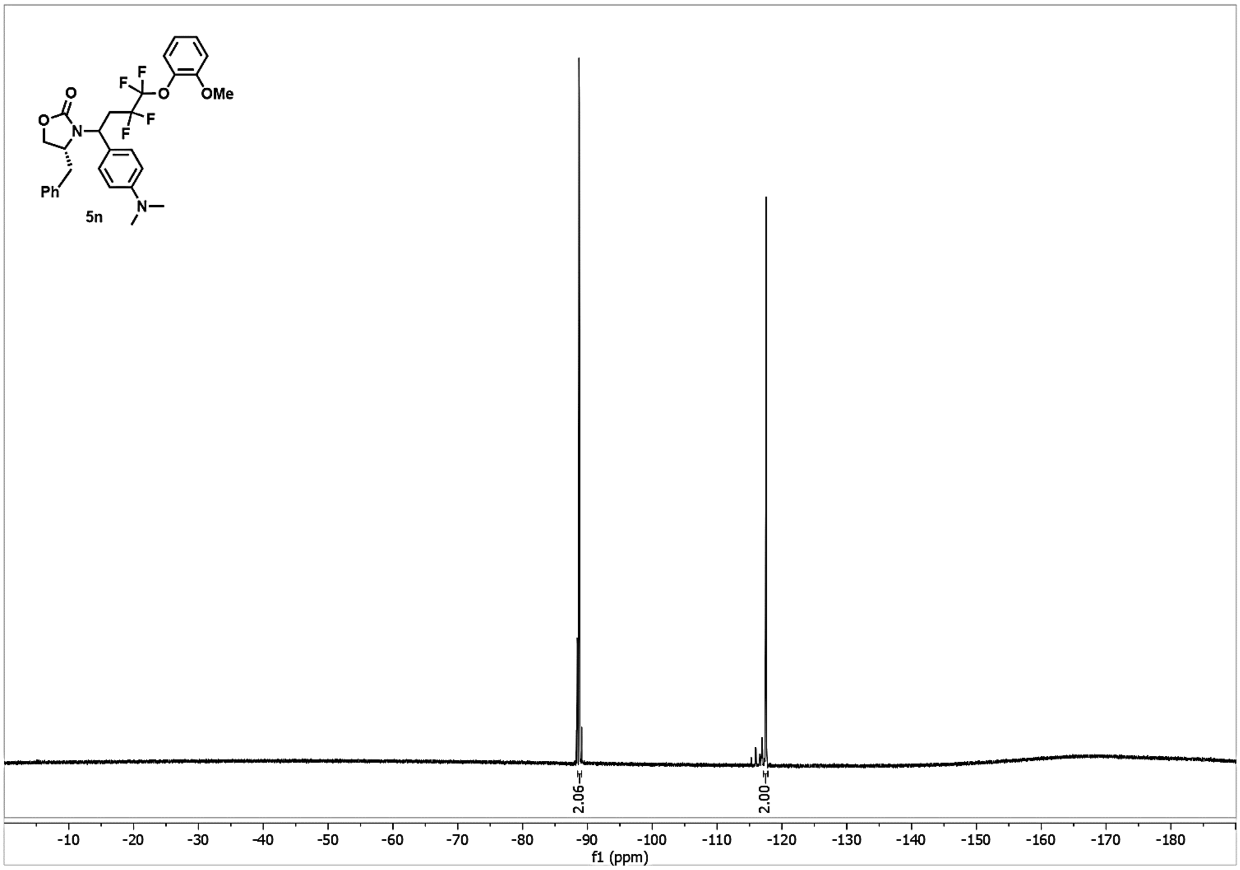

Compound 5o. Top: ^{1}H NMR ($CDCl_3$, 400 MHz). Bottom: ^{13}C NMR ($CDCl_3$, 100 MHz). Integrations shown for the major diastereoisomer.

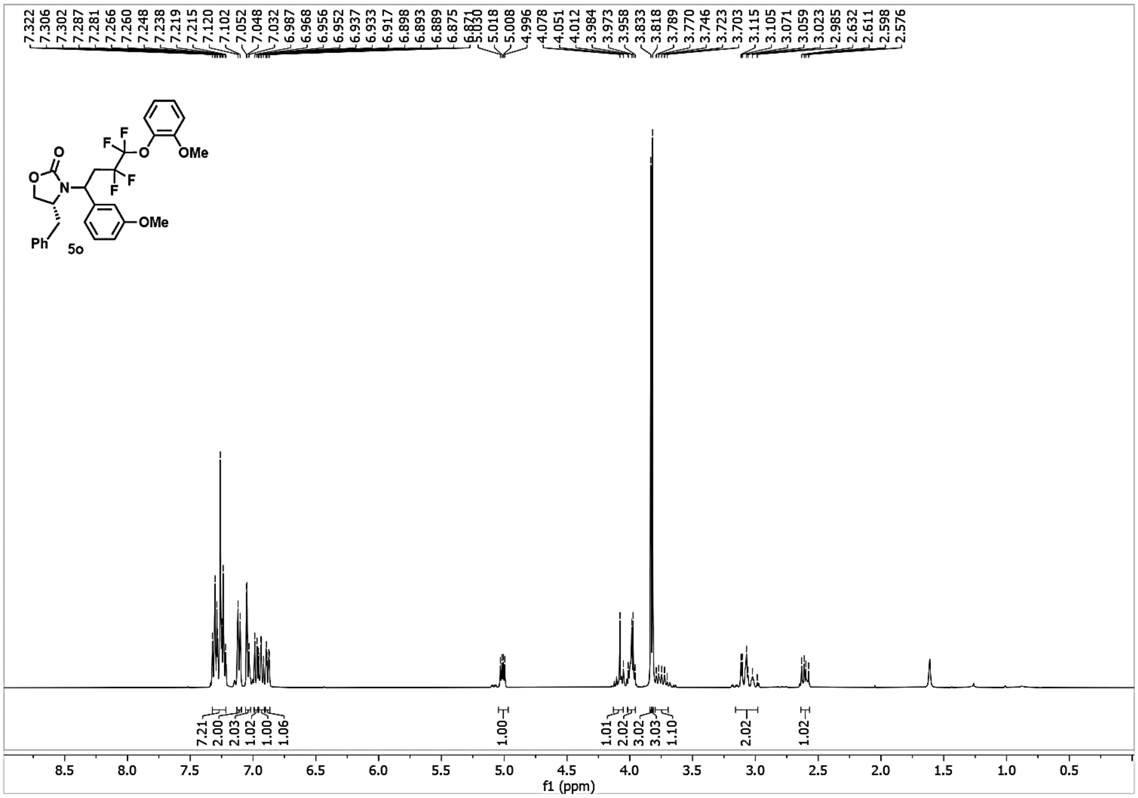

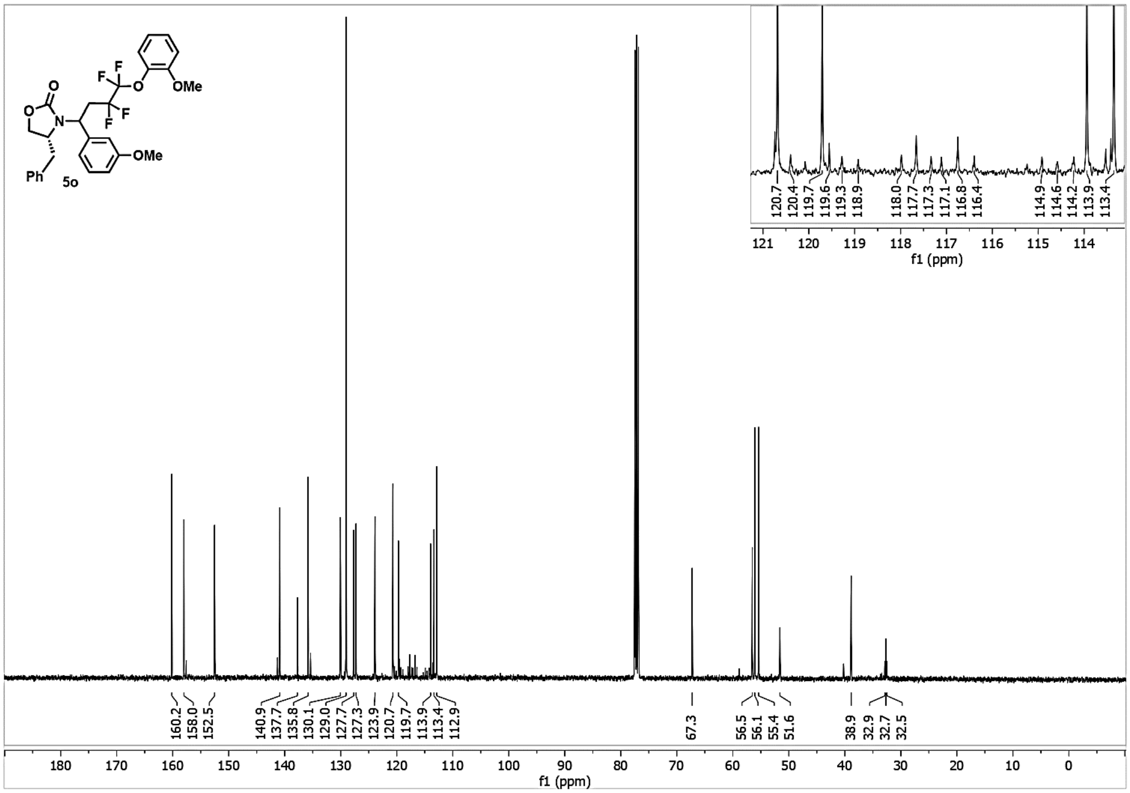

Compound 5o. ^{19}F NMR ($CDCl_3$, 376 MHz).

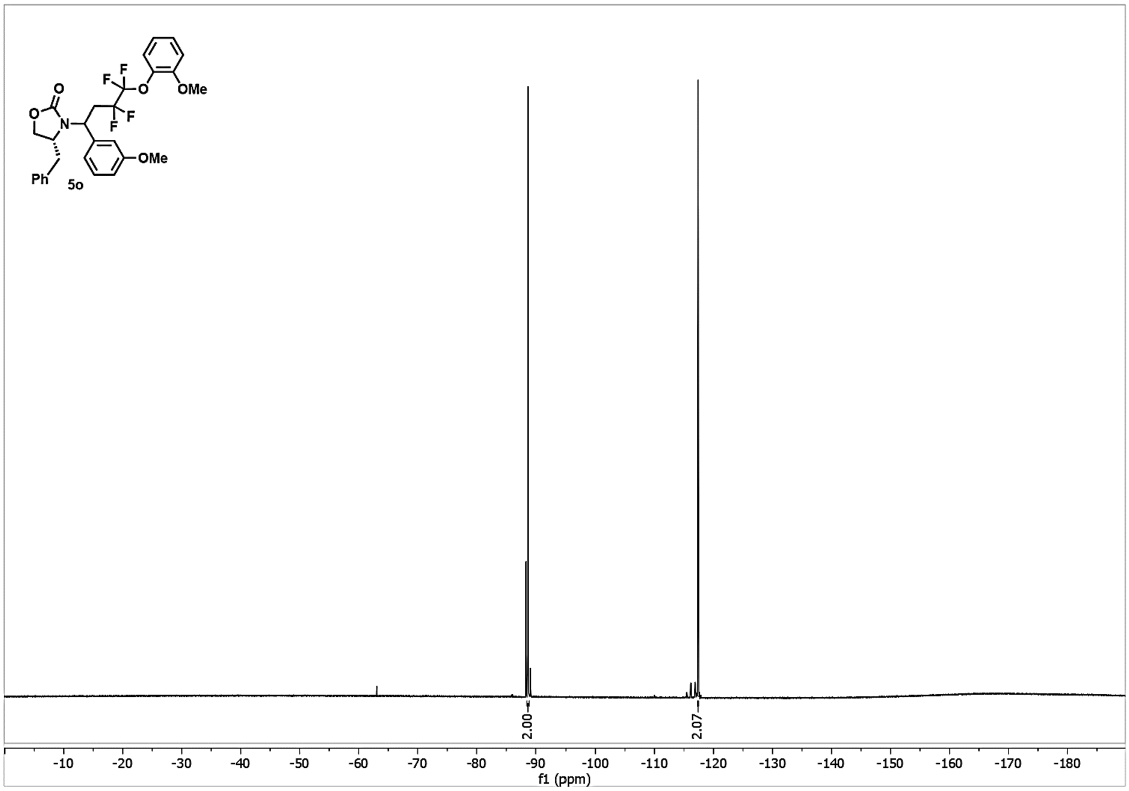

Compound 5p. Top: ^{1}H NMR ($CDCl_3$, 400 MHz). Bottom: ^{13}C NMR ($CDCl_3$, 100 MHz).

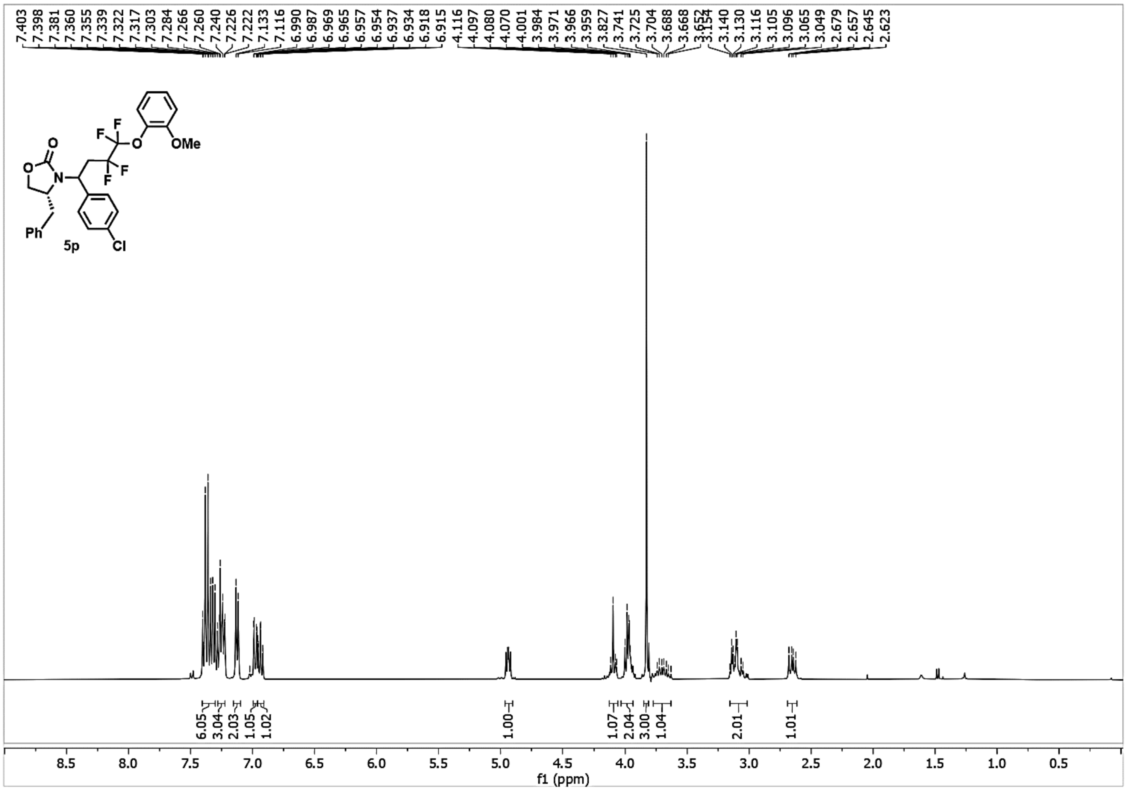

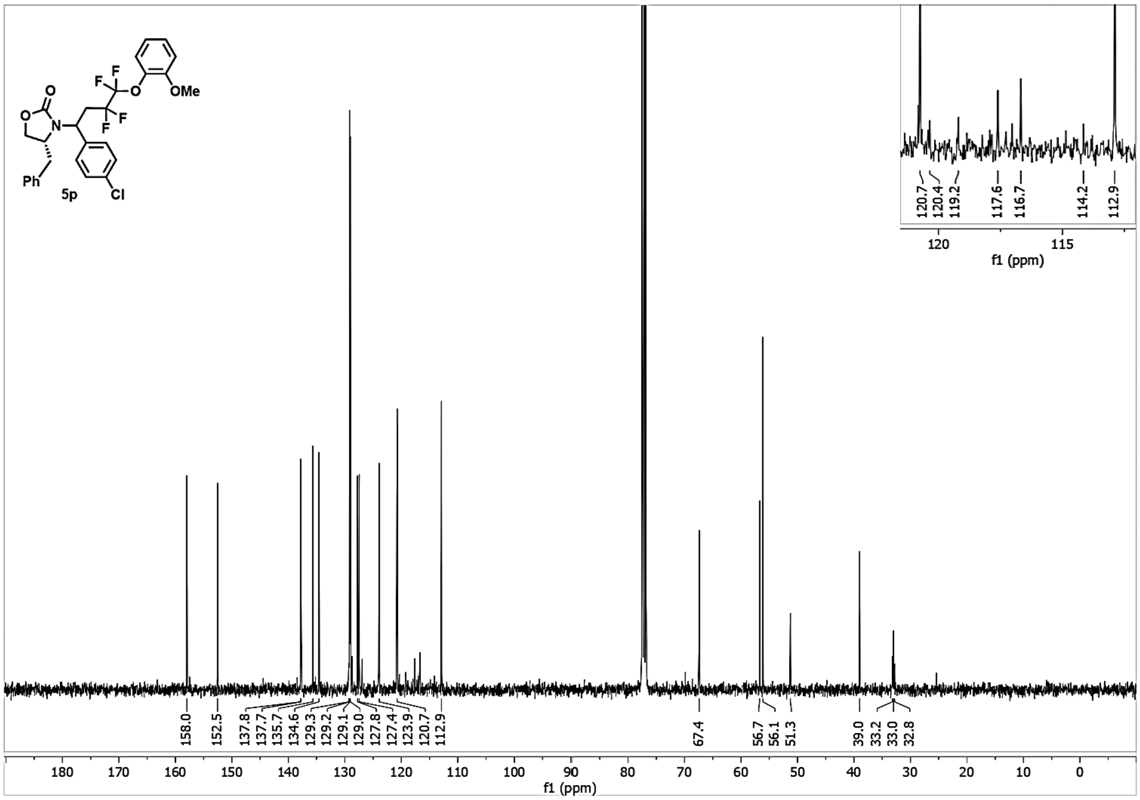

Compound 5p. ^{19}F NMR ($CDCl_3$, 376 MHz).

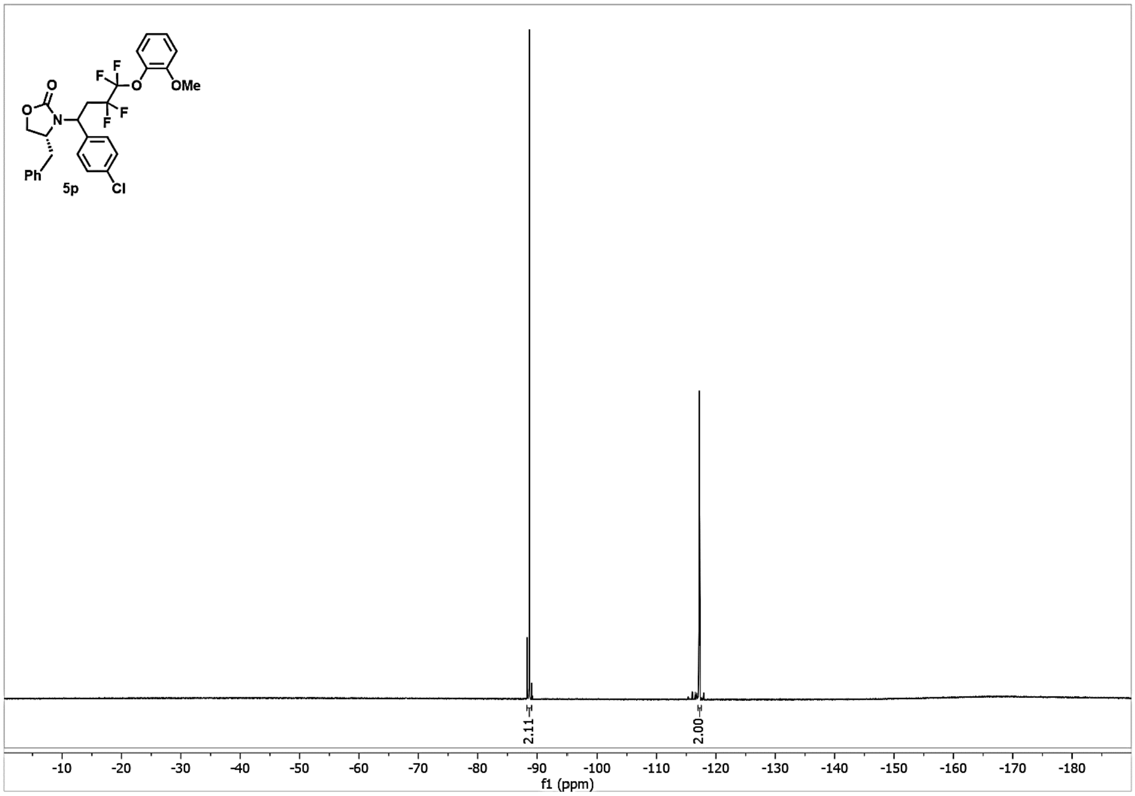

Compound 5q. Top: ^1H NMR ($CDCl_3$, 400 MHz). Bottom: ^{13}C NMR ($CDCl_3$, 100 MHz).

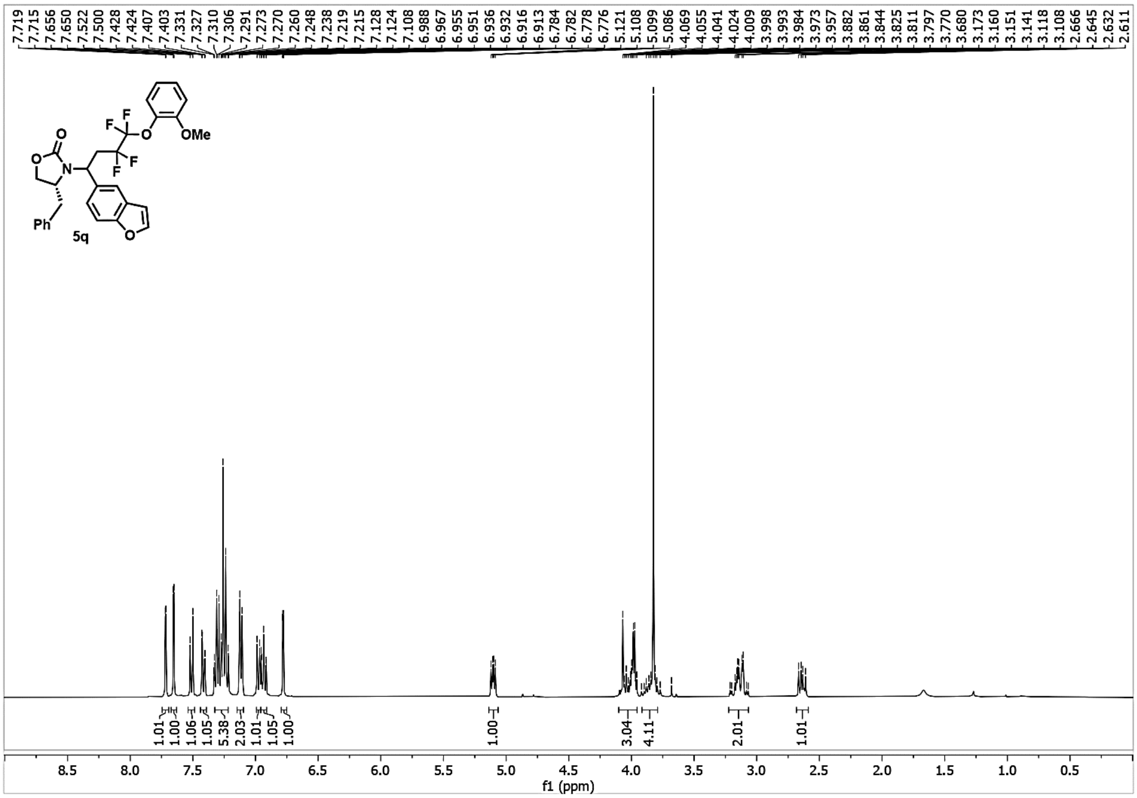

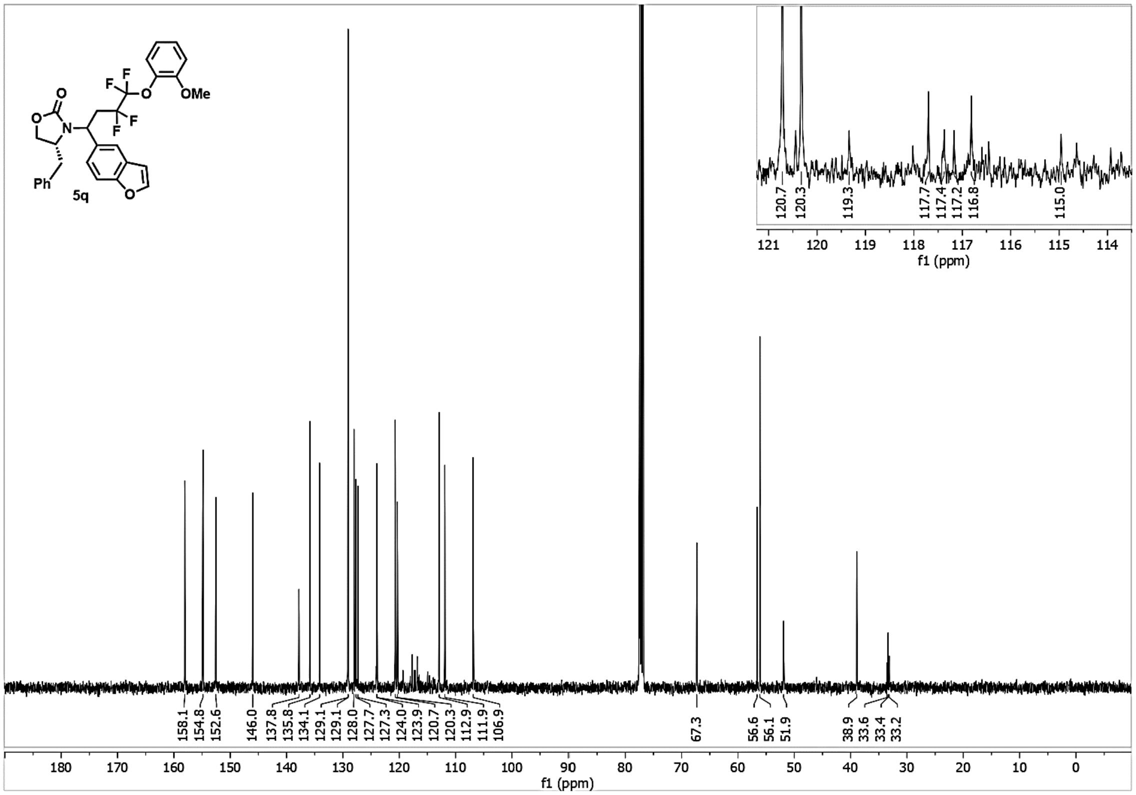

Compound 5q. ^{19}F NMR ($CDCl_3$, 376 MHz).

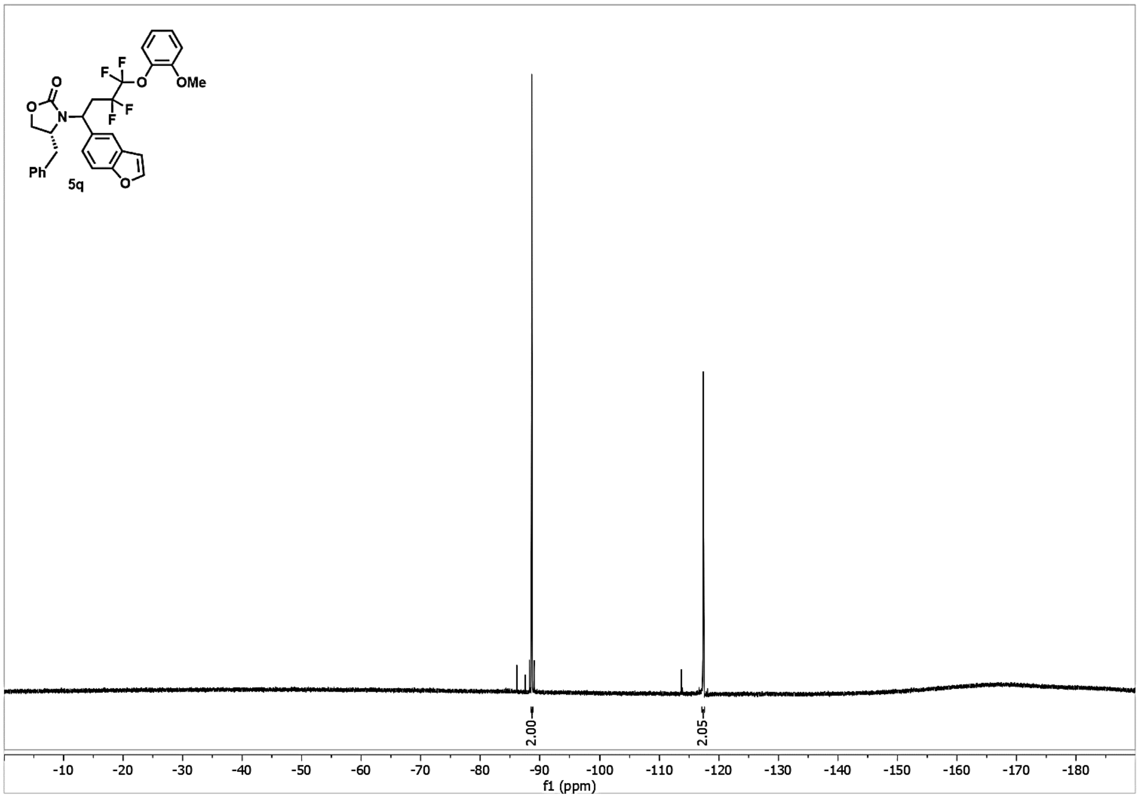

Compound 5r. Top: ^1H NMR ($CDCl_3$, 400 MHz). Bottom: ^{13}C NMR ($CDCl_3$, 100 MHz).

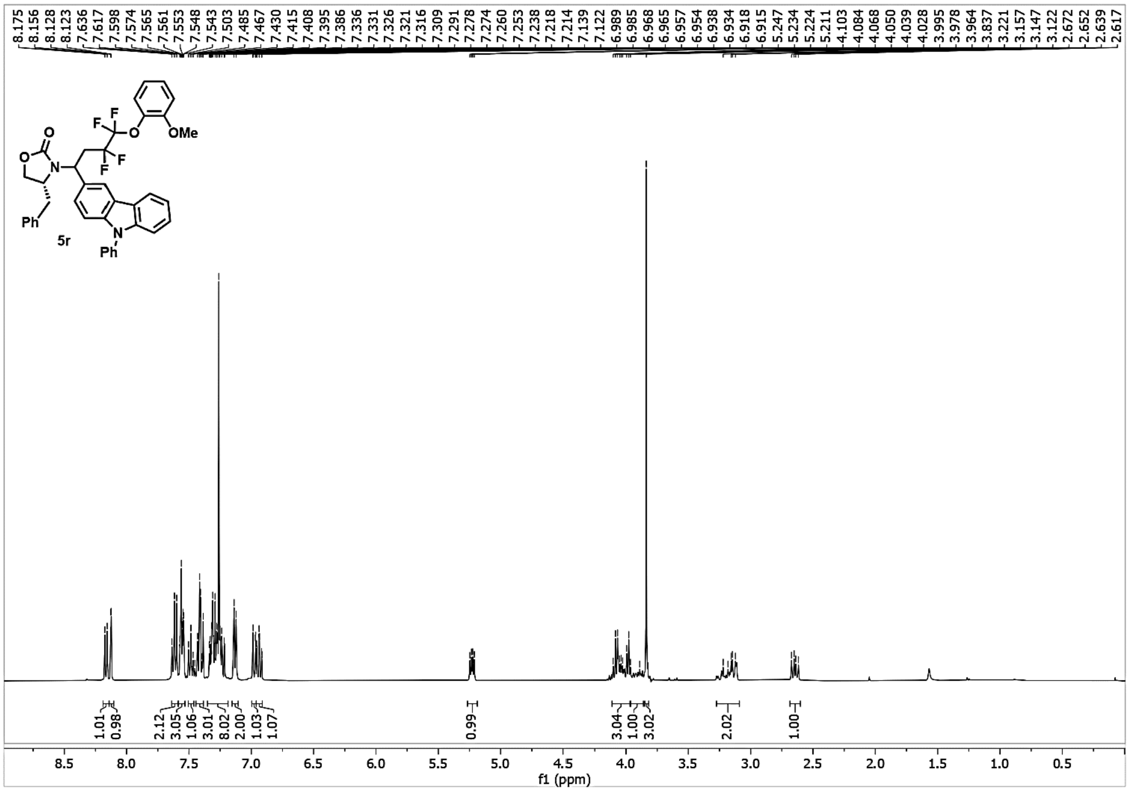

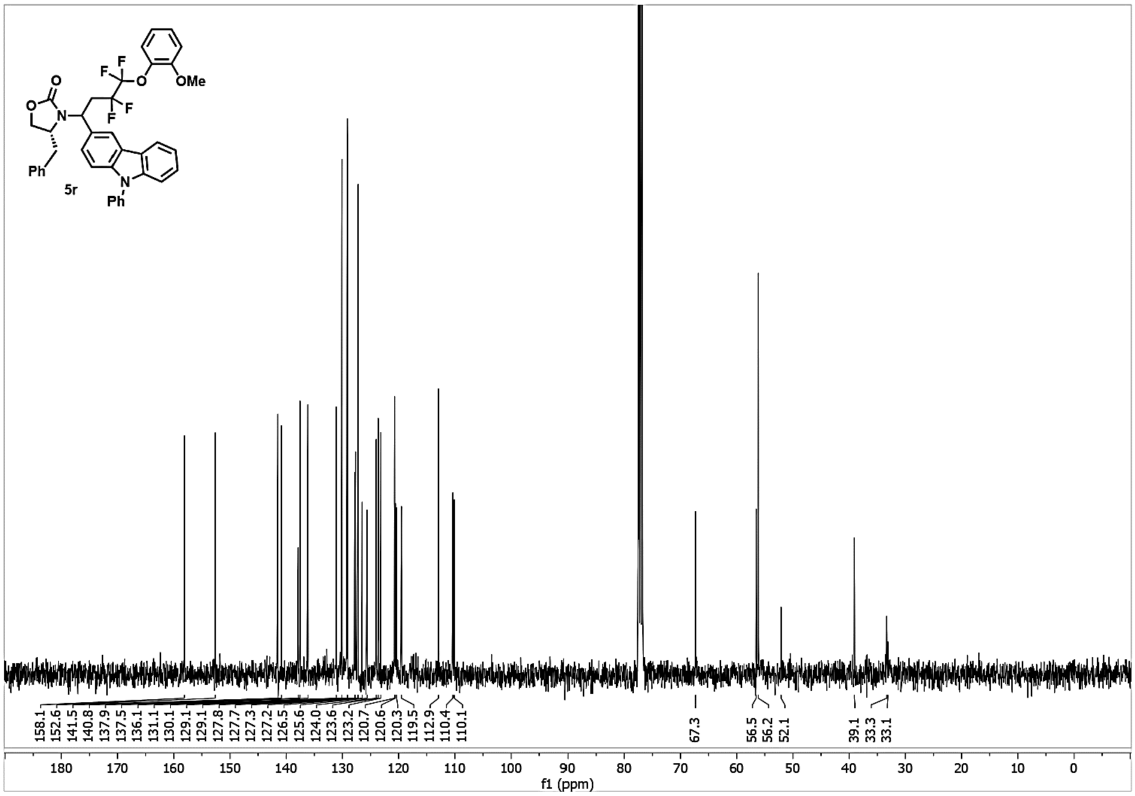

Compound 5r. ^{19}F NMR ($CDCl_3$, 376 MHz).

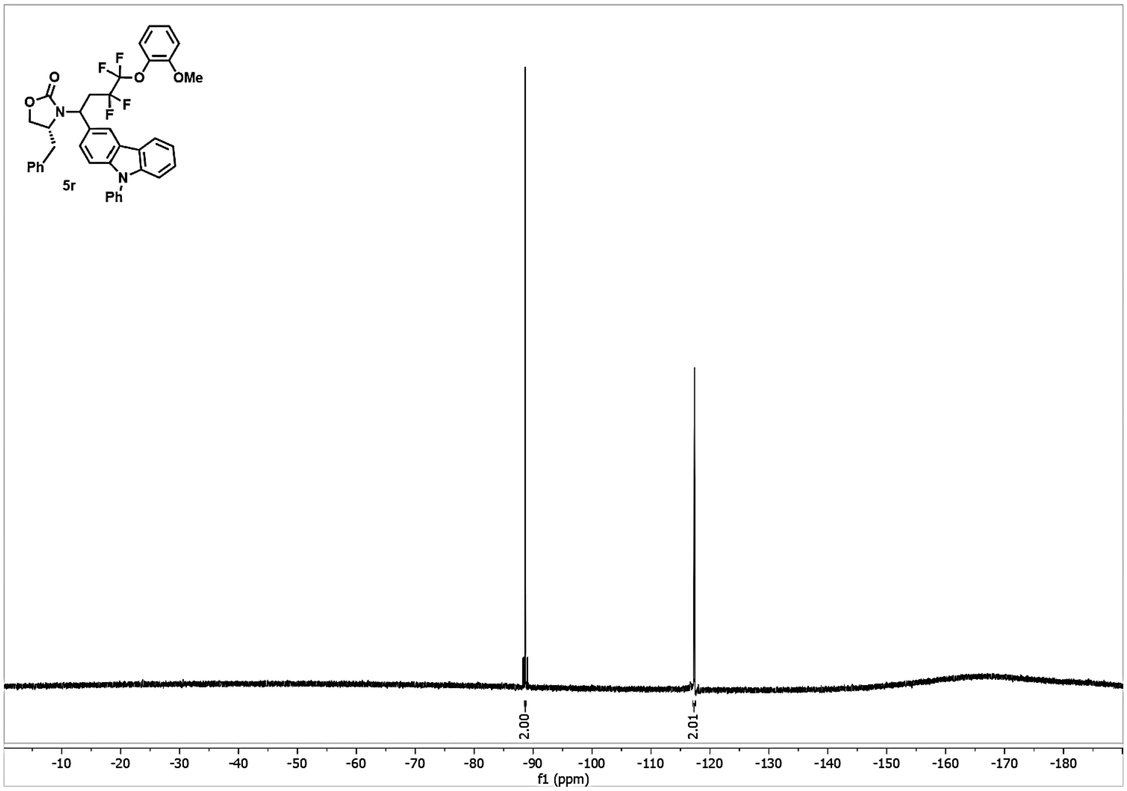

Compound 5s. Top: ^{1}H NMR ($CDCl_3$, 400 MHz). Bottom: ^{13}C NMR ($CDCl_3$, 100 MHz).

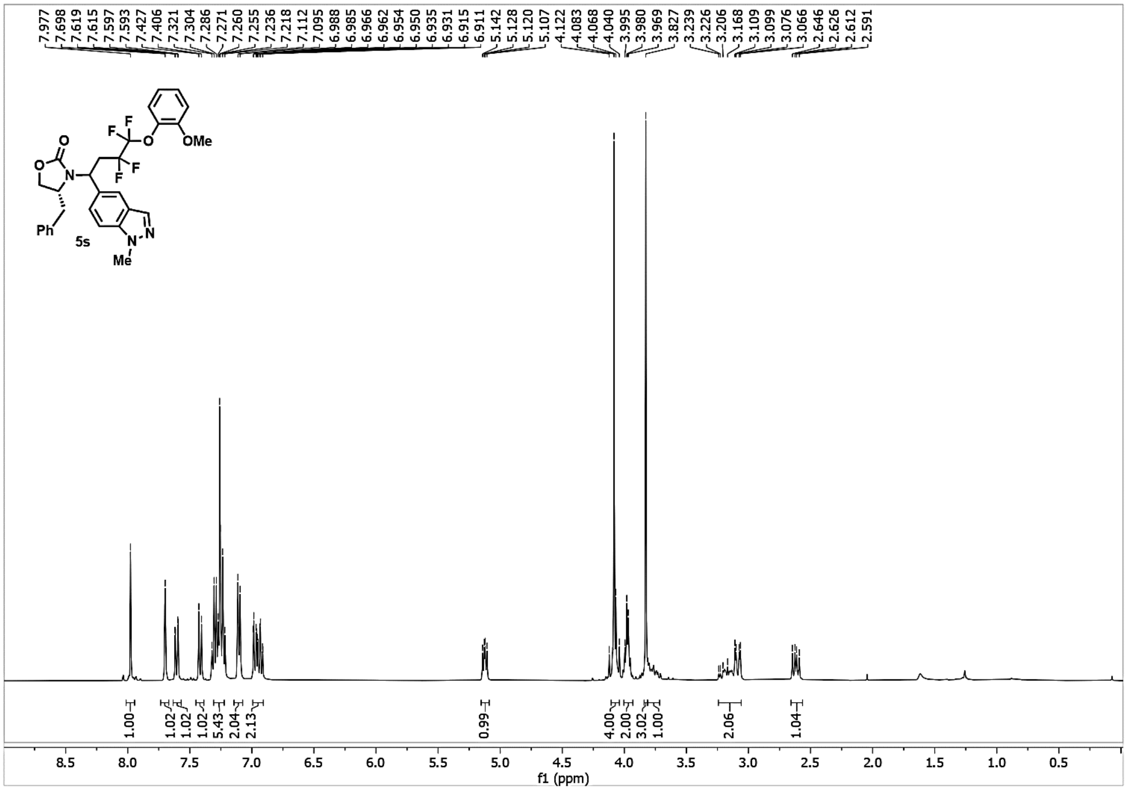

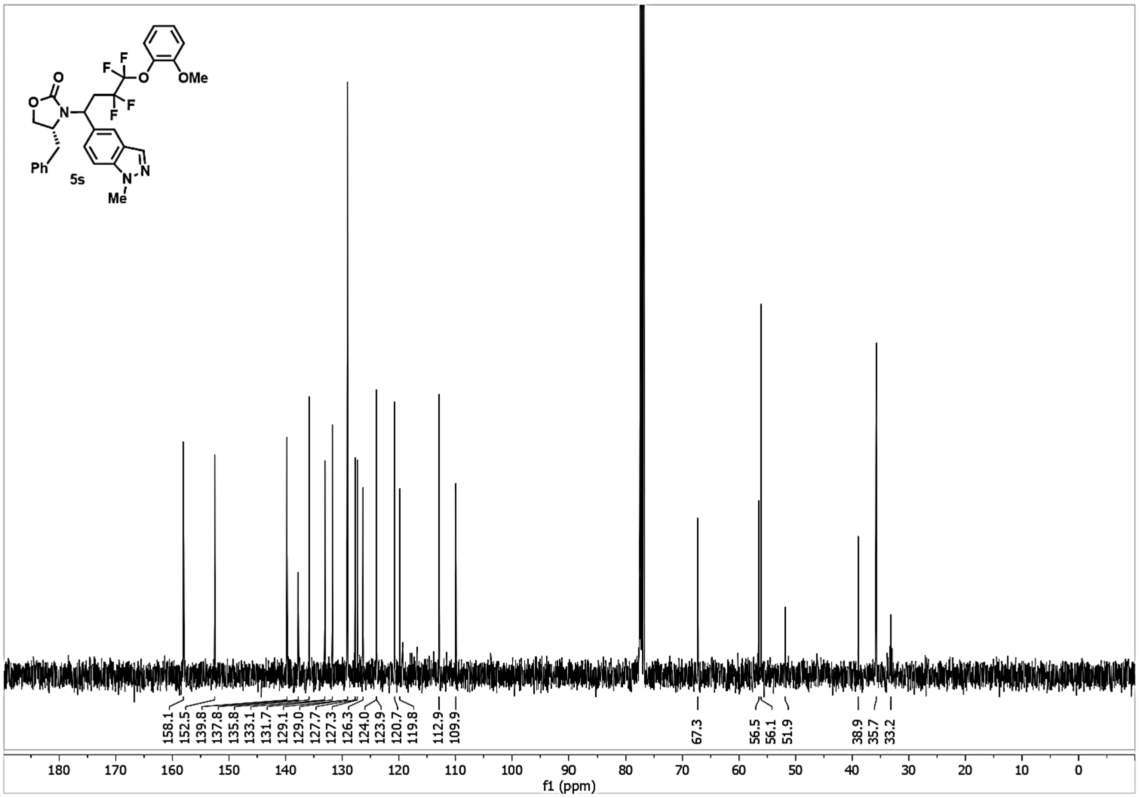

Compound 5s. ^{19}F NMR ($CDCl_3$, 376 MHz).

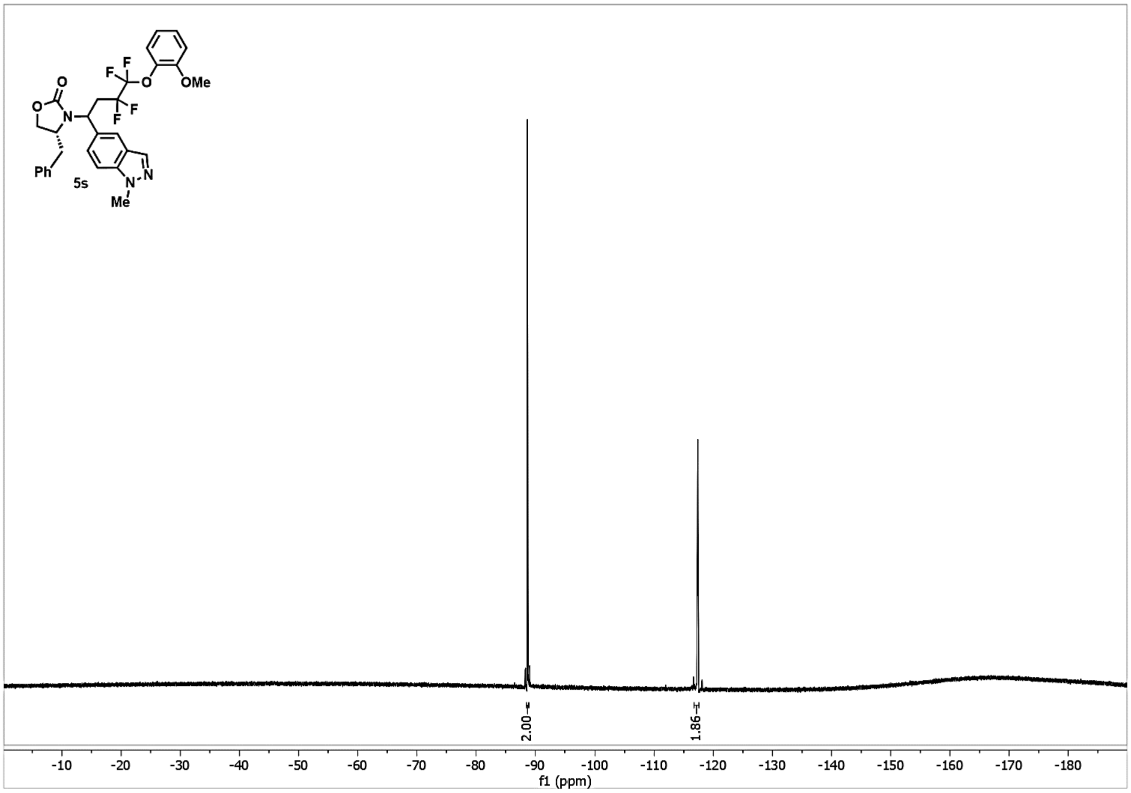

Compound 5t. Top: ^{1}H NMR ($CDCl_3$, 400 MHz). Bottom: ^{13}C NMR ($CDCl_3$, 100 MHz).

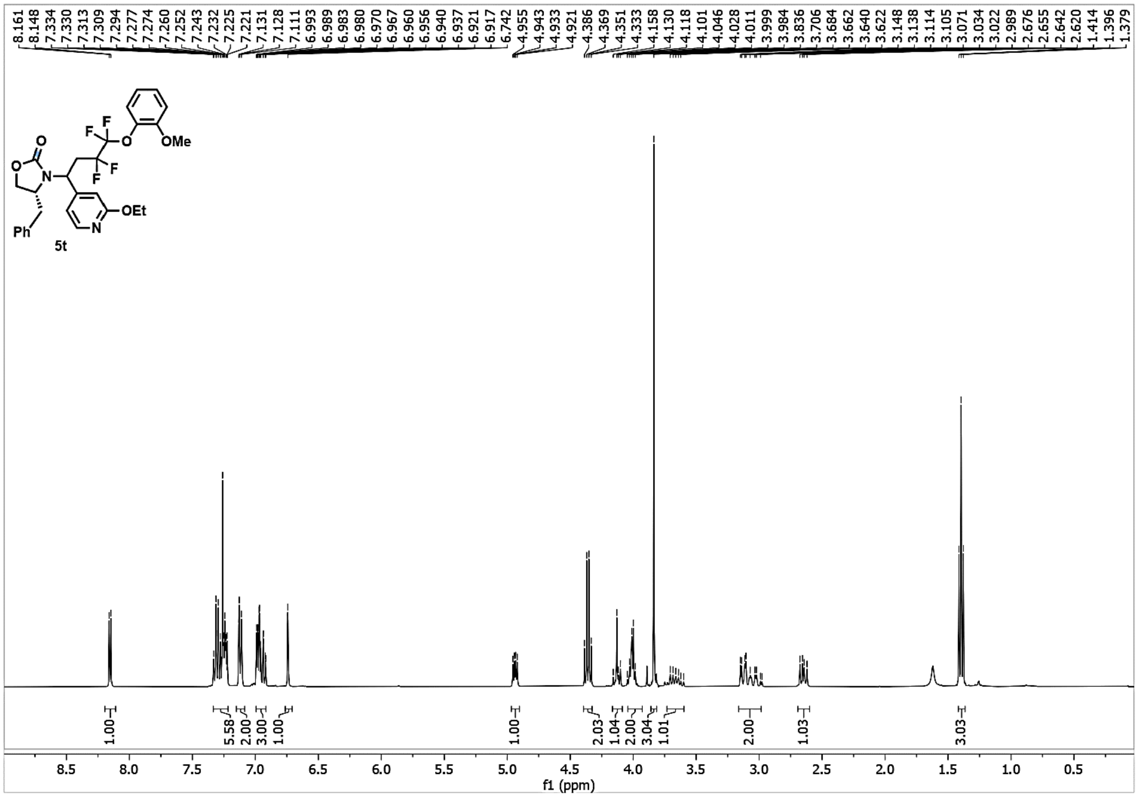

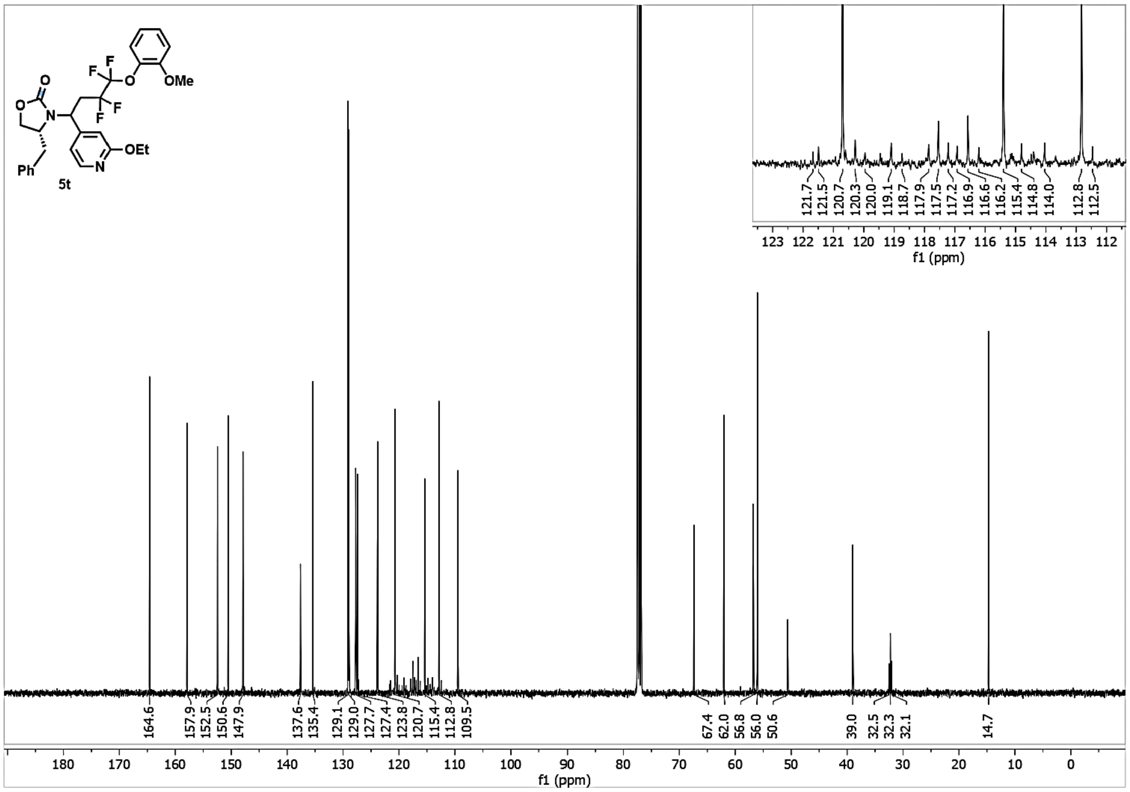

Compound 5t. ^{19}F NMR ($CDCl_3$, 376 MHz).

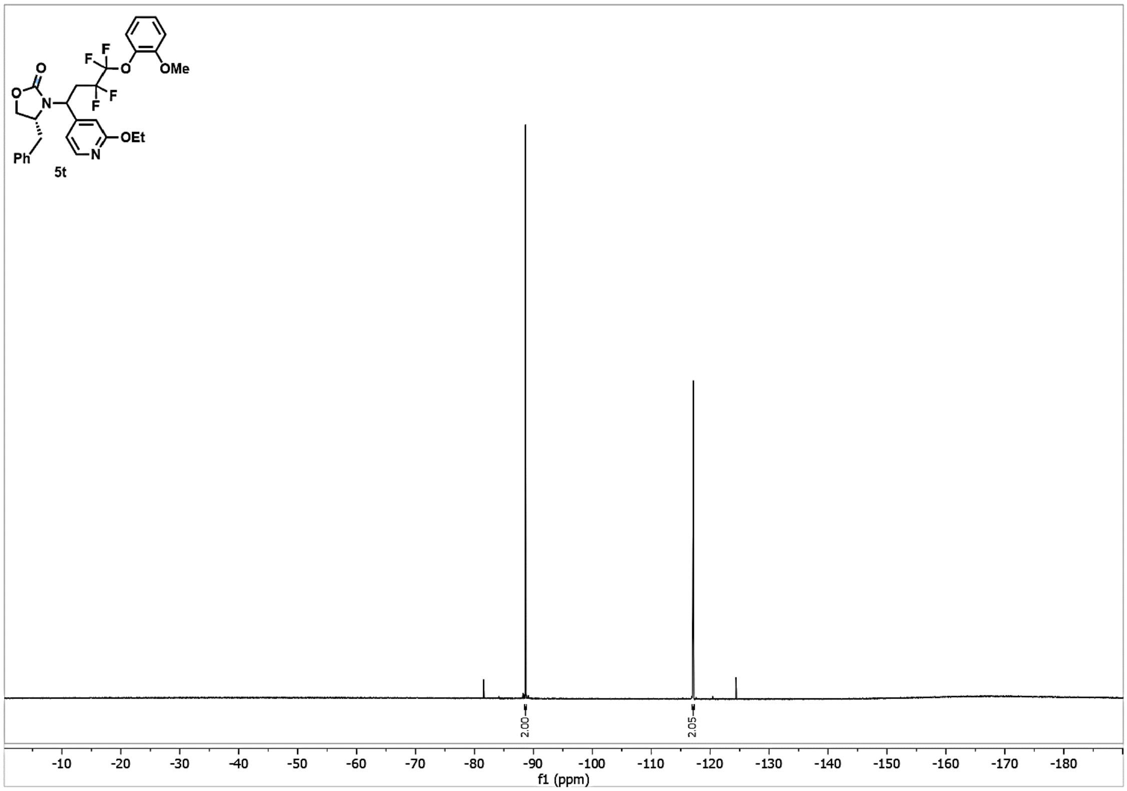

Compound 6a. Top: ^{1}H NMR ($CDCl_3$, 400 MHz). Bottom: ^{13}C NMR ($CDCl_3$, 100 MHz).

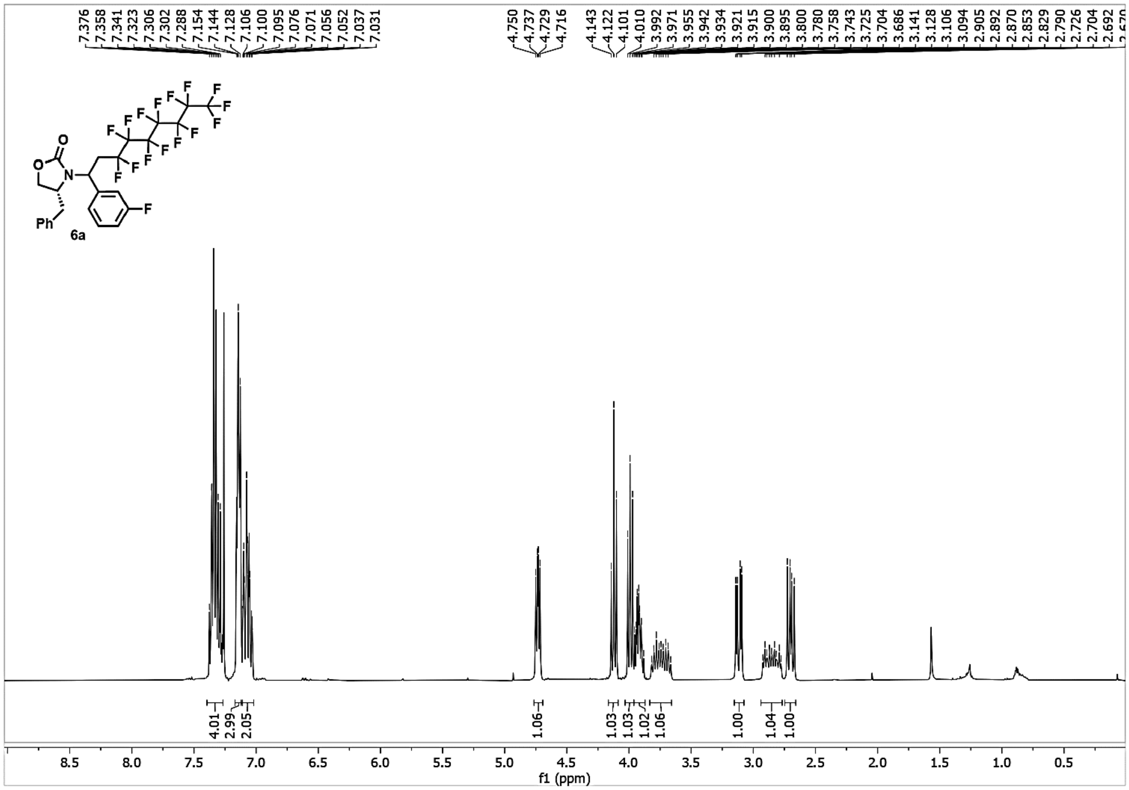

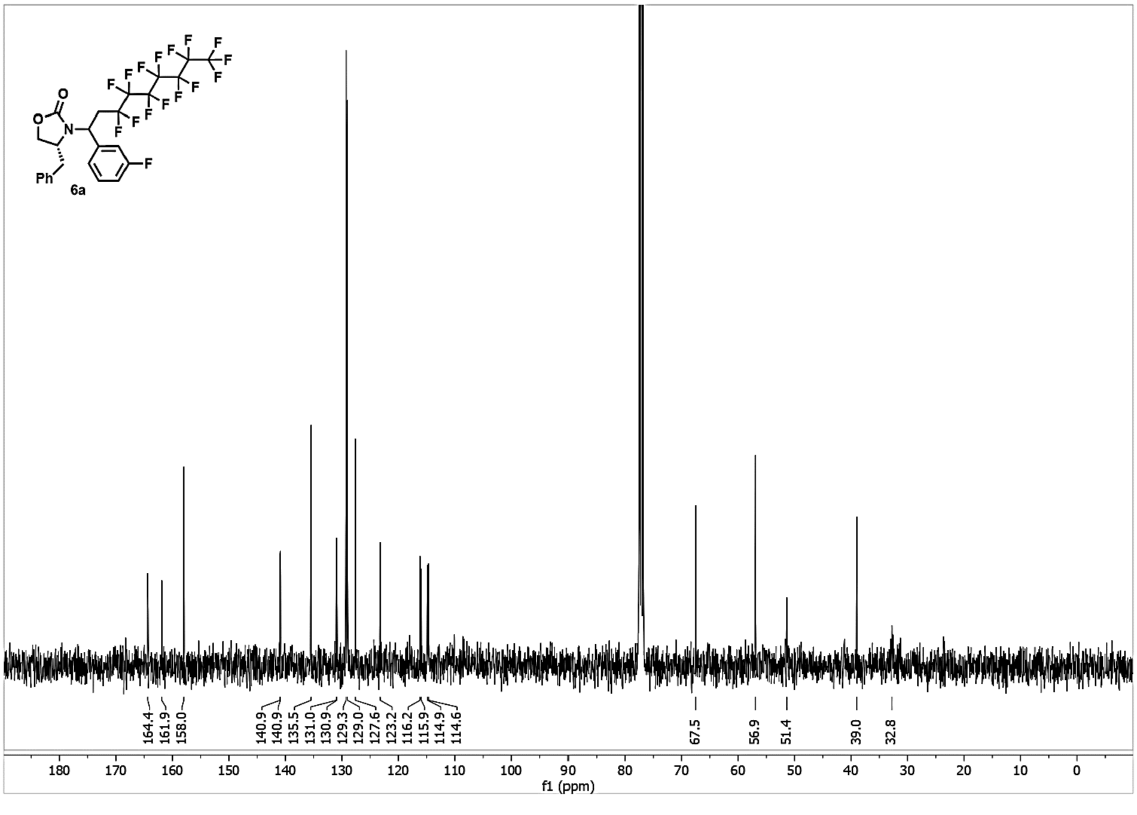

Compound 6a. ^{19}F NMR ($CDCl_3$, 376 MHz).

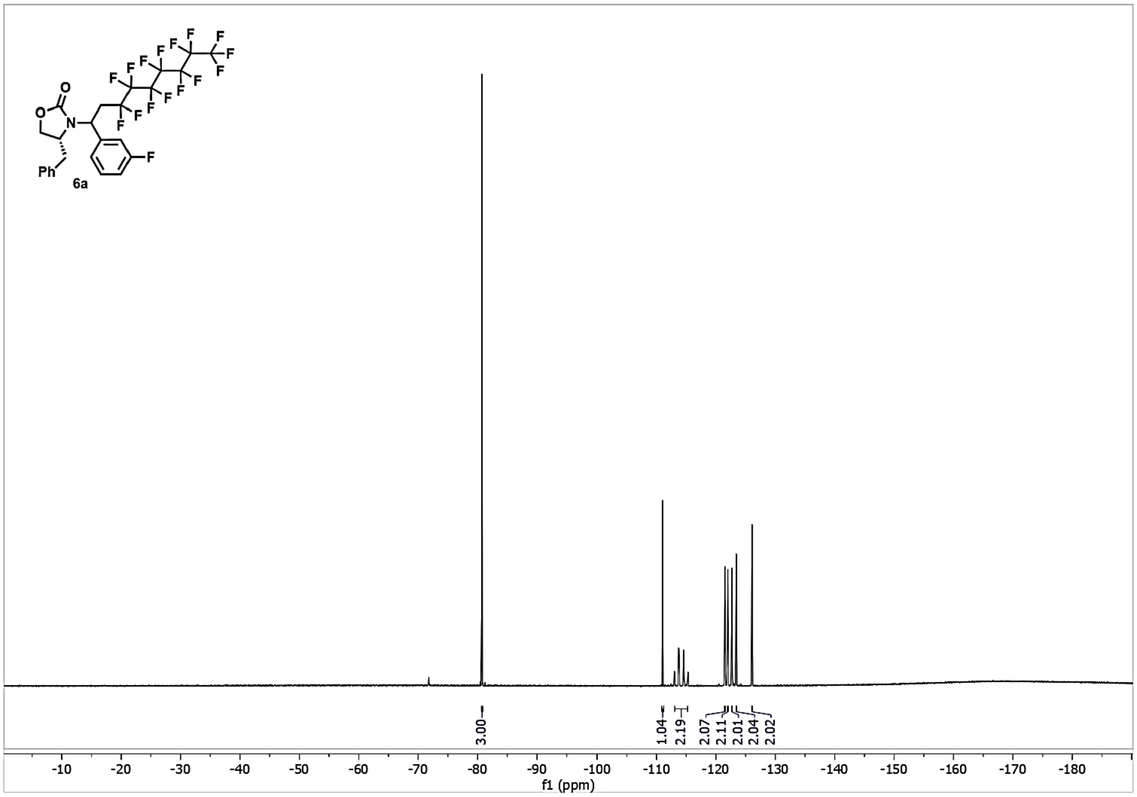

Compound 6b. Top: ^1H NMR ($CDCl_3$, 400 MHz). Bottom: ^{13}C NMR ($CDCl_3$, 100 MHz).

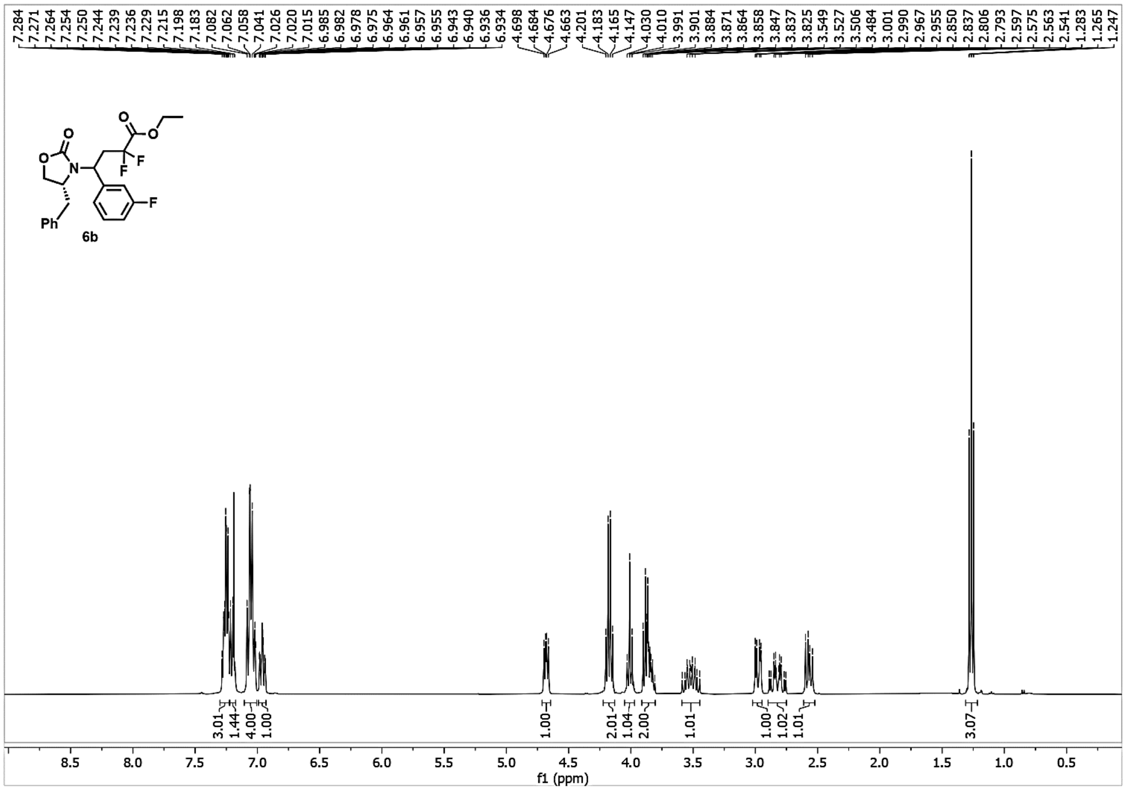

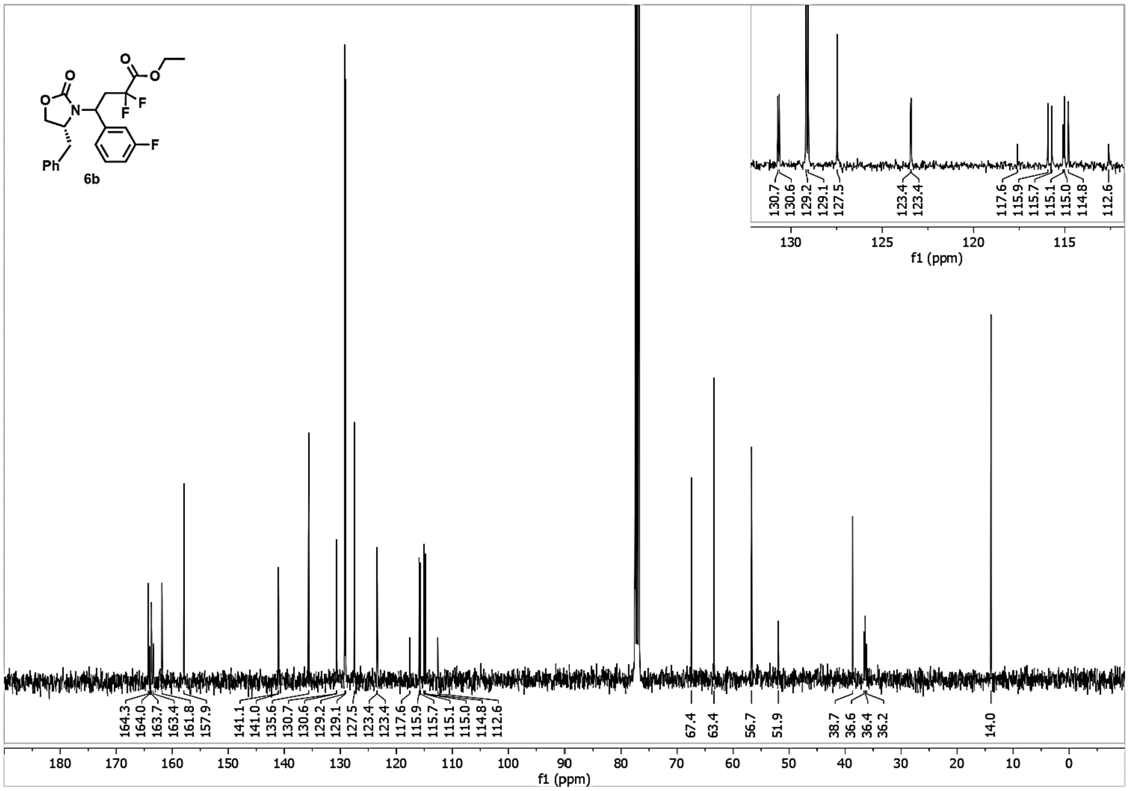

Compound 6b. ^{19}F NMR ($CDCl_3$, 376 MHz).

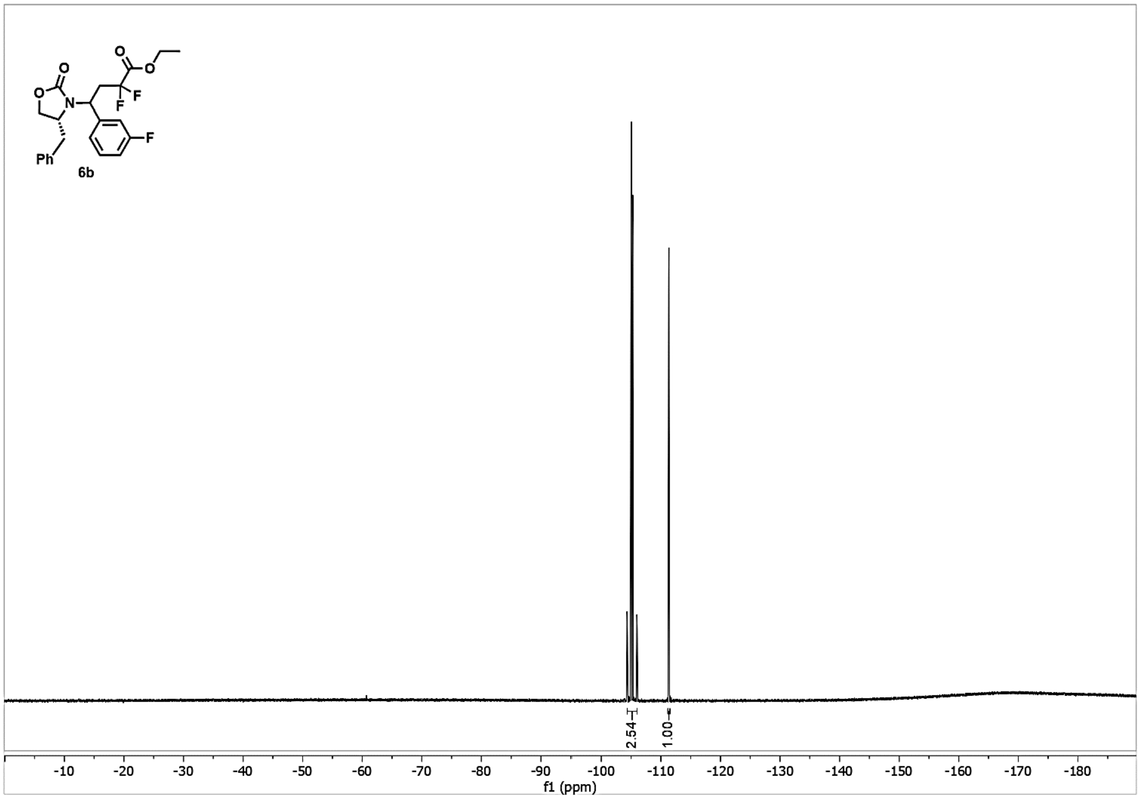

Compound 6c. Top: ^{1}H NMR ($CDCl_3$, 400 MHz). Bottom: ^{13}C NMR ($CDCl_3$, 100 MHz).

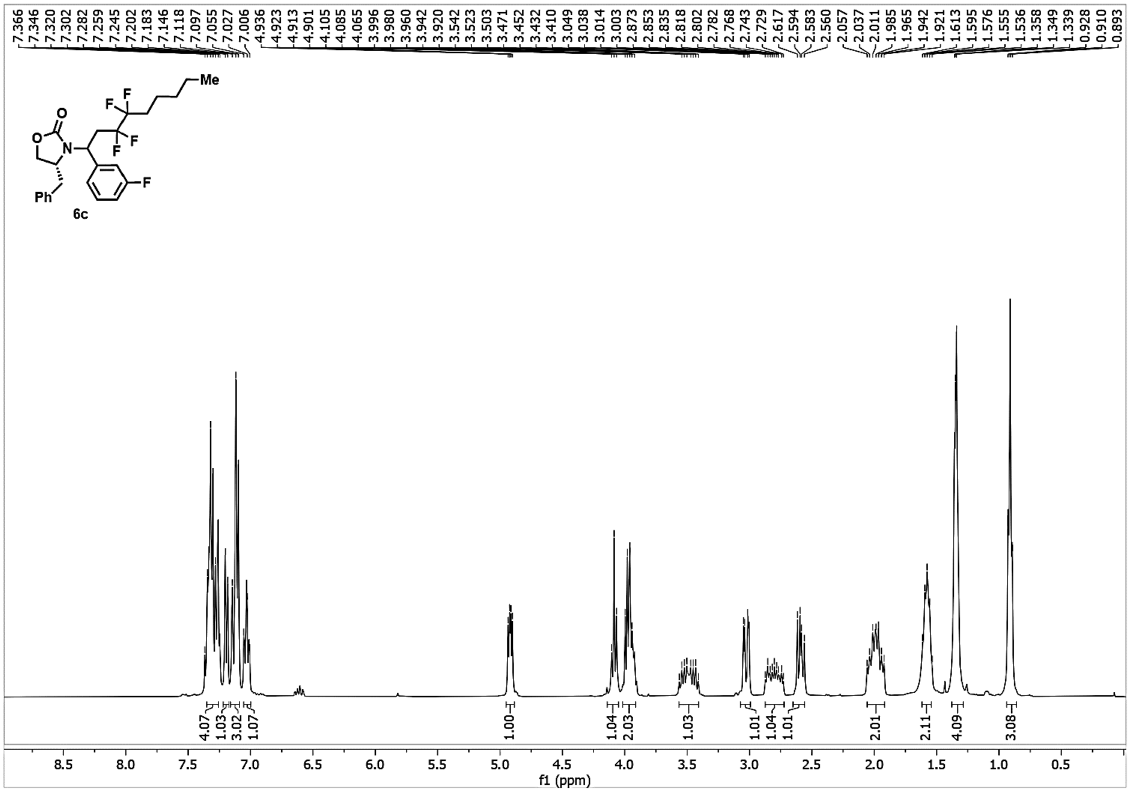

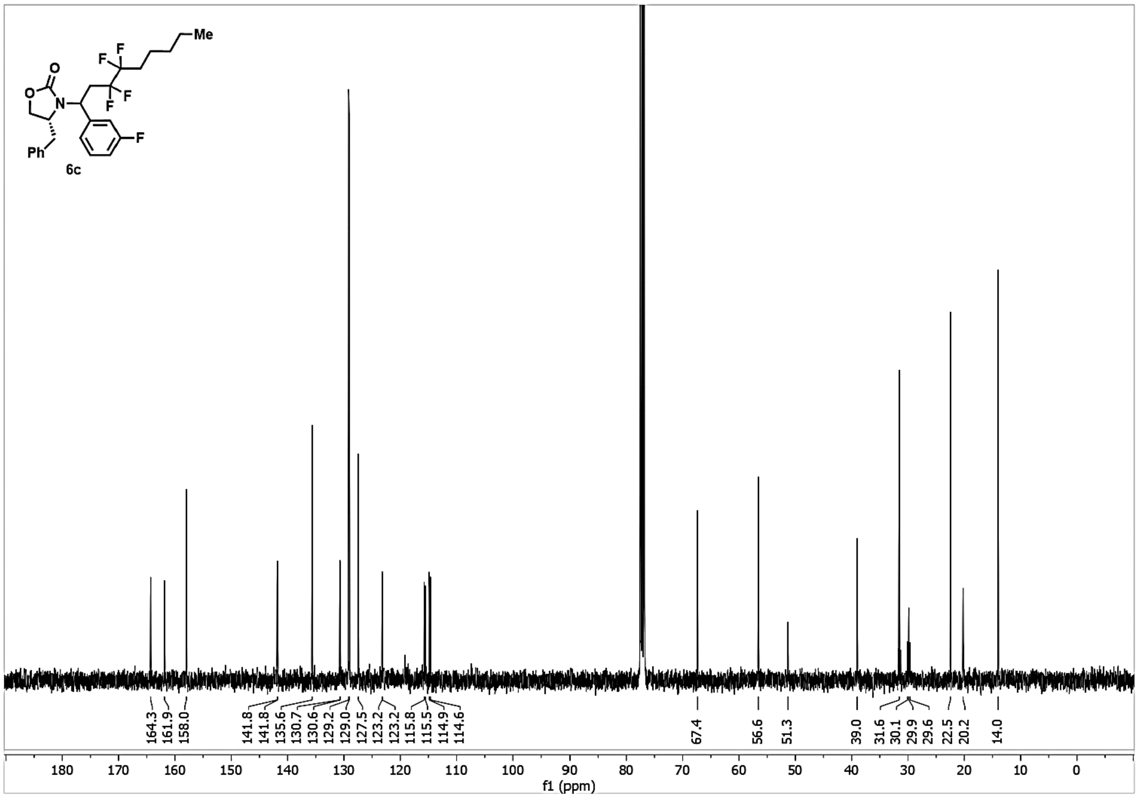

Compound 6c. ^{19}F NMR ($CDCl_3$, 376 MHz).

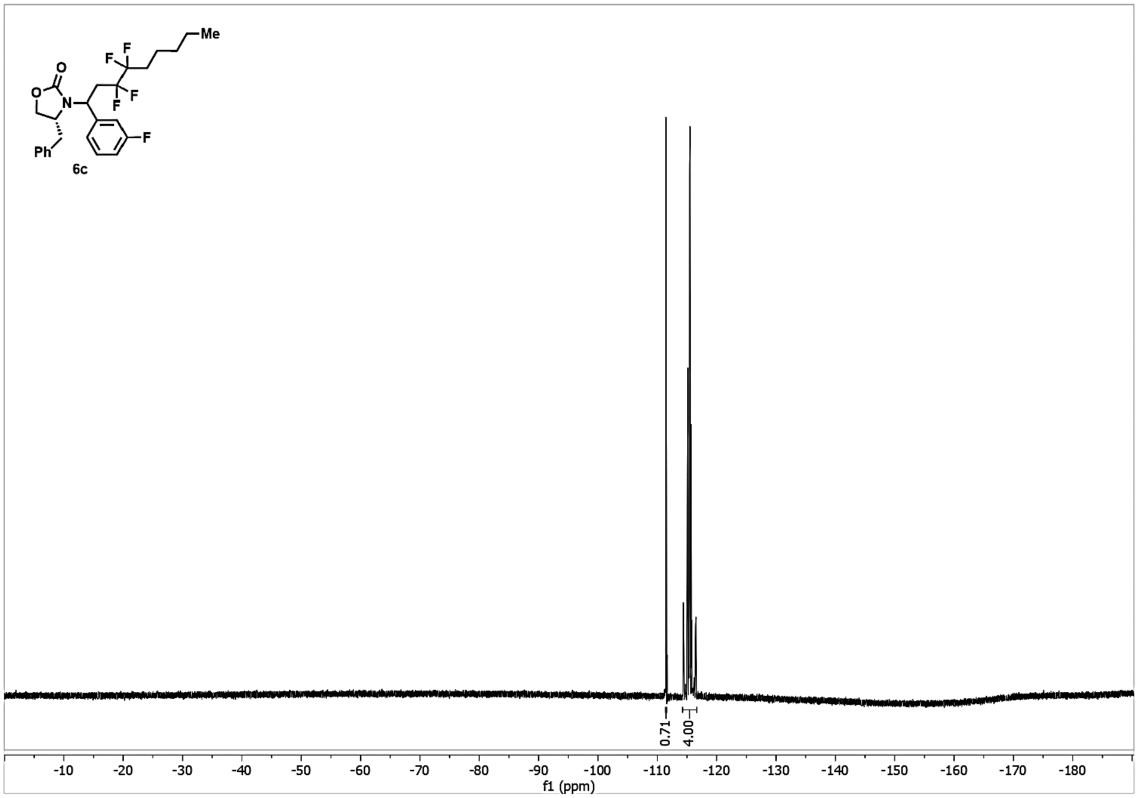

Compound 6d. Top: ^{1}H NMR ($CDCl_3$, 400 MHz). Bottom: ^{13}C NMR ($CDCl_3$, 100 MHz).

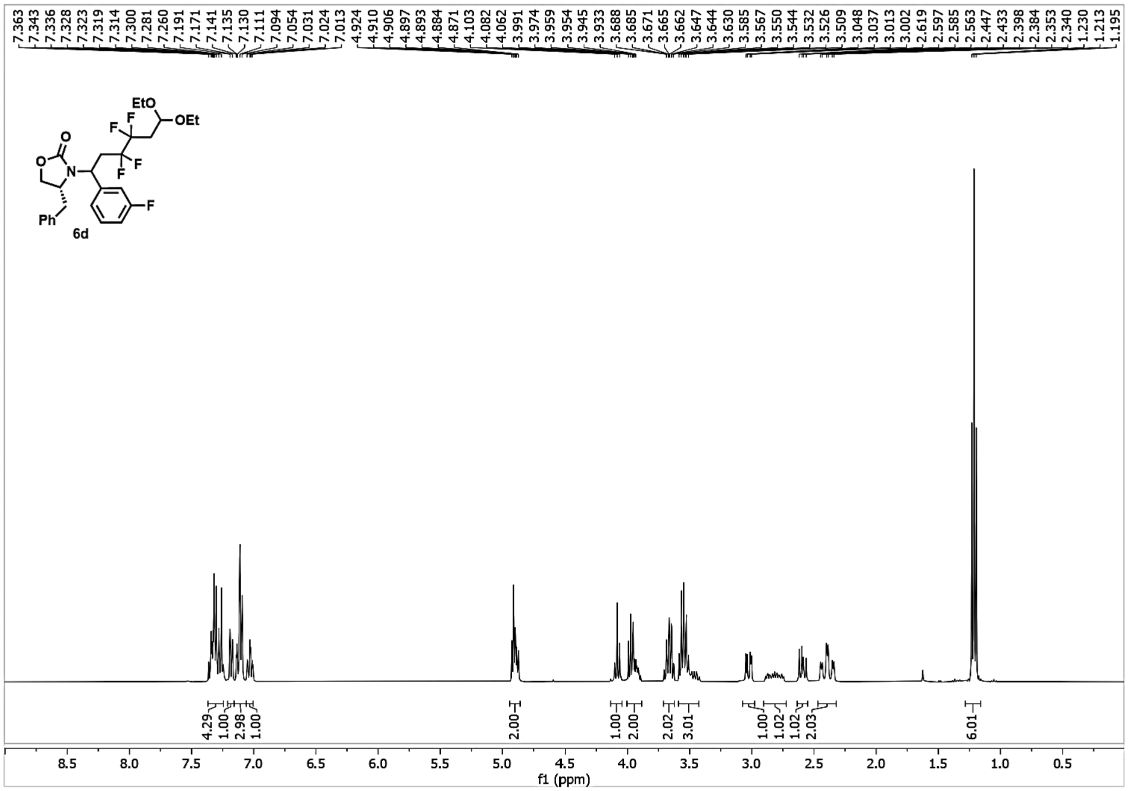

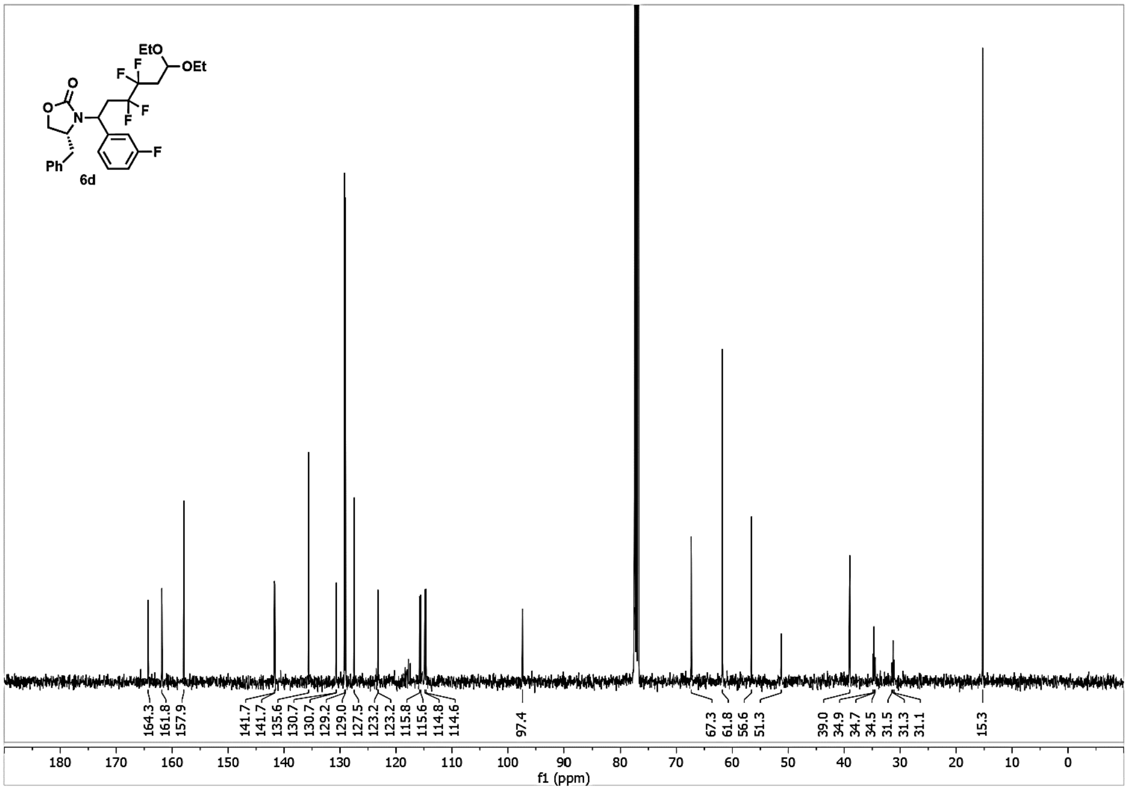

Compound 6d. ^{19}F NMR ($CDCl_3$, 376 MHz).

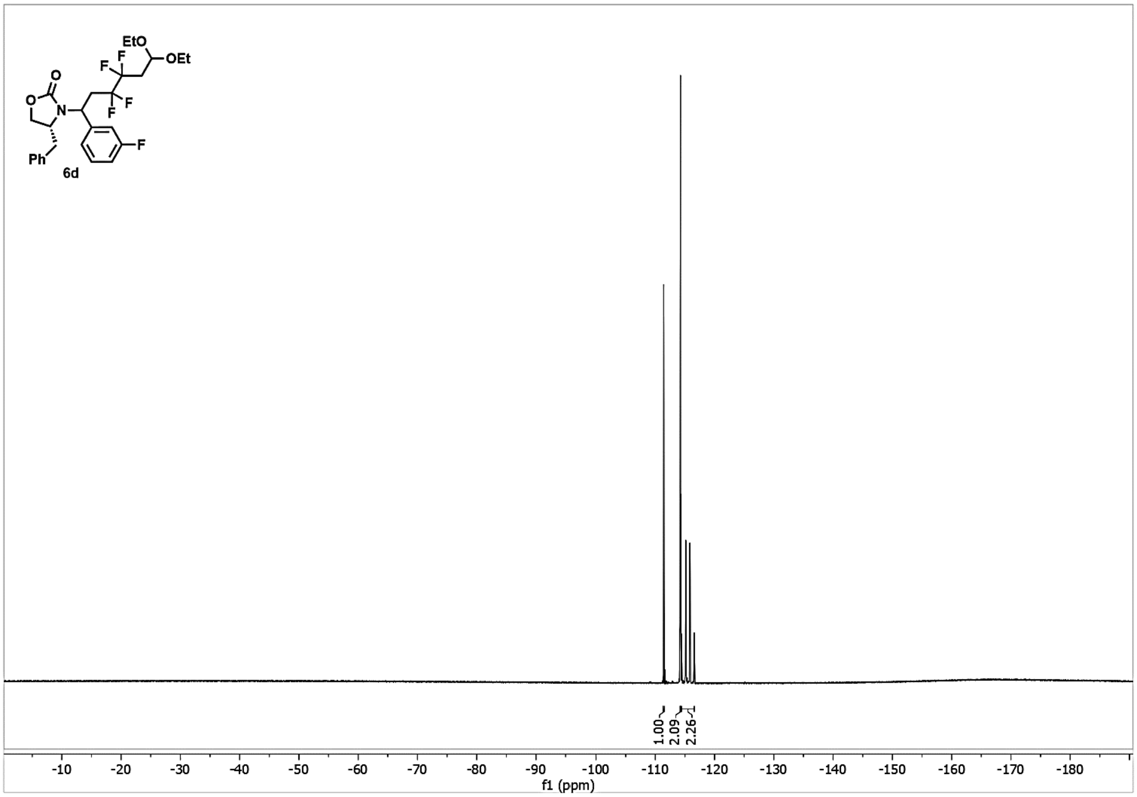

Compound 6e. Top: ^{1}H NMR ($CDCl_3$, 400 MHz). Bottom: ^{13}C NMR ($CDCl_3$, 100 MHz).

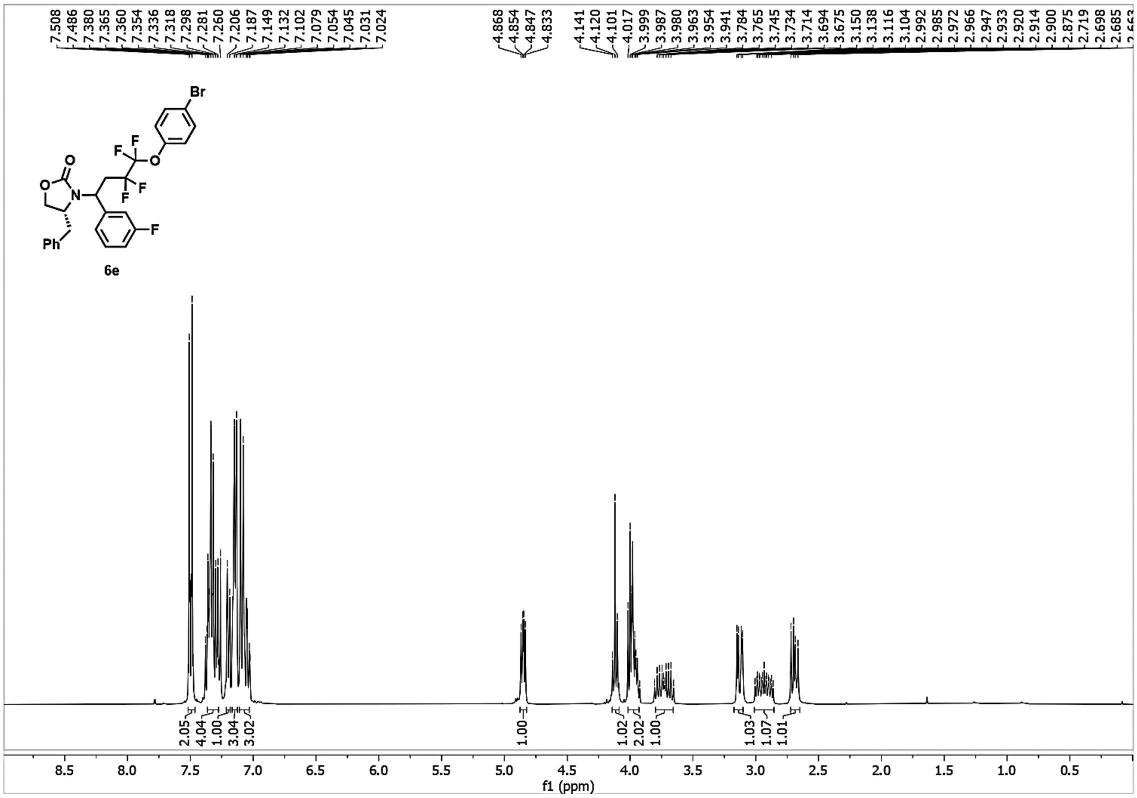

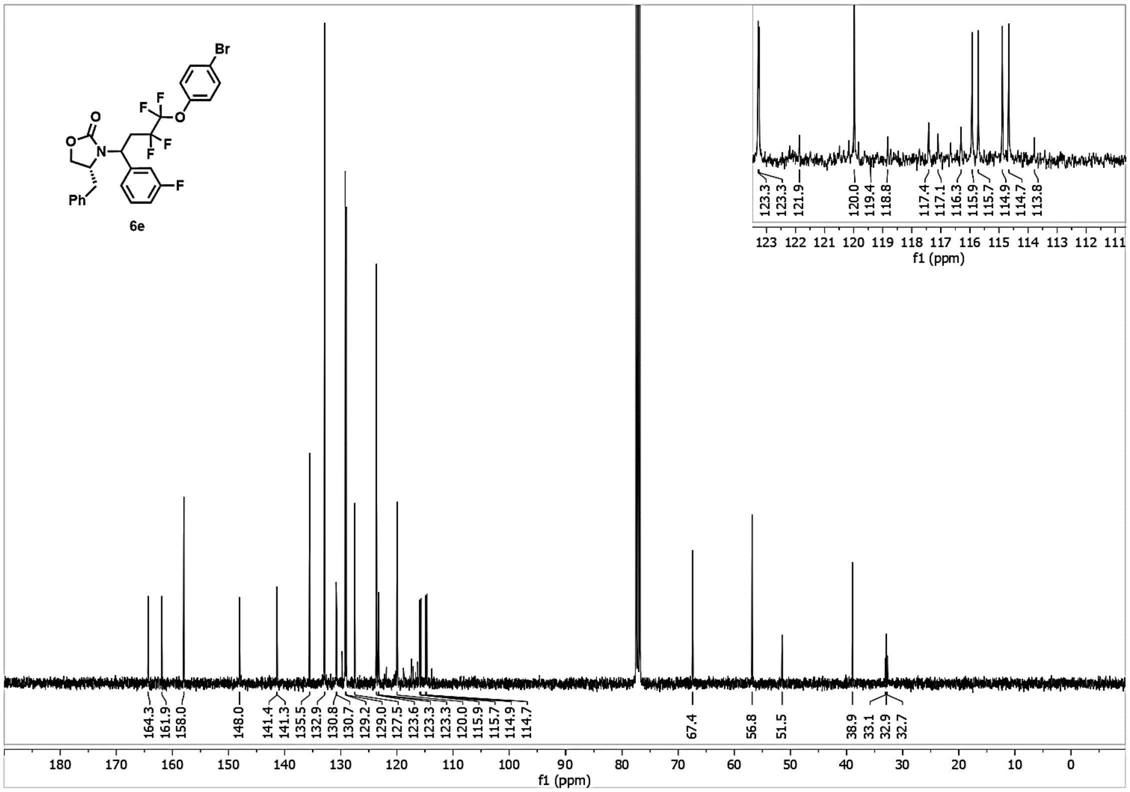

Compound 6e. ^{19}F NMR (CDCl$_3$, 376 MHz).

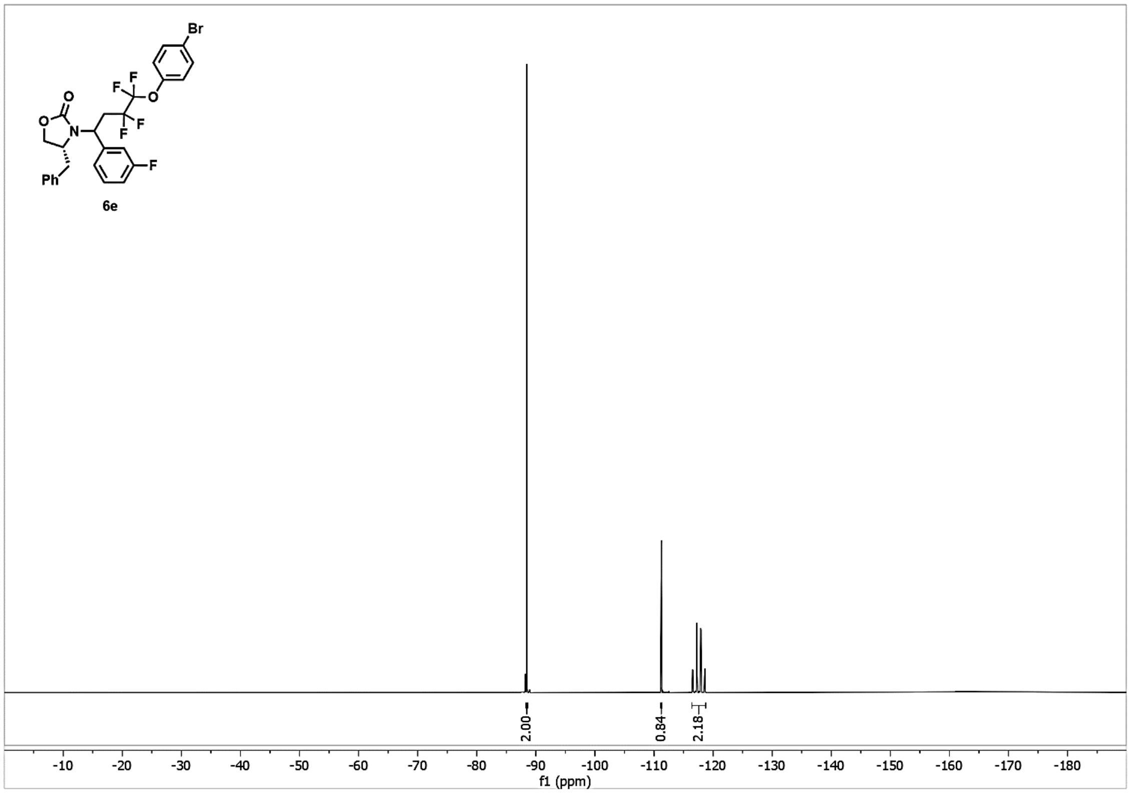

Compound 6f. Top: ^{1}H NMR ($CDCl_3$, 400 MHz). Bottom: ^{13}C NMR ($CDCl_3$, 100 MHz).

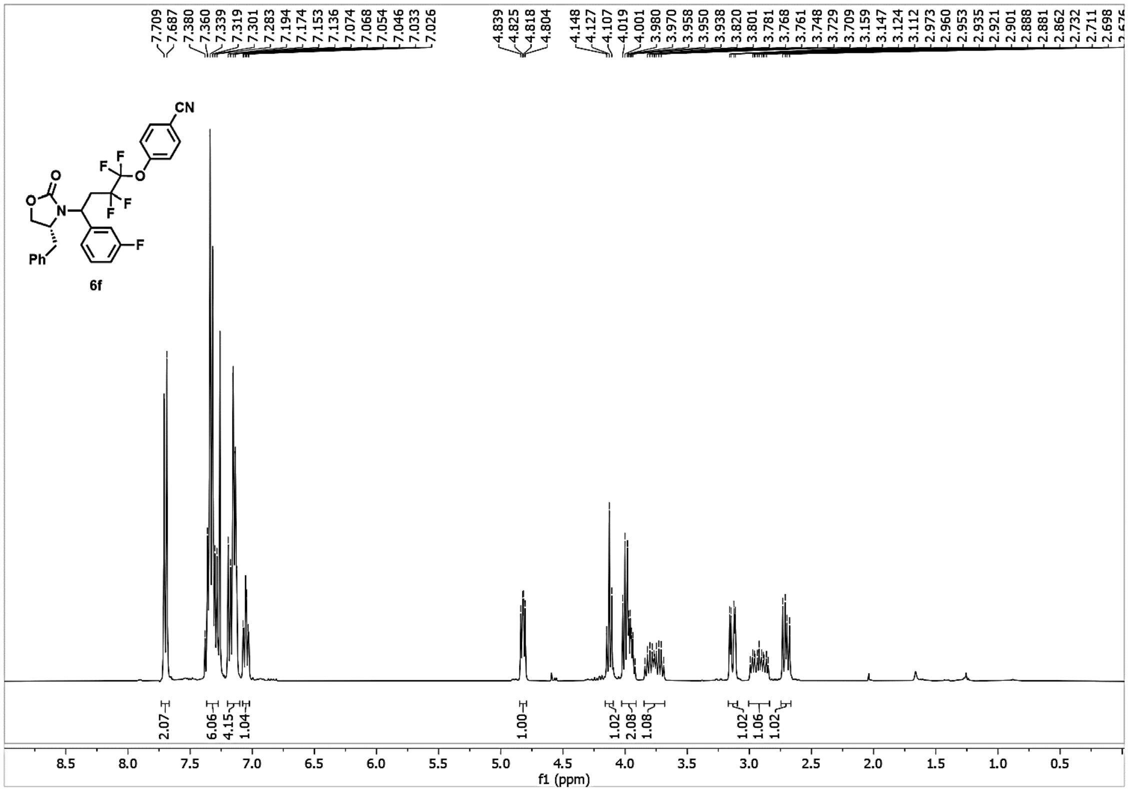

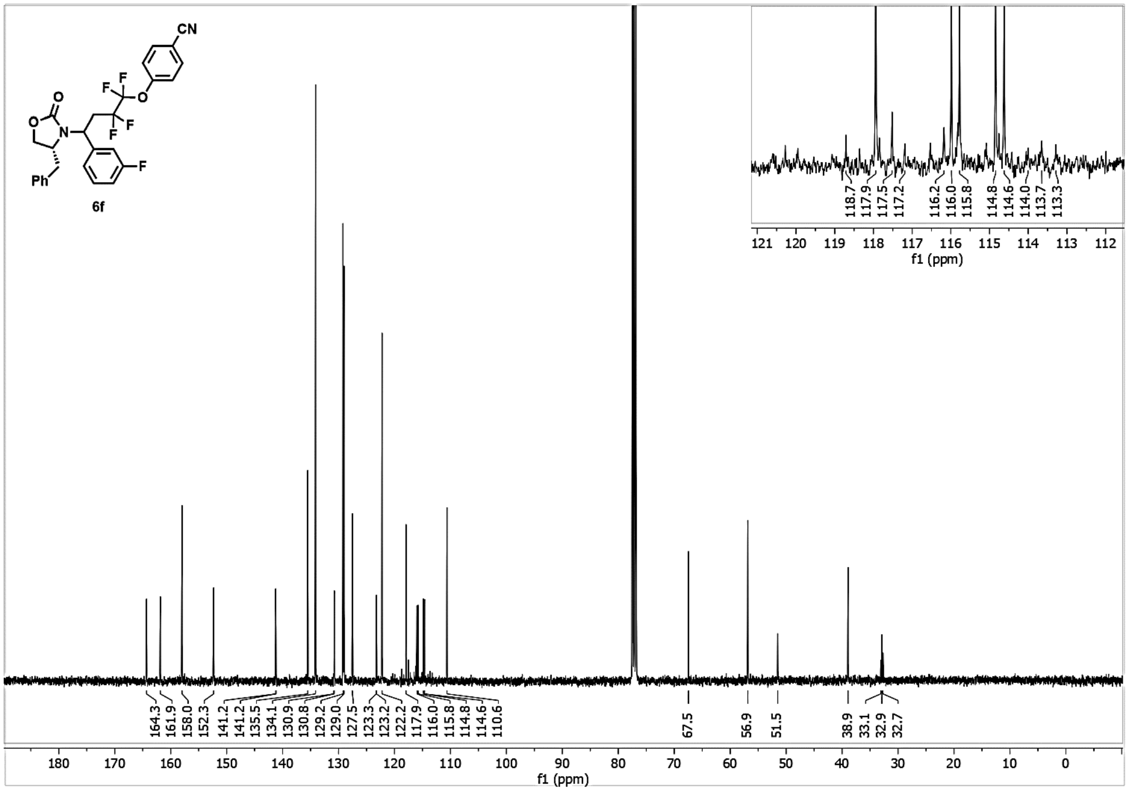

Compound 6f. ^{19}F NMR ($CDCl_3$, 376 MHz).

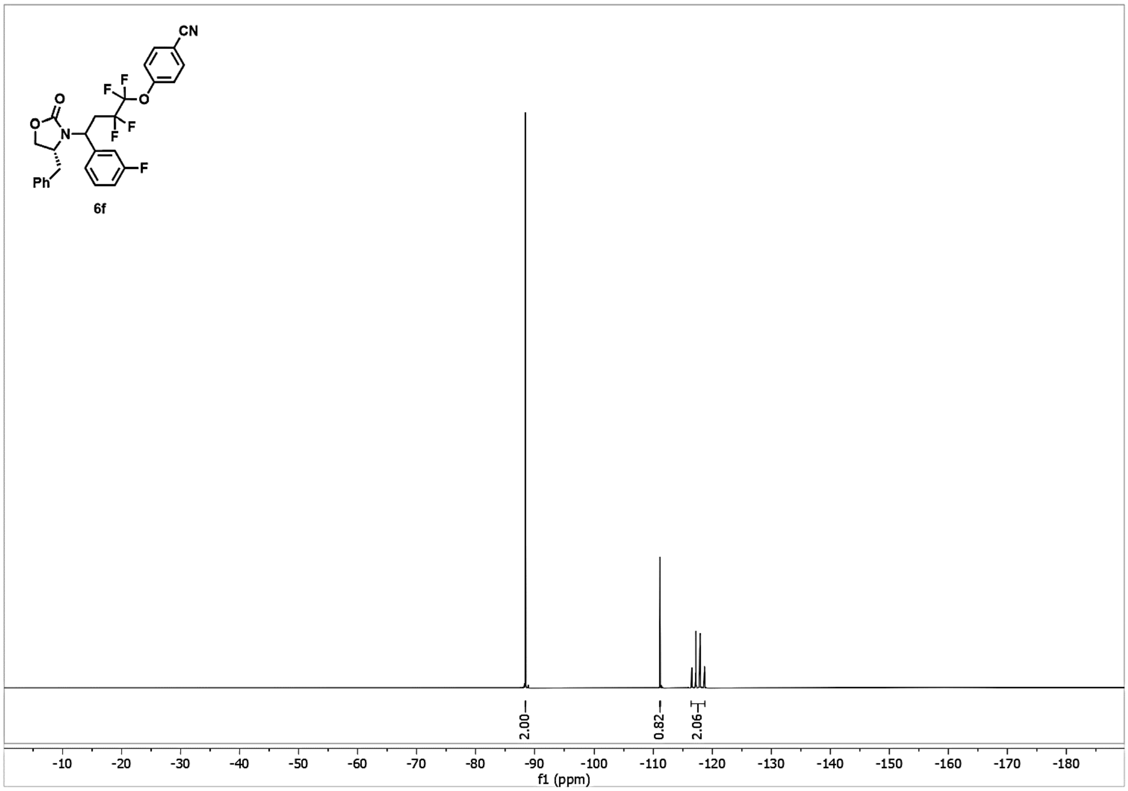

Compound 6g. Top: ^{1}H NMR ($CDCl_3$, 400 MHz). Bottom: ^{13}C NMR ($CDCl_3$, 100 MHz).

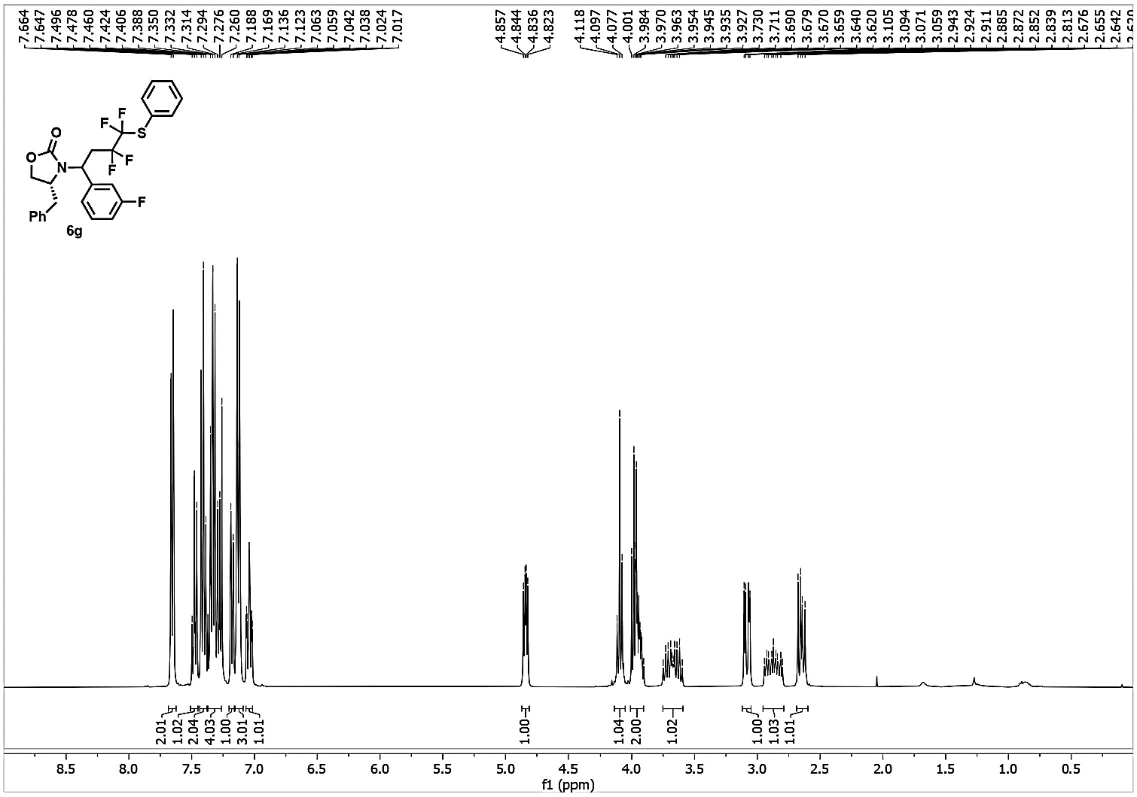

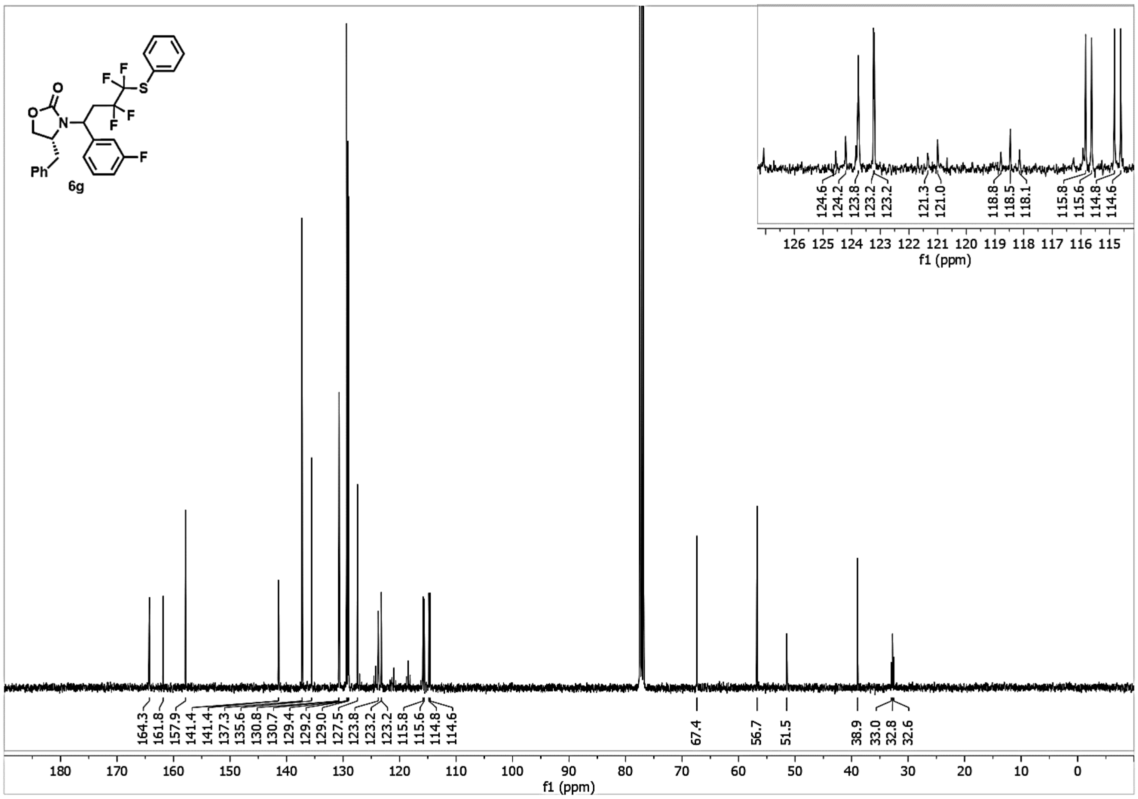

Compound 6g. ^{19}F NMR ($CDCl_3$, 376 MHz).

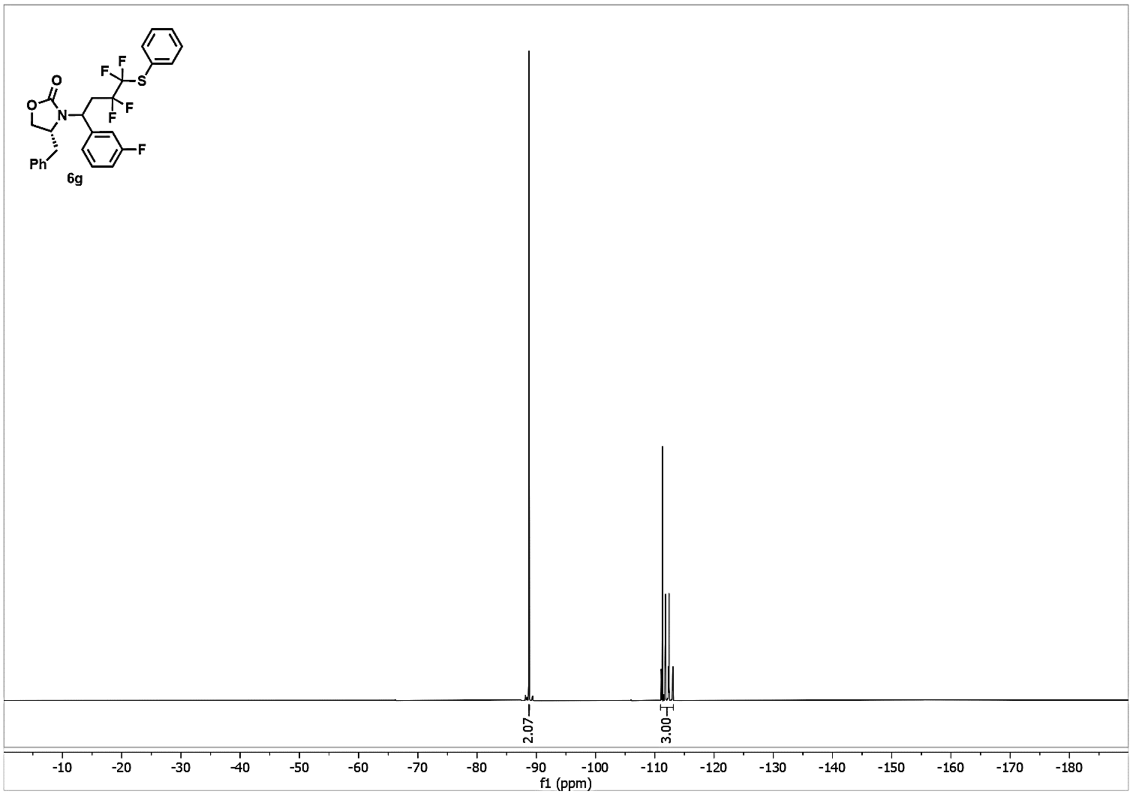

Compound 6h. Top: ^{1}H NMR ($CDCl_3$, 400 MHz). Bottom: ^{13}C NMR ($CDCl_3$, 100 MHz).

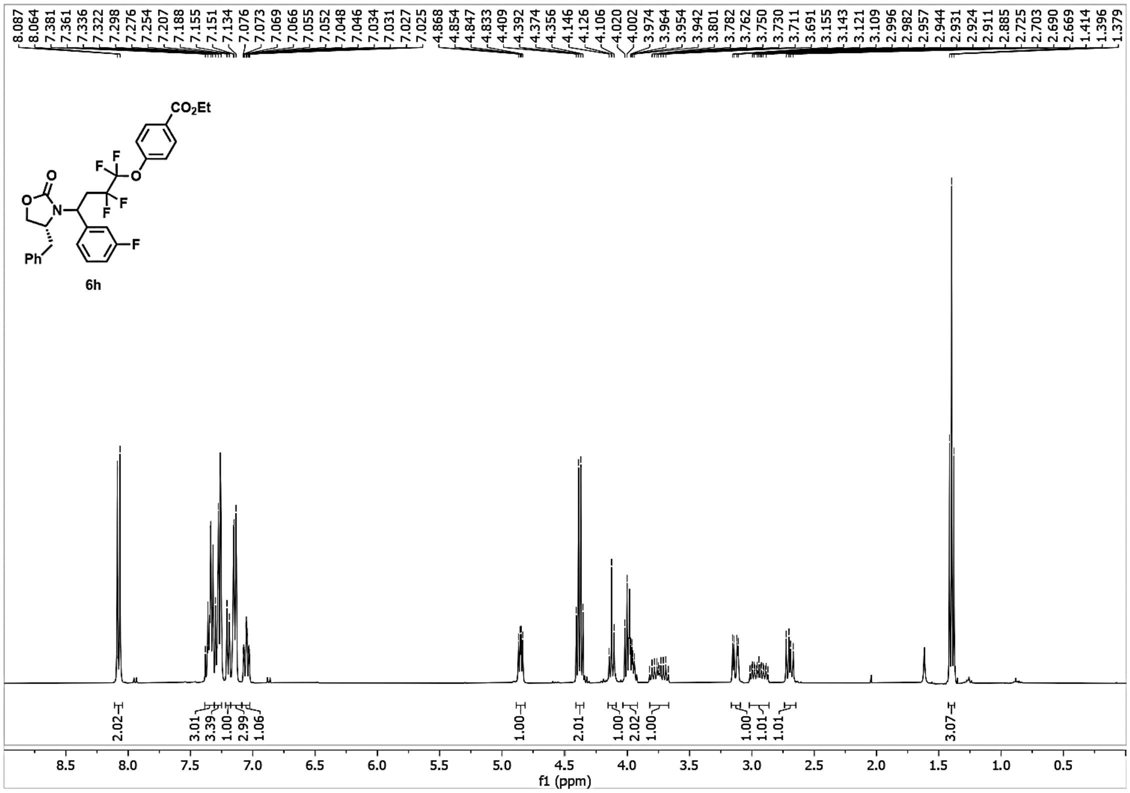

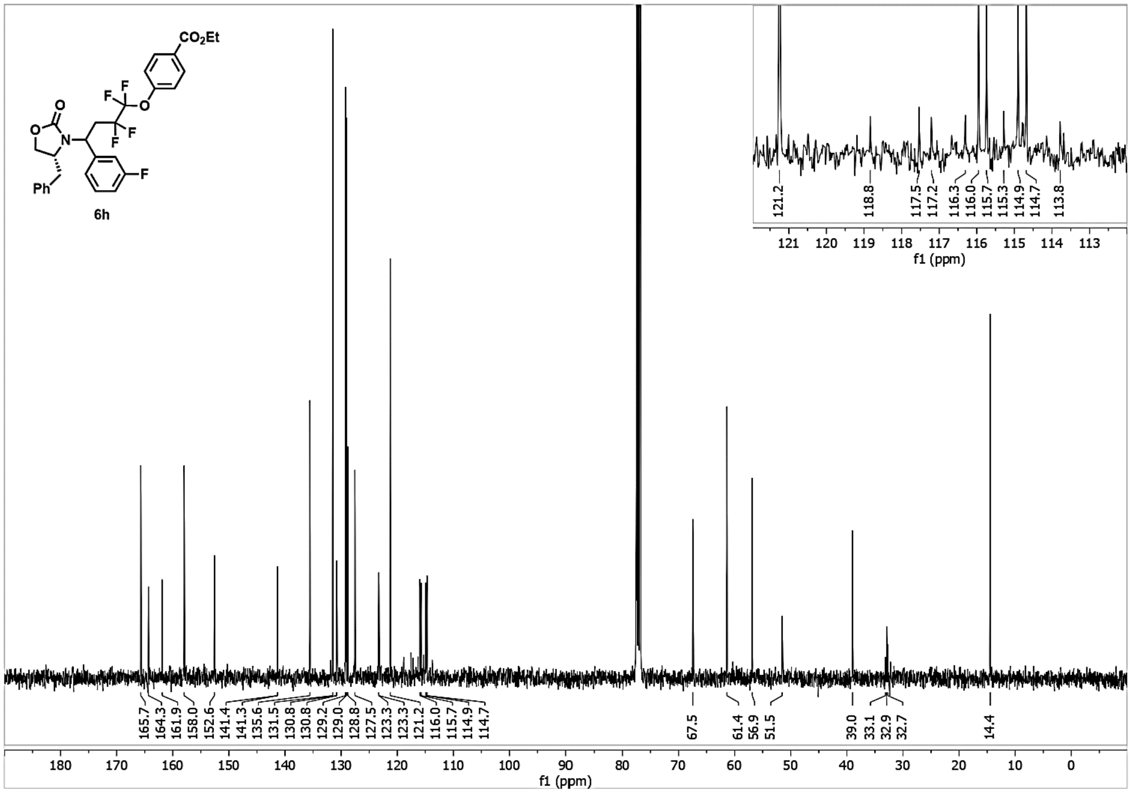

Compound 6h. ^{19}F NMR ($CDCl_3$, 376 MHz).

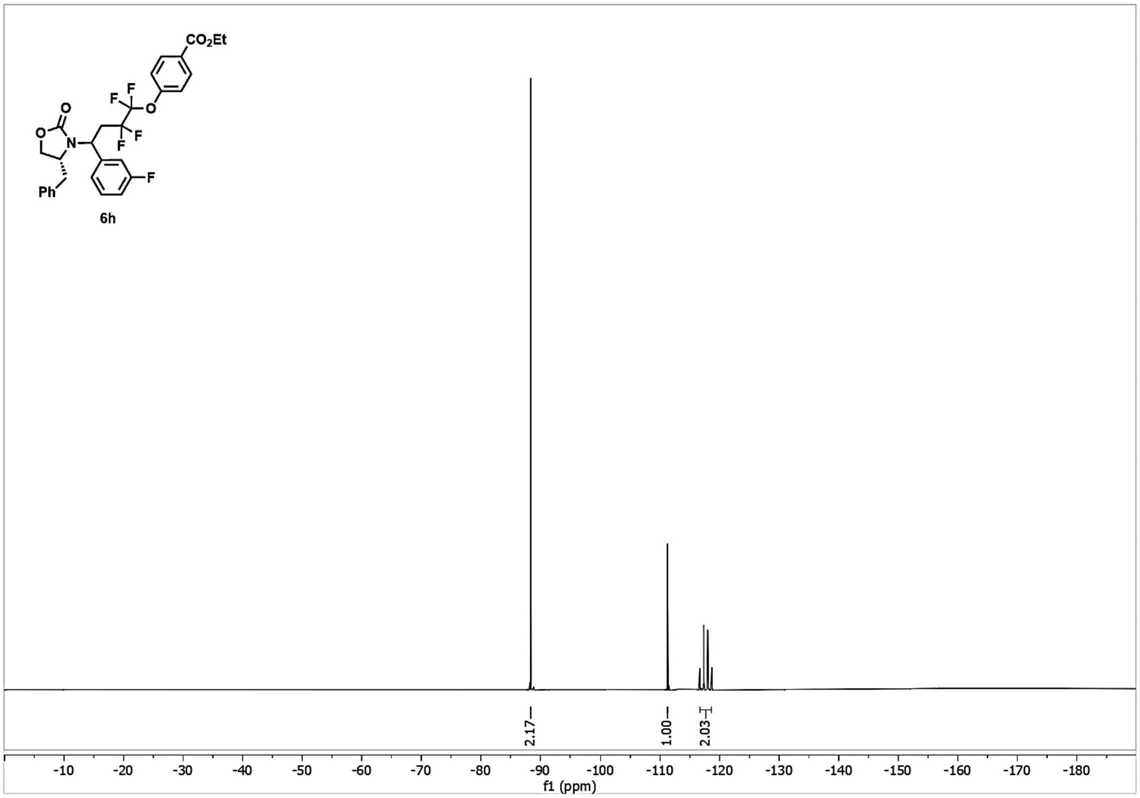

Compound 6i. Top: ^{1}H NMR ($CDCl_3$, 400 MHz). Bottom: ^{13}C NMR ($CDCl_3$, 100 MHz).

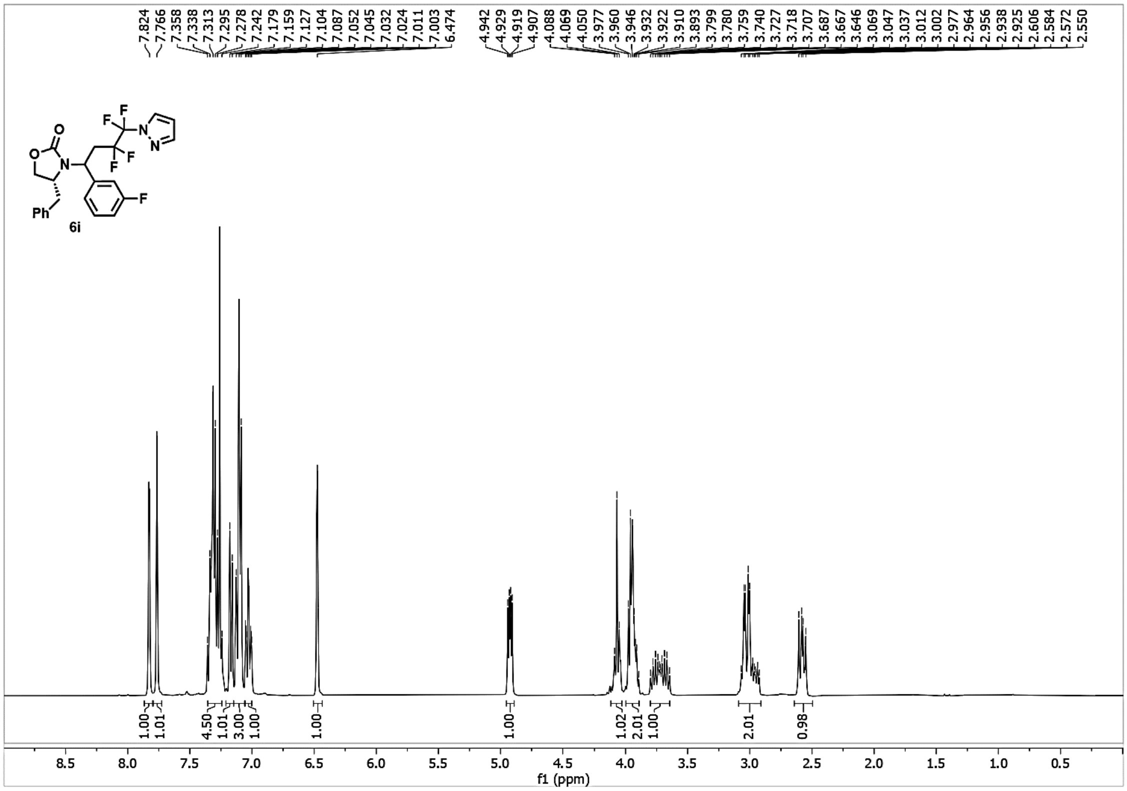

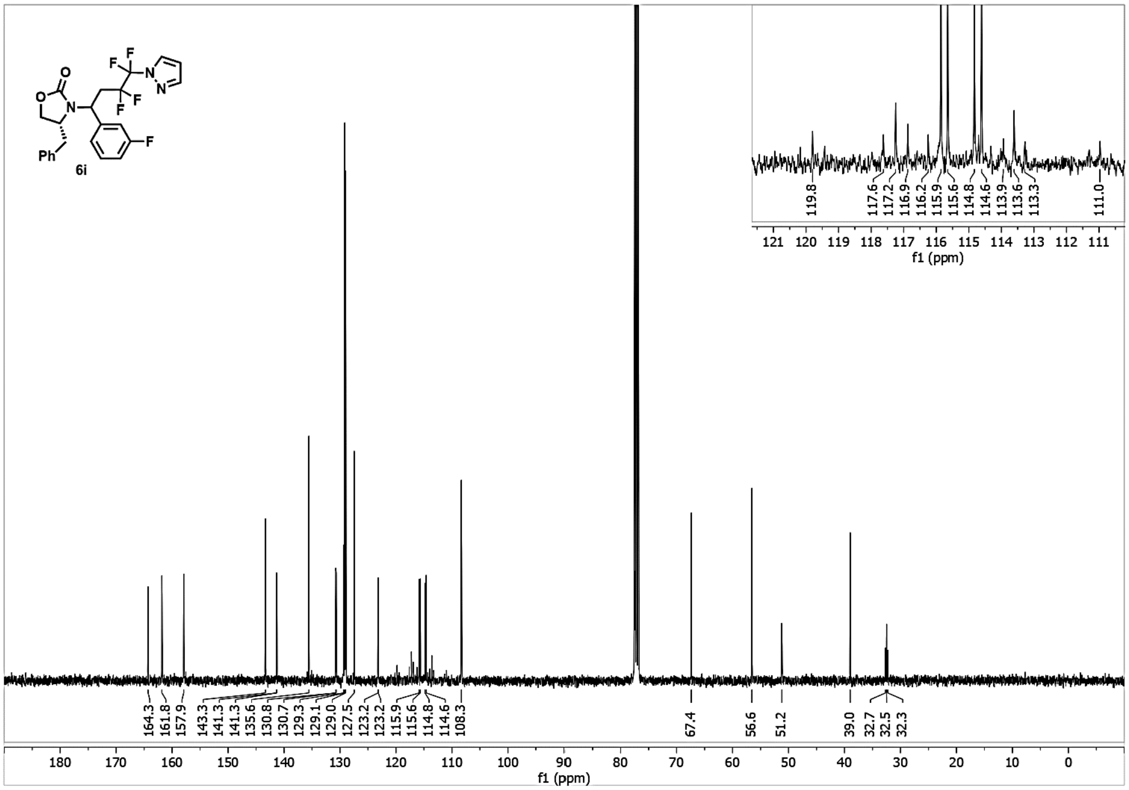

Compound 6i. ^{19}F NMR ($CDCl_3$, 376 MHz).

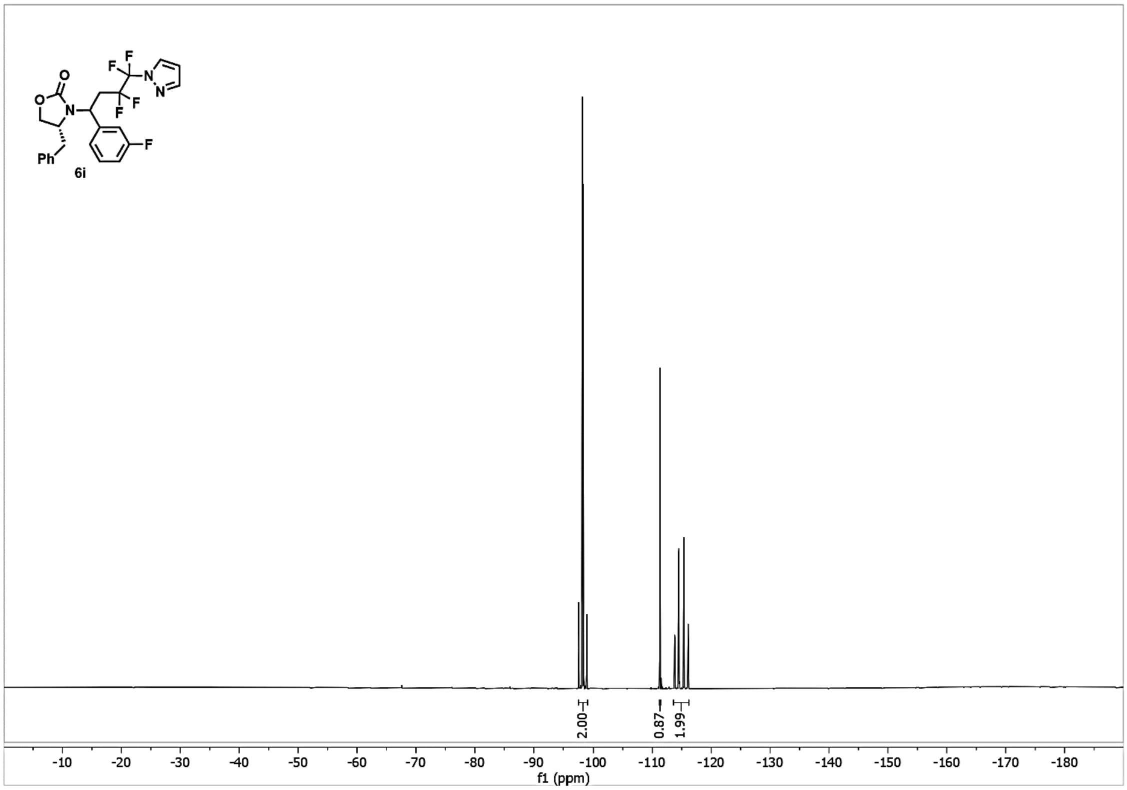

Compound 6j. Top: ^{1}H NMR ($CDCl_3$, 400 MHz). Bottom: ^{13}C NMR ($CDCl_3$, 100 MHz).

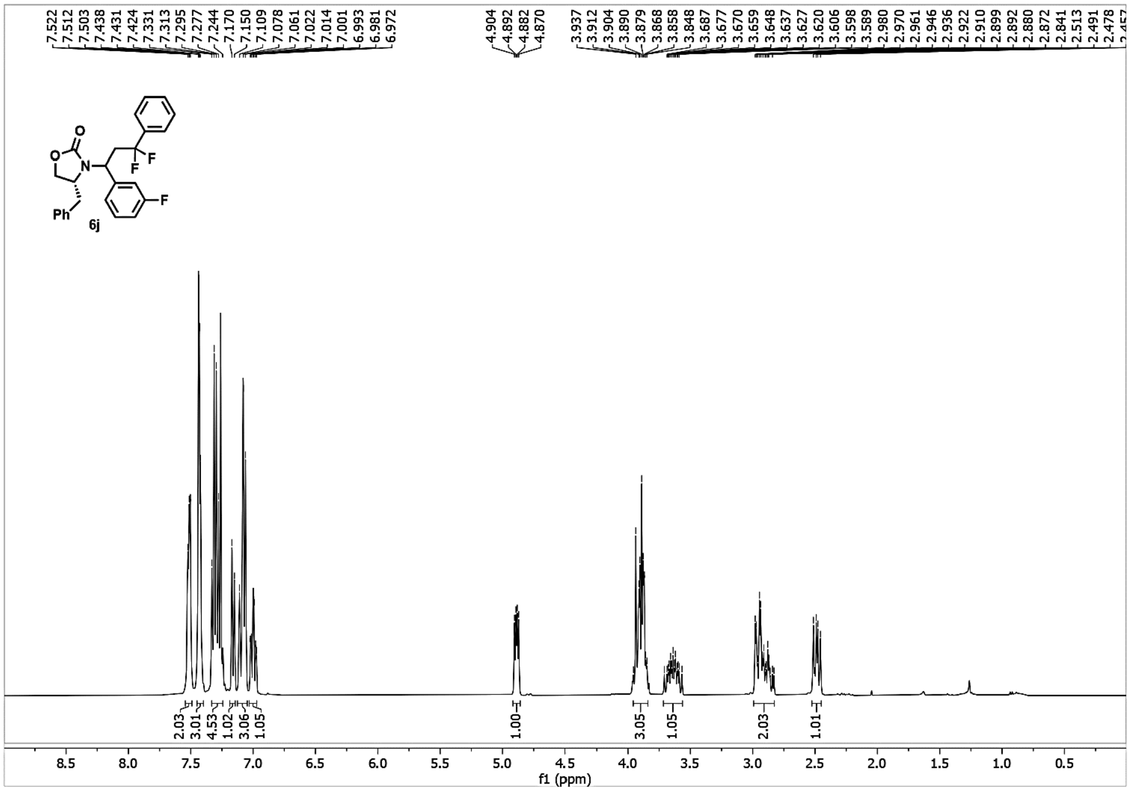

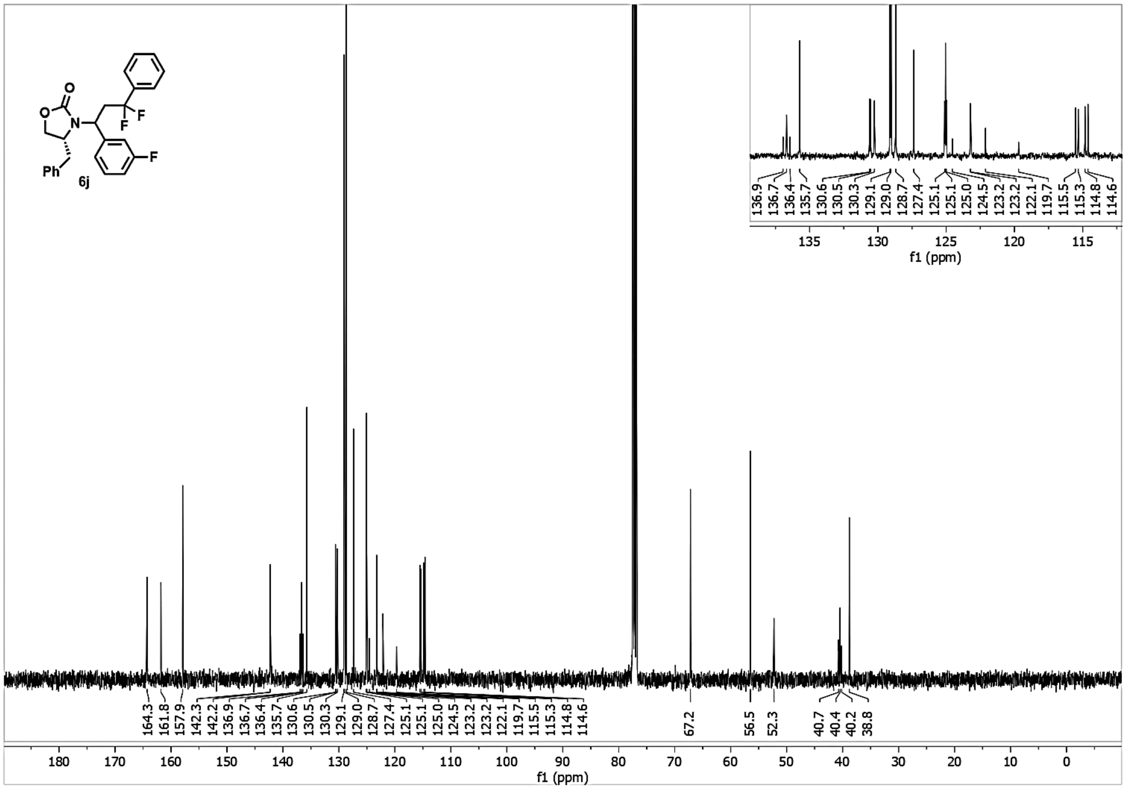

Compound 6j. ^{19}F NMR (CDCl$_3$, 376 MHz).

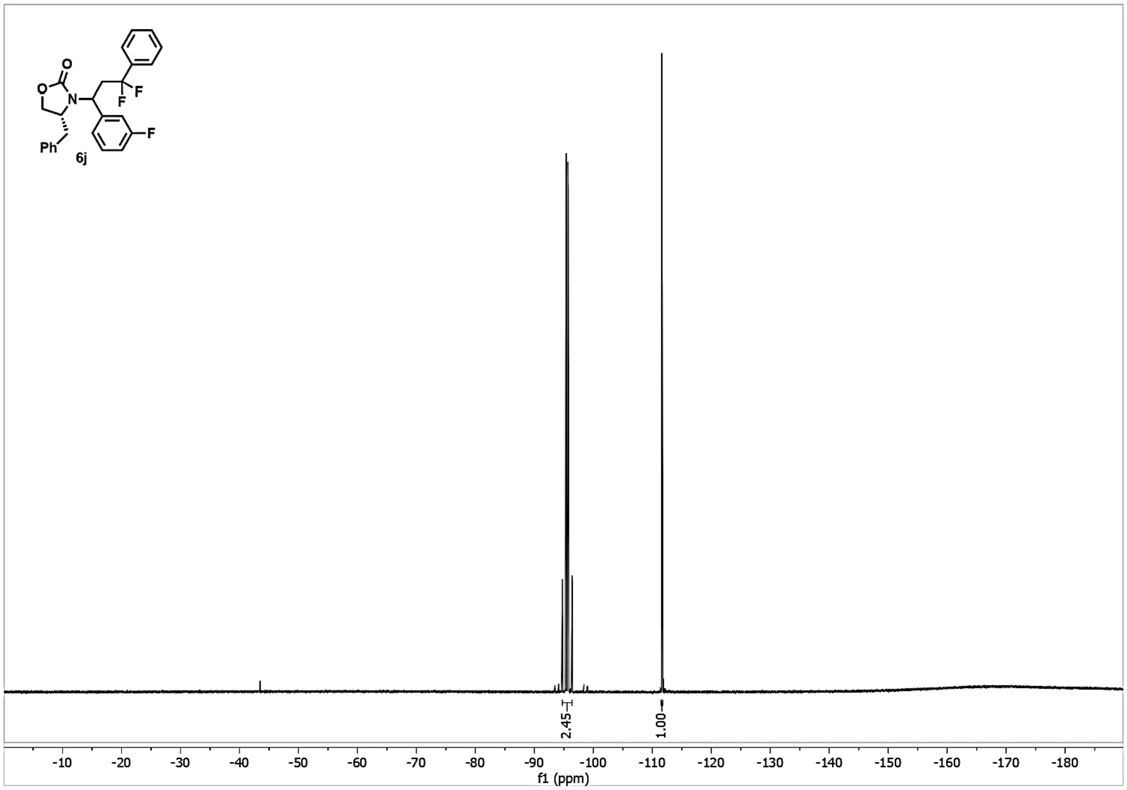

Compound 7. Top: ^{1}H NMR ($CDCl_3$, 400 MHz). Bottom: ^{13}C NMR ($CDCl_3$, 100 MHz).

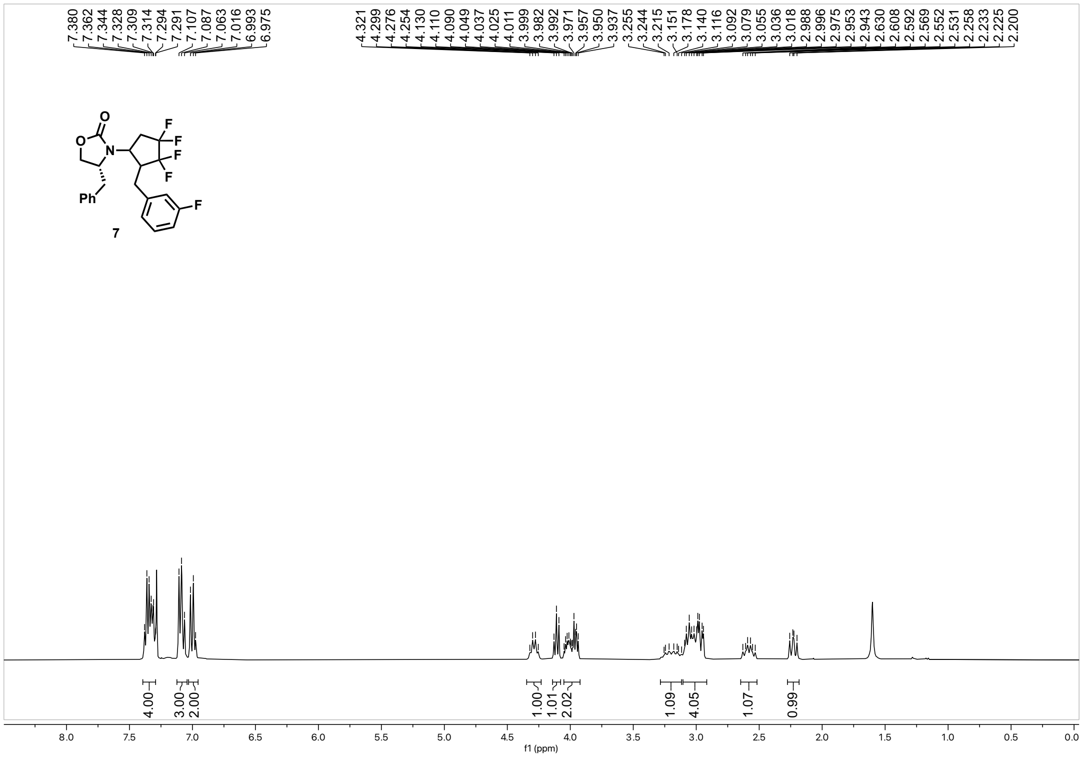

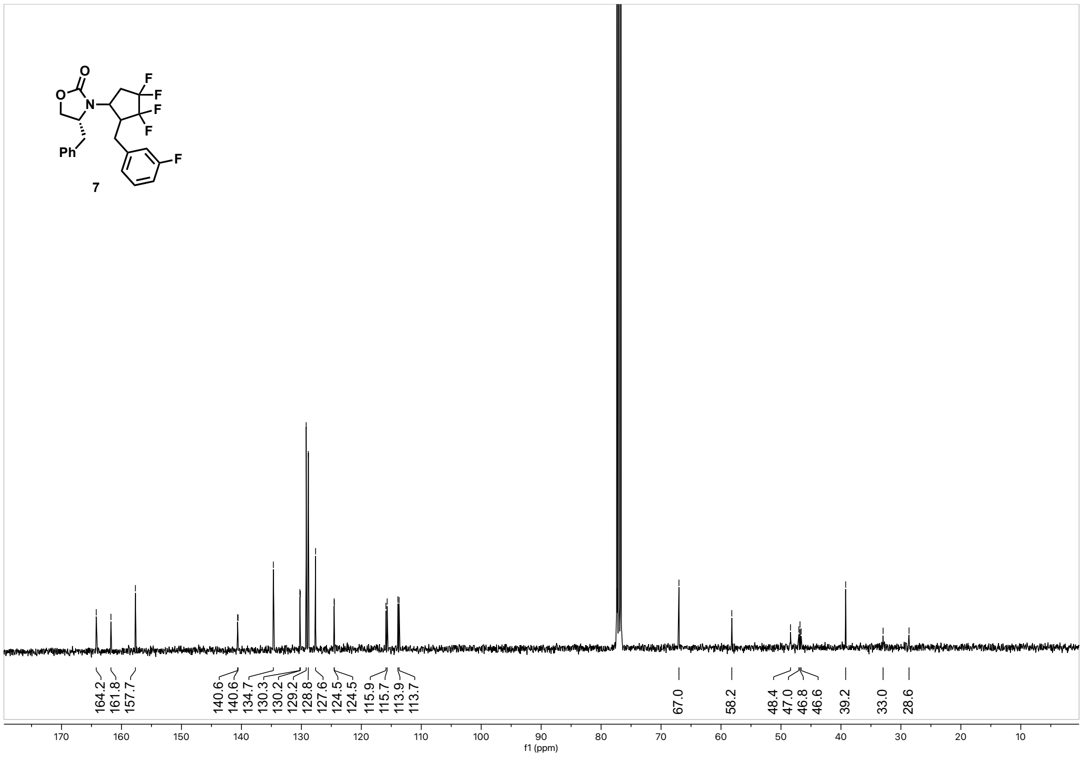

Compound 7. ^{19}F NMR ($CDCl_3$, 376 MHz).

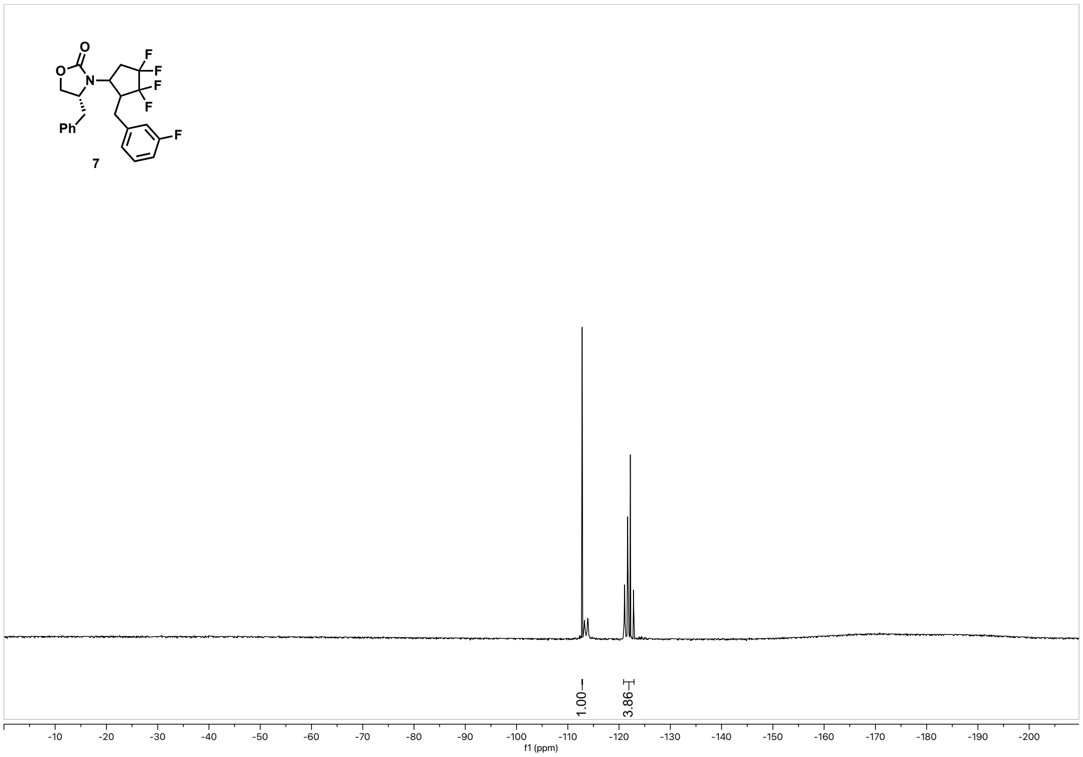

11. Cartesian Coordinates

Table S1. Cartesian coordinates (xyz format) of all the structures involved in each reaction mechanism studied calculated at the uB3LYP-D3/def2svp-SMD(THF) and single-point energies.

$MeCF_2CF_2$·
E(scf) = -515.045739961 a.u.
ν_{min} = 62.82 cm^{-1}

C	9.986017	1.632916	11.268743
C	10.446362	1.293689	9.860021
F	9.130374	0.762951	11.787279
F	10.973143	1.896071	12.114021
C	11.251164	0.022030	9.753680
F	9.327807	1.210796	9.081920
F	11.179674	2.354956	9.412886
H	11.534450	-0.125201	8.701104
H	12.155410	0.103171	10.372673
H	10.645704	-0.828561	10.096572

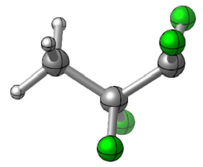

Zero-point correction= 0.059762 (Hartree/Particle)
Thermal correction to Energy= 0.066697
Thermal correction to Enthalpy= 0.067641
Thermal correction to Gibbs Free Energy= 0.027646
Sum of electronic and zero-point Energies= -514.985978
Sum of electronic and thermal Energies= -514.979043
Sum of electronic and thermal Enthalpies= -514.978099
Sum of electronic and thermal Free Energies= -515.018094

uM06L-d3/def2-TZVPP-SMD(THF)
E(scf) = -515.575515539 a.u.

$MeCF_2CF_2Br$
E(scf) = -3088.98469794 a.u.
ν_{min} = 64.81cm^{-1}

C	9.978000	1.646428	11.262665
C	10.451615	1.297201	9.825738
F	9.221339	0.645671	11.733582
F	11.046444	1.772275	12.061346
C	11.249462	0.021350	9.765507
F	9.344878	1.207933	9.041300
F	11.189110	2.345577	9.372674
H	11.537565	-0.145950	8.717996
H	12.150286	0.112747	10.386357
H	10.638347	-0.819492	10.119030
Br	8.938949	3.313027	11.323400

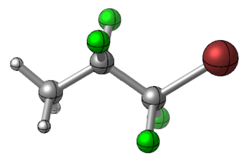

Zero-point correction= 0.062087 (Hartree/Particle)
Thermal correction to Energy= 0.070261
Thermal correction to Enthalpy= 0.071206
Thermal correction to Gibbs Free Energy= 0.028075
Sum of electronic and zero-point Energies= -3088.922611
Sum of electronic and thermal Energies= -3088.914437
Sum of electronic and thermal Enthalpies= -3088.913492
Sum of electronic and thermal Free Energies= -3088.956623

uM06L-d3/def2-TZVPP-SMD(THF)
E(scf) = -3089.70745128 a.u.

(*R*)-1
E(scf) = -556.82751318 a.u.
ν_{min} = 62.41 cm^{-1}

C	6.539652	1.707887	14.602376	C	8.831079	2.090920	15.797913
C	7.807343	2.571483	14.721035	C	8.182027	2.265586	17.183045
C	7.757454	1.360430	12.704202	H	7.279633	1.645815	17.304192
H	6.322749	1.102483	15.489808	H	8.889851	1.970588	17.973983
H	5.654854	2.318767	14.359325	H	7.898571	3.316322	17.362423
H	7.542962	3.617741	14.939389	C	9.220455	0.619278	15.577479
O	6.804730	0.821456	13.502164	H	9.950757	0.303606	16.339855
N	8.290790	2.469324	13.336395	H	8.355039	-0.057583	15.648781
O	8.046037	0.904934	11.624539	H	9.689927	0.471647	14.591566
C	9.127010	3.417944	12.737335	C	10.100940	2.958453	15.743159
H	9.246217	4.308321	13.359609	H	10.774027	2.691263	16.573774
C	9.732000	3.346721	11.541532	H	10.661870	2.812732	14.807952
H	10.325595	4.201160	11.208427	H	9.863346	4.031053	15.838348
H	9.648438	2.477321	10.890735				

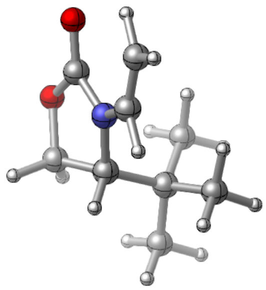

Zero-point correction= 0.232474 (Hartree/Particle)
Thermal correction to Energy= 0.244654
Thermal correction to Enthalpy= 0.245598
Thermal correction to Gibbs Free Energy= 0.195036
Sum of electronic and zero-point Energies= -556.595039
Sum of electronic and thermal Energies= -556.582859
Sum of electronic and thermal Enthalpies= -556.581915
Sum of electronic and thermal Free Energies= -556.632477

uM06L-d3/def2-TZVPP -SMD(THF)
E(scf) = -557.345292358 a.u.

TS1
E(scf) = -1071.88543068 a.u.
ν_{min} = -38.61cm^{-1}

C	6.448791	2.627372	15.386613
C	7.892292	3.033724	15.042340
C	6.765183	2.126390	13.182687
H	6.355324	2.026630	16.298399
H	5.788951	3.506719	15.466537
H	8.076793	4.081094	15.326882
O	6.018639	1.828263	14.271719
N	7.820196	2.934432	13.576696
O	6.499338	1.733538	12.073760
C	8.684286	3.608429	12.715156
H	9.327116	4.318113	13.239959
C	8.800813	3.444308	11.380734
H	8.158509	2.774590	10.813869
H	9.514108	4.068430	10.839829
C	8.998502	2.147345	15.698570
C	10.336423	1.287066	11.319417
C	10.523850	1.029132	9.835896
F	9.617882	0.344761	11.934188
F	11.486942	1.485028	11.969867
C	11.301510	-0.217331	9.494259
F	9.276445	0.972479	9.277412
F	11.148272	2.130791	9.316684
H	11.377698	-0.300873	8.400134
H	12.307683	-0.154345	9.931970
H	10.780706	-1.097183	9.897384
C	8.941230	2.372032	17.220874
H	7.981635	2.052911	17.656927
H	9.734850	1.793149	17.719391
H	9.092583	3.434831	17.474404
C	8.785533	0.659309	15.372543
H	9.572456	0.053287	15.849968
H	7.815455	0.286232	15.735978
H	8.842053	0.475564	14.288874
C	10.390011	2.574907	15.198586
H	11.169843	2.031392	15.756272
H	10.539699	2.348803	14.133500
H	10.562076	3.653264	15.351422

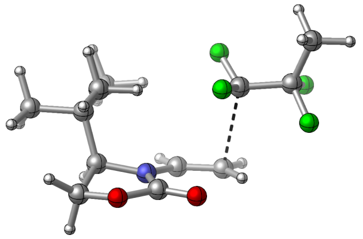

Zero-point correction= 0.293160 (Hartree/Particle)
Thermal correction to Energy= 0.313372
Thermal correction to Enthalpy= 0.314316
Thermal correction to Gibbs Free Energy= 0.242358
Sum of electronic and zero-point Energies= -1071.592270
Sum of electronic and thermal Energies= -1071.572059
Sum of electronic and thermal Enthalpies= -1071.571115
Sum of electronic and thermal Free Energies= -1071.643073

uM06L-d3/def2-TZVPP-SMD(THF)
E(scf) = -1072.92703897 a.u.

Int·
E(scf) = -1071.94154058 a.u.
ν_{min} = 26.67 cm^{-1}

C	6.610677	2.457650	15.384749
C	8.062547	2.822931	15.029239
C	6.882097	1.972713	13.174218
H	6.507971	1.856589	16.295122
H	5.978192	3.356003	15.475422
H	8.275480	3.868728	15.300676
O	6.146680	1.676962	14.272828
N	7.988186	2.713038	13.562377
O	6.566761	1.626899	12.060228
C	8.860815	3.373895	12.706300

H	9.555098	4.050149	13.201698
C	9.119026	2.933182	11.304753
H	8.193268	2.667327	10.778676
H	9.618157	3.742256	10.753054
C	9.148265	1.916570	15.694108
C	10.034647	1.716308	11.207112
C	10.319434	1.243667	9.763432
F	9.506566	0.653131	11.885423
F	11.248651	1.977842	11.789190
C	11.205110	0.033222	9.654551
F	9.110666	1.003906	9.170044
F	10.876298	2.303772	9.098091
H	11.341745	-0.204091	8.589859
H	12.179165	0.244038	10.115513
H	10.733641	-0.816348	10.165808
C	9.075151	2.134319	17.217371
H	8.110550	1.817553	17.643592
H	9.861350	1.548960	17.720080
H	9.229894	3.194988	17.477874
C	8.912948	0.434765	15.356295
H	9.681956	-0.189312	15.839584
H	7.931090	0.078399	15.705056
H	8.977452	0.260821	14.271566
C	10.553873	2.324922	15.219154
H	11.315361	1.761341	15.782649
H	10.713482	2.113050	14.153683
H	10.741198	3.398324	15.388863

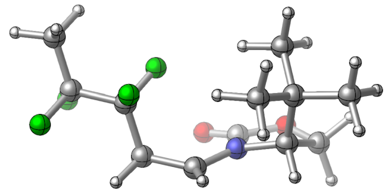

Zero-point correction= 0.296228 (Hartree/Particle)
Thermal correction to Energy= 0.315724
Thermal correction to Enthalpy= 0.316669
Thermal correction to Gibbs Free Energy= 0.247972
Sum of electronic and zero-point Energies= -1071.645312
Sum of electronic and thermal Energies= -1071.625816
Sum of electronic and thermal Enthalpies= -1071.624872
Sum of electronic and thermal Free Energies= -1071.693568

uM06L-d3/def2-TZVPP-SMD(THF)
E(scf) = -1072.97843307 a.u.

^{4}A
E(scf) = -5539.20687667 a.u.
ν_{min} = 10.17 cm^{-1}

C	-5.226887	3.271756	-1.604484
C	-5.040674	2.515185	-2.934106
P	-3.727171	4.288434	-1.095738
P	-3.866639	3.378614	-4.124417
Fe	-2.511270	4.895018	-2.976721
C	-4.525674	5.598528	-0.021604
C	-5.097401	5.117962	1.321925
C	-3.621676	6.832709	0.157581
H	-5.374447	5.920394	-0.654791
C	-5.832487	6.253628	2.050064
H	-4.272950	4.756940	1.961693
H	-5.777449	4.263093	1.169426
C	-4.369073	7.960432	0.880644
H	-2.729665	6.560204	0.747427
H	-3.250787	7.165955	-0.825937
C	-4.936190	7.485959	2.224727
H	-6.196849	5.900243	3.030140
H	-6.727015	6.536534	1.464676
H	-3.698285	8.823962	1.028949
H	-5.199077	8.312908	0.240206
H	-5.496460	8.299883	2.715973
H	-4.097735	7.229614	2.899247
C	-2.779761	3.137291	0.038771
C	-2.248138	1.948993	-0.790306
C	-1.617495	3.876121	0.729118
H	-3.469833	2.750753	0.809909
C	-1.353601	1.013663	0.032142
H	-1.674523	2.349510	-1.648477

H	-3.084866	1.375890	-1.218566
C	-0.728906	2.929681	1.546493
H	-1.007836	4.377123	-0.046082
H	-2.005266	4.672815	1.381339
C	-0.199613	1.773042	0.693427
H	-0.968032	0.205221	-0.612393
H	-1.964465	0.528044	0.815517
H	0.106246	3.496168	1.992581
H	-1.316648	2.519614	2.388705
H	0.412327	1.088972	1.305665
H	0.466668	2.176712	-0.091769
H	-4.594319	1.526522	-2.747356
H	-6.014675	2.334630	-3.417851
H	-6.050363	3.996718	-1.694557
H	-5.505653	2.575799	-0.796212
C	-3.163073	1.926279	-5.074243
H	-2.917448	1.217621	-4.260631
C	-1.833335	2.277709	-5.768567
H	-2.010178	3.027878	-6.557797
H	-1.148290	2.750272	-5.043896
C	-1.188710	1.033828	-6.393932
H	-0.257433	1.312903	-6.915670
H	-0.901694	0.331788	-5.588879
C	-2.151182	0.327622	-7.357098
H	-2.357720	0.995000	-8.214592
H	-1.681946	-0.580807	-7.771839
C	-3.474450	-0.023699	-6.666124
H	-3.282959	-0.771463	-5.874183
H	-4.169702	-0.494513	-7.382227
C	-4.127815	1.216247	-6.037786
H	-5.056109	0.933139	-5.513826
H	-4.417943	1.913292	-6.843177
C	-5.022206	4.290638	-5.286094
H	-5.738455	3.562496	-5.707032
C	-4.224089	4.946037	-6.428704
H	-3.416200	5.552945	-5.980916
H	-3.738049	4.173308	-7.044239
C	-5.102191	5.834495	-7.319504
H	-4.479561	6.311667	-8.095656
H	-5.839978	5.204198	-7.850784
C	-5.844923	6.894236	-6.500257
H	-5.107969	7.578158	-6.040494
H	-6.487287	7.509335	-7.153503
C	-6.676156	6.236618	-5.394631
H	-7.469715	5.618515	-5.854942
H	-7.186824	7.002252	-4.785573
C	-5.807242	5.354374	-4.490202
H	-6.439522	4.871560	-3.728003
H	-5.082098	5.987961	-3.947177
Br	-1.754035	7.052480	-3.729403

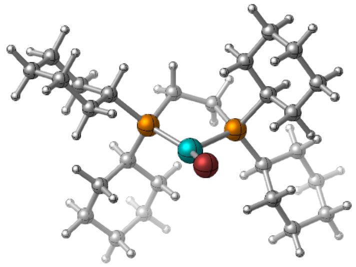

Zero-point correction= 0.701943 (Hartree/Particle)
Thermal correction to Energy= 0.735638
Thermal correction to Enthalpy= 0.736583
Thermal correction to Gibbs Free Energy= 0.633077
Sum of electronic and zero-point Energies= -5538.504933
Sum of electronic and thermal Energies= -5538.471238
Sum of electronic and thermal Enthalpies= -5538.470294
Sum of electronic and thermal Free Energies= -5538.573800

uM06L-d3/def2-TZVPP-SMD(THF)
E(scf) = -5540.55830147 a.u.

^{5}B
E(scf) = -8113.18316441 a.u.
ν_{min} = 17.04 cm^{-1}

Fe	5.953849	10.979421	3.711609
P	4.032054	9.986494	2.495537

P	4.226232	12.472471	4.674303
C	2.469845	10.736572	3.191979
H	1.682374	10.723095	2.421387
H	2.143615	10.048396	3.985071
C	2.627338	12.160796	3.761748
H	1.776985	12.401023	4.420001
H	2.609027	12.896415	2.944724
C	3.738230	8.147196	2.588494
H	4.623946	7.722035	2.083537
C	3.764193	7.654363	4.048916
H	2.927577	8.110405	4.608094
H	4.691910	7.983579	4.541028
C	3.631721	6.127601	4.121860
H	4.523994	5.666457	3.659760
H	3.622190	5.805085	5.176839
C	2.374884	5.634270	3.396816
H	2.317385	4.532987	3.429890
H	1.479777	6.013050	3.924931
C	2.346477	6.125640	1.945327
H	1.418300	5.801230	1.444651
H	3.182819	5.663250	1.389391
C	2.476047	7.653625	1.859031
H	2.488193	7.964492	0.802409
H	1.585304	8.118534	2.318701
C	4.006085	10.402245	0.678115
H	3.067865	9.994375	0.262195
C	5.202400	9.736857	-0.030835
H	5.140713	8.640245	0.060492
H	6.131220	10.051945	0.476235
C	5.276162	10.126842	-1.512846
H	6.165895	9.664164	-1.972555
H	4.396703	9.715525	-2.042731
C	5.306914	11.647867	-1.693110
H	5.338182	11.908255	-2.764815
H	6.231453	12.048084	-1.239297
C	4.094363	12.298826	-1.020453
H	4.135592	13.396443	-1.124889
H	3.172666	11.966814	-1.533946
C	4.005868	11.929500	0.465977
H	3.095023	12.372511	0.898060
H	4.865239	12.366337	1.001381
C	4.469788	14.318898	4.575945
H	5.388144	14.482212	5.168097
C	3.336779	15.159831	5.190091
H	2.397285	14.958338	4.644417
H	3.163175	14.874546	6.239617
C	3.653784	16.660732	5.111592
H	2.811969	17.241624	5.525532
H	4.529500	16.875202	5.751495
C	3.959307	17.104097	3.676704
H	4.219523	18.176005	3.653954
H	3.048619	16.987236	3.059760
C	5.088293	16.264834	3.068839
H	6.025990	16.459484	3.621164
H	5.272321	16.562505	2.022587
C	4.773330	14.764589	3.131695
H	3.898932	14.559425	2.488645
H	5.612405	14.179300	2.726048
C	3.844500	12.119378	6.464526
H	2.999241	12.769676	6.751356
C	3.411704	10.652027	6.656237
H	4.188780	9.990143	6.238836
H	2.482849	10.454916	6.098607
C	3.196840	10.310425	8.136529
H	2.932557	9.243573	8.233124
H	2.334394	10.885855	8.521827
C	4.435007	10.636606	8.977462
H	4.250178	10.404455	10.040133
H	5.273925	9.995730	8.651229
C	4.831888	12.107076	8.812423
H	5.741173	12.330810	9.395655
H	4.028801	12.748625	9.220898
C	5.065689	12.465203	7.339068
H	5.938819	11.904948	6.960885
H	5.307334	13.537045	7.251499
Br	7.508063	12.101949	2.242922
Br	6.954142	9.524548	5.358788

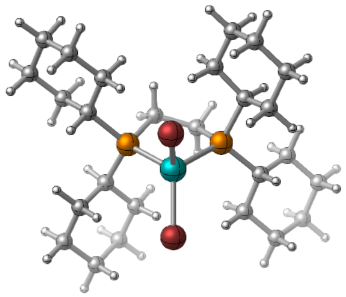

Zero-point correction= 0.704274 (Hartree/Particle)
Thermal correction to Energy= 0.740138
Thermal correction to Enthalpy= 0.741083
Thermal correction to Gibbs Free Energy= 0.632079
Sum of electronic and zero-point Energies= -8112.478890
Sum of electronic and thermal Energies= -8112.443026
Sum of electronic and thermal Enthalpies= -8112.442082
Sum of electronic and thermal Free Energies= -8112.551086

uM06L-d3/def2-TZVPP-SMD(THF)
E(scf) = -8114.71636962 a.u.

^{5}C
E(scf) = -5770.72188917 a.u.
ν_{min} = 5.53 cm^{-1}

Br	10.034914	5.179432	10.341106
Fe	9.170977	6.281744	12.351042
P	8.409734	8.547387	11.658959
P	10.951099	7.584488	13.514092
C	9.332329	9.806390	12.685970
H	9.337270	10.781783	12.173409
H	8.739212	9.938557	13.602109
C	10.770652	9.386263	13.049783
H	11.160215	10.022428	13.860724
H	11.435818	9.540165	12.188392
C	6.609082	8.902939	11.988570
H	6.424053	9.977151	11.809549
C	6.260144	8.576266	13.455308
H	6.553722	7.533851	13.666779
H	6.839494	9.210846	14.143943
C	4.762186	8.743823	13.739294
H	4.552712	8.469850	14.787171
H	4.486481	9.809135	13.629039
C	3.912430	7.900617	12.783511
H	2.838435	8.055643	12.982509
H	4.118213	6.830193	12.967011
C	4.239452	8.234152	11.324557
H	3.940700	9.277962	11.114637
H	3.654708	7.595906	10.640624
C	5.735750	8.072520	11.026189
H	5.936248	8.352202	9.980010
H	6.016372	7.007921	11.125023
C	8.710236	9.027417	9.882301
H	8.217812	8.214740	9.318838
C	8.106461	10.370041	9.437036
H	7.023548	10.400812	9.634487
H	8.560660	11.188514	10.024449
C	8.362368	10.618819	7.942309
H	7.811653	9.860238	7.356155
H	7.951366	11.600558	7.650917
C	9.853228	10.537129	7.594965
H	10.386472	11.367651	8.094565
H	10.001775	10.675555	6.510468
C	10.458837	9.206097	8.054926
H	11.541751	9.181164	7.844872
H	10.007085	8.376992	7.480158
C	10.213908	8.967415	9.549749
H	10.744301	9.748785	10.122152
H	10.627290	7.994888	9.856317
C	12.750173	7.209607	13.201391
H	13.352690	7.958954	13.745874
C	13.084290	7.305194	11.698569
H	12.415706	6.626780	11.141700
H	12.890511	8.321453	11.320321
C	14.544041	6.924114	11.420086
H	14.740169	6.979701	10.335760
H	15.214144	7.661384	11.900705
C	14.868643	5.524604	11.952373
H	14.268318	4.779804	11.398252

H	15.928439	5.277668	11.769896	C	9.071354	7.703939	17.264476
C	14.544898	5.419362	13.445967	H	9.126744	6.620933	17.478160
H	15.216668	6.090903	14.012371	H	8.050666	8.025698	17.532350
H	14.738860	4.397269	13.813161	C	9.312367	7.928185	15.767323
C	13.088941	5.804869	13.739269	H	9.161927	8.998274	15.539534
H	12.903450	5.747830	14.823782	H	8.577649	7.366355	15.170090
H	12.412553	5.073278	13.260468	C	8.087722	5.044772	13.593165
C	10.742977	7.518415	15.367952	C	8.736558	4.383066	14.660591
H	10.845504	6.441787	15.590640	H	9.804254	4.560504	14.833568
C	11.785437	8.277642	16.204010	C	8.065928	3.501269	15.519503
H	11.728987	9.356838	15.973967	C	6.701653	3.247926	15.334246
H	12.805807	7.950489	15.948728	H	6.169960	2.564218	16.002767
C	11.538632	8.067466	17.706460	C	6.026444	3.876361	14.280976
H	11.712505	7.003163	17.950103	H	4.960672	3.681832	14.121556
H	12.272776	8.647555	18.291301	C	6.714499	4.753794	13.431570
C	10.109815	8.448429	18.111963	H	6.154351	5.227263	12.617530
H	9.949801	8.245260	19.184514	H	8.606937	3.010952	16.335608
H	9.973361	9.537410	17.973933				

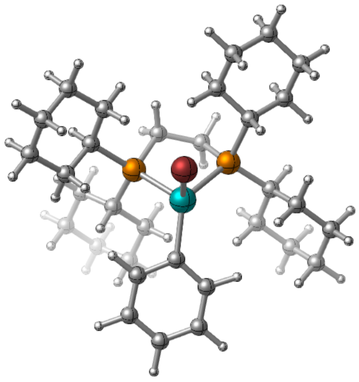

Zero-point correction= 0.792628 (Hartree/Particle)
Thermal correction to Energy= 0.832061
Thermal correction to Enthalpy= 0.833005
Thermal correction to Gibbs Free Energy= 0.715517
Sum of electronic and zero-point Energies= -5769.929261
Sum of electronic and thermal Energies= -5769.889828
Sum of electronic and thermal Enthalpies= -5769.888884
Sum of electronic and thermal Free Energies= -5770.006372

uM06L-d3/def2-TZVPP-SMD(THF)
E(scf) = -5772.27200485 a.u.

^{4}TS2-(*R*,*R*)
E(scf) = -6842.69770256 a.u.
ν_{min} = -43.20 cm^{-1}

Br	10.423386	8.410426	7.490336	H	8.961279	12.520906	11.252429
Fe	9.518816	8.875787	9.767950	C	10.967765	12.017939	10.582434
P	8.582346	11.200969	9.252128	H	11.409351	12.659611	11.361329
P	11.217676	10.226457	11.048783	H	11.579902	12.161688	9.680640
C	9.513342	12.430165	10.306724	C	6.789720	11.542813	9.661976
H	9.482043	13.424527	9.832015	H	6.622612	12.625076	9.515489

C	6.467381	11.201478	11.130304
H	6.723787	10.146672	11.315756
H	7.082214	11.805335	11.815310
C	4.982645	11.418865	11.451063
H	4.785811	11.131489	12.498152
H	4.746056	12.496257	11.369522
C	4.077889	10.630341	10.498739
H	3.015946	10.822712	10.728683
H	4.247537	9.548701	10.650500
C	4.384053	10.983775	9.039939
H	4.124726	12.043637	8.858245
H	3.757762	10.383733	8.358049
C	5.864651	10.759187	8.708927
H	6.058604	11.035856	7.660655
H	6.092930	9.685302	8.797418
C	8.808815	11.838311	7.503781
H	8.445355	10.999188	6.884289
C	8.018902	13.107915	7.136400
H	6.944089	12.983630	7.333163
H	8.362145	13.947200	7.768789
C	8.218138	13.470008	5.655796
H	7.772161	12.671693	5.034331
H	7.665857	14.396120	5.420762
C	9.698785	13.621657	5.291147
H	10.113107	14.500084	5.820605
H	9.808850	13.824427	4.212045
C	10.497404	12.376309	5.690576
H	11.570609	12.521720	5.478806
H	10.170510	11.513457	5.082291
C	10.301485	12.040840	7.173785
H	10.695259	12.878585	7.775915
H	10.874400	11.140871	7.436938
C	13.067918	10.063929	10.791459
H	13.497662	11.015960	11.155654
C	13.380042	9.925935	9.287296
H	12.904225	9.007723	8.908074
H	12.936975	10.757468	8.715827
C	14.890322	9.861078	9.028295
H	15.073192	9.732546	7.947738
H	15.355614	10.822787	9.316309
C	15.540642	8.722195	9.821048
H	15.136112	7.760040	9.461186
H	16.630695	8.704361	9.649418
C	15.235561	8.857898	11.316889
H	15.708327	9.778489	11.708309

H	15.673646	8.014783	11.877977
C	13.723392	8.911985	11.576340
H	13.532957	9.006839	12.655588
H	13.289683	7.952141	11.259646
C	10.951337	10.147608	12.895641
H	11.248372	9.115606	13.152200
C	11.798234	11.107259	13.748162
H	11.540345	12.151074	13.495753
H	12.870784	10.983873	13.531394
C	11.535387	10.877216	15.244379
H	11.869554	9.858540	15.514612
H	12.140767	11.578316	15.843739
C	10.047991	11.026814	15.585302
H	9.877891	10.827929	16.657028
H	9.741053	12.074249	15.406104
C	9.184426	10.094948	14.726980
H	9.402874	9.047608	14.991298
H	8.113497	10.258794	14.936095
C	9.455404	10.303225	13.232438
H	9.124479	11.316780	12.952549
H	8.851459	9.605578	12.630256
C	7.777590	7.816979	10.080882
C	7.112616	7.568103	11.298686
H	7.549482	7.912722	12.241368
C	5.887289	6.888184	11.361864
C	5.280850	6.424673	10.189164
H	4.326846	5.891356	10.231474
C	5.912571	6.655924	8.960500
H	5.450363	6.302253	8.033165
C	7.133867	7.340291	8.915797
H	7.605311	7.511776	7.942654
H	5.405329	6.720749	12.330457
C	10.365299	4.827510	7.671028
C	9.751020	4.921227	9.084018
C	12.003389	5.629202	9.021726
H	10.200136	3.869146	7.168597
H	10.017900	5.649862	7.029778
H	8.848549	5.543937	9.069362
O	11.781893	4.976490	7.868789
N	10.823054	5.682955	9.763140
O	13.080880	6.072671	9.345314
C	10.697518	6.727062	10.688699
H	11.621626	7.301627	10.697932
C	10.113055	6.528983	12.056443
H	9.862486	7.506952	12.482804

H	9.189003	5.947666	12.048019	C	10.623206	2.616493	9.764246
C	9.383475	3.521969	9.680527	H	10.353455	1.636477	10.190763
C	11.097378	5.889760	13.026619	H	11.077556	2.434795	8.777835
C	10.529505	5.614933	14.437389	H	11.389424	3.065378	10.415977
F	12.190429	6.702995	13.192772	C	8.314036	2.894145	8.761684
F	11.577914	4.702501	12.545875	H	7.987361	1.925823	9.172962
C	11.494043	4.979764	15.399253	H	7.425977	3.544463	8.690588
F	10.088549	6.816483	14.931791	H	8.677668	2.704434	7.740673
F	9.417951	4.830392	14.284074	C	8.756431	3.640347	11.079194
H	10.985399	4.838851	16.363510	H	9.497097	3.899801	11.841304
H	11.821223	4.008054	15.006700	H	7.943914	4.384514	11.098744
H	12.366946	5.632524	15.531317	H	8.324784	2.670482	11.375729

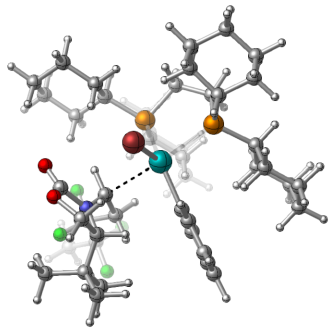

Zero-point correction= 1.093287 (Hartree/Particle)
Thermal correction to Energy= 1.152384
Thermal correction to Enthalpy= 1.153328
Thermal correction to Gibbs Free Energy= 0.996637
Sum of electronic and zero-point Energies= -6841.604416
Sum of electronic and thermal Energies= -6841.545319
Sum of electronic and thermal Enthalpies= -6841.544375
Sum of electronic and thermal Free Energies= -6841.701066

uM06L-d3/def2-TZVPP-SMD(THF)
E(scf) = -6845.27344883 a.u.

^{4}TS2-(*R*,*S*)
E(scf) = -6842.69807557 a.u.
ν_{min} = -46.27 cm^{-1}

Br	9.580481	5.679187	9.497755	C	6.744190	8.961821	13.816153
Fe	9.007780	6.164432	11.870556	H	6.775745	7.863619	13.891251
P	8.586419	8.690416	11.687230	H	7.550359	9.350192	14.457800
P	11.143170	6.985776	13.018406	C	5.391156	9.460695	14.340680
C	9.884132	9.563219	12.705177	H	5.249016	9.116920	15.379579
H	10.013160	10.596462	12.342715	H	5.397375	10.566348	14.371792
H	9.492605	9.638328	13.730051	C	4.233431	8.988391	13.455352
C	11.230301	8.825977	12.708266	H	3.273963	9.379571	13.834887
H	11.918298	9.279129	13.439270	H	4.169540	7.885772	13.499488
H	11.718763	8.920073	11.727832	C	4.451342	9.418362	12.001389
C	6.974580	9.371786	12.348283	H	4.422548	10.522410	11.938691
H	7.044829	10.473685	12.307735	H	3.634289	9.044076	11.361397

C	5.796506	8.913748	11.464548
H	5.933210	9.252502	10.425465
H	5.781531	7.812735	11.436749
C	8.752984	9.443970	9.977612
H	8.113881	8.790202	9.357385
C	8.270419	10.897926	9.823076
H	7.231544	11.014912	10.164639
H	8.889749	11.556720	10.458917
C	8.371865	11.359097	8.360005
H	7.665410	10.763822	7.752540
H	8.047927	12.410827	8.277210
C	9.786238	11.187821	7.795470
H	10.471929	11.877044	8.323137
H	9.810882	11.474456	6.730142
C	10.285024	9.750730	7.979823
H	11.325272	9.654469	7.624314
H	9.676737	9.063859	7.363551
C	10.194898	9.314849	9.446990
H	10.858800	9.964238	10.044357
H	10.550106	8.280612	9.557279
C	12.901818	6.521926	12.552969
H	13.520423	7.385735	12.860682
C	13.006293	6.361082	11.022828
H	12.342524	5.539896	10.709110
H	12.640125	7.264223	10.508115
C	14.444009	6.055133	10.584889
H	14.474593	5.914140	9.490814
H	15.090179	6.924772	10.809947
C	14.992100	4.815419	11.300330
H	14.397581	3.936139	10.996807
H	16.036765	4.625167	10.999275
C	14.892438	4.975145	12.821568
H	15.552928	5.799640	13.150703
H	15.249248	4.062662	13.329369
C	13.450147	5.269031	13.258260
H	13.407573	5.376026	14.352508
H	12.834174	4.401575	12.984949
C	11.053426	6.797928	14.874536
H	11.294453	5.733311	15.028996
C	12.051094	7.619947	15.704692
H	11.837218	8.696413	15.581644
H	13.082765	7.457774	15.353195
C	11.935069	7.255179	17.192917
H	12.226590	6.197076	17.325913
H	12.645223	7.856932	17.785301
C	10.504862	7.452749	17.711310
H	10.436504	7.152425	18.770693
H	10.253286	8.529186	17.672014
C	9.490253	6.669347	16.868895
H	9.664630	5.587477	17.000006
H	8.462361	6.871262	17.215512
C	9.616207	7.016846	15.380896
H	9.329171	8.070600	15.230771
H	8.909600	6.418928	14.783753
C	7.094308	5.519194	12.324498
C	6.528333	5.268218	13.592237
H	7.129039	5.416695	14.496700
C	5.208127	4.822388	13.751934
C	4.401786	4.601713	12.629559
H	3.371787	4.252629	12.746594
C	4.934972	4.829195	11.354250
H	4.318452	4.656162	10.465999
C	6.254619	5.278216	11.213165
H	6.647380	5.449676	10.205148
H	4.808174	4.645974	14.755781
C	9.532607	2.096795	9.136174
C	8.634760	2.261787	10.379799
C	10.918067	2.598517	10.879104
H	9.364602	1.164936	8.584204
H	9.429854	2.952847	8.454705
H	7.908985	3.072641	10.216387
O	10.878541	2.076372	9.638039
N	9.627525	2.728803	11.359916
O	11.951315	2.872666	11.446932
C	9.256729	3.457154	12.482913
H	8.190536	3.424131	12.661781
C	10.118539	3.539846	13.702004
H	11.133190	3.866581	13.467425
H	9.669199	4.268637	14.386779
C	7.857968	0.972346	10.796265
C	10.245871	2.246745	14.491092
C	11.148245	2.372562	15.742746
F	10.756324	1.237716	13.724391
F	9.016211	1.818627	14.922840
C	11.300490	1.112409	16.546993
F	12.370697	2.822251	15.321642
F	10.622110	3.374385	16.521775
H	11.951196	1.322379	17.407717
H	10.315738	0.777304	16.898514
H	11.752038	0.330374	15.922447

C	6.887701	0.627608	9.650112	H	9.418126	-0.453936	10.161661
H	7.407701	0.409069	8.704435	H	9.527336	0.051988	11.868142
H	6.295194	-0.264087	9.910331	C	7.017609	1.214259	12.064856
H	6.183437	1.456438	9.466650	H	6.357179	0.349288	12.240814
C	8.827569	-0.192751	11.054153	H	7.639603	1.332061	12.962237
H	8.268481	-1.094422	11.353015	H	6.378697	2.106907	11.965295

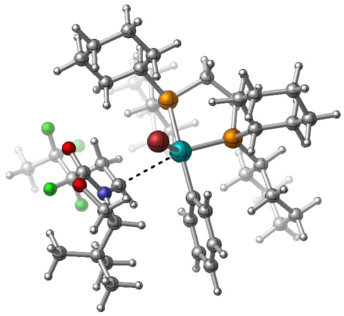

Zero-point correction= 1.093065 (Hartree/Particle)
Thermal correction to Energy= 1.152324
Thermal correction to Enthalpy= 1.153268
Thermal correction to Gibbs Free Energy= 0.996468
Sum of electronic and zero-point Energies= -6841.605011
Sum of electronic and thermal Energies= -6841.545751
Sum of electronic and thermal Enthalpies= -6841.544807
Sum of electronic and thermal Free Energies= -6841.701608

uM06L-d3/def2-TZVPP-SMD(THF)
E(scf) =-6845.27519871 a.u.

^{4}D-(*R*,*S*)
E(scf) = -6842.70409351 a.u.
ν_{min} = 17.08 cm^{-1}

Br	9.892237	5.618324	9.506045	C	4.356794	8.899090	13.901032
Fe	9.172436	5.766606	11.894318	H	3.470091	9.402822	14.322417
P	8.586210	8.304457	11.791534	H	4.198881	7.812997	14.031159
P	11.252062	6.721009	13.044723	C	4.491771	9.203398	12.405645
C	9.918623	9.239501	12.707416	H	4.545401	10.297937	12.255040
H	9.988625	10.264192	12.307150	H	3.600271	8.851292	11.859389
H	9.589447	9.337071	13.751202	C	5.745895	8.540871	11.822606
C	11.281648	8.543192	12.649088	H	5.817318	8.746643	10.743653
H	12.000497	9.041801	13.317999	H	5.645543	7.453127	11.922650
H	11.705871	8.607112	11.636986	C	8.621477	9.072196	10.068697
C	7.018015	8.996499	12.564649	H	8.070175	8.338012	9.455419
H	7.110714	10.092490	12.462975	C	7.944986	10.450219	9.924795
C	6.887392	8.686011	14.068256	H	6.909366	10.437179	10.289491
H	6.855945	7.594549	14.214459	H	8.489734	11.188575	10.541266
H	7.761261	9.057933	14.622903	C	7.942447	10.912042	8.458549
C	5.619290	9.318675	14.660554	H	7.310416	10.219607	7.872479
H	5.536651	9.048679	15.727324	H	7.469798	11.906559	8.384632
H	5.716222	10.419640	14.621329	C	9.349851	10.940830	7.855232

H	9.942598	11.727698	8.358198
H	9.304668	11.215040	6.787296
C	10.053078	9.592634	8.039025
H	11.089456	9.643039	7.663238
H	9.538813	8.818478	7.441111
C	10.057674	9.161599	9.510532
H	10.624314	9.910428	10.091220
H	10.571070	8.197346	9.613772
C	13.035687	6.292635	12.641143
H	13.611746	7.188232	12.940653
C	13.201653	6.089853	11.122239
H	12.579405	5.237310	10.812921
H	12.826963	6.964861	10.567346
C	14.663749	5.817974	10.747706
H	14.739608	5.646363	9.660212
H	15.276150	6.712561	10.970255
C	15.219590	4.616778	11.519958
H	14.657185	3.713821	11.224852
H	16.278228	4.444198	11.259743
C	15.065490	4.824311	13.030492
H	15.689699	5.680699	13.349155
H	15.435487	3.942575	13.581413
C	13.602158	5.087202	13.414419
H	13.530285	5.244886	14.501222
H	13.017827	4.190650	13.167167
C	11.155058	6.627226	14.911616
H	11.451941	5.588429	15.128458
C	12.112939	7.549317	15.683744
H	11.843089	8.603681	15.496789
H	13.149794	7.419354	15.334859
C	12.025771	7.272574	17.192507
H	12.379497	6.243825	17.388967
H	12.704253	7.949920	17.738572
C	10.590400	7.421060	17.711811
H	10.545172	7.179663	18.787336
H	10.279100	8.477853	17.612771
C	9.615782	6.535898	16.925268
H	9.848836	5.474357	17.115147
H	8.580560	6.702129	17.269033
C	9.713646	6.807139	15.419358
H	9.385983	7.838374	15.222465
H	9.021308	6.153744	14.862294
C	7.190006	5.357652	11.995804
C	6.498288	5.127441	13.198002
H	7.039290	5.144552	14.150609
C	5.122451	4.860984	13.213330
C	4.408834	4.789129	12.011262
H	3.336882	4.573218	12.017113
C	5.085388	4.988178	10.801390
H	4.540461	4.926848	9.854085
C	6.456594	5.275727	10.797184
H	6.966554	5.446648	9.845051
H	4.608524	4.701120	14.166273
C	9.668504	2.098531	9.179137
C	8.724125	2.365322	10.372286
C	10.983688	2.634854	10.961196
H	9.488478	1.142810	8.673045
H	9.623309	2.914017	8.443386
H	8.061579	3.212090	10.142572
O	10.988000	2.060220	9.737283
N	9.691454	2.808666	11.386933
O	12.010917	2.901918	11.549587
C	9.300422	3.662411	12.475805
H	8.277424	3.400869	12.727911
C	10.138474	3.563877	13.739028
H	11.180919	3.828917	13.560820
H	9.722373	4.267973	14.468299
C	7.831030	1.141507	10.757216
C	10.143905	2.221335	14.442831
C	10.948406	2.222814	15.766762
F	10.665964	1.227710	13.663264
F	8.866272	1.828879	14.763779
C	11.027650	0.893768	16.464393
F	12.204878	2.690595	15.486713
F	10.369118	3.156173	16.590902
H	11.598487	1.020130	17.395277
H	10.014826	0.537895	16.695205
H	11.531931	0.166343	15.815108
C	6.917468	0.843402	9.551591
H	7.481430	0.573260	8.645205
H	6.247963	-0.000933	9.782258
H	6.286084	1.715262	9.310640
C	8.692014	-0.085581	11.096886
H	8.050719	-0.936417	11.380422
H	9.310746	-0.411788	10.245763
H	9.361779	0.128757	11.942893
C	6.919239	1.462880	11.956378
H	6.181440	0.654135	12.088817
H	7.481795	1.541205	12.895142
H	6.362812	2.400809	11.803206

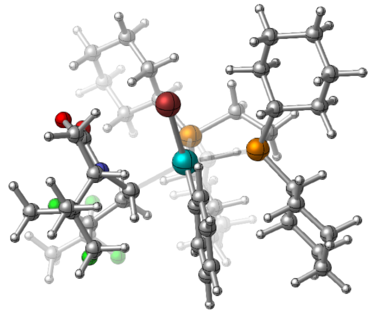

Zero-point correction= 1.095806 (Hartree/Particle)
Thermal correction to Energy= 1.154802
Thermal correction to Enthalpy= 1.155746
Thermal correction to Gibbs Free Energy= 1.001793
Sum of electronic and zero-point Energies= -6841.608288
Sum of electronic and thermal Energies= -6841.549292
Sum of electronic and thermal Enthalpies= -6841.548348
Sum of electronic and thermal Free Energies= -6841.702300

uM06L-d3/def2-TZVPP-SMD(THF)
E(scf) = -6845.28345259 a.u.

^{4}D-(*R*,*R*)
E(scf) = -6842.70609068 a.u.
ν_{min} = 20.37cm^{-1}

Br	10.617598	8.332521	7.579067
Fe	9.704985	8.618075	9.906185
P	8.656492	10.925356	9.375917
P	11.337736	10.097264	11.124375
C	9.585339	12.220743	10.352916
H	9.507290	13.188665	9.831717
H	9.054786	12.341333	11.306467
C	11.054391	11.863280	10.609532
H	11.493775	12.538401	11.360979
H	11.645853	11.990251	9.691972
C	6.875423	11.318557	9.815904
H	6.751740	12.392911	9.590314
C	6.564500	11.119684	11.311597
H	6.737656	10.066026	11.579454
H	7.236774	11.722491	11.939957
C	5.109451	11.490834	11.632749
H	4.909544	11.307012	12.702300
H	4.968806	12.575366	11.467797
C	4.116189	10.716024	10.760560
H	3.082241	11.030227	10.983621
H	4.182988	9.640120	11.004417
C	4.428742	10.906633	9.272869
H	4.263127	11.964242	8.994428
H	3.740844	10.304875	8.654921
C	5.878008	10.515267	8.958814
H	6.080974	10.646155	7.884566
H	6.009659	9.445507	9.169985
C	8.841000	11.510976	7.596603
H	8.557307	10.620415	7.009126
C	7.951306	12.696313	7.173843
H	6.889085	12.499587	7.373295
H	8.226329	13.588292	7.766145
C	8.122664	13.004050	5.677217
H	7.746009	12.142558	5.095614
H	7.493133	13.867731	5.402137
C	9.584391	13.264053	5.299969
H	9.920783	14.201113	5.781970
H	9.677824	13.419176	4.211443
C	10.484975	12.114284	5.762267
H	11.542947	12.341335	5.545546
H	10.237970	11.196094	5.199419
C	10.310961	11.839572	7.260475
H	10.616266	12.742659	7.817426
H	10.968184	11.016150	7.568514
C	13.189185	9.936981	10.891732
H	13.606296	10.895618	11.253467
C	13.530057	9.785331	9.395683
H	13.062408	8.863381	9.017131
H	13.098649	10.611854	8.808170
C	15.045003	9.717480	9.167192

H	15.248260	9.580327	8.091319
H	15.505950	10.681005	9.456468
C	15.677574	8.584201	9.981688
H	15.275029	7.619823	9.625956
H	16.770456	8.561464	9.829359
C	15.345652	8.736065	11.470063
H	15.813616	9.659888	11.860294
H	15.772203	7.897986	12.047397
C	13.829048	8.792882	11.701647
H	13.622301	8.903937	12.776487
H	13.399054	7.831096	11.388132
C	11.059902	10.048777	12.971350
H	11.354185	9.020118	13.242481
C	11.909727	11.018657	13.809984
H	11.650093	12.059321	13.546333
H	12.981678	10.893941	13.592915
C	11.651591	10.802027	15.309265
H	11.989037	9.786531	15.586859
H	12.258217	11.509573	15.899658
C	10.165565	10.951927	15.656058
H	10.000893	10.758298	16.729555
H	9.856930	11.998195	15.473105
C	9.297788	10.015757	14.806538
H	9.513467	8.969261	15.076070
H	8.227867	10.184660	15.016802
C	9.566193	10.216644	13.310790
H	9.246442	11.233621	13.031586
H	8.952401	9.526303	12.709436
C	7.887100	7.724143	10.019208
C	7.148448	7.540401	11.200924
H	7.562873	7.853706	12.162983
C	5.869920	6.963587	11.190108
C	5.302309	6.538080	9.984974
H	4.309711	6.079393	9.972644
C	6.019188	6.710156	8.793920
H	5.584757	6.388678	7.842245
C	7.286990	7.303293	8.814708
H	7.821772	7.452835	7.872510
H	5.319911	6.842881	12.128432
C	10.731397	4.784244	7.696497
C	9.877181	5.015832	8.959512
C	12.126004	5.587517	9.294894
H	10.595257	3.801292	7.232725
H	10.561088	5.571372	6.948166
H	9.046495	5.692908	8.738928
O	12.090580	4.872526	8.150317
N	10.849897	5.740944	9.798883
O	13.162923	6.002614	9.769352
C	10.623831	6.870946	10.673883
H	11.616286	7.318112	10.778513
C	10.099633	6.572538	12.070215
H	9.897907	7.517681	12.586864
H	9.152759	6.030479	12.052772
C	9.284222	3.684123	9.530171
C	11.064025	5.813162	12.962058
C	10.538510	5.545212	14.392099
F	12.238307	6.507386	13.107239
F	11.400266	4.596768	12.432316
C	11.463536	4.751842	15.271898
F	10.279437	6.767650	14.961456
F	9.329621	4.911779	14.275689
H	10.995188	4.636466	16.259685
H	11.639931	3.763968	14.826826
H	12.419747	5.281822	15.373731
C	10.392360	2.656207	9.814491
H	9.960134	1.740021	10.250115
H	10.937758	2.361270	8.904568
H	11.120535	3.057834	10.535211
C	8.298787	3.136654	8.476272
H	7.848434	2.195598	8.831030
H	7.479677	3.852997	8.296332
H	8.778459	2.921966	7.509018
C	8.481423	3.916108	10.820508
H	9.135914	4.083035	11.682565
H	7.790887	4.768017	10.724140
H	7.878063	3.022404	11.050052

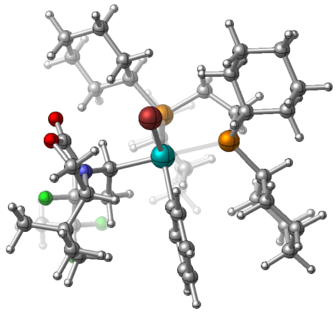

Zero-point correction= 1.096318 (Hartree/Particle)
Thermal correction to Energy= 1.154934
Thermal correction to Enthalpy= 1.155878
Thermal correction to Gibbs Free Energy= 1.003865
Sum of electronic and zero-point Energies= -6841.609772
Sum of electronic and thermal Energies= -6841.551157
Sum of electronic and thermal Enthalpies= -6841.550213
Sum of electronic and thermal Free Energies= -6841.702225

uM06L-d3/def2-TZVPP-SMD(THF)
E(scf) = -6845.28277550 a.u.

[4]TS3-(*R*,*R*)
E(scf) = -6842.68206747 a.u.
ν_{min} = -286.78 cm^{-1}

Br	10.523643	8.364000	7.489113
Fe	9.561378	8.912703	9.750499
P	8.620695	11.196949	9.303602
P	11.218147	10.211758	11.114538
C	9.480191	12.411798	10.433423
H	9.447165	13.419981	9.988520
H	8.874421	12.458040	11.348971
C	10.929283	12.029185	10.783158
H	11.281812	12.616563	11.646326
H	11.596141	12.280321	9.946940
C	6.810211	11.543664	9.606739
H	6.647293	12.622496	9.433551
C	6.395896	11.223289	11.055720
H	6.618200	10.164614	11.264419
H	6.980005	11.819704	11.773884
C	4.898801	11.478062	11.278915
H	4.627958	11.211268	12.314956
H	4.694954	12.559498	11.168410
C	4.036169	10.695374	10.282948
H	2.967901	10.926307	10.435168
H	4.160341	9.614524	10.471547
C	4.448984	10.995657	8.838303
H	4.222498	12.052589	8.603918
H	3.859162	10.383131	8.135167
C	5.945278	10.740148	8.615977
H	6.213942	10.981242	7.575165
H	6.156411	9.666607	8.752778
C	8.951202	11.849050	7.578257
H	8.613072	11.019551	6.932006
C	8.202912	13.132086	7.176539
H	7.115969	13.019779	7.303853
H	8.516427	13.960234	7.838479
C	8.499800	13.507013	5.715522
H	8.081703	12.722616	5.057995
H	7.977604	14.444333	5.457105
C	10.002644	13.638691	5.445297
H	10.397117	14.503292	6.011521
H	10.182360	13.853808	4.377976
C	10.755278	12.375151	5.875879
H	11.841679	12.504550	5.731525
H	10.452090	11.524815	5.238501
C	10.463787	12.025755	7.339577
H	10.837296	12.847107	7.976290
H	11.000393	11.110253	7.623813
C	13.062447	10.062812	10.844167
H	13.535963	10.900822	11.389155
C	13.394642	10.194884	9.343445
H	12.838235	9.422111	8.787544
H	13.053426	11.166197	8.951404
C	14.896616	10.034199	9.075920
H	15.086682	10.104572	7.991199
H	15.446776	10.869966	9.548302

C	15.424181	8.706376	9.628572
H	14.939273	7.872210	9.090331
H	16.509611	8.616474	9.450169
C	15.111400	8.580680	11.122707
H	15.668373	9.357802	11.679807
H	15.456545	7.606322	11.508935
C	13.609077	8.733519	11.395602
H	13.421544	8.653342	12.476284
H	13.078276	7.893864	10.919708
C	10.950186	10.001498	12.951291
H	11.136702	8.926173	13.111318
C	11.882586	10.778657	13.893569
H	11.743079	11.864303	13.743486
H	12.938142	10.559242	13.669717
C	11.578033	10.427461	15.359001
H	11.802910	9.357707	15.523247
H	12.243334	10.998321	16.028979
C	10.109189	10.690116	15.713568
H	9.909405	10.398904	16.758796
H	9.910521	11.776116	15.645218
C	9.163215	9.946176	14.762714
H	9.269695	8.859602	14.917123
H	8.112805	10.198646	14.987460
C	9.474909	10.280487	13.299485
H	9.260166	11.349211	13.128589
H	8.810102	9.719882	12.623094
C	8.066448	7.626183	10.186493
C	7.215601	7.665261	11.321444
H	7.606518	7.980219	12.289768
C	5.859891	7.336791	11.239099
C	5.287982	6.956977	10.018345
H	4.227176	6.701740	9.955807
C	6.101198	6.923369	8.877526
H	5.675038	6.644785	7.908860
C	7.459473	7.238504	8.959273
H	8.066575	7.205764	8.052968
H	5.240550	7.394289	12.139324
C	9.878612	4.560509	7.762903
C	9.204832	4.667241	9.139086
C	11.398337	5.501452	9.148426
H	9.753877	3.594001	7.262700
H	9.549383	5.371871	7.093407
H	8.232653	5.159097	9.053060
O	11.277650	4.744277	8.037808
N	10.163992	5.592328	9.781562
O	12.446395	5.981073	9.512200
C	9.953582	6.701269	10.655970
H	10.863884	7.304603	10.558667
C	9.739252	6.444314	12.135290
H	9.581382	7.409885	12.629225
H	8.852091	5.836988	12.337216
C	8.961362	3.287025	9.831037
C	10.923167	5.815328	12.848590
C	10.698367	5.570543	14.360815
F	12.027190	6.617552	12.748333
F	11.271990	4.609717	12.305348
C	11.844985	4.907713	15.071623
F	10.430879	6.790339	14.932998
F	9.557964	4.824917	14.493312
H	11.584601	4.804637	16.134591
H	12.026909	3.916616	14.636135
H	12.748879	5.522431	14.968727
C	10.255691	2.465742	9.949704
H	10.055306	1.513029	10.467521
H	10.686121	2.224187	8.965689
H	11.015894	3.007210	10.531451
C	7.929023	2.525664	8.973642
H	7.698742	1.551606	9.434287
H	6.984577	3.090490	8.895400
H	8.288009	2.325086	7.952507
C	8.347954	3.473909	11.228550
H	9.087130	3.826417	11.954414
H	7.503034	4.181804	11.209311
H	7.966775	2.510044	11.603838

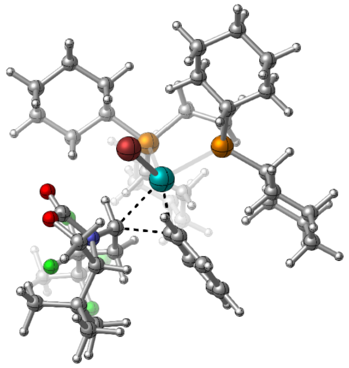

Zero-point correction= 1.094204 (Hartree/Particle)
Thermal correction to Energy= 1.152661
Thermal correction to Enthalpy= 1.153605
Thermal correction to Gibbs Free Energy= 1.000656
Sum of electronic and zero-point Energies= -6841.587864
Sum of electronic and thermal Energies= -6841.529406
Sum of electronic and thermal Enthalpies= -6841.528462
Sum of electronic and thermal Free Energies= -6841.681411

uM06L-d3/def2-TZVPP-SMD(THF)
E(scf) = -6845.25624834 a.u.

[4]TS3-(*R*,*S*)
E(scf) = -6842.68277840 a.u.
ν_{min} = -263.65 cm^{-1}

Br	9.651273	5.757071	9.415548
Fe	9.118442	6.135434	11.826267
P	8.630576	8.603589	11.775452
P	11.158566	6.897279	13.102434
C	9.905312	9.481089	12.817759
H	10.036798	10.516008	12.461220
H	9.489845	9.550982	13.832610
C	11.256030	8.748390	12.851801
H	11.910700	9.183317	13.623745
H	11.781169	8.875974	11.894418
C	6.999086	9.235439	12.432281
H	7.019698	10.337199	12.357056
C	6.776766	8.864947	13.910803
H	6.822191	7.768933	14.017411
H	7.575022	9.281489	14.544255
C	5.415519	9.366709	14.412778
H	5.273068	9.061773	15.463819
H	5.411897	10.472716	14.402491
C	4.263242	8.848656	13.545408
H	3.301399	9.256984	13.900230
H	4.201855	7.750934	13.646126
C	4.481949	9.197305	12.069553
H	4.429202	10.294486	11.939649
H	3.676817	8.766622	11.450131
C	5.842172	8.695004	11.568823
H	5.977305	8.975423	10.511871
H	5.859684	7.593348	11.603763
C	8.796675	9.390231	10.080416
H	8.217406	8.706616	9.434761
C	8.235848	10.815171	9.926933
H	7.180636	10.865541	10.233695
H	8.793081	11.500998	10.591408
C	8.357429	11.301183	8.473446
H	7.705550	10.674990	7.836734
H	7.976858	12.333958	8.392234
C	9.796251	11.217422	7.952506
H	10.425017	11.936842	8.510042
H	9.837764	11.519863	6.892039
C	10.369156	9.808044	8.135404
H	11.423493	9.774838	7.811147
H	9.819506	9.096023	7.493423
C	10.258907	9.348060	9.593538
H	10.869461	10.022685	10.219328
H	10.664110	8.332080	9.700606
C	12.906624	6.411718	12.634172
H	13.563354	7.218576	13.010939
C	13.022170	6.351179	11.097458
H	12.301532	5.608353	10.721003

H	12.736782	7.316043	10.647121	H	7.014127	4.941093	10.173811
C	14.436046	5.964172	10.647527	H	4.930775	5.193629	14.686301
H	14.468232	5.901052	9.546155	C	9.103957	2.187140	9.084207
H	15.149430	6.758172	10.939915	C	8.280649	2.079960	10.378123
C	14.871124	4.634909	11.273725	C	10.484185	2.792625	10.784438
H	14.204323	3.834174	10.908582	H	9.026816	1.314539	8.426178
H	15.897260	4.375533	10.960445	H	8.852773	3.100543	8.524556
C	14.775478	4.699254	12.801816	H	7.359948	2.672511	10.299496
H	15.500518	5.443068	13.183725	O	10.463601	2.292020	9.531230
H	15.054929	3.729139	13.247897	N	9.205837	2.753846	11.309758
C	13.361143	5.080909	13.260546	O	11.504181	3.158762	11.324610
H	13.332612	5.130974	14.358702	C	8.764110	3.405322	12.480601
H	12.672061	4.282860	12.953463	H	7.836101	2.950970	12.810287
C	11.062878	6.664859	14.954418	C	9.726480	3.564321	13.638975
H	11.176339	5.575538	15.076701	H	10.696511	3.938701	13.310567
C	12.153865	7.342346	15.799226	H	9.301740	4.279897	14.352229
H	12.066533	8.439548	15.707386	C	7.880142	0.619493	10.766859
H	13.158736	7.072790	15.438348	C	9.961047	2.286380	14.424263
C	12.005036	6.949787	17.277746	C	10.952638	2.428820	15.603996
H	12.169839	5.861100	17.375497	F	10.440136	1.284191	13.627763
H	12.785794	7.444867	17.880095	F	8.779011	1.827280	14.952170
C	10.612352	7.298566	17.817861	C	11.153747	1.180467	16.416706
H	10.516444	6.975207	18.868383	F	12.143719	2.857826	15.087145
H	10.490099	8.397941	17.814037	F	10.494351	3.446991	16.404579
C	9.505862	6.668585	16.962478	H	11.876265	1.396776	17.216398
H	9.544766	5.570890	17.064259	H	10.197474	0.865303	16.854175
H	8.512336	6.987102	17.322075	H	11.542812	0.381164	15.772264
C	9.667420	7.041746	15.484090	C	6.916289	0.099704	9.682395
H	9.517825	8.128919	15.374150	H	7.384644	0.051760	8.687100
H	8.886993	6.560888	14.875591	H	6.578159	-0.918591	9.932934
C	7.424912	5.103630	12.303251	H	6.021341	0.739986	9.605226
C	6.773890	5.176156	13.567105	C	9.114162	-0.292914	10.861653
H	7.370334	5.251407	14.480927	H	8.810094	-1.314078	11.144529
C	5.384820	5.126250	13.692593	H	9.655793	-0.362298	9.905647
C	4.569524	4.999559	12.558259	H	9.815148	0.070249	11.627664
H	3.481608	4.960077	12.656427	C	7.130853	0.592745	12.111729
C	5.178204	4.931584	11.295633	H	6.717503	-0.413996	12.287820
H	4.559584	4.846113	10.396420	H	7.787534	0.822951	12.960474
C	6.567803	4.979740	11.169940	H	6.288642	1.304510	12.120587

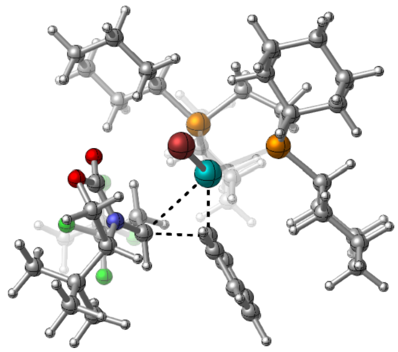

Zero-point correction= 1.093486 (Hartree/Particle)
Thermal correction to Energy= 1.152338
Thermal correction to Enthalpy= 1.153283
Thermal correction to Gibbs Free Energy= 0.998706
Sum of electronic and zero-point Energies= -6841.589293
Sum of electronic and thermal Energies= -6841.530440
Sum of electronic and thermal Enthalpies= -6841.529496
Sum of electronic and thermal Free Energies= -6841.684073

uM06L-d3/def2-TZVPP-SMD(THF)
E(scf) = -6845.25781426 a.u.

P-(*R*,*R*)
$E(scf)_{gas}$ = -1303.49584910 a.u.
ν_{min} = 28.72 cm^{-1}

C	8.118608	7.122962	10.350866
C	6.908343	6.957438	11.038208
H	6.877391	6.409482	11.981641
C	5.717592	7.486201	10.523354
C	5.721680	8.185484	9.314116
H	4.792146	8.598289	8.913343
C	6.925984	8.356781	8.619746
H	6.941450	8.907753	7.675547
C	8.110996	7.828913	9.135282
H	9.048658	7.964320	8.588083
H	4.782763	7.346423	11.072792
C	9.416899	4.365670	7.926713
C	8.897466	4.381271	9.375646
C	10.926163	5.538926	9.147393
H	9.388679	3.384341	7.440781
H	8.876580	5.092784	7.297510
H	7.866036	4.761490	9.406614
O	10.789906	4.778355	8.032784
N	9.810662	5.389279	9.945102
O	11.891330	6.237715	9.349616
C	9.438678	6.516207	10.819705
H	10.226261	7.259570	10.632753
C	9.474458	6.235340	12.325929
H	9.161291	7.147303	12.853429
H	8.784841	5.440067	12.623462
C	8.913216	2.975485	10.059072
C	10.849027	5.894777	12.860276
C	10.880459	5.645932	14.387654
F	11.729312	6.905859	12.596785
F	11.362639	4.768037	12.274770
C	12.238293	5.325150	14.947055
F	10.364287	6.759026	14.993888
F	10.003844	4.623341	14.646285
H	12.140468	5.161582	16.029665
H	12.630880	4.418422	14.468565
H	12.922471	6.162931	14.758665
C	10.313151	2.340358	10.002785
H	10.306034	1.363072	10.512762
H	10.656066	2.171793	8.970310
H	11.053732	2.975816	10.511792
C	7.894666	2.085181	9.320295
H	7.856490	1.086842	9.784637
H	6.879991	2.515533	9.369663
H	8.148096	1.939975	8.258771
C	8.474079	3.073255	11.528699
H	9.249737	3.531187	12.153143
H	7.540786	3.650230	11.637163
H	8.289969	2.065098	11.934007

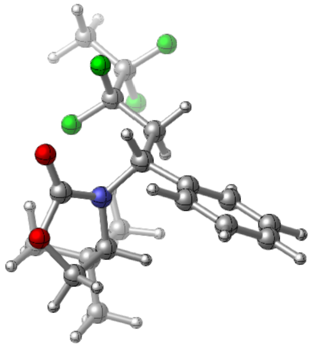

Zero-point correction= 0.391296 (Hartree/Particle)
Thermal correction to Energy= 0.415250
Thermal correction to Enthalpy= 0.416194
Thermal correction to Gibbs Free Energy= 0.337835
Sum of electronic and zero-point Energies= -1303.104553
Sum of electronic and thermal Energies= -1303.080599
Sum of electronic and thermal Enthalpies= -1303.079655
Sum of electronic and thermal Free Energies= -1303.158014

uM06L-d3/def2-TZVPP-SMD(THF)
E(scf) = -1304.73374953 a.u.

P-(*R*,*S*)
E(scf) = -1303.49995877 a.u.
ν_{min} = 28.72 cm^{-1}

C	7.161754	4.310645	12.520595
C	7.434676	5.582277	13.043561
H	8.402389	5.772680	13.517130
C	6.483946	6.607024	12.964777
C	5.246346	6.367361	12.361260
H	4.501619	7.165310	12.298193
C	4.964491	5.099228	11.837538
H	3.998700	4.905424	11.362972
C	5.914283	4.077728	11.915469
H	5.685954	3.091318	11.505974
H	6.712021	7.593177	13.378244
C	6.447027	1.524765	15.342798
C	7.735310	2.289769	14.984845
C	6.841005	1.151043	13.133947
H	6.516352	0.919687	16.254019
H	5.584708	2.206057	15.432207
H	7.619164	3.357799	15.230206
O	6.223239	0.644624	14.230785
N	7.728552	2.124816	13.522558
O	6.594311	0.750995	12.017309
C	8.201365	3.199242	12.634698
H	9.082218	3.628696	13.123552
C	8.653882	2.717252	11.245354
H	7.858432	2.163954	10.736482
H	8.899517	3.602988	10.643642
C	9.020280	1.765679	15.699489
C	9.891268	1.848462	11.247313
C	10.371416	1.439278	9.833684
F	9.710077	0.694391	11.955738
F	10.939845	2.505326	11.846677
C	11.592360	0.561628	9.812058
F	9.323629	0.814215	9.215483
F	10.602012	2.594443	9.135427
H	11.846214	0.344767	8.764759
H	12.430047	1.080092	10.296816
H	11.383508	-0.375539	10.344330
C	8.857642	2.004797	17.212163
H	8.016559	1.438642	17.641530
H	9.768353	1.689773	17.746302
H	8.693683	3.073300	17.432525
C	9.236828	0.269894	15.415970
H	10.151245	-0.083770	15.919628
H	8.401185	-0.349143	15.778403
H	9.356428	0.088264	14.337384
C	10.255158	2.549654	15.222305
H	11.138405	2.256568	15.812973
H	10.490031	2.349014	14.169214
H	10.117959	3.636662	15.349095

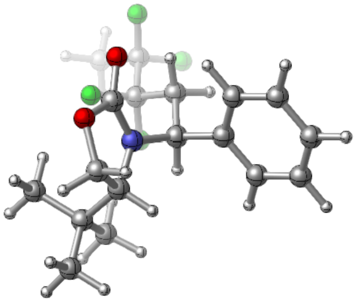

Zero-point correction= 0.391073 (Hartree/Particle)
Thermal correction to Energy= 0.415157
Thermal correction to Enthalpy= 0.416101
Thermal correction to Gibbs Free Energy= 0.337227
Sum of electronic and zero-point Energies= -1303.108885
Sum of electronic and thermal Energies= -1303.084802
Sum of electronic and thermal Enthalpies= -1303.083858
Sum of electronic and thermal Free Energies= -1303.162732

uM06L-d3/def2-TZVPP-SMD(THF)
E(scf) = -1304.73560508 a.u.
